# High-resolution global diversity copy number variation maps and association with ctyper

**DOI:** 10.1101/2024.08.11.607269

**Published:** 2024-08-11

**Authors:** Walfred Ma, Mark JP Chaisson

**Affiliations:** 1. Quantitative and Computational Biology, University of Southern California, CA, USA.; 2. The Genomic and Epigenomic Regulation Program, USC Norris Cancer Center, University of Southern California, Los Angeles, California 90033, USA

## Abstract

Genetic analysis of copy number variations (CNVs), especially in complex regions, is challenging due to reference bias and ambiguous alignment of Next-Generation Sequencing (NGS) reads to repetitive DNA. Consequently, aggregate copy numbers are typically analyzed, overlooking variation between gene copies.

Pangenomes contain diverse sequences of gene copies and enable the study of sequence-resolved CNVs. We developed a method, ctyper, to discover sequence-resolved CNVs in NGS data by leveraging CNV genes from pangenomes. From 118 public assemblies, we constructed a database of 3,351 CNV genes, distinguishing each gene copy as a resolved allele. We used phylogenetic trees to organize alleles into highly similar allele-types that revealed events of linked small variants due to stratification, structural variation, conversion, and duplication. Saturation analysis showed that new samples share an average of 97.8% CNV alleles with the database. The ctyper method traces individual gene copies in NGS data to their nearest alleles in the database and identifies allele-specific copy numbers using multivariate linear regression on k-mer counts and phylogenetic clustering. Applying ctyper to 1000 Genomes Project (1kgp) samples showed Hardy-Weinberg Equilibrium on 99.3% of alleles and a 97.6% F1 score on genotypes based on 641 1kgp trios. Leave-one-out analysis on 39 assemblies matched to 1kgp samples showed that 96.5% of variants in query sequences match the genotyped allele. Genotyping 1kgp data revealed 226 population-specific CNVs, including a conversion on SMN2 to SMN1, potentially impacting Spinal Muscular Atrophy diagnosis in Africans.

Our results revealed two models of CNV: recent CNVs due to ongoing duplications and polymorphic CNVs from ancient paralogs missing from the reference. To measure the functional impact of CNVs, after merging allele-types, we conducted genome-wide Quantitative Trait Locus analysis on 451 1kgp samples with Geuvadis rRNA-seqs. Using a linear mixed model, our genotyping enables the inference of relative expression levels of paralogs within a gene family. In a global evolutionary context, 150 out of 1,890 paralogs (7.94%) and 546 out of 16,628 orthologs (3.28%) had significantly different expression levels, suggesting divergent expression from original genes. Specific examples include lower expression on the converted *SMN* and increased expression on translocated *AMY2B* (GTEx pancreas data). Our method enables large cohort studies on complex CNVs to uncover hidden health impacts and overcome reference bias.

## Introduction

Human genomes are characterized by frequent duplications and deletions, leading to copy number variations (CNVs). Such variation impacts 5–10% of protein-coding genes and displays distinct distributions across human populations^1–3^. A wide range of traits are associated with CNVs including body mass index^4^, various cancers^5^, cardiovascular diseases^6^, diabetes^7^, and neurodevelopmental disorders^3,8,9^.

We consider only CNVs on genes. While CNVs are infrequent for most genes, genomic regions with long, low-copy repeats called segmental duplications (SDs) are enriched in genes and are catalysts for frequent CNVs^10,11^. This leads to regions with high structural heterozygosity including *TBC1D3*, *NPIP*, and *NBPF*^12,13^ genes. The diverse mechanisms contributing to CNVs, along with the elevated mutations in SD regions, result in variation not only in aggregate copy number (aggreCN) but also a high degree of sequence variation among the copies themselves^1,14–16^. This variation can influence phenotypes and disease susceptibility^17–20^, such as hypertension, and type 2 diabetes^21^.

Despite years of study, there is scarce information about variation in non-reference gene duplicates, particularly in studies using short-read next-generation sequencing (NGS) data. Most CNV calling tools depend on aligning NGS reads to a single genome reference^22–24^, and solely detect excess coverage rather than sequence variants^25–27^. Furthermore, NGS alignment to a single reference genome contains ambiguity and bias^28^.

Advances in single-molecule sequencing have enabled generation of pangenomes from diverse populations with sequence-resolved CNVs^29–31^. Although reference bias may be reduced by organizing pangenomes as a graph^32^, the variants distinguishing paralogs may be obscured by merged paralogous genes in the graph^33^. Furthermore, as pangenomes grow, diversity among populations, frequent gene conversion, and genome rearrangements present an even greater challenge^16^.

Here, we developed an alignment-free approach to genotype sequence-resolved copy-number variation and overcome the limitations of alignments on repetitive DNA in pangenomes. This entails two challenges: annotating sequences orthologous and paralogous copies of a given gene and organizing into functionally equivalent groups, and genotyping sequence composition with estimated copy-number on these groups. We leverage the pangenome to represent complex genetic architectures that may not be simply represented as single variants and sidestep the limitations of traditional NGS alignment. As a result, we have developed a method, ctyper, that can sequence-resolved CNVs using NGS data and a pangenome reference.

## Results

### Pangenome annotation and representation of pangenome-alleles

**Figure 1.**
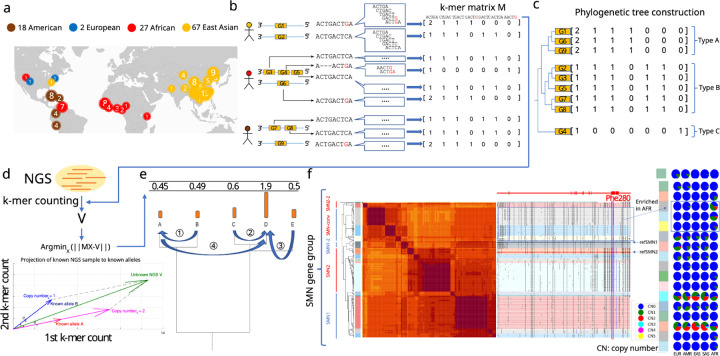
**a,** The demographic composition of the reference pangenome assemblies, HPRC (46 diploid), CPC (57 diploid), HGSVC (9 diploid), T2T-YAO (1 diploid), and CN1, as well as GRCh38 and CHM13. **b,** An overview of the approach to construct pangenome *k*-mer matrices for CNV genes. Each individual gene is represented as a vector of counts of *k*-mers exclusively found within the gene-group. All copies of genes including paralogs and orthologs are included and integrated as a *k*-mer matrix. **c,** Construction of phylogenetic trees based on *k*-mer matrices. The cosine distance is determined for each pair of genes. A phylogenetic tree is then constructed based on pairwise distances via UPGMA. **d,** Schematic of approach to estimate genotypes of alleles using NGS data. The *k*-mers from each matrix are counted in NGS data and normalized by sequencing depth. The normalized *k*-mer counts are projected to all pangenome genes via multi-variable non-negative linear regression. **e,** Reprojection to an integer solution based on the gene family phylogenetic tree. Starting from the leaves (coefficients of individual genes from the regression), at each hierarchy of phylogenetic tree, the remainder from the last step is used to determine the local optimal integer solution with the least residual to the observation. The integer solutions from all steps and then collected and represented as the final allele-type copy-number. **f,** An illustrative annotation and genotyping results on *SMN1/2* genes using HPRC samples. All *SMN* genes are categorized into 5 major allele-types and 17 sub allele-types. *SMN1*/*SMN2* correspond to the major allele-types of each paralog; *SMN1-2*, a copy of *SMN1* partially converted to *SMN2*; *SMN*-conv: additional converted SMN genes, mostly mapped to the *SMN2* locus, and is found to be enriched in African populations. The GRCh38 assembly includes *SMN1-2* and *SMN2*; SMN2-2: a rare outgroup of *SMN2*. On the right-side of the classification, the phylogenetic tree and heatmap of pairwise similarities are shown along with a mutant plot based on multiple sequence alignment highlighting point differences to *SMN1* in CHM13. Phe-280, the variant found to disrupt splicing of *SMN2* transcripts is highlighted. The genotyping results in 1KG continental populations is shown on the right.

We focused on genes previously annotated as CNV^29,31^. among 230 assemblies including HPRC, HGSVC, and CPC, two *de novo* telomere-to-telomere assemblies^34,35^, GRCh38 and CHM13^36^ (Fig. 1a). To construct databases used for querying genotypes, we annotated sequences with which CNV genes share homology across all assemblies, and extracted those sequences into pangenome alleles (PAs): genic segments with locally phased variants that are combinatorially heritable (Fig. 1b). The PAs sharing homology are further organized as gene-groups. The counts of low-copy *k*-mers (*k*=31) found exclusively in a gene-group are used to represent each PA and are combined by each gene-group into a matrix of *k*-mer counts that is later used for genotyping. Each row of the matrix corresponds to a single PA, and columns contain the counts of each *k*-mer (Methods) (Fig. 1b). The rows in the matrix are ordered by the phylogenetic relationship of PAs. To genotype an NGS sample, ctyper counts all *k*-mers for each gene-group in the NGS reads and identifies the combination of PAs most similar to what is likely in the sample as well as their individual copy number by projecting the NGS *k*-mer counts into the vector-space of the gene-group, and using phylogenetic rounding to determine an integer copy-number (Methods, Figs. 1c–e). As an example, the gene-group for *SMN* contains 178 PAs including copies of *SMN1* and *SMN2* as well as paralogs that have undergone gene conversion^37^ including genes found on the *SMN2* locus but identical to *SMN1* regarding the phe-280, the SNP responsible for dysfunctional exon 7 splicing of *SMN2*^38^ (Fig. 1f).

**Figure 2.**
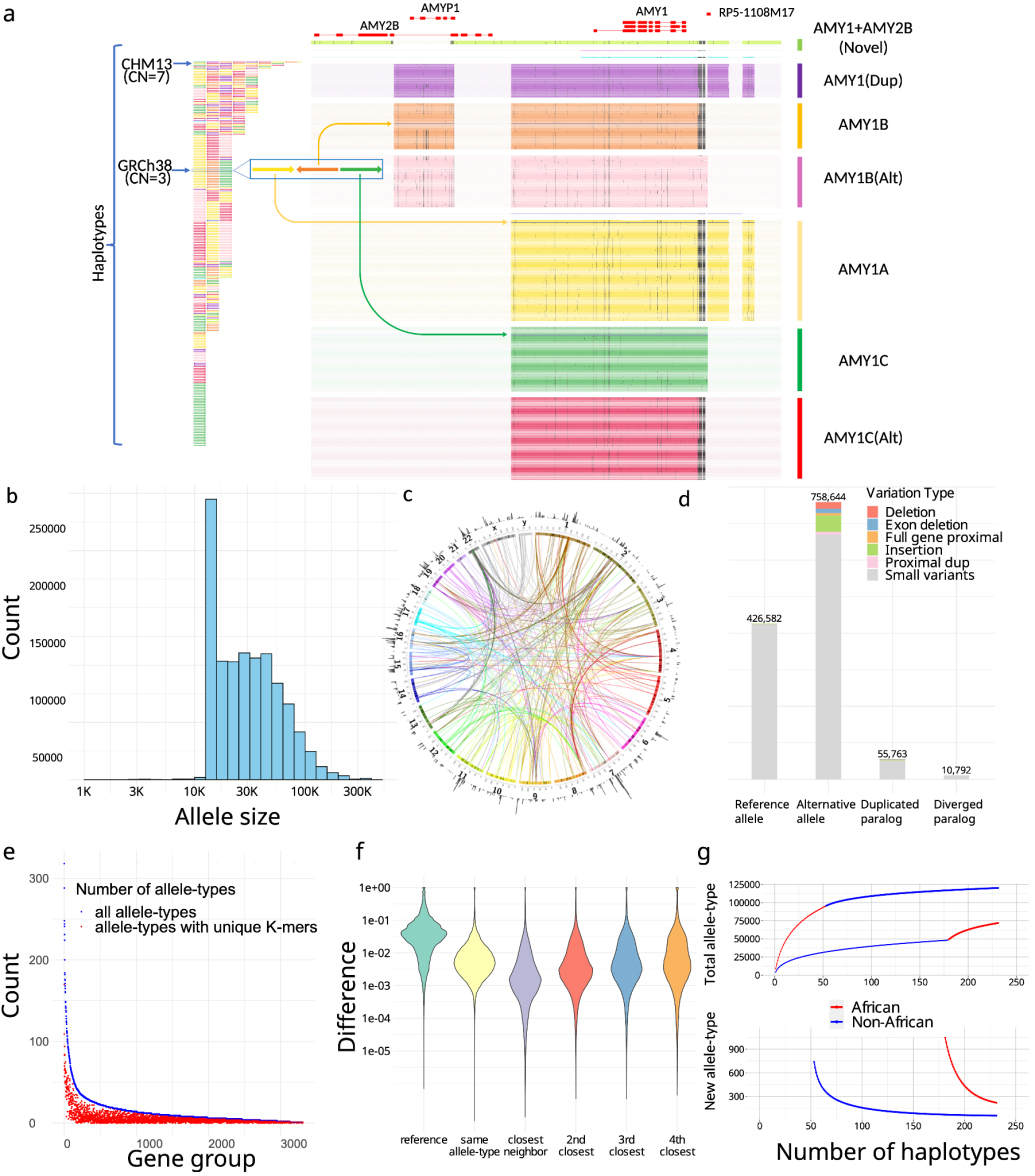
**a,** An example overview of amylase 1 pangenome-alleles (PAs). The left-side shows the corresponding order of all *AMY1* PAs on assemblies, which are colored based on their major allele-types. *AMY1* genes are extracted as PAs as well as their flanking genes and sequences, including an *AMY2B* translocated proximal to *AMY1*, and two pseudogenes: *AMYP1* and *RP5-1108M17*. All PAs are vertically ordered according to the phylogenetic tree and aligned via graphic multiple sequence alignments (gMSA). Homologous sequences are vertically aligned. Mutations are visualized as dots, and large gaps (deletions) are visualized as spaces. Seven major allele-types are categorized including five paralogs and two orthologs. There are no pseudogenes around *AMY1C*, while *AMY1A* has *RP5-1108M17* nearby and *AMY1B* has *AMYP1* nearby. There are alternative versions of *AMY1B and AMY1C*, with sequence substitutions. A new paralog called *AMY1(Dup)* found primarily on haplotypes with duplications, and has both pseudogenes nearby. The paralog of *AMY1* found with translocated *AMY2B* is called *AMY1+AMY2B*. **b,** The size distribution of PAs on a log-density. The minimum sizes of PAs is 15kb, though smaller alleles may be annotated on alternative haplotypes on GRCh38 and as partial loci when dividing large genes into alleles without recombination. **c**. CIRCOS plot of all PAs (outer ring). The density of PAs in each megabase on GRCh38. (arcs) Interchromosomal PAs included in the same groups. **d,** Annotation of PAs according to orthology and variation with respect to GRCh38. Duplicative paralogs are alleles with distal duplications and proximal duplications are included into Alternative alleles due to potential interaction with original genes. **e,** Identifiability of alle-types by unique *k*-mers. The total number of allele-types (blue), and the number of allele-types that may be identified by paralog-specific *k*-mers (red) are shown for each gene group with size at least three. **f,** The distribution of pairwise distances of PAs depending on orthology and phylogenetic relationship. Small neighbor distances are an indicator of strong representativeness of the current cohort.**g,** Saturation analysis for all allele-types using a recapture mode according to two sorted orders: African genomes considered first, and non-African genomes considered first. It estimates that a new African sample shares an average of 94.9% allele-types with the database and a new non-African sample shares an average of 98.7% allele-types with the database.

Overall, 3,351 CNV genes (Supplementary Table 1) were classified into PAs that either contained the entire gene with flanking cis-elements for most genes, or are broken into smaller units not likely to be interrupted by recombination for large genes (Methods). In total, 1,408,209 PAs were defined and organized into 3,307 gene-groups (Figs. 2b–c). The average PA length was 33 ± 29kb, and included full genes (69%), processed pseudogenes (20%), intronic duplications (5%), and decoys (7%) .

We annotated the context of CNVs with respect to corresponding reference genes (Methods). We discovered 164,237 PAs that are distal duplications (>20kb from source gene) on 6,389 loci and 6,673 PAs that contain proximal duplications (<20k from source gene), including 1,646 PAs that have runway duplications (at least three proximal duplications) on 36 genes^39^, with an example on *HPR* (Supplementary Figure 1). We identified 10,792 PAs with diverged paralogs (<80% similarity in k-mer with reference locus) from 333 gene-groups. For example, some amylase PAs contain paralogs of both *AMY1* and *AMY2B*, so not classified as either (Fig. 2a).

Highly similar PAs were merged into 89,236 allele-types to reduce dimensionality of genotypes for population analysis (Methods). Allele-types have a median of 4 members but are skewed to large clusters: 50% PAs are in allele-types with at least 73 members (Supplementary Figure 2). The average pairwise *k*-mer similarity is 94.4% within each allele-type, compared to 78.0% within each gene-group, noting one base change can lead to *k* difference *k*-mers. Between two phylogenetically neighboring allele-types with both having at least three members, the average between-type variance versus within-type variance (F-statistic) is 6.03, showing strong distinctiveness.

The ctyper genotyping estimates the PAs from the pangenome that are closest to genes contained by an NGS sample, along with their copy numbers. Thus the genotype of a gene-group is represented as a vector of PA copy numbers (paCNV). We compared the fine-grained paCNV genotypes to common representations of CNVs: copy-numbers of reference genes^1,39^, single unique nucleotide *k*-mers^1,39,40^ (SUNKs), and large haplotype structures^14,41–43^. First, we characterized the information gained by representing a genome as paCNV compared to copy-numbers of reference alleles. For each PA, we used the nearest neighbor in our pangenome database as a proxy for the optimal genotyping results of samples containing that PA, and its closest GRCH38 genes for comparison of single-reference based CNV. The nearest neighbor demonstrated an average 94.7% reduction in differences compared to GRCh38 matches, and 57.3% had identical nearest neighbors showing paCNVs more closely reflect the genotyped genomes.

We then assessed the proportion of allele-types that may be identified by *k*-mers uniquely contained by all members of an allele-type, analogous to SUNKs. Only 38.8% allele-types (with at least three members) can be represented by such *k*-mers (Fig. 2e). For example, no SUNK is found between *SMN1*, *SMN2* and *SMN-converted* due to gene conversion (Fig. 1f), however there are unique combinations of *k*-mers used by ctyper genotyping.

We investigated the extent to which diversity is represented by novel combinations of alleles versus novel alleles using leave-one-out tests to see if a holdout genome may be represented by allele-types from remaining genomes. For the amylase locus, we found that 40% (90 out of 226) of haplotypes cannot be represented, particularly those with greater copies than GRCH38 (45 out of 67). When all PAs devoid of SV between were combined into a single large allele-type, 20% (46 out of 226) of haplotypes still remained singleton, especially those with additional copies (26 out of 67). Furthermore, many allele-types, such as the novel PAs containing both *AMY1* and *AMY2B* in proximity, are not unique to individual haplotypes (Fig. 2a).

Finally, we performed saturation analysis using a recapture model^44,45^ to estimate the saturation of the current cohort in representing all possible allele-types among worldwide populations,. This model estimates the average number of novel allele-types within each new haplotype. Among the current cohort, each new African haplotype has 221 out of 4363 (5.1%) novel allele-types, and non-Africans have 56 out of 4358 (1.3%).

### Genotyping Pangenome-alleles among NGS samples and Benchmarking results

**Figure 3.**
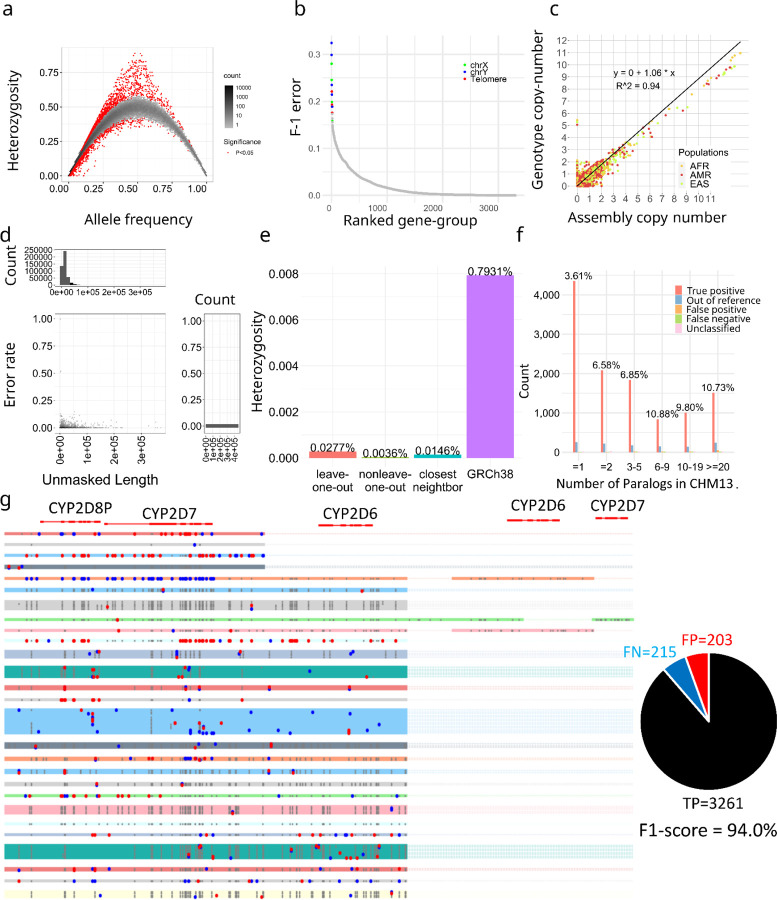
**a,** Hardy-Weinberg equilibrium of genotyping results on 1kgp unrelated samples. **b,** Genotype concordance of genotyping results on 1kgp trios, ordered by F-1 error. The gene groups with F-1 error more than 15% labeled by genomic location. **c,** Copy number comparison between assemblies and genotyping results on 1kgp unrelated samples. **d,** Sequence similarity between genotyped alleles and original alleles during leave-one-out test. Based on pairwise alignment of the region represented by *k-mers* (Unmasked region), the number of mismatched bases are reported and the error rate is the ratio of this value to the total length of the alignment. **e,** Average mismatch rates of genotyped alleles to the ground truth. Leave-one-out genotype: test performed in (d); Non leave-one-out genotype: genotyping to the full pangenome set including the original sample; Closest neighbor: aligning the closest neighbor on the phylogenetic tree to alleles in query samples, indicating the optimal solution of genotyping in current cohort; GRCh38: Aligning the closest GRCh38 alleles to alleles in query samples, comparing it to Leave-one-out test can estimate the percentage of reference variants that can be represented by genotyping. **f,** Detailed leave-one-out comparison in the diploid T2T genome CN1. The results are categorized regarding the number of paralogs in CHM13 to show performances on different levels of genome complexity and the main sources of errors. **g,** Example leave-one-out genotyping results on *CYP2D6* genes (star alleles) including a mutant map based on gMSA for alleles of *CYP2D6* genes from 39 HPRC samples. Black dots show the true positive variants, red dots show false positive variants and blue dots show false negative variants based on sequence comparisons of genotyped alleles.

We applied ctyper to genotype NGS samples within the 1000 Genomes Project (1kgp) including 2,504 unrelated individuals and 641 offspring. The genotype accuracy was measured using Hardy-Weinberg Equilibrium (HWE), trio concordance (Supplementary Table 2), and comparisons to reference assemblies, excluding intronic/decoy PAs (Methods). There are significant HWE violations (p < 0.05) for 0.75% (1896 out of 252,817) of allele-types excluding sex-chromosomes, and setting the maximum copy-number to two (Fig. 3a). There are 27 gene-groups having >15% allele-types with significant disequilibrium, which are mostly small genes (median = 4,564 bp) with few unique k-mers. The average F-1 score for trio concordance is 97.58% (Fig. 3b). There are 18 gene groups having >15% discordance, primarily for genes close to telomeres or on sex chromosomes (Supplementary Table 3).

The paCNVs had an overall high agreement with assembly annotations (slope=1.060) (Fig. 3c), where the discrepancy between genotyping and assembly annotation are largely due to low-quality or truncated genes that are excluded from our database; the high-quality gene-groups that have no filtered sequences are more correlated (slope=0.996).

We then assessed how well the genotyped alleles reflect the original sequence of the sample using 39 HPRC samples with both NGS and assemblies. Each sample was genotyped with its corresponding PAs excluded from the database (leave-one-out), or the full database. Because genotypes are not phased, we used matching to pair the genotyped PAs to the corresponding assembly (Methods), excluding intron/decoys and sequences with <1kb unmasked bases, and used global alignments^46^ between paired PAs to measure how close the genotyped allele is to the query. We performed a similar analysis treating the closest neighbor from the database to each assembly PA as the correct genotyped locus. Across samples, 2.9% of PAs from the left-out assembly and 1.0% PAs using the full database could not be paired, which is likely due to missing-typing, assembly-error or copy number error. Using the full database, paired PAs have 0.36 mismatches per 10kb with 93.0% having no mismatches on unmasked regions. The leave-one-out have 2.7 mismatches per 10kb on unmasked regions, with 57.3% alleles having no mismatches, and 77.0% were mapped to the optimal solution (Fig. 3d). The leave-one-out results were on average 96.5% closer to the original PAs compared to the closest GRCh38 gene at 79.3 mismatches per 10kb, indicating sufficient diversity for accurate representation of new samples (Fig. 3e).

To isolate from errors due to misassembled duplications, we directly compared leave-one-out genotyping results to a telomere-to-telomere phased assembly, filtering out intronic/decoy sequences. The sample genotypes had 11,627 correctly matched allele-types, 599 (4.8%,) mistyped to other allele-types, 131 out-of-reference (1.1%), 127 FP (false-positive) (0.5% F-1), 93 FN (false-negative) (0.4% F-1) for a total F-1 error of 6.7% (Methods) (Fig. 3f).

We examined the *CYP2D* genotypes including *CYP2D6* (star-allele) to assess accuracy at a medically relevant locus associated with drug resistance^47^. All CNVs were correctly called. When measuring SNPs are identified through genotyping with F1-score = 94.0%, outperform Aldy^48^ at 85.2% (Fig. 3g). Regarding the allele-types, 4 out of 74 (5.4%) were out-of-reference, and 4 were mistyped (method).

### Sequence level diversity of CNVs in global populations

**Figure 4.**
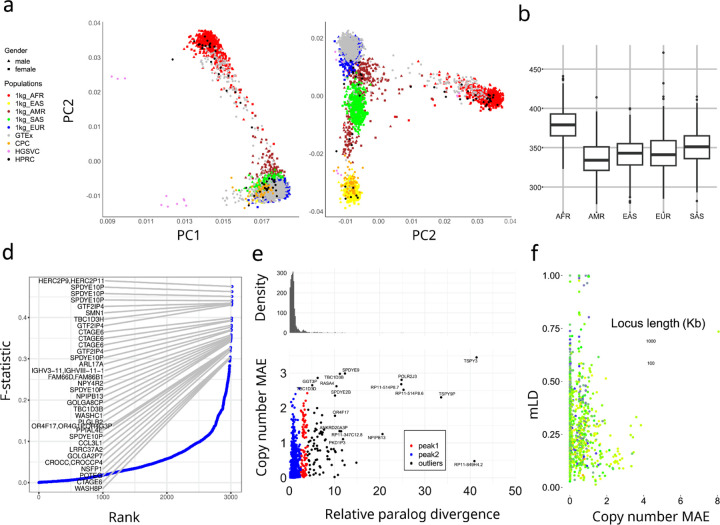
**a,b**. PCA of allele-specific copy numbers on genotype results and assembly annotations. **c,** Distribution of total autosomal gene copy numbers among 2504 unrelated 1kgp samples. **d,** Population differentiation measured by F-statistics of allele-types among different continental populations. The genes with an allele-type with an F-statistic more than 0.3 are labeled. **e,** Copy number and paralog sequence diversities. Based on our genotyping results on 2504 unrelated 1kgp, for genes found to be CNV to the population median in more than 20 samples, we determined the average aggregate copy number difference (MAE) between individuals and estimated the average paralog differences relative to orthologs difference. **f,** Multi-allelic linkage disequilibrium between pairs of CNV genes less than 100kb apart. The largest MAE value of each pair is used for the x-axis values. The total locus length denotes the length from the beginning of the first gene to the end of the last gene.

We used principal component analysis (PCA) to examine the population structure of PA genotypes on 2,504 unrelated 1kgp samples, 879 Genotype-Tissue Expression (GETx) samples, and 114 genome assemblies (Figs. 4a,b). We filtered low frequency (<0.05) allele-types and capped maximum copy numbers at 10. The 1kgp, GETx and genome assembly data were found to cluster by population as opposed to data source, suggesting little bias between genotyping and assembly, as well as across different cohorts. However, the HGSVC assemblies show as outliers on PC1, possibly due to unassembled or filtered alleles.

The top 0.1% highest weighted allele-types on PC1 have an average aggreCNs of 26.33, compared to an overall at 4.00 (p-value=1.11e-16, F-test). Similarly, the PC2 and PC3 have mean aggreCNs variance of 19.73 and 7.20, suggesting CNVs are weakly associated with sequence variants. Furthermore, the PC1 is the only PC that clustered all samples into the same sign with a geographic center away from 0, suggesting it corresponds to modulus variance (hence aggreCNs) if we are treating samples as vectors of paCNVs. Meanwhile, the PC2 and PC3 are similar to the PCA plots based on SNP data on global samples^49^, suggesting they are associated with the sequence diversity among those CNV genes. The total number of duplication events are elevated in AFR populations (Fig. 4c), reflected in the order of PC1 (Fig 4a).

We next used the F-statistic that is similar to the F_st_ but accommodates more than two genotypes (Methods) to test the differences in copy number distributions across five continental populations (Fig. 4d). In total, 223 out of 5065 (4.4%) of duplicative allele-types showed population specificity (F-statistic > 0.2). The allele-type with the highest F-statistic (0.48) contains duplicates of the *HERC2P9* gene that is known to have population differentiation^11,50^. Another example of a highly differentiated allele-type is a converted copy of *SMN2* annotated as a duplication of *SMN1* that is enriched in AFR populations (F-statistic=0.43).

We then measured whether duplicated genes were similar or diverged from reference copies, which would indicate recent or ancient duplications, as well as provide a measure on reference bias due to missing paralogs. We used multiple sequence alignment (Methods) for each gene group to determine the pairwise differences on their unrepetitive homologous sequences. We determined the average paralog divergence relative to ortholog divergence (Methods), which we refer to as relative paralog divergence (RPD). We also determined the mean absolute error (MAE) of the gene copy number in the populations (Fig. 4e). Based on RPD, using Density-Based Spatial Clustering of Applications with Noise^51^, we identified two peaks at 0.71 and 3.2, with MAE centers at 0.18 and 0.93. The first peak indicates genes with rare and contemporary CNVs, while the second peak indicates more divergent and common CNVs, often CNVs that may be inherited as different structural haplotypes. For example, *AMY1A* has a high RPD at 3.10. This is because of the truncated duplications of *AMY1A* (blue gene annotations in Fig. 2a). These results are consistent with ancient bursts of duplications in humans and primate ancestors^52^.

We next investigated if gene families could be represented by a limited number of distinct structural haplotypes, or if paralogs are decoupled by recombination. We determined multi-allelic linkage disequilibrium (mLDs) between PAs using the 1kg genotypes^53^. We computed mLDs for 989 PAs that were adjacent and less than 100kb apart on GRCH38 (Fig. 4f), and found the average within each gene-group. Among all mLDs, there was a strong negative rank correlation between MAEs of the copy number and mLD (*ϱ*=−0.24, p-value=3.4e-15, Spearman’s rank), which is stronger than the rank correlation between MAEs of gene copy number and total locus length (*ϱ*=−0.21, p-value = 1.5e-11), suggesting a reduced haplotype linkage on genes with frequent CNVs. The lowest mLD=0.013 found on *FAM90*, a gene on chromosome 8 with frequent duplications and rearrangements^54^. Not surprisingly, for highest locus with mLDs > 0.7, 19 out 29 are sex chromosome genes, and two *HLAs* (*HLA-B and HLA-DRB*, the locus found no real recent duplication but deletions after sequencing bias correction, Supplementary Methods). The *amylase* locus has a value of 0.293 due to a high degree of recombination (Fig. 1a).

### Quantitative trait locus (QTLs) on pangenome alleles

To investigate the functional impact of paCNVs, we performed QTL analysis based on two RNA-seq cohorts: Geuvadis^55^ and the GTEx project^56^. The analysis of eQTLs for repetitive genes is challenging due to degenerate alignments in repetitive sequences and divergent structural haplotypes^57^. To resolve this, we pooled expression from genes that cannot be distinguished by transcript alignments^58^ on GRCh38 (Methods), denoting the distinguishable individual genes or pooled genes as QTL units. In total the expression of 4,512 genes could be uniquely identified by RNA-seq alignments, and 44 genes had pooled expression values such as *SMN1/2* (Supplementary Table 4).

**Figure 5.**
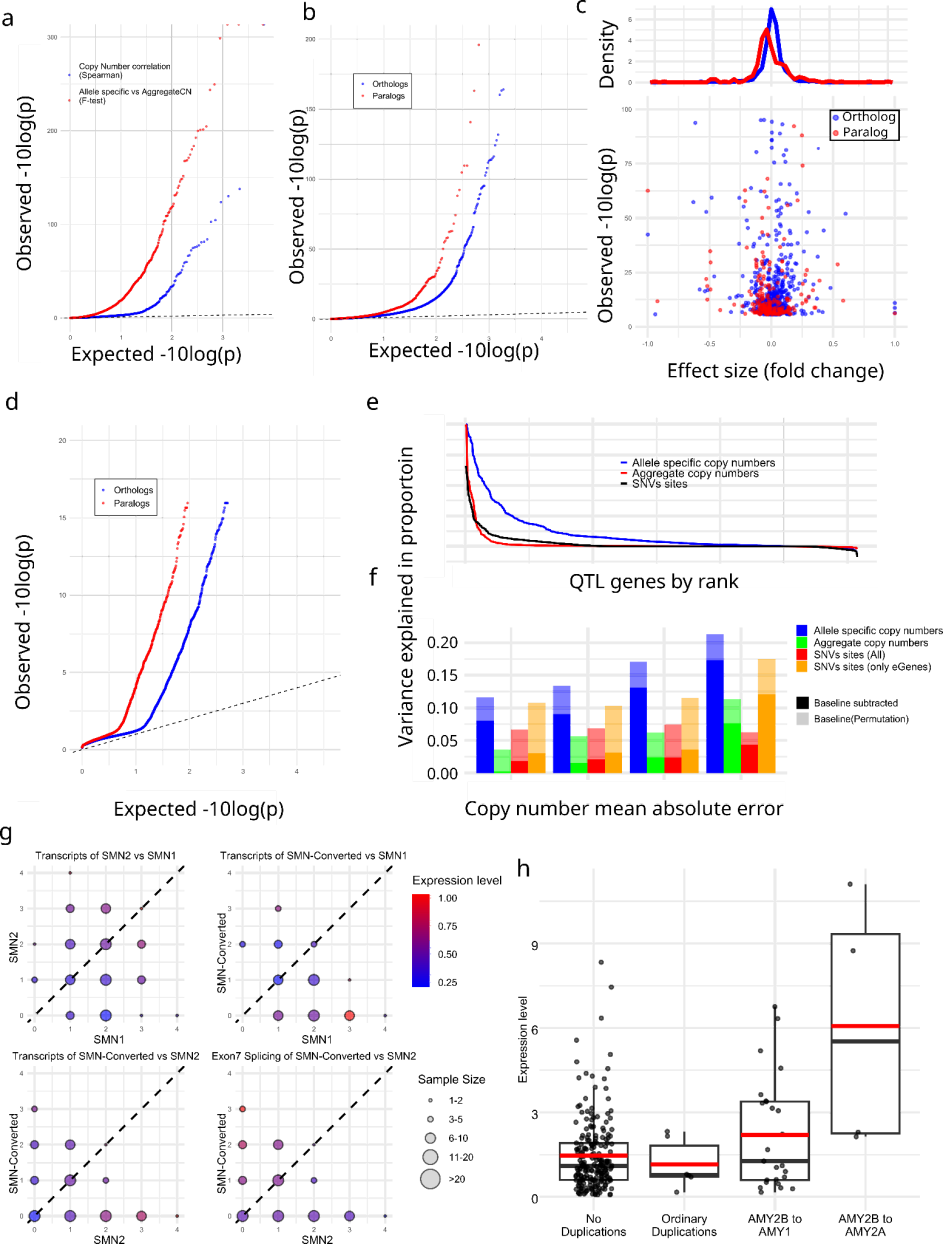
**a.** Q-Q plot of association of aggregate (*blue*) and allele-specific (*red*) copy numbers to gene expression in Geuvadis samples. **b,** Comparative gene expression of orthologs (*blue*) and paralogs (*red*). **c,** Fold change effect size of all alternative expressions. For all allele-types found to be significant, the fold change as well as p-values shown. **d,** Preferential tissue expression of orthologs and paralogs. **e,** Model evaluation for PAs representing gene expression diversities. **f,** Quantification of variance explained by different representations of genomic diversity: full paCNV genotypes, aggregate copy number, and SNVs. **g,** Case study on *SMN* genes showing decreased gene expression on converted *SMN*. The average relative expression level in Geuvadis samples is shown under different copy numbers of *SMN1*, *SMN2*, and converted *SMN*. Transcript levels are the total coverage of all isoforms, and exon 7 splicing level is measured by counting isoforms with a valid exon 7 splicing junction. **h,** Case study on amylase genes showing increased gene expression on translocated *AMY2B*. Using GTEx samples from the pancreas, expression levels are compared among samples with different types of *AMY2B* duplications. Samples with multiple types of duplications are excluded.

We corrected experimental bias on expression using PEER^59^ with the first three PCs from genotypes^60^, and performed association analyses using paCNV genotypes. Using aggreCN, we found 178 out of 3,224 QTL genes (5.5%) showed significance (corrected-p = 1.6e-0.5, Pearson-correlation) as previously observed^39^. We then tested whether using paCNVs would provide stronger fitting by updating the aggreCNs with individual paCNVs and performing multivariable linear regression on expression (Methods). We found significant improvements on 890 QTL units (27.6%) (corrected-p=1.6e-05, one-tailed F-test) (Fig. 5a).

The improved fit could be explained by non-uniform effects on expression of PAs in the same gene-unit. To test this, we used a linear mixed model^61,62^ (Methods) to regress total expression to individual PAs to estimate individual expression levels, and compared these values to their peers (Supplementary Table 5). We found that 150 out of 1,890 paralogs (7.94%) and 546 out of 16,628 orthologs (3.28%) had significantly different expression levels (corrected with sample size = number of paralogs + orthologs, corrected-p = 2.7e-06, Chi-square test) (Fig. 5b).

We further investigated if these deviations were tissue-specific. Using GTEx, we compared across 57 tissues to see if PAs had different most expressed tissues than their peers. Similarly, we applied linear mixed model analysis to estimate the expression levels on each tissue (Supplementary Table 6). We found tissue specificity for 132 of 2,820 paralogs (4.7%) and 225 of 19,197 orthologs (1.2%) (corrected-p = 6.4e-08, union of two Chi-square tests) (Methods) (Fig. 5c).

Additionally, we used analysis of variance (ANOVA) to estimate the proportion of expression variance explained by paCNV genotypes using Geuvadis, and compared it to a model based on known conventional QTL sites^63^ based on SNVs. As expected, the highly granular paCNV genotypes explain the most variance: on average, 10.3% (14.3% including baseline). In contrast, 58.0% of QTL genes are eGenes with known QTLs sites that in total explained valid variance by 2.14% (1.60% considering experimental noise, in agreement with prior study with the same data at 1.97%^64^). On average, 1.98% of the variance was explained by aggreCNs (Methods), and 8.58% was explained by allele-type information. When combining both paCNVs and SNVs QTL sites, 10.40% (19.0% including baseline ) of the valid variance was explained (Fig. 5d).

We examined *SMN* and *AMY2B* genes as case studies due to their importance in disease and evolution^38,65^. We classified *SMN* genes into three categories: *SMN1*, *SMN2*, and the previously mentioned *SMN-converted*. We estimated both the total expressions of all transcripts and the expressions of only isoforms with a valid exon 7 splicing junction. For total expression, no significant difference was found between *SMN1* and *SMN2* (0.281 ± 0.008 vs 0.309 ± 0.009, p-value = 0.078, Chi-square test). However, significant differences were found between *SMN-converted* and *SMN1/2* (0.226 ± 0.012 vs 0.294 ± 0.002, p-value = 1.75e-07, Chi-square test), with a 23.0% reduction in expression of *SMN-converted*. In contrast, *SMN-converted* had 5.93✕ the expression of *SMN2* (p-value = 2.2e-16, Chi-square test) regarding valid exon 7 splicing, indicating that while *SMN-converted* has full functional splicing, its overall expression level is lower (Fig. 5e).

For *AMY2B*, we studied the expressions of duplications when they are translocated to proximal to other *AMY* genes, such as the PAs containing *AMY1* and *AMY2B* at figure 2a. Using GTEx pancreas data, we estimated their expressions as well as other duplications. We found that these translocated *AMY2B* genes had significantly higher expression than other duplications (1.384 ± 0.233 vs −0.275 ± 0.183, p-value = 7.87e-09, Chi-square test) (Fig. 5f).

## Discussion

New pangenomes present both opportunities and challenges for the study of complex genetic variation: while the landscape of complex variation is becoming more clear, it is challenging to use these sequences to analyze biobank (NGS) cohorts. To enable this analysis, we developed an approach to divide assemblies into pangenome-alleles: sequences that are copy number variable and inherited in complex forms, and to genotype their copy number in NGS samples.

The use of ctyper genotypes increases the scope of studies on copy-number variation to include the context of sequence variation between copies. For example, our finding that CNVs reflect two modes of variation: high-identity (and likely recent), and low-identity (ancient and polymorphic) duplications, is based on ctyper genotypes rather derived strictly from assembly annotations. As another example the ctyper genotypes yield tissue specific expression of paralogs as well as relative contributions to expression of different forms of duplications such as *SMN*.

We investigated the reasons behind the disparity between the ANOVA on PAs versus SNVs/indels. First, in contrast to PAs, biallelic SNV found very few or very many QTLs sites per gene, indicating LD of QTL (Supplementary Figure 3) as addressed by fine-mapping^66^, increasing multiple testing burden^67^. Additionally, there are greater proportions of variance explained among genes with more CNVs, which is contrary to the fact that variant calling is more challenging among CNV genes^68^. This could also be explained by indirect associations (an example in Supplementary Figure 1 on *HPR* genes). Furthermore, as CNVs increase, the explained variance by known QTL sites increases (t= 3.80, p-value = 1.6e-04), while the number of known QTL sites is inversely correlated (t = −4.79, p-value = 2.1e-06), suggesting that larger effects like CNVs might obscure the discovery of other not linked variants. This is expected because binary SNV association relies on comparing the expression between groups with/without the variant relative to variations within groups, but the difference in other variants will increase variation within groups and weaken the confidence^69^. However, this type of noise can be potentially solved using the non-binary representation of PAs to decouple variants. Another potential reason is that gene expression might not be a simple linear additive effect of all the variants^70^. For example, although *SMN*-converted contains variants that can be found in either *SMN1* or *SMN2*, its overall expression is lower than both. By applying pangenome representation into association analysis, PA might help in those issues, highlighting a wider potential to be applied to even non-CNV genes in the future.

Due to limited sample size, our associations are at the level of allele-types rather than individual PAs. Different cohort sizes may require different levels of granularity when defining allele-types. For example, the three allele-types of *SMN-converted* showed little difference in expression. Our current classification on allele-types was designed to work on large cohorts like Biobank data, so smaller cohorts may need to test on allele-types that aggregate more PAs. The granularity of genotyping is additionally defined by the length of PA sequences; genotypes using shorter PAs will more accurately reflect NGS samples, while longer sequences can preserve phase and may be preferable in regions with low recombination such as *HLA-DRB*.

Ctyper also has limitations. First, while it is possible to detect CNVs smaller than PA units using ctyper (Supplementary Methods), it is not currently supported due to lack of benchmarking data. Second, while each PA reflects a locally phased gene, the genotypes are not phased at larger scale. Third, at this early version, ctyper does not provide confidence value for the results. Finally, the high-dimensionality of PAs increases the complexity of interpretation and the need for large sample sizes to support associations on multiallelic variants.

The average runtime for genotyping at 30x coverage was 80.2 minutes on a single core (Supplementary Figure 4), indicating that ctyper is suitable for biobank scale analysis. As new high-quality references become available, we anticipate ctyper to be a useful method for interpreting the association between CNV and traits at scale.

## Online Methods

### Constructing pangenome allele database

We initiated our study by identifying gene duplicates within pangenome assemblies. Our pangenome cohort was composed of assemblies from the Human Pangenome Reference Consortium (HPRC) (N=92, excluding HG02080 due abundant flagged regions), the Chinese-Pangenome Consortium (CPC) (N=114), and the Human Genome Structural Variation Consortium (HGSVC) (N=18, only Pacbio HiFI assemblies were used), two telomere to telomere diploid assemblies (N=4), and reference genomes (GRCh38 including alternative loci and CHM13 T2Tv1). The gene database used for annotation was GENCODE v39 based on the GRCh38 reference genome.

The initial application of this study was on 3,203 genes known to have copy number variation detected by the HPRC and CPC studies.

We organized genes into gene ‘query sets’ where each query set encompassed genes with functional or similar sequence including pseudogenes and genes with distant homologies within the same gene family. The query sets were initially defined based on genes with shared name prefixes, and were used to locate copies of duplicated genes within the pangenome.

To detect copies of genes in pangenome, we created an alignment scheme to search for copies of genes in all pangenome assemblies (including references). For each query set, we mapped *k-mers* (*k*=31) from all initial reference genes that appeared fewer than 255 times in the CHM13 genome to each of the pangenome assemblies and references. We then located *k*-mer hotspots, defined by maximal intervals of mapped k-mers containing more than 200 k-mers within any 1000-base window within the interval. To aid in mapping small and fragmented pseudogenes, we included an additional criteria to define hotpots using 50 exonic *k-*mers on the same interval search. Subsequently, we used BLASTn^71^ to refine the boundaries of each hotspot by aligning all reference genes in this query set to each *k*-mer hotspot extended by 10kb flanking sequences.

The *k*-mer defined hotspots include both individual loci mapped by multiple genes from a query set as well as loci with tandemly duplicated genes multi-mapped by individual genes in a query set. To account for this redundancy, we merged alignments that were less than 10,000 bases apart, causing tandem duplicated genes to be merged into single loci. To avoid genotyped loci may be split by recombination, if an intron exceeded 20,000 bases, we divided the locus at the midpoint of the introns. To ensure the overall sequence size was comparable, flanking sequences both upstream and downstream were adjusted to achieve a total length of 15,000 bases. These methods aimed to standardize the size of each sequence to be roughly 30,000 bases, approximating the size of linkage disequilibrium (LD) blocks. The collection of all sequences mapped by a query set are referred to as initial gene-groups.

### Definition of gene-groups and *k-mer* selection

Because the initial gene-groups were defined from aligned query sets that potentially arbitrarily grouped genes with unrelated sequences based on name, we used subsequent steps of refinement to exclude unrelated sequences.

Initially, for each genome we extracted all *k*-mers exclusive to aligned locations of the initial gene-groups (hence not found elsewhere in the genome). We also filtered out *k*-mers identified as repetitive DNA such as Variable Number Tandem Repeats (VNTRs), microsatellites, and transposable elements, as well as those demonstrating high (>70% or < 30%) GC content bias.

Subsequently, we filtered sequences predominantly composed of the *k*-mers removed in the previous step. The remaining sequences were then categorized into subgroups based on the number of shared *k*-mers. This classification was achieved using graph partitioning. Each sequence was represented as a node, and edges were made between node pairs sharing an excess of 500 unique *k*-mers, except for *NBPF* and *ANKRD* genes, for which a higher threshold of 2,000 unique *k*-mers was set to manage group sizes more effectively for computational efficiency. Each partition represents a singular gene-group, and a unique list of *k*-mers specific to that group was compiled and termed its ‘*k*-mer list’.

As an additional filtration, we filtered out genes from the non-confidence regions reported by the HPRC, as well as truncated genes from small scaffolds. The genes included needed to be at least 10,000 base pairs away from both ends of a scaffold, except for sequences from genes taken from the reference genomes located at the telomeres.

### *k*-mers based phylogenetic tree construction

We constructed phylogenetic trees for each gene group based on their *k*-mer composition. Initially, for every gene group, we assembled a *k*-mer matrix, M, that encapsulates pangenome-alleles in the gene group. Within this matrix, individual rows represent distinct gene sequences, while each column corresponds to a unique *k*-mer from the *k*-mer list exclusive to the gene group. The matrix cell values are the counts of each *k*-mer present in the respective gene sequence, which is mostly 0 or 1, but occasionally more than 1 when there are low-copy repeated sequences in the gene, or the row represents a tandemly duplicated locus.

The matrix M allows us to measure the concordance between any two sequences, ${\text{G}}_{\text{i}}$ and ${\text{G}}_{\text{j}}$, by calculating their inner product, denoted as $<{\text{G}}_{\text{i}}\;*\;{\text{G}}_{\text{j}}>$. Consequently, the norm matrix, $N\;=\;\text{M}\;*\;{\text{M}}^{\text{T}}$, reflects the k-mer concordances for all sequence pairs within the gene group.

We constructed a similarity matrix, S, where $S_{\text{i},j}$ is the cosine similarity of ${\text{G}}_{\text{i}}$ and ${\text{G}}_{\text{j}}$ representing the sequences. The cosine similarity for any two sequences, ${\text{G}}_{\text{i}}$ and ${\text{G}}_{\text{j}}$ can be obtained by normalizing the norm matrix N according to the squares *k-mer* vectors (approximately equal to sequence lengths) of the sequences in question.

Finally, we used the Unweighted Pair Group Method with Arithmetic Mean (UPGMA) algorithm on the similarity matrix S to generate the phylogenetic tree for each gene group.

### Clustering of pangenome alleles into alle-types

We used phylogenetic trees for the annotation and classification of closely related groups of alleles, which we term ‘pangenome allele-types’. The classification of pangenome allele-types is guided by two primary criteria applied across all allele-types:

Homogeneity within allele-types: A allele-type must exhibit near-identical characteristics amongst its members, which is quantified by ensuring the largest k-mer distance between any two members does not exceed 155 *k*-mers, which is roughly equivalent to the variation caused by 5 single nucleotide polymorphisms or a structural variation of approximately 95 base pairs, such that allele-types are capable of representing most common variants in about 30K range.

Distinctiveness of allele-types: Each allele-type must be distinct from its neighboring allele-types. This is measured using a *k*-mer F-statistic score, which must exceed 2 when compared with adjacent allele-types. In cases where allele-types are composed of fewer than three members, the F-statistic may not be reliable; hence, we default this score to 0 for such small allele-types, but change the cutoff of the former criteria to 155 * 3 to detect singleton rare events.

Employing a ‘bottom-up’ recursive approach starting from leaves, we applied these criteria to all allele-types, aiming to identify and report the largest possible near-identical allele-types. These are later used to identify equivalent loci after genotyping.

### Pangenome allele annotation relative to the reference genome

We annotate CNVs events and duplicated alleles in the pangenome assemblies in relative the GRCH38 genome. This requires us to find out the corresponding GRCH38 gene for each pangenome allele. However, this is a known challenging problem of orthology assignment^72^.

First, PAs often align to multiple paralogs on GRCH38, and the gene overlap with their liftover locations may not be the most similar reference gene due to gene conversions and translocation (Figs. 1f and 2a). To address this problem, we designed a method to match PAs to their closest GRCh38 genes based on their *k*-mer similarity. We obtained all pairwise similarities between each pair of PAs across both haplotype genomes. Starting from the most similar pair, we iterate this until all PAs are matched or failed to match (has no reference gene with >90% similarity). Secondary redundant matches (match to reference genes that are already matched) are annotated as duplications (interspersed).

Second, the former failed to match PAs are likely alleles with large SVs, including local proximal duplications. We attempted to lift them back to GRCh38 using their flanking sequences (100K either side). Because it is challenging to directly liftover genes in the regions with large segmental duplications, we designed this liftover to be a two-stepped liftover. First, we used lift PAs to the region with the best local alignment coverage, allowing SVs to break alignments. Second, we performed a global pairwise alignment between PAs and the lifted region to locate the best aligned gene with the presence of translocations and tandem duplications (Supplementary Methods).

Third, to annotate the proximal duplications mentioned in the last step as well as to annotate diverged paralogs that failed to match from both liftover methods, we annotate PAs regarding the gene transcripts. We aligned all exons from the same gene group to PAs, and based on the exon orders and alignment scores, we determined the optimal combinations of transcripts on each PA (Supplementary Methods). The PAs containing no exons are annotated as introns and PAs containing only transcripts of other non-interested genes are annotated as decoys. Introns and decoys are usually filtered out from analysis and the rest PAs are considered as valid alleles, including pseudogenes that have no intact protein-coding transcripts and putative protein-coding genes with intact protein-coding transcripts.

It is important to note that, because proximal duplication may be highly associated in inheritance and potentially interference with each other functionally such as co-expression (which found between *HP* vs *HPR*, Supplementary Figure 1), and exonic expansion can be found in gene *LPA* and *NBPF,* we treated PAs with proximal duplications as a new type of whole unit, instead of treating them as multiple independent copies of singletons.

### Definition of orthologs and paralogs in the pangenome

Based annotation results, to illustrate the relation of PAs to their corresponding reference genes regarding the copy number variations, we classified PAs into four categories, including two types of orthologs and two types of paralogs:

1. Reference alleles include alleles from GRCh38, representing the alleles almost identical to the reference sequences.
2. Alternative alleles are orthologs located at the same genomic loci as the reference gene but are distinctly different, including alleles that have large structural variations on their unique sequences, or alleles that have a list of small variants in strong linkage. These alleles may possess proximal gene/exon duplications or deletions, as observed in genes like *HPR*, *NBPF*, and the *CYP2D6* (star-alleles) gene.
3. Duplicative paralogs (alleles) consisting of paralogs that have been translocality duplicated to different loci from the reference alleles. Despite being translocated, they retain high similarities to the reference alleles. These alleles often reflect large, recent segmental duplications in the genome, including similar paralogs, such as AMY1A, AMY1B, and AMY1C, which are still often considered as the same gene despite their distinct locations.
4. Diverged paralogs (alleles) not only differ in their translocation status but also have sequences that are significantly divergent from reference alleles, such that cannot be simply assigned to a single reference gene. These are typically characterized by highly diverse non-reference paralogs, incomplete gene duplications, and processed pseudogenes. An illustrative example of the novel allele-type is found among amylase genes, which indicates a proximal translocation event between *AMY1* and *AMY2B* genes.

### Genotyping NGS sample with ctyper

The goal of ctyper is to select a list of pangenome alleles and determine individual copy numbers to represent the CNVs of unknown NGS samples. Instead of sequence alignment, our genotyping is based on *k*-mer comparison, which is not only more efficient but also not affected by misalignments which frequently happens in the genomic region enriched in structural variations and repetitive elements. Another advantage is that there is little bias in k-mers between high quality long-reads and NGS data^73^, so the *k*-mer data based on assemblies can be applied on predicting NGS data.

The genotyping proceeds per-gene. Given an NGS sample and a *k*-mer matrix M derived from pangenome allele annotation, we find a vector V for an NGS dataset that includes the counts of each k-mer found in the matrix for the NGS sample, which is normalized by the sequencing coverage. We seek to find a vector X of copy-number of each pangenome allelee that minimizes the squared distance to the *k*-mer counts we observed in NGS data, e.g. ${\mathrm{argmin}}_{x}\left(\left\|\text{M}\;*\;\text{X}-\text{V}\right\|\right)$. Compared with absolute distance, squared distance is more suitable for NGS data’s normal-like noise^74,75^. Although directly obtaining integer solution (mixed-integer linear program, MILP) has been shown is NP-hard and can only be used with very few selective variants/k-mers^48,76^, which restricts its application on the pangenome, the relaxed non-integer solution has an analytic solution, which can be efficiently solved. In essence, the computational problem is akin to a multivariable linear regression. The non-negative least error (NNLS) solution can be further obtained via Lawson-Hanson algorithm^77^.

The last-step is referred to as reversed phylogenetic regression. Our mathematical analysis (Supplementary Methods) reveals two strong relationships between NNLS and the integer solutions under a phylogenetic relationship. First, the coefficient on each known allele G is inversely proportional to its cosine vector distance to the unknown NGS allele. Hence, in a pangenome with a good representation, the coefficients of NNLS will be mostly located on the genes that are very similar to the unknown gene. Second, when the coefficients are located on genes that are very similar to the unknown gene, the sum of total coefficients will be very close to the sum of its integer copy number.

Based on the solution’s high convergence on the phylogenetic tree, we designed a greedy algorithm to efficiently collect non-integer solutions and round it to integer solutions. This algorithm is iterative, employing a bottom-up approach from leaves to root. At each level of the hierarchy, we round the non-integer values to the integer solution with the least overall residual, and propagate the remainder to the next hierarchy. Because at each hierarchy, there are only two remainders from either branch of the tree, this solution is highly efficient.

### Trio analysis

Trio analysis is to determine if the genotype combinations of child-father-mother show possible Mendelian violations. When the copy number of child = 0, the parents need to be 0 or 1; When the copy number of child =1, the parents can not both be 0 or both be 2; When the copy number of child =2, the parents both need to be 1 or 2. When the copy number of a child is more than 2, the parents need to have the sum to be greater or equal to this number.

### Leave-one-out comparison of genotyping results to pangenome assemblies

To find out the extent to which the genotyping results can represent the individual small variants on each PAs, we aligned PAs in the original assemblies to their corresponding PAs in the genotyping results.

First, the original assembly PAs were one-to-one paired to genotyped PAs. This pairing was finished by a greedy method. We obtained all pairwise similarities in *k*-mer between each pair of the PAs across original assemblies and genotyping results. Starting from the most similar pair, we paired those alleles without replacement and iterated this until all original assemblies PAs are either paired or failed to be paired (has no genotyped PAs with >90% similarity).

Second, the paired PAs were then aligned using global pairwise alignment tool Stretcher^78^. From the global alignments, we obtained the number of mismatched bases in the unmasked region, where the low copy repeat *k*-mers are used in *k*-mer matrices.

### Classification of errors

We classified four types of errors for our benchmarking:

1. False positive: the genotyping results have an additional copy;
2. False negative: the genotyping results have a missing copy;
3. Miss typing: assign a copy to incorrect type;
4. Out of reference: the singleton type among the pangenome and lost reference during leave-one-out.

### Benchmarking *CYP2D* genes and visualization

We plotted the benchmarking results from all 39 HPRC samples with NGS data from the leave-one-out analysis. First, we plotted the graphic MSA for all 39 HPRC samples. Second, we obtained the graphic MSA results of their corresponding genotype results and mapped to the same location. Third, for overlapped variants (TP) we colored with black, for variants missed in original PAs (FP) we colored with red, and for variants missed in genotyping results (FN), we colored with blue.

In comparison, using the same comparing scheme, we also benchmarked a *CYP2D6* genotyping tool Aldy with the default setting. We took the phased results of Aldy and matched them to their corresponding original PAs, in a range about 6k, where the variants could be found, Aldy genotyped the variants at F-1 score = 85.2%. However, it is important to note our benchmarking strictly requires all variants being both correctly called and phased, so the “incorrected” variants might still be correctly called but incorrectly phased.

### Total number of duplication events from genotyping results

Based on ctyper’s genotyping results, we calculated the total number of duplication events for each 1kgp sample, excluding 7 due to having extreme values different from the population mean by more than five standard deviations. The total number of each reference gene is measured in each genome and compared to GRCh38 chromosomes excluding alternate haplotypes. Each duplication event is called if the genome has more copy number than GRCh38. The total number of duplication events is reported for each genome.

### Obtain F-statistic values

Because allele-types may have copy numbers beyond of two and may not be applicable to Fixation index (F_st_), we instead used F-statistic value to measure the population specificity of allele-types. The F-statistic value is based on the F-test, where we obtained the variances of copy numbers within all individual populations (within-group-variance), and use it to divide the variances of copy numbers across different populations (between-group-variance).

### Relative paralog divergence (RPD)

Relative paralog divergence measures the mean divergences of the paralogs to other alleles, in relative to the mean divergence between only orthologs. RPD is determined for each reference gene and based on the graphic multiple sequence alignments (gMSAs, Supplementary Methods) of PAs assigned to that reference gene as well as our genotyping results.

First, the divergence value is determined for each pair of PAs assigned to the same reference gene. It is measured based on the alignment scores (misalignment and gap open = −4, and gap extend = 0, normalized by total alignment length) from gMSAs.

Second, we obtained mean divergence of the orthologs by averaging divergence values between the two PAs from samples with CN = 2.

Third, we then determined the population median copy numbers for each reference gene, and divided samples into those with additional copy numbers (copy numbers more than the median) and those with no additional copy numbers (copy numbers nt more than the median).

It is unreliable to directly distinguish the paralog from orthologs due to complex rearrangements (e.g. Figure 2a). To overcome this limitation and only obtain the divergence values from additional copies, we performed statistical estimations based on large populations. We first estimated the mean divergence values from samples with no duplications and used it as the unit baseline B. When the population median CN = Y, because there are $Y\left(\text{Y}-1\right)/2$ pairs, then the total baseline is $B\;*\;\text{Y}\left(\text{Y}-1\right)/2$, which will be subtracted from total divergence values of samples with duplications.

After subtracting the total baseline, the mean paralog divergence value of the additional copies are determined for all samples with duplications. This mean paralog divergence is then normalized by mean divergence of the orthologs obtained in step two.

### Multi-allelic linkage disequilibrium

Multi-allelic linkage disequilibrium (mLDs) is an analytic continuation of SNP-based biallelic linkage disequilibrium to allow computing linkages between multiple genotypes of neighboring loci. When there are only two genotypes on both loci, mLDs equals LD value. When there are more than two genotypes, mLDs measures LDs between each pair of allele-types across different loci, and takes the weighted average of all pairs. This weight is the product of both allele frequencies of the pair.

### Defining QTL units

We represent each gene by the major transcripts from the MANE (Matched Annotation from NCBI and EMBL-EBI^79^) project. Second, individual exons are aligned. Transcripts are recursively clustered together if they overlap with previously clustered transcripts with more than 98% overall similarity taking the average similarity of all aligned exons from the transcripts. We refer to these clusters as QTL units. Third, for each QTL unit, we identify all its exons and look for unique exons that do not overlap with exons from other QTL units. Fourth, we use these unique exons to represent each QTL unit and filter out QTL units that have no unique exons (2079 out of 2579 filtered genes are known pseudogenes). Lastly, we assign PAs to each QTL unit if they contain any of the corresponding unique exons with at least 98% similarity.

### Expression correction

For individual tissue analysis, similar to the prior study^60^, we logistically corrected the raw TPMs using tool PEER together with the first three principal components obtained from reported genotypes in chr1^80^. For cross-tissue analysis we corrected raw TPMs using DESeq2^81^.

### Association between CNVs to gene expression

We first associate gene aggregate copy number to expression levels using Pearson correlation (linear-fitting). The p-values and residuals of this fit are recorded. To test if including allele-specific information would improve the correlation, we used the ctyper pangnome allele-specific copy numbers to replace the aggregate copy numbers to perform multi-variable linear regression using allele-specific copy numbers as dependent variables and gene expression level as independent variables. We compared the residuals of multi-variable linear regression with residuals from Pearson correlation using F-test, and one-tailed p-values of the reduced residual is reported.

### Linear mixed model

We performed the linear-mixed-model to measure the individual expression of each allele-type. We used the total gene expression values as the vector of observed dependent variables, different allele-types as the vector of independent fixed variables and the copy numbers from ctyper genotyping results are used as their coefficient matrix. The effect sizes of fixed variables are then solved using ordinary least squares (OLS) regression.

### Determine alternative expression of allele-types

In order to determine if an allele-type has an alternative expression level in comparison with other allele-types of the same genes. We merged all other allele-types of the same genes as a single variable separate from the currently tested allele-type, as well as other PAs such as other paralogs that might also affect the estimation of total expression. Using a linear mixed model and R’s linear fitting function^62^, we regressed the expression values to all variables to get their effect sizes. We then compared the effect-size of currently tested allele-type and the effect-size of other allele-types of the same genes using chi-squared distribution with the linearHypothesis tool^82^.

### Across tissue expression comparison

In order to determine if an allele-type has alternative expression tissue compared to other allele-types of the same gene, we merged other allele-types of the same genes as a single variable separate from the currently tested allele-type, as well as other PAs that might also affect the expression. We performed linear mixed models to estimate the gene expression level of each allele-type within each of the 57 tissues in GTEx V8. The tissue with the highest expression level was recorded and compared to the tissue with the second highest expression using the chi-squared test. We then compared the results between currently tested allele-type and all other allele-types of the same gene to see if they have the different highest expressed tissue. When the highest expressed tissues were different, we tested the p-value of either events happening by combining the p-values from both side as p-combined = p1 + p2 - p1 * p2. This p-value is then corrected by both the number of alleles tested and the number of total tissues.

### Anova (Analysis Of Variance) test on gene expression

We first measured the total expression variance for each QTL gene unit, filtering out units with per-sample variance less than 0.1 to exclude genes not sufficiently expressed in the Geuvadis cohort. We estimated experimental noise by measuring expression variance between different trials of the same individuals (mean = 10.5% of the total variance) and excluded units where experimental noise exceeded 70% of the total variance, resulting in 639 total QTL units on expression. In case of over-fitting, we applied the one-in-ten rule to restrict the number of variants tested to be not greater than 45 (10% of the sample-size). We filtered out 18 units involving more than 45 PAs; When there were more than 45 eQTL sites, we used 45 variants with the lowest p-values. The valid expression variance was obtained by subtracting experimental noise from total expression variance. Using ANOVA, we estimated the explained valid variance and adjusted the results by subtracting a baseline, defined as the mean expression variance explained by permuting the orders of all samples (estimated by the mean of 100 trials). If there are no reported QTL sites, a value of 0 is used.

For paCNV, we further investigated the part of variance explained by gene aggreCNs, applying ANOVA to a random matrix with aggreCN information, such that has randomly assigned allele-types, but with the total copy number equal to the original matrix. We subtracted the variance explained by this random matrix from the total explained variance to obtain the variance explained by allele-type information.

## Supplementary Material

Supplement 1
