# Genotyping sequence-resolved copy-number variation using pangenomes reveals paralog-specific global diversity and expression divergence of duplicated genes

**DOI:** 10.1101/2024.08.11.607269

**Published:** 2024-08-29

**Authors:** Walfred Ma, Mark JP Chaisson

**Affiliations:** 1. Quantitative and Computational Biology, University of Southern California, CA, USA.; 2. The Genomic and Epigenomic Regulation Program, USC Norris Cancer Center, University of Southern California, Los Angeles, California 90033, USA

## Abstract

Human pangenomes contain assemblies of non-reference copy-number variable (CNV) genes. We developed a new method, ctyper, to identify the copy-number of specific alleles of CNV genes cataloged in pangenomes with NGS datasets. Applying ctyper to the 1000-genomes samples revealed population stratification of paralogs and two classes of CNVs: recent CNVs due to ongoing duplications, and polymorphic CNVs from non-reference ancient paralogs. Expression quantitative trait locus analysis determined allele-specific expression within gene families, revealing that 7.94% of paralogs and 3.28% orthologs had significantly divergent expression. Case studies of individual genes include finding lower expression on *SMN*-1 copies that arose from conversion from *SMN-*2, and increased expression on a form of *AMY2B* that has undergone a translocation. Moreover, 4.7% of paralogs and 1.2% of orthologs had different most-expressed tissues. Furthermore, the genotypes explain more expression variance than known eQTL variants. Overall, ctyper enables biobank-scale genotyping of sequence-resolved CNVs.

## Introduction

Human genomes are characterized by frequent duplications and deletions, leading to copy number variation (CNV). Up to 10% of protein-coding genes are known to be copy-number variable, showing distinct distributions across human populations^1,2^ and association with traits such as body mass index^3^ and disease including cancer^4^, cardiovascular diseases^5^, and neurodevelopmental disorders^6,7^. While CNVs are infrequent genome-wide, regions of long, low-copy repeats called segmental duplications (SDs) are enriched in genes and are catalysts for frequent CNVs^8,9^. This leads to highly diverse gene families such as *TBC1D3*, *NPIP*, and *NBPF*^10,11^. The mechanisms contributing to CNVs, along with the elevated mutations in SD regions^12^, result in variation not only in aggregate copy number (aggreCN) but also a high degree of sequence variation among the copies themselves^12–14^. This variation can influence phenotypes and disease susceptibility^15–17^, such as hypertension, and type 2 diabetes^18^.

Despite years of study, there is scarce information about variation in non-reference gene duplicates, particularly in studies using short-read next-generation sequencing (NGS) data. Existing CNV calling tools solely detect excess coverage rather than sequence variants^19^. Furthermore, NGS alignment to a single reference genome contains ambiguity and bias^20^. Advances in single-molecule sequencing have enabled the generation of pangenomes from diverse populations with sequence-resolved CNVs^21–23^. Although reference bias may be reduced by representing pangenomes as a graph^24^, the variants distinguishing paralogs may be obscured by merged paralogous genes in the graph^25^. Furthermore, as pangenomes grow, diversity among populations, frequent gene conversion, and genome rearrangements present an even greater challenge^12^.

Here, we developed an approach to genotype sequence-resolved copy-number variation in pangenomes. This first required a method to catalog orthologous and paralogous copies of a given gene in a pangenome and organize them into equivalent groups by sequence similarity. This leverages the pangenome to represent complex genetic architectures that may not be simply represented by single variants. We next developed a method, ctyper, that uses the gene sequence catalogs to perform alignment-free genotyping of copy-numbers in NGS data. This approach overcomes reference alignment bias to provide sequence-resolved CNV maps in NGS cohorts, uncovering a new class of variation for biobank-scale analysis of genomes.

## Results

### Pangenome annotation and representation of pangenome-alleles

**Figure 1.**
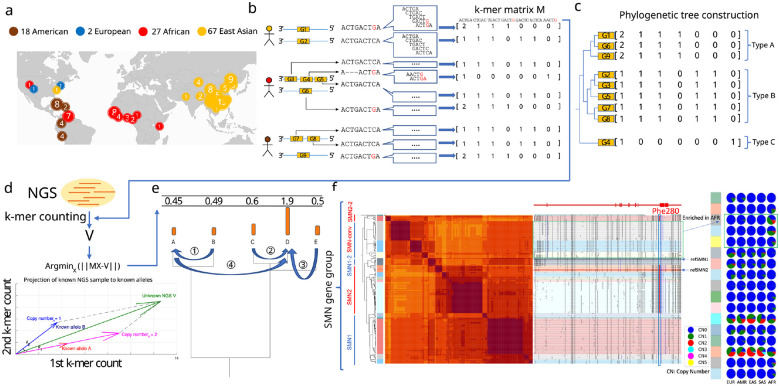
**a,** The demographic composition of the reference pangenome assemblies, HPRC (46 diploid), CPC (57 diploid), HGSVC (9 diploid), T2T-YAO (1 diploid), and CN1(1 diploid), as well as GRCh38 and CHM13. **b,** An overview of the approach to construct pangenome *k*-mer matrices for CNV genes. Each individual gene is represented as a vector of counts of *k*-mers exclusively found within the gene-group. All copies of genes including paralogs and orthologs are included and integrated as a *k*-mer matrix. **c,** Construction of phylogenetic trees based on *k*-mer matrices. **d,** Schematic of approach to estimate genotypes of alleles using NGS data. The *k*-mers from each matrix are counted in NGS data and normalized by sequencing depth. The normalized *k*-mer counts are projected to all pangenome genes. **e,** Reprojection to an integer solution based on the phylogenetic tree. **f,** An illustrative annotation and genotyping results on *SMN1/2* genes using HPRC samples. All *SMN* genes are categorized into 5 major allele-types and 17 sub allele-types. *SMN1*/*SMN2* correspond to the major allele-types of each paralog; *SMN1–2*, a copy of *SMN1* partially converted to *SMN2*; *SMN*-conv: additional converted SMN genes, mostly mapped to the *SMN2* locus, and is found to be enriched in African populations. The GRCh38 assembly includes *SMN1–2* and *SMN2*; SMN2–2: a rare outgroup of *SMN2*. On the right-side of the classification, the phylogenetic tree and heatmap of pairwise similarities are shown along with a mutant plot based on multiple sequence alignment highlighting point differences to *SMN1* in CHM13. Phe-280, the variant found to disrupt splicing of *SMN2* transcripts is highlighted. The genotyping results in 1KG continental populations is shown on the right.

We focused on genes previously annotated as CNV^21,23^ among 230 assemblies including HPRC, HGSVC, and CPC, two *de novo* telomere-to-telomere assemblies^26,27^, GRCh38 and CHM13^28^ (Fig. 1a). To construct databases used for querying genotypes, we annotated sequences with which CNV genes share homology across all assemblies, and extracted those sequences into pangenome alleles (PAs): genic segments containing locally phased variants, and that are combinatorially heritable (Fig. 1b). Homologous PAs are further organized into gene-groups. The counts of low copy *k*-mers (*k*=31) found exclusively in a gene-group are used to represent each PA and are combined by each gene-group into a matrix that is later used for genotyping. Each row of the matrix corresponds to a single PA, and columns contain the counts of each *k*-mer (Methods) (Fig. 1b). To genotype an NGS sample, ctyper first counts all *k*-mers from each gene-group in the NGS reads. It then identifies the combination of PAs as well as their individual copy number that is most similar to *k*-mers counts in the sample. This is achieved by projecting the NGS *k*-mer counts into the vector-space of the gene-group, and using phylogenetic rounding to determine an integer copy-number (Methods, Figs. 1c–e). As an example, the gene-group for *SMN* (the gene associates with spinal muscular atrophy) contains 178 PAs including copies of *SMN1* and *SMN2* as well as paralogs that have undergone gene conversion^29^ including genes found on the *SMN2* locus but identical to *SMN1* regarding phe-280, the SNP responsible for dysfunctional exon 7 splicing of *SMN2*^30^ (Fig. 1f).

**Figure 2.**
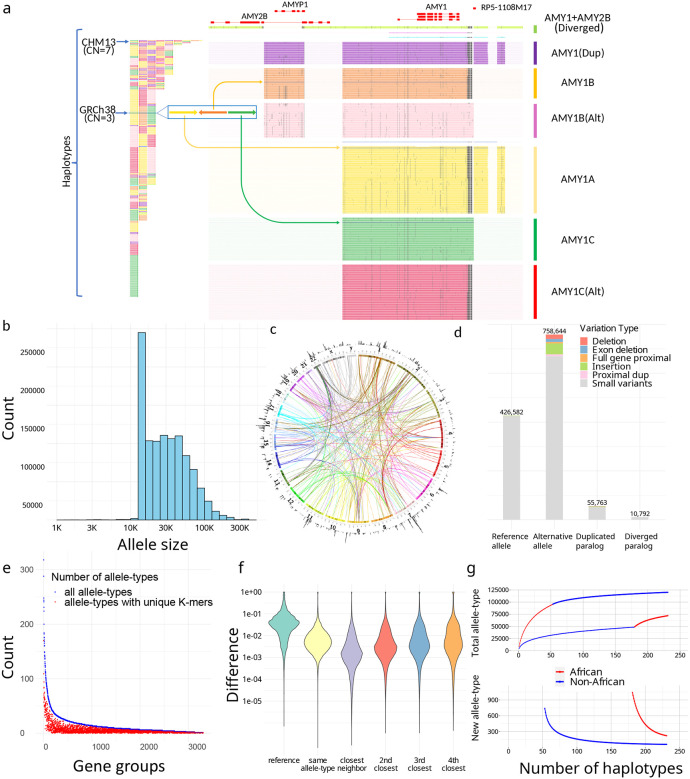
**a,** An overview of amylase 1 pangenome-alleles (PAs). (left) The corresponding order of all *AMY1* PAs on assemblies, which are colored based on their major allele-types. (right) *AMY1* genes are extracted as PAs as well as their flanking genes and sequences, including an *AMY2B* translocated proximal to *AMY1*, and two pseudogenes: *AMYP1* and *RP5–1108M17*. All PAs are vertically ordered according to the phylogenetic tree and aligned via graphic multiple sequence alignments (gMSA, Supplementary Methods). Homologous sequences are vertically aligned. Mutations are visualized as dots, and large gaps (deletions) are visualized as spaces. Seven major allele-types are categorized including five paralogs and two orthologs. There are no pseudogenes around *AMY1C*, while *AMY1A* has *RP5–1108M17* nearby and *AMY1B* has *AMYP1* nearby. There are alternative versions of *AMY1B and AMY1C*, with sequence substitutions. A new paralog called *AMY1(Dup)* found primarily on haplotypes with duplications, and has both pseudogenes nearby. The paralog of *AMY1* found with translocated *AMY2B* is called *AMY1+AMY2B*. There are also two rare paralogs (blue and violet) and one singleton ortholog (steel-blue). **b,** The size distribution of PAs on a log-density. The minimum sizes of PAs is 15kb, though smaller alleles may be annotated on alternative haplotypes on GRCh38 and as partial loci when dividing large genes into alleles without recombination. **c**. CIRCOS plot of all PAs. (outer ring) The density of PAs in each megabase on GRCh38. (arcs) Interchromosomal PAs included in the same groups. **d,** Annotation of PAs according to orthology and variants with respect to GRCh38. Duplicated paralogs are alleles with distal duplications and proximal duplications are included into Alternative alleles due to potential interaction with original genes. **e,** Identifiability of alle-types by unique *k*-mers. The total number of allele-types (blue), and the number of allele-types that may be identified by paralog-specific *k*-mers (red) are shown for each gene group with size at least three. **f,** The distribution of logistic pairwise distances of PAs depending on orthology and phylogenetic relationship. The values shown are average values from all gene-groups. Small neighbor distances are an indicator of strong representativeness of the current cohort. **g,** Saturation analysis for all allele-types using a recapture mode according to two sorted orders: African genomes considered first, and non-African genomes considered first.

Overall, 3,351 CNV genes (Supplementary Table 1) were classified into PAs that either contained the entire gene with flanking cis-elements for most genes, or were broken into smaller units not likely to be interrupted by recombination for large genes (Methods). In total, 1,408,209 PAs were defined and organized into 3,307 gene-groups (Figs. 2b–c). The average PA length was 33 ± 29kb, and included full genes (69%), processed pseudogenes (20%), intronic duplications (5%), and decoys (7%).

We annotated the proximity of CNVs with respect to corresponding reference genes (Methods). We discovered 164,237 PAs that are distal duplications (>20kb from source gene) on 6,389 loci and 6,673 PAs that contain proximal duplications (<20k from source gene), including 1,646 PAs that have runway duplications (at least three proximal duplications) on 36 genes^31^, for example the *HPR* locus (Supplementary Figure 1). We identified 10,792 PAs with diverged paralogs (<80% *k*-mer similarity with reference locus) from 333 gene-groups. For example, some amylase PAs contain paralogs of both *AMY1* and *AMY2B*, so are not classified as either (Fig. 2a). Based on proximity annotations, we defined orthologs and paralogs and further divided orthologs to reference-allele and alternative-allele and paralogs into duplicated alleles and diverged paralogs based on their similarity to reference genes (Methods).

To reduce genotype dimensionality for population analysis, highly similar PAs were merged into 89,236 allele-types (Methods). Allele-types have a median of 4 members but are skewed to large clusters: 50% PAs are in allele-types with at least 73 members (Supplementary Figure 2). The average pairwise *k*-mer similarity is 94.4% within each allele-type, compared to 78.0% within each gene-group, noting one base change can lead to *k* different *k*-mers. Between two phylogenetically neighboring allele-types with both having at least three members, the between-type variance is on average 6.03× greater than the within-type variance, showing strong stratification.

The ctyper genotyping estimates the PAs from the pangenome that are closest to genes contained by an NGS sample, along with their copy numbers. Thus the genotype of a gene-group is represented as a vector of PA-specific copy numbers (paCNVs). We compared the paCNVs to other representations of CNVs with less resolution of variants: copy-numbers of reference genes^1,31^, single unique nucleotide *k*-mers^1,31,32^ (SUNKs), and large haplotype sequences^13,33–35^. First, we characterized the information gained by representing a genome as paCNVs compared to copy-numbers of reference alleles. For each PA, we used the nearest neighbor in our pangenome database as a proxy for the optimal genotyping results of samples containing that PA, and its closest GRCH38 genes for comparison of single-reference based CNV. The nearest neighbor demonstrated an average 94.7% reduction in differences compared to GRCh38 matches; 57.3% had identical nearest neighbors showing common paCNVs alleles.

We then assessed the proportion of allele-types that may be identified by *k*-mers uniquely shared by all members of an allele-type, analogous to SUNKs. Only 38.8% allele-types (with at least three members) are represented by such *k*-mers (Fig. 2e). For example, no SUNKs are found between *SMN1*, *SMN2* and *SMN-converted* due to gene conversion (Fig. 1f), however there are unique combinations of *k*-mers used by ctyper genotyping.

We investigated the extent to which diversity is represented by large haplotypes structures by determining the number of singleton haplotypes with unique paCNVs that could not be represented by remaining haplotypes during leave-one-out tests. For the amylase locus, we found that 40% (90/226) of haplotypes cannot be represented, particularly those with greater copies than GRCh38 (45/67). When all PAs devoid of SV were combined into a single large allele-type, 20% (46/226) of haplotypes remained singleton, especially those with additional copies (26/67). Furthermore, many allele-types, such as the novel PAs containing both *AMY1* and *AMY2B* in proximity, are found within different structural haplotypes (Fig. 2a).

Finally, we performed saturation analysis using a recapture model^36,37^ to estimate the saturation of the current cohort in representing all possible allele-types among worldwide populations. This estimates the average number of novel allele-types within each new haplotype at different cohort sizes. Among the current cohort, each new African haplotype has 221 out of 4363 (5.1%) novel allele-types, and non-Africans have 56 out of 4358 (1.3%).

### Genotyping Pangenome-alleles among NGS samples and benchmarking results

**Figure 3.**
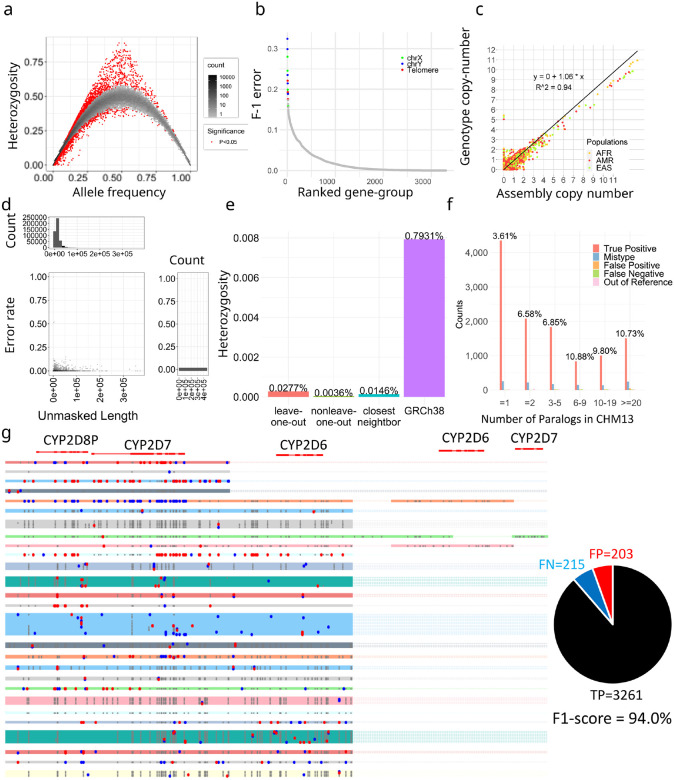
**a,** Hardy-Weinberg equilibrium of genotyping results on 1kgp unrelated samples. **b,** Genotype concordance of genotyping results on 1kgp trios, ordered by F-1 error. The gene groups with F-1 error more than 15% labeled by genomic location. **c,** Copy number comparison between assemblies and genotyping results on 1kgp unrelated samples. **d,** Sequence similarity between genotyped alleles and original alleles during leave-one-out test. Based on pairwise alignment of the region represented by *k-mers* (Unmasked region), the number of mismatched bases are reported and the error rate is the ratio of this value to the total length of the unmasked bases. **e,** Average mismatch rates of genotyped alleles to the ground truth. Leave-one-out genotype: test performed in (d); Non leave-one-out genotype: genotyping to the full pangenome set including the original sample; Closest neighbor: aligning the closest neighbor on the phylogenetic tree to alleles in query samples, indicating the optimal solution of genotyping in current cohort; GRCh38: Aligning the closest GRCh38 alleles to alleles in query samples, comparing it to Leave-one-out test can estimate the percentage of reference variants that can be represented by genotyping. **f,** Detailed leave-one-out comparison in the diploid T2T genome CN1. The results are categorized regarding the number of paralogs in CHM13 to show performances on different levels of genome complexity and the main sources of errors. **g,** Example leave-one-out genotyping results on *CYP2D6* genes (star alleles) including a mutant map based on gMSA for alleles of *CYP2D6* genes from 39 HPRC samples. Black dots show the true positive variants, red dots show false positive variants and blue dots show false negative variants based on sequence comparisons of genotyped alleles.

We applied ctyper to genotype NGS samples within the 1000 Genomes Project (1kgp) including 2,504 unrelated individuals and 641 offspring. The genotype accuracy was measured using Hardy-Weinberg Equilibrium (HWE), trio concordance (Supplementary Table 2), and comparisons to reference assemblies, excluding intronic/decoy PAs (Methods). There are significant HWE violations (p < 0.05) for 0.75% (1896 out of 252,817) of allele-types after excluding sex-chromosomes and setting the maximum copy-number to two (Fig. 3a). There are 27 gene-groups having >15% allele-types with significant disequilibrium, which are mostly small genes (median = 4,564 bp) with few unique *k*-mers (Supplementary Table 3). The average F-1 score for trio concordance is 97.58% (Fig. 3b), while 18 gene-groups have high discordance (>15%), primarily for subtelomeric genes or on sex chromosomes (Supplementary Table 4).

The paCNVs had an overall high agreement with assembly annotations (***ρ***=1.060) (Fig. 3c), where the discrepancy between genotyping and assembly annotation are largely due to low-quality or truncated genes excluded from our database; the high-quality genegroups without filtered sequences are more correlated (***ρ***=0.996).

We then assessed how well the genotyped alleles reflect the sample assembly using 39 HPRC samples having both NGS and assemblies. Each sample was genotyped with the database excluding its corresponding PAs (leave-one-out), or the full database (non-leave-one-out). Because genotypes are not phased, we used matching to assign the genotyped PAs to the corresponding assembly (Methods), excluding intron/decoys and sequences with <1kb unmasked bases, and measured the similarity between the genotyped allele and assigned query using global alignment^38^. We performed a similar analysis treating the closest neighbor from the database to each assembly PA as the correct genotyped locus. Across samples, 2.9% of PAs from the left-out assembly and 1.0% PAs from non-leave-one-out could not be paired, which is primarily due to missing-typing, assembly-error or copy number error. Using the full database, paired PAs have 0.36 mismatches per 10kb with 93.0% having no mismatches on nonrepetitive regions. The leave-one-out have 2.7 mismatches per 10kb on nonrepetitive regions, with 57.3% alleles having no mismatches, and 77.0% were mapped to the optimal solution (Fig. 3d). The leave-one-out results were on average 96.5% closer to the original PAs compared to the closest GRCh38 gene at 79.3 mismatches per 10kb, indicating sufficient diversity for accurate representation of new samples (Fig. 3e).

To isolate sources of errors in cases of misassembled duplications, we directly compared leave-one-out genotyping results to a telomere-to-telomere phased assembly, filtering out intronic/decoy sequences. The sample genotypes had 11,627 correctly matched allele-types, 599 (4.8%) mistyped to other allele-types, 131 out-of-reference (1.1%), 127 FP (false-positive) (0.5% F-1), 93 FN (false-negative) (0.4% F-1) for a total F-1 error of 6.7% (Methods) (Fig. 3f), showing most errors are not copy number errors and a 3% increase in mistyped on this genome compared to trio discordance.

We examined the *CYP2D* genotypes including *CYP2D6* (star-allele) to assess accuracy at a medically relevant locus associated with drug resistance^39^ (Methods). All CNVs were correctly called. The SNPs identified through genotyping show an F1-score of 94.0% (95.7% for CYP2D6 region only), outperforming Aldy^40^ at 85.2% (Fig. 3g). Regarding the allele-types, 4 out of 74 (5.4%) were out-of-reference, and 4 were mistyped.

### Sequence level diversity of CNVs in global populations

**Figure 4.**
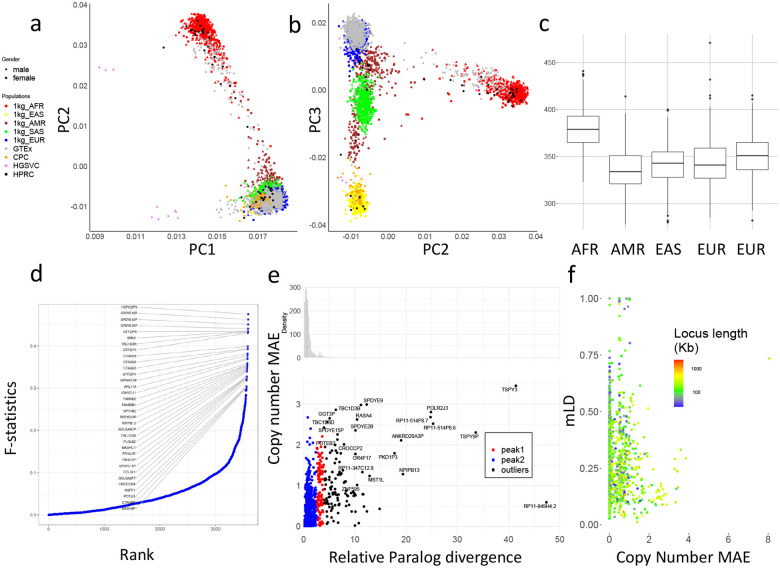
**a,b**. PCA of allele-specific copy numbers on genotype results and assembly annotations. **c,** Distribution of total autosomal gene copy numbers among 2504 unrelated 1kgp samples. **d,** Population differentiation measured by F-statistics of allele-types among different continental populations. The genes with an allele-type with an F-statistic more than 0.3 are labeled. **e,** Copy number and relative paralog divergence. Based on our genotyping results on 2504 unrelated 1kgp, for genes found to be CNV to the population median in more than 20 samples, we determined the average aggregate copy number difference (MAE) between individuals and estimated the average paralog differences relative to orthologs difference. **f,** Multi-allelic linkage disequilibrium between pairs of CNV genes less than 100kb apart. The largest MAE value of each pair is used for the x-axis values. The total locus length denotes the length from the beginning of the first gene to the end of the last gene.

We used principal component analysis (PCA) to examine the population structure of PA genotypes on 2,504 unrelated 1kgp samples, 879 Genotype-Tissue Expression (GETx) samples, and 114 diploid assemblies (Figs. 4a,b). We filtered low frequency (<0.05) allele-types and capped copy numbers at 10. The 1kgp, GETx and genome assemblies were clustered by population as opposed to data source, suggesting little bias between genotyping and assembly, as well as across different cohorts. However, the HGSVC assemblies show as outliers on PC1, possibly due to unassembled or filtered alleles.

The top 0.1% highest weighted allele-types on PC1 have an average aggregate copy number (aggreCN) variance of 26.33, compared to an overall of 4.00 (p-value=1.11e-16, F-test). Similarly, PC2 and PC3 have mean aggreCN variance of 19.73 and 7.20, suggesting CNVs are weakly associated with sequence variants. Furthermore, PC1 is the only PC that clustered all samples into the same sign with a geographic center away from 0, suggesting it corresponds to modulus variance (hence aggreCN) if we are treating samples as vectors of paCNVs. Meanwhile, PC2 and PC3 are similar to the PCA plots based on SNP data on global samples^41^, suggesting they are associated with the sequence diversity among those CNV genes. The total number of duplication events are elevated in African populations (Fig. 4c), reflected in the order of PC1 (Fig 4a).

We next used the F-statistic that is similar to the F_st_ but accommodates more than two genotypes (Methods) to test the differences in copy number distributions across five continental populations (Fig. 4d). In total, 223 out of 5065 (4.4%) of duplicated allele-types showed population specificity (F-statistic > 0.2, Supplementary Table 5). The alleletype with the highest F-statistic (0.48) contains duplications of the *HERC2P9* gene that is known to have population differentiation^9,42^. Another example of a highly differentiated allele-type is a converted copy of *SMN2* annotated as a duplication of *SMN1* that is enriched in African populations (F-statistic=0.43).

We then measured whether duplicated genes were similar or diverged from reference copies, which would indicate recent or ancient duplications, and provide a measure on reference bias from missing paralogs. We used multiple sequence alignments (Methods) for each gene group to determine the pairwise differences at non-repetitive homologous sequences. We determined the average paralog divergence relative to ortholog divergence (Methods), which we refer to as relative paralog divergence (RPD). We also measured diversity by the mean absolute error (MAE) of the gene copy number in the populations (Fig. 4e). Based on RPD, using Density-Based Spatial Clustering of Applications with Noise^43^, we identified two peaks at 0.71 and 3.2, with MAE centers at 0.18 and 0.93. The first peak indicates genes with rare and contemporary CNVs, while the second peak indicates more divergent and common CNVs, often CNVs that may be inherited as different structural haplotypes. For example, *AMY1A* has a high RPD at 3.10 because of the truncated duplications of *AMY1A* (blue gene annotations in Fig. 2a). These results are consistent with ancient bursts of duplications in humans and primate ancestors^44^.

We next studied haplotype linkage of PAs to investigate if gene duplications could be represented by a limited number of distinct structural haplotypes, or if paralogs are decoupled by recombination. We determined multi-allelic linkage disequilibrium (mLDs) between PAs using the 1kg genotypes^45^ (Methods). We computed mLDs for 989 allele-types that were adjacent and less than 100kb apart on GRCh38 (Fig. 4f), and found the average within each gene-group. Among all mLDs, there was a strong negative rank correlation between MAEs of the copy number and mLD (***ρ***=−0.24, p-value=3.4e-15, Spearman’s rank), which is stronger than the rank correlation between MAEs of gene copy number and total locus length (***ρ***=−0.21, p-value = 1.5e-11), suggesting a reduced haplotype linkage on genes with frequent CNVs. The lowest mLD=0.013 found on *FAM90*, a gene with frequent duplications and rearrangements^46^. Not surprisingly, the 29 highest loci (mLDs > 0.7) are enriched in the sex chromosomes (N=19). Furthermore, *HLA-B* and *HLA-DRB*, had mLD >0.7 and only copy-number variation by deletion. The *HLA-DRB* deletions were only apparent after correcting HLA-specific coverage bias (Supplementary Methods). The *amylase* locus has a value of 0.293 due to a high degree of recombination (Fig. 1a).

### Expression quantitative trait locus (eQTLs) on pangenome alleles

To investigate the expression impact of paCNVs, we performed eQTL analysis with two RNA-seq cohorts: Geuvadis^47^ and the GTEx^48^. There were 4,512 genes that could be uniquely mapped in RNA-seq alignments, and 44 without unique sequences such as *SMN1/2* and *AMY1A/1B/1C* (Methods, Supplementary Table 6), for which expression was pooled among indistinguishable copies for eQTL analysis. Genes after pooling together each of those with unique regions are called gene-units.

**Figure 5.**
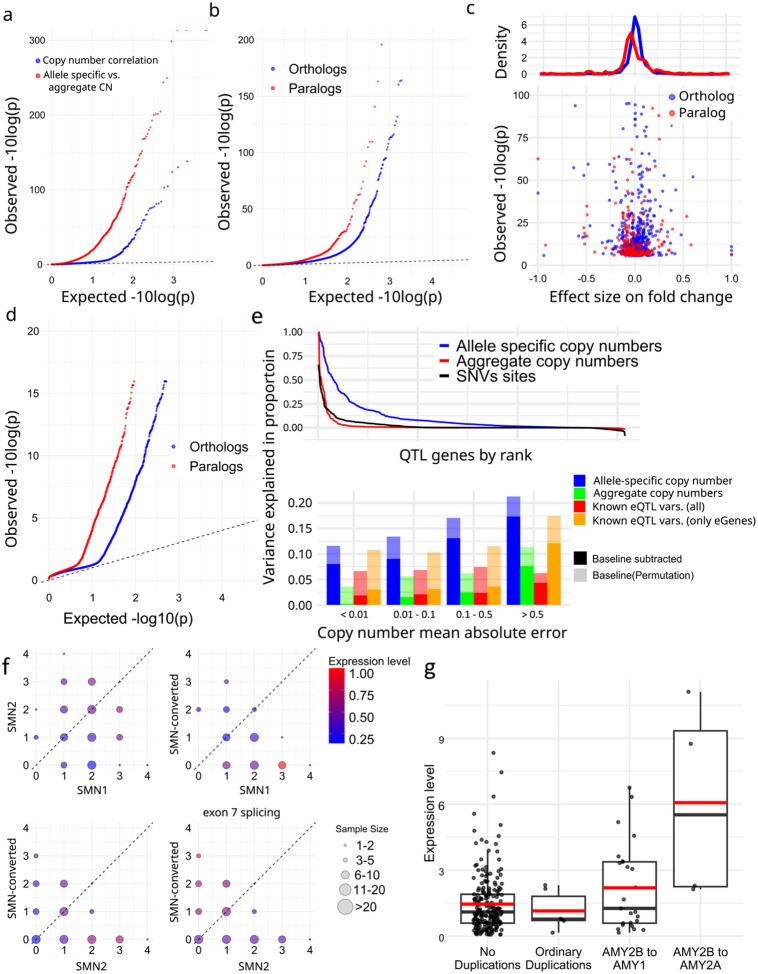
**a.** Q-Q plot of association of aggregate (*blue*) and allele-specific (*red*) copy numbers to gene expression in Geuvadis samples. **b,** Comparative gene expression of orthologs (*blue*) and paralogs (*red*). **c,** Fold change effect size of all alternative expressions. For all allele-types found to be significant, the fold changes as well as pvalues were shown. **d,** Preferential tissue expression of orthologs and paralogs. **e,** (top), Model evaluation for PAs representing gene expression diversities. (bottom) Quantification of variance explained by different representations of genomic diversity: full paCNV genotypes, aggregate copy number, and known eQTLs variants. **f,** Case study on *SMN* genes showing decreased gene expression on converted *SMN*. The average corrected expression level in Geuvadis samples is shown under different copy numbers of *SMN1*, *SMN2*, and converted *SMN*. Transcript levels are the total coverage of all isoforms, and exon 7 splicing level is measured by counting isoforms with a valid exon 7 splicing junction. **g,** Case study on amylase genes showing increased gene expression on translocated *AMY2B*.

We corrected expression bias using PEER^49^ with the first three PCs from reported genotypes^50^, and performed association analyses with paCNVs. After merging paCNs to aggreCNs, we found 178 out of 3,224 gene-units (5.5%) showed significance (corrected-p = 1.6e-0.5, Pearson-correlation) as previously observed^31^. We then tested whether using paCNVs would provide a stronger fit by updating the aggreCNs with individual paCNVs and performing multivariable linear regression on expression (Methods). We found significant improvements in fitting for 890 gene-units (27.6%) (corrected p=1.6e-05, one-tailed F-test) (Fig. 5a).

The improved fit could be explained by non-uniform effects on expression of alleles in the same gene-unit. To test this, we used a linear mixed model (LMM, Methods)^51,52^ to regress total expression to individual allele-types to estimate allele-specific expression levels, and compared these values to their peers (Supplementary Table 7). For alleletypes within solvable matrices and found in more than 10 samples, we found that 150 out of 1,890 paralogs (7.94%) and 546 out of 16,628 orthologs (3.28%) had significantly different expression levels (corrected with sample size = number of paralogs + orthologs, corrected-p = 2.7e-06, Chi-squared test, Fig. 5b). Overall, paralogs are found to have reduced expression (Fig. 5c), consistent with previous findings on duplicated genes^53^.

Using GTEx, we compared across 57 tissues to see if allele-types had different most expressed tissues than their peers. Similarly, we applied LMM analysis to estimate the expression levels on each tissue (Methods, Supplementary Table 8). We found alternative tissue specificity for 132 of 2,820 paralogs (4.7%) and 225 of 19,197 orthologs (1.2%) (corrected-p = 6.4e-08, union of two Chi-squared tests, Methods, Fig. 5d).

Additionally, we used analysis of variance (ANOVA) to estimate the proportion of expression variance explained by paCNVs using Geuvadis, and compared it to a model based on known conventional eQTL variants^54^ including SNPs, indels and SVs (Methods). As expected, the highly granular paCNVs explain the most variance: on average, 10.3% (14.3% including baseline). In contrast, 58.0% of gene-units are eGenes with known eQTL variants that in total explained valid variance by 2.14% (1.60% considering experimental noise, in agreement with a previous estimate of 1.97%^55^). On average, 1.98% of the variance was explained by aggreCNs, and 8.58% was explained by allele-type information. When combining both paCNVs and known eQTL sites, 10.4% (19.0% including baseline) of the valid variance was explained (Fig. 5e).

We examined *SMN* and *AMY2B* genes as case studies due to their importance in disease and evolution^30,56^. The *SMN* genes were classified into three categories: *SMN1*, *SMN2*, and the previously mentioned *SMN-converted*. We estimated both the total expressions of all transcripts and the expressions of only isoforms with valid exon 7 splicing junctions. For total expression, no significant difference was found between *SMN1* and *SMN2* (0.281 ± 0.008 vs 0.309 ± 0.009, p=0.078, Chi-squared test). However, significant differences were found between *SMN-converted* and *SMN1/2* (0.226 ± 0.012 vs 0.294 ± 0.002, p=1.75e-07, Chi-squared test), with a 23.0% reduction in expression of *SMN-converted*. In contrast, despite with lower overall expression, *SMN-converted* had 5.93× the expression of *SMN2* (p=2.2e-16, Chi-square test) regarding valid exon 7 splicing, indicating while *SMN-converted* has full functional splicing^57^, its overall expression level is lower (Fig. 5f).

For *AMY2B*, we studied the expressions of duplications when they are translocated to proximal to other *AMY* genes, such as the PAs containing *AMY1* and *AMY2B* at figure 2a. Using GTEx pancreas data, we estimated their expressions as well as other duplications. We found that these translocated *AMY2B* genes had significantly higher expression than other duplications (1.384 ± 0.233 vs −0.275 ± 0.183, p=7.87e-09, Chi-squared test) (Fig. 5g).

## Discussion

New pangenomes present both opportunities and challenges for the study of complex genetic variation: while they reveal the landscape of complex variation, it is challenging to use these sequences to analyze biobank (NGS) cohorts. To enable this, we developed an approach to divide assemblies into pangenome-alleles: sequences that are copy number variable and inherited with low disequilibrium in gene families, and to genotype their copy number in NGS samples.

The use of ctyper genotypes increases the scope of studies on CNVs to include sequence variation between copies. For example, our finding that CNVs reflect two modes of variation: high-identity (and likely recent), and low-identity (ancient and polymorphic) duplications, is based on large cohort ctyper genotypes rather than assembly annotations. As another example, the ctyper genotypes yield tissue-specific expression of paralogs as well as relative contributions to expression of different forms of duplications such as *SMN*.

We investigated the reasons behind the disparity between the ANOVA on PAs versus known eQTL variants. PAs represent variants in multi-allelic form to preserve assembly information while eQTL variants are standard bi-allelic variants. First, in contrast to PAs, there were either very few or very many eQTLs variants per gene, indicating LD (Supplementary Figure 3) as addressed by fine-mapping^58^, increasing multiple testing burden^59^. Additionally, there was a greater proportion of variance explained among genes with more CNVs by eQTL variants, which is contrary to the fact that variant calling is more challenging among CNV genes^60^. This could also be explained by indirect association by LD (for example the *HPR* genes, Supplementary Figure 1). Furthermore, as the frequency of CNVs increase, the explained variance by eQTL variants increases (t= 3.80, p-value = 1.6e-04), while the number of eQTL variants decreases (t = −4.79, p-value = 2.1e-06), suggesting that larger effects like CNVs might overshadow the discovery of other not linked variants. The larger effects will increase uncertainty, which reduces the association and weakens the confidence for other variants with smaller effects that are not linked with larger effect variants. Another potential reason is that gene expression might not be a simple linear additive effect of all variants^61^. For example, although *SMN*-converted contains variants that are either from *SMN1* or *SMN2*, its overall expression is lower than both. In this manner, the concept of PAs may have a wider potential for genome-wide association analysis (including non-CNV genes) in the future.

Due to limited sample size, our associations are at the level of allele-types rather than individual PAs. Different cohort sizes may require different levels of granularity when defining allele-types. For example, the three allele-types of *SMN-converted* showed little difference in expression. Our current classification on allele-types was designed to work on large biobank cohorts, so smaller cohorts may need to test on allele-types that aggregate more PAs. The granularity of genotyping is additionally defined by the length of PA sequences; genotypes using shorter PAs will more accurately reflect NGS samples, while longer sequences can preserve phase and may be preferable in regions with low recombination such as *HLA-DRB*.

Ctyper also has limitations. First, while it is possible to detect CNVs smaller than PA units using ctyper (Supplementary Methods), it is not currently supported due to lack of benchmarking data. Second, while each PA reflects a locally phased gene, the genotypes are not phased at larger scale. Third, ctyper currently does not provide confidence values for results. Finally, the high-dimensionality of PAs increases the complexity of interpretation and the need for large sample sizes to support associations on multiallelic variants.

The average runtime for genotyping at 30x coverage was 80.2 minutes on a single core (Supplementary Figure 4), indicating that ctyper is suitable for biobank analysis. As new high-quality references become available, we anticipate ctyper to be a useful method for interpreting the association between CNV and traits at scale.

## Supplementary Material

Supplement 1

1

## Data Availability

Software is available at https://github.com/ChaissonLab/Ctyper. Pangenome allele matrices and annotations are available at https://doi.org/10.5281/zenodo.13381931.
