# Genotyping sequence-resolved copy-number variations using pangenomes reveals paralog-specific global diversity and expression divergence of duplicated genes

**DOI:** 10.1101/2024.08.11.607269

**Published:** 2024-10-15

**Authors:** Walfred Ma, Mark JP Chaisson

**Affiliations:** 1 Quantitative and Computational Biology, University of Southern California, CA, USA.; 2 The Genomic and Epigenomic Regulation Program, USC Norris Cancer Center, University of Southern California, Los Angeles, California 90033, USA

## Abstract

Copy-number variable (CNV) genes are important in evolution and disease, yet sequence variation in CNV genes is a blindspot for large-scale studies. We present a method, ctyper, that leverages pangenomes to produce copy-number maps with allele-specific sequences containing locally phased variants of CNV genes from NGS reads. We extensively characterized accuracy and efficiency on a database of 3,351 CNV genes including *HLA*, *SMN*, and *CYP2D6* as well as 212 non-CNV medically-relevant challenging genes. The genotypes capture 96.5% of underlying variants in new genomes, requiring 0.9 seconds per gene. Expression analysis of ctyper genotypes explains more variance than known eQTL variants. Comparing allele-specific expression quantified divergent expression on 7.94% of paralogs and tissue-specific biases on 4.7% of paralogs. We found reduced expression of *SMN-1* converted from *SMN-2,* which potentially affects diagnosis of spinal muscular atrophy, and increased expression of a duplicative translocation of *AMY2B*. Overall, ctyper enables biobank-scale genotyping of CNV and challenging genes.

## Introduction

Human genomes are characterized by frequent duplications and deletions, leading to copy number variation (CNV). Up to 10% of protein-coding genes are known to be copy-number variable, showing distinct distributions across human populations^1,2^ and association with traits such as body mass index^3^ and disease including cancer^4^, cardiovascular diseases^5^, and neurodevelopmental disorders^6,7^. While CNVs are infrequent genome-wide, regions of long, low-copy repeats called segmental duplications (SDs) are enriched in genes and are catalysts for frequent CNVs^8,9^. This leads to diverse gene families such as *TBC1D3*, *NPIP*, and *NBPF*^10,11^. The mechanisms contributing to CNVs, along with the elevated mutations in SDs^12^, result in variation not only in aggregate copy number (aggreCN) but also elevated sequence variation among the copies^12–14^. This variation can influence phenotypes and disease susceptibility^15–17^, including hypertension and type 2 diabetes^18^. Many CNV genes in SDs are found to be human specific, quickly evolving, and highly associated with brain function^19–21^.

There is scarce information about variation in non-reference gene duplicates, particularly in studies using short-read next-generation sequencing (NGS) data. Existing CNV calling tools detect excess coverage rather than sequence variants^22^. Furthermore, NGS alignment to a reference genome contains ambiguity and bias^23^. Advances in single-molecule sequencing enabled assembly of pangenomes from diverse populations with sequence-resolved CNVs^24–26^. Although reference bias may be reduced using graph pangenomes^27^, the variants distinguishing paralogs may be obscured during graph construction^28^. Furthermore, as pangenomes grow, diversity among populations, frequent gene conversion, and genome rearrangements present an even greater challenge^12^.

Here, we developed an approach to genotype sequence-resolved copy-number variation, providing copy-number maps with locally-phased variation for each copy. Our method, ctyper, uses alignment-free genotyping to call copy-number and allele-specific variants from NGS data leveraging a database of gene sequences derived from pangenome assemblies. This overcomes reference alignment bias and uncovers variation missed from single reference analysis and limitation of NGS alignments in repetitive regions. The efficiency of ctyper enables scaling of this analysis to biobank data.

## Results

### Pangenome annotation and representation of pangenome-alleles

**Figure 1.**
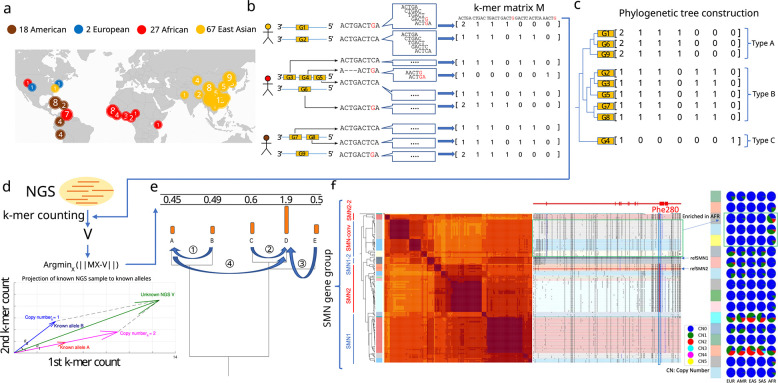
**a,** Demography of the reference pangenome assemblies, HPRC (46 diploid), CPC (57 diploid), HGSVC (9 diploid), T2T-YAO (1 diploid), and CN1(1 diploid), as well as GRCh38 and CHM13. **b,** Construction of pangenome *k*-mer matrices for CNV genes. Each individual gene is represented as a vector of counts of *k*-mers exclusively found within the gene-group. All copies of genes including paralogs and orthologs are included and integrated as a *k*-mer matrix. **c,** Construction of phylogenetic trees based on *k*-mer matrices. **d,** Schematic of approach to estimate genotypes of alleles using NGS data. The *k*-mers from each matrix are counted in NGS data and normalized by sequencing depth. The normalized *k*-mer counts are projected to all pangenome genes. **e,** Reprojection to an integer solution based on the phylogenetic tree. **f,** An illustrative annotation and genotyping results on *SMN1/2* genes using HPRC samples. All *SMN* genes are categorized into 5 major allele-types and 17 sub allele-types. *SMN1*/*SMN2* correspond to the major allele-types of each paralog; *SMN1–2*, a copy of *SMN1* partially converted to *SMN2*; *SMN*-conv: additional converted SMN genes, mostly mapped to the *SMN2* locus, and is found to be enriched in African populations. The GRCh38 assembly includes *SMN1–2* and *SMN2*; SMN2–2: a rare outgroup of *SMN2*. On the right-side of the classification, the phylogenetic tree and heatmap of pairwise similarities are shown along with a mutant plot based on multiple sequence alignment highlighting point differences to *SMN1* in CHM13. Phe-280, the variant found to disrupt splicing of *SMN2* transcripts is highlighted. The genotyping results in 1KG continental populations is shown on the right.

We focused on genes previously annotated as CNV^24,26^ among 230 assemblies from the HPRC, HGSVC, and CPC, two telomere-to-telomere assemblies^29,30^, GRCh38 and CHM13^31^ (Fig. 1a). To construct databases used for querying genotypes, we annotated sequences with which CNV genes share homology across all assemblies, and extracted those sequences into pangenome alleles (PAs): genic segments containing locally phased variants, similar to an HLA allele^32^ (Fig. 1b). Homologous PAs were further organized into gene-groups. The counts of low copy *k*-mers (*k*=31) found exclusively in a gene-group are used to represent each PA and are combined by each gene-group into a matrix that is later used for genotyping. Each row of the matrix corresponds to a single PA, and columns contain the counts of each *k*-mer (Methods) (Fig. 1b). To genotype an NGS sample, ctyper first counts all *k*-mers from each gene-group in the sample. It then identifies a combination of PAs as well as their copy number that is most similar to *k*-mers counts of the sample. This is achieved by projecting the NGS *k*-mer counts into the vector-space of the gene-group, and using phylogenetic rounding to determine an integer copy-number (Methods, Figs. 1c–e). As an example, the gene-group for *SMN* (the gene associates with spinal muscular atrophy) contains 178 PAs including copies of *SMN1* and *SMN2* as well as paralogs that have undergone gene conversion^33^ including genes found on the *SMN2* locus containing the *SMN1* phe-280, the SNP responsible for dysfunctional exon 7 splicing of *SMN2*^34^ (Fig. 1f).

**Figure 2.**
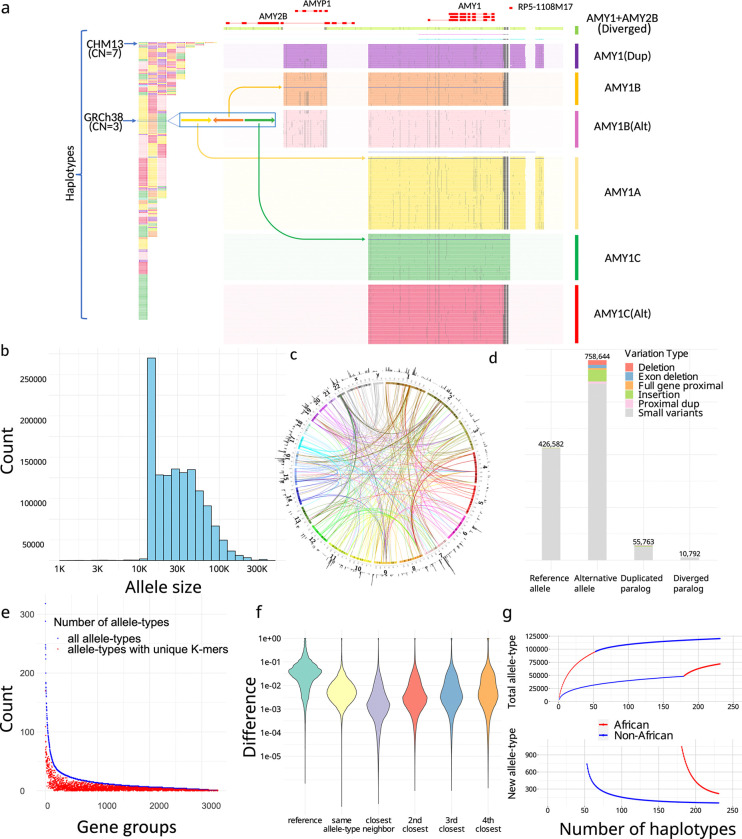
**a,** An overview of amylase 1 pangenome-alleles (PAs). (left) The corresponding order of all *AMY1* PAs on assemblies, which are colored based on their major allele-types. (right) *AMY1* genes are extracted as PAs as well as their flanking genes and sequences, including an *AMY2B* translocated proximal to *AMY1*, and two pseudogenes: *AMYP1* and *RP5–1108M17*. All PAs are vertically ordered according to the phylogenetic tree and aligned via graphic multiple sequence alignments (gMSA, Supplementary Methods). Homologous sequences are vertically aligned. Mutations are visualized as dots, and large gaps (deletions) are visualized as spaces. Seven major allele-types are categorized including five paralogs and two orthologs. There are no pseudogenes around *AMY1C*, while *AMY1A* has *RP5–1108M17* nearby and *AMY1B* has *AMYP1* nearby. There are alternative versions of *AMY1B and AMY1C*, with sequence substitutions. A new paralog called *AMY1(Dup)* found primarily on haplotypes with duplications, and has both pseudogenes nearby. The paralog of *AMY1* found with translocated *AMY2B* is called *AMY1+AMY2B*. There are also two rare paralogs (blue and violet) and one singleton ortholog (steel-blue). **b,** The size distribution of PAs on a log-density. The minimum sizes of PAs is 15kb, though smaller alleles may be annotated on alternative haplotypes on GRCh38 and as partial loci when dividing large genes into alleles without recombination. **c**. CIRCOS plot of all PAs. (outer ring) The density of PAs in each megabase on GRCh38. (arcs) Interchromosomal PAs included in the same groups. **d,** Annotation of PAs according to orthology and variants with respect to GRCh38. Duplicated paralogs are alleles with distal duplications and proximal duplications are included into Alternative alleles due to potential interaction with original genes. **e,** Identifiability of alle-types by unique *k*-mers. The total number of allele-types (blue), and the number of allele-types that may be identified by paralog-specific *k*-mers (red) are shown for each gene group with size at least three. **f,** The distribution of logistic pairwise distances of PAs depending on orthology and phylogenetic relationship. The values shown are average values from all gene-groups. Small neighbor distances are an indicator of strong representativeness of the current cohort. **g,** Saturation analysis for all allele-types using a recapture mode according to two sorted orders: African genomes considered first, and non-African genomes considered first.

Overall, 3,351 CNV genes (Supplementary Table 1) were classified into PAs that either contained the entire gene with flanking cis-elements, or were broken into smaller units not likely to be interrupted by recombination for larger genes (Methods). In total, 1,408,209 PAs were defined and organized into 3,307 gene-groups (Figs. 2b–c). The average PA length was 33 ±29 kb, and included full genes (69%), processed pseudogenes (20%), intronic duplications (5%), and decoys (7%) .

We annotated the proximity of CNVs with respect to corresponding reference genes (Methods). Overall, 164,237 PAs are distal duplications (>20kb from source gene) in 6,389 loci, and 6,673 PAs that contain proximal duplications (<20k from source gene), including 1,646 PAs that have runway duplications (at least three proximal duplications) on 36 genes^35^, for example the *HPR* locus (Supplementary Figure 1). We identified 10,792 PAs with diverged paralogs (<80% *k*-mer similarity with reference locus) from 333 gene-groups. For example, some amylase PAs contain paralogs of both *AMY1* and *AMY2B*, so are classified distinctly (Fig. 2a). The PAs were defined as reference-orthologs or paralogs by reference alignment. Orthologs were subdivided into reference-allele and alternative-allele by variation, and paralogs into duplicated or diverged alleles based on their similarity to reference genes (Methods).

To reduce genotype dimensionality for population analysis, highly similar PAs were merged into 89,236 allele-types (Methods). Allele-types have a median of 4 members but are skewed to large clusters: 50% PAs are in allele-types with at least 73 members (Supplementary Figure 2). The average pairwise *k*-mer similarity is 94.4% within each allele-type, compared to 78.0% within each gene-group, noting one base change adds up to *k* different *k*-mers. Between two phylogenetically neighboring allele-types having at least three members each, the between-type variance is 6.03× greater than the within-type variance, showing strong stratification.

The genotype of a gene-group is a vector of PA-specific copy numbers (paCNVs). We compared the paCNVs to other representations of CNVs with lower resolution of variants: copy-numbers of reference genes^1,35^, single unique nucleotide *k*-mers^1,35,36^ (SUNKs), and large haplotype sequences^13,37–39^. First, we characterized the information gained by representing a genome as paCNVs compared to copy-numbers of reference alleles. For each PA, we used the nearest neighbor in our pangenome database as a proxy for the optimal genotyping results of samples containing that PA, and its closest GRCH38 genes for comparison of single-reference based CNV. The nearest neighbor demonstrated an average 94.7% reduction in differences compared to GRCh38 matches; 57.3% had identical nearest neighbors showing common paCNVs alleles.

We then assessed the proportion of allele-types identifiable by *k*-mers uniquely shared by all their members, analogous to SUNKs. Only 38.8% allele-types (with at least three members) contain such *k*-mers (Fig. 2e). For example, no SUNKs are found between *SMN1*, *SMN2* and *SMN-converted* due to gene conversion (Fig. 1f), however there are unique combinations of *k*-mers used by ctyper genotyping.

We investigated the extent to which diversity is represented by large haplotypes structures by determining how allelic variation and recombination creates unique combinations of amylase genes that cannot be represented during leave-one-out analysis. There were 40% (90/226) of haplotypes that could not be represented, particularly those with greater copies than GRCh38 (45/67). When all PAs devoid of SV were combined into a single large allele-type, 20% (46/226) of haplotypes remained singleton, especially those with additional copies (26/67). Furthermore, many allele-types, such as the novel PAs containing both *AMY1* and *AMY2B* in proximity, are found within different structural haplotypes (Fig. 2a). While such issues may be mitigated by a larger pangenome, genotyping at the level of PA increases the ability to identify the genetic composition of an NGS sample at highly variable multicopy gene loci.

Finally, we performed saturation analysis using a recapture model^40,41^ to estimate the extent to which the current cohort represents all possible allele-types among worldwide populations. This estimates the average number of novel allele-types within each new genome at increasing cohort sizes. Among the current cohort, each new African genome has 221 of 4363 (5.1%) novel allele-types, and non-Africans have 56 of 4358 (1.3%).

### Genotyping Pangenome-alleles among NGS samples and benchmarking results

**Figure 3.**
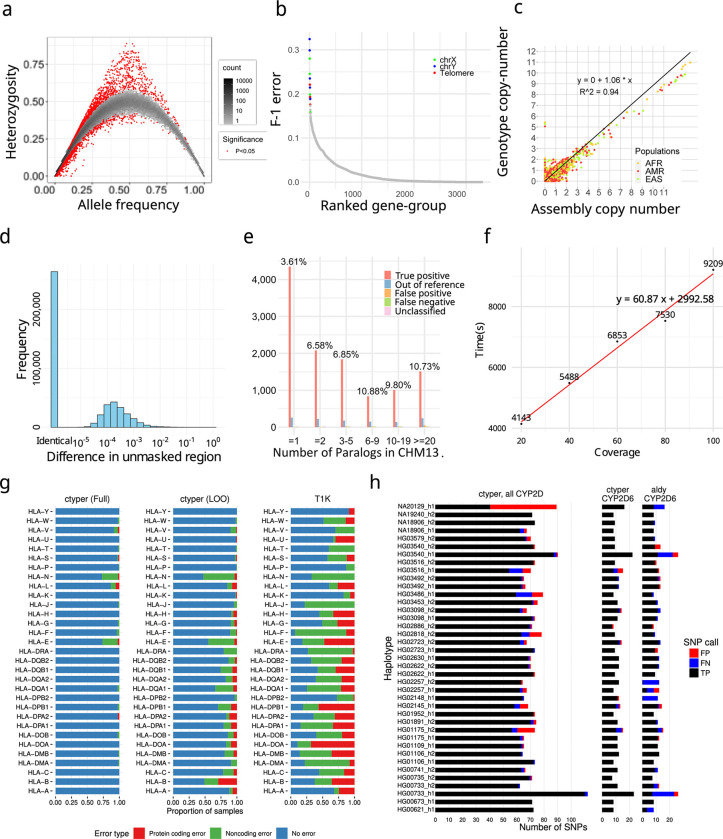
**a,** Hardy-Weinberg equilibrium of genotyping results on 1kgp unrelated samples. **b,** Genotype concordance of genotyping results on 1kgp trios, ordered by F-1 error. The gene groups with F-1 error more than 15% labeled by genomic location. **c,** Copy number comparison between assemblies and genotyping results on 1kgp unrelated samples. **d,** Sequence differences between genotyped and original alleles during leave-one-out test using on Stretcher pairwise alignment of non-repetitive sequences. **e,** Detailed leave-one-out comparison in the diploid T2T genome CN1. The results are categorized regarding the number of paralogs in CHM13 to show performances on different levels of genome complexity and the main sources of errors. **f** Runtime of ctyper on CN00001 on all loci for varying coverage. **g,** Benchmarking of *HLA* genotyping using ctyper on full, and leave-one-out (LOO) databases, compared with T1K on 31 *HLA* genes. **h,** Benchmarking of *CYP2D* annotation on all *CYP2D* genes and *CYP2D6* exclusively.

We applied ctyper to genotype NGS samples within the 1000 Genomes Project (1kgp) including 2,504 unrelated individuals and 641 offspring. Accuracy was measured using Hardy-Weinberg Equilibrium (HWE), trio concordance (Supplementary Table 2), and comparisons to reference assemblies, excluding intronic/decoy PAs (Methods). There are significant HWE violations (p < 0.05) for 0.75% (1896/252,817) of allele-types after excluding sex-chromosomes and setting the maximum copy-number to two (Fig. 3a). There are 27 gene-groups having >15% allele-types with significant disequilibrium, which are mostly small genes (median = 4,564 bp) with few unique *k*-mers (Supplementary Table 3). The average F-1 score for trio concordance is 97.58% (Fig. 3b), while 18 gene-groups have high discordance (>15%), primarily for subtelomeric genes or on sex chromosomes (Supplementary Table 4).

The paCNVs had an overall high agreement with assembly annotations (***ρ***=1.060) (Fig. 3c), where the discrepancy between genotyping and assembly annotation are largely due to low-quality or truncated genes excluded from our database; high-quality gene-groups without filtered sequences are more correlated (***ρ***=0.996).

We then assessed how well the genotyped alleles reflect the sample assembly using 39 HPRC samples having both NGS and assemblies. Each sample was genotyped with the full database (full-set) or the database excluding its corresponding PAs (leave-one-out). We used a matching script to assign the genotyped PAs to the corresponding assembly (Methods), excluding intron/decoys and sequences with <1kb unmasked bases, and measured the similarity between the genotyped allele and assigned query using global alignment^42,43^. We performed a similar analysis treating the closest neighbor from the database to each assembly PA as the correct genotyped locus. Across samples, 2.9% of PAs from the leave-one-out assembly and 1.0% PAs from full-set could not be paired, which is primarily due to miss-typing, assembly-error or copy number error. Using the full-set, paired PAs have 0.36 mismatches per 10kb with 93.0% having no mismatches on less repetitive regions. The leave-one-out have 2.7 mismatches per 10kb on less repetitive regions, which has 1.2 additional mismatches per 10kb from the optimal solutions (closest neighbors), and 57.3% alleles had no mismatches, and 77.0% were mapped to the optimal solution (Fig. 3d). The leave-one-out results were 96.5% closer to the original PAs compared to the closest GRCh38 gene at 79.3 mismatches per 10kb.

To isolate sources of errors in cases of misassembled duplications, we directly compared leave-one-out genotyping results to a telomere-to-telomere phased assembly, filtering out intronic/decoy sequences. The sample genotypes had 11,627 correctly matched allele-types, 599 (4.8%) mistyped to other allele-types, 131 out-of-reference (1.1%), 127 false-positive (0.5% F-1), 93 false-negative (0.4% F-1) for a total F-1 error of 6.7% (Methods) (Fig. 3e), showing most errors are not copy number errors with a 3% increase in mistyped on this genome compared to trio discordance.

The average runtime for genotyping at 30x coverage was 80.2 minutes (1.0 min/1×coverage for sample preprocessing, and 0.9 s/gene for genotyping) on a single core (Fig. 3f), indicating that ctyper is suitable for biobank analysis.

We compared benchmarking results on *HLA*, *KIR*, and *CYP2D6* to the locus-specific methods T1K^44^ and Aldy^45^. For 31 *HLA* genes, ctyper reached 97.7% accuracy of predicting all four fields of *HLA* nomenclature^32,46^ against the full-set and 86.0% among the leave-one-out, while T1K had 46.5%. Regarding protein-coding products, ctyper reached 99.8% accuracy against the full-set and 96.3% among the leave-one-out, while T1K had 84.7% (Fig. 3g). For 14 *KIRs*, ctyper reached 98.8% accuracy of predicting full three fields against the full-set and 68.0% among leave-one-out. Regarding protein-coding products, ctyper reached 99.5% against the full-set and 86.1% among leave-one-out (Supplementary Figure 4). Benchmarking *CYP2D6* star annotation based on assemblies^47^, ctyper reached 100.0% against the full-set and 83.2% among leave-one-out, compared to 80.0% accuracy using Aldy (Fig. 3h). The SNP variants inferred by ctyper genotypes had a 100.0% F1-score against the full-set and 95.7% among leave-one-out, compared to 85.2% using Aldy.

Finally, we used ctyper to genotype 273 challenging medically relevant genes^48^, 62 of which show CNV. Unrepetitive (unmasked) regions had 0.29 mismatches per 10kb against the full-set, 99.7% closer to the reference genome, and 4.9 mismatches per 10kb against leave-one-out, 94.8% closer to the reference genome (Supplementary Figures 5–7). Including masked regions, there were 10.5 mismatches per 10kb against the full-set, and 74.7 mismatches per 10kb among leave-one-out (Supplementary Figures 8–10).

### Sequence level diversity of CNVs in global populations

**Figure 4.**
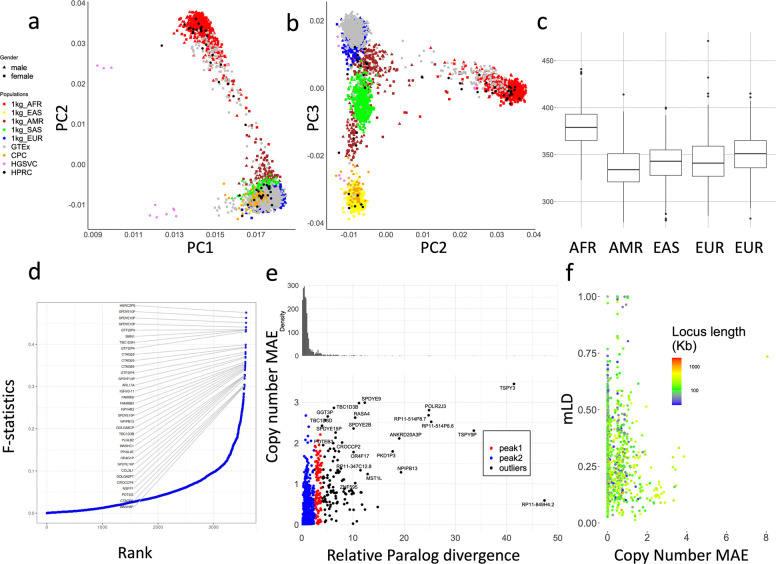
**a,b**. PCA of allele-specific copy numbers on genotype results and assembly annotations. **c,** Distribution of total autosomal gene copy numbers among 2504 unrelated 1kgp samples. **d,** Population differentiation measured by F-statistics of allele-types among different continental populations. The genes with an allele-type with an F-statistic more than 0.3 are labeled. **e,** Copy number and relative paralog divergence. Based on our genotyping results on 2504 unrelated 1kgp, for genes found to be CNV to the population median in more than 20 samples, we determined the average aggregate copy number difference (MAE) between individuals and estimated the average paralog differences relative to orthologs difference. **f,** Multi-allelic linkage disequilibrium between pairs of CNV genes less than 100kb apart. The largest MAE value of each pair is used for the x-axis values. The total locus length denotes the length from the beginning of the first gene to the end of the last gene.

We used principal component analysis (PCA) to examine the population structure of PA genotypes on 2,504 unrelated 1kgp samples, 879 Genotype-Tissue Expression (GETx) samples, and 114 diploid assemblies (Figs. 4a,b) after filtering low frequency (<0.05) allele-types and limiting copy numbers to 10. The 1kgp, GETx and genome assemblies were clustered by population as opposed to data source, suggesting little bias between genotyping and assembly, or across cohorts. The HGSVC assemblies are outliers on PC1, possibly due to assembly quality.

The top 0.1% highest weighted allele-types on PC1 have an average aggregate copy number (aggreCN) variance of 26.33, compared to an overall of 4.00 (p-value=1.11e-16, F-test). Similarly, PC2 and PC3 have mean aggreCN variance of 19.73 and 7.20, suggesting CNVs are weakly associated with sequence variants. Furthermore, PC1 is the only PC that clustered all samples into the same sign with a geographic center away from 0, suggesting it corresponds to modulus variance (hence aggreCN) if treating samples as vectors of paCNVs. Meanwhile, PC2 and PC3 are similar to the PCA plots based on SNP data on global samples^49^, suggesting they are associated with the sequence diversity on CNV genes. The total number of duplications are elevated in African populations (Fig. 4c), reflected in the order of PC1 (Fig 4a).

We next used the F-statistic that is similar to the F_st_ but accommodates more than two genotypes (Methods) to test the differences in distributions across five continental populations (Fig. 4d). In total, 4.4% (223/5,065) of duplicated allele-types showed population specificity (F-statistic > 0.2, Supplementary Table 5). The allele-type with the highest F-statistic (0.48) contains duplications of the *HERC2P9* gene that is known to have population differentiation^9,50^. Another example is a converted copy of *SMN2* annotated as a duplication of *SMN1* that is enriched in African populations (F-statistic=0.43).

We then measured whether duplicated genes were similar or diverged from reference copies, indicating recent or ancient duplications, and providing a measure on reference bias from missing paralogs. We constructed multiple sequence alignments (Methods) for each gene group, and measured the pairwise differences at non-repetitive sequences. We determined the average paralog divergence relative to ortholog divergence (Methods), which we refer to as relative paralog divergence (RPD). We also measured diversity by the mean absolute error (MAE) of the gene copy number in the populations (Fig. 4e). Based on RPD, using Density-Based Spatial Clustering of Applications with Noise^51^, we identified two peaks at 0.71 and 3.2, with MAE centers at 0.18 and 0.93. The first peak indicates genes with rare and recent CNVs, while the second peak indicates more divergent and common CNVs, often CNVs that may be inherited as different structural haplotypes. For example, *AMY1A* has a high RPD at 3.10 because of the truncated duplications of *AMY1A* (blue gene annotations in Fig. 2a). These results are consistent with ancient bursts of duplications in humans and primate ancestors^52^.

We next studied haplotype linkage of PAs to investigate the levels of recombination at different loci. We determined multi-allelic linkage disequilibrium (mLDs) between PAs using the 1kg genotypes^53^ (Methods) for 989 allele-types that were adjacent and less than 100kb apart on GRCh38 (Fig. 4f), and found the average within each gene-group. Among all mLDs, there was a strong negative rank correlation between MAEs of the copy number and mLD (***ρ***=−0.24, p-value=3.4e-15, Spearman’s rank), which is stronger than the rank correlation between MAEs of gene copy number and total locus length (***ρ***=−0.21, p-value = 1.5e-11), suggesting a reduced haplotype linkage on genes with frequent CNVs. The lowest mLD=0.013 found on *FAM90*, a gene with frequent duplications and rearrangements^54^. Not surprisingly, the 29 highest loci (mLDs > 0.7) are enriched in the sex chromosomes (N=19). Furthermore, *HLA-B* and *HLA-DRB*, had mLD >0.7 and only copy-number variation by deletion. The *HLA-DRB* deletions were only apparent after correcting HLA-specific coverage bias (Supplementary Methods). The *amylase* locus has a value of 0.293 due to recombination (Fig. 1a).

### Expression quantitative trait locus (eQTLs) on pangenome alleles

To investigate the expression impact of paCNVs, we performed eQTL analysis in the Geuvadis^55^ and the GTEx^56^ cohorts. There were 4,512 genes that could be uniquely mapped in RNA-seq alignments, and 44 without unique sequences such as *SMN1/2* and *AMY1A/1B/1C* (Methods, Supplementary Table 6), for which expression was pooled among indistinguishable copies for eQTL analysis. Genes after pooling together each of those with unique regions are called gene-units.

**Figure 5.**
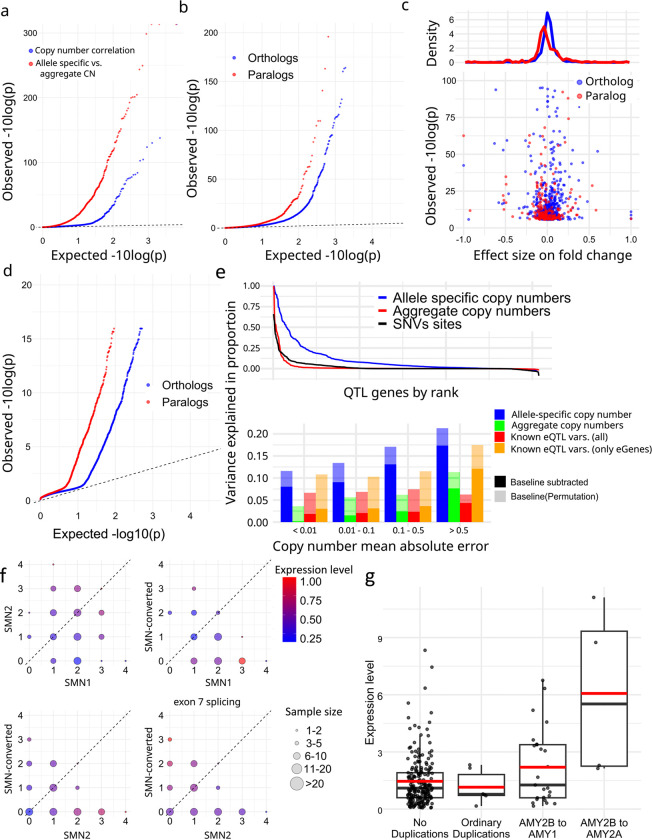
**a.** Q-Q plot of association of aggregate (*blue*) and allele-specific (*red*) copy numbers to gene expression in Geuvadis samples. **b,** Comparative gene expression of orthologs (*blue*) and paralogs (*red*). **c,** Fold change effect size of all alternative expressions. For all allele-types found to be significant, the fold changes as well as p-values were shown. **d,** Preferential tissue expression of orthologs and paralogs. **e,** (top), Model evaluation for PAs representing gene expression diversities. (bottom) Quantification of variance explained by different representations of genomic diversity: full paCNV genotypes, aggregate copy number, and known eQTLs variants. **f,** Case study on *SMN* genes showing decreased gene expression on converted *SMN*. The average corrected expression level in Geuvadis samples is shown under different copy numbers of *SMN1*, *SMN2*, and converted *SMN*. Transcript levels are the total coverage of all isoforms, and exon 7 splicing level is measured by counting isoforms with a valid exon 7 splicing junction. **g,** Case study on amylase genes showing increased gene expression on translocated *AMY2B*.

We corrected expression bias using PEER^57^ with the first three PCs from reported genotypes^58^, and performed association analyses with paCNVs. After merging paCNs to aggreCNs, 5.5% (178/3,224) of gene-units showed significance (corrected-p = 1.6e-0.5, Pearson-correlation) as previously observed^35^. We then tested whether using paCNVs would provide a stronger fit by updating the aggreCNs with individual paCNVs and performing multivariable linear regression on expression (Methods). There were significant improvements in fitting for 890 gene-units (27.6%) (corrected p=1.6e-05, one-tailed F-test) (Fig. 5a).

The improved fit could be explained by non-uniform effects on expression of alleles in the same gene-unit. To test this, we used a linear mixed model (LMM, Methods)^59,60^ to regress total expression to individual allele-types and estimate allele-specific expression, then compared these values to peers (Supplementary Table 7). For allele-types within solvable matrices with >10 samples, we found that 7.94% (150/1,890) paralogs and 3.28% of (546/16,628) orthologs had significantly different expression levels (corrected with sample size = number of paralogs + orthologs, corrected-p = 2.7e-06, Chi-squared test, Fig. 5b). Overall, paralogs are found to have reduced expression (Fig. 5c), consistent with previous findings on duplicated genes^61^.

We compared across 57 tissues in the GTEx samples to see if allele-types had different most-expressed tissues than their peers using LMMs to estimate the expression levels on each tissue (Methods, Supplementary Table 8). There was alternative tissue specificity for 132 of 2,820 paralogs (4.7%) and 225 of 19,197 orthologs (1.2%) (corrected-p = 6.4e-08, union of two Chi-squared tests, Methods, Fig. 5d).

Additionally, we used analysis of variance (ANOVA) to estimate the proportion of expression variance explained by paCNVs using Geuvadis, and compared it to a model based on known SNPs, indel, and SV eQTL variants^62^ (Methods). As expected, the highly granular paCNVs explain the most variance: on average, 10.3% (14.3% including baseline). In contrast, 58.0% of gene-units are eGenes with known eQTL variants that explained valid variance by 2.14% (1.60% considering experimental noise, in agreement with a previous estimate of 1.97%^63^). On average, 1.98% of the variance was explained by aggreCNs, and 8.58% by allele-type information. When combining both paCNVs and known eQTL sites, 10.4% (19.0% including baseline) of the valid variance was explained (Fig. 5e).

We examined *SMN* and *AMY2B* genes as case studies due to their importance in disease and evolution^34,64^. The *SMN* genes were classified into three categories: *SMN1*, *SMN2*, and *SMN-converted*. We estimated the total expressions of all transcripts and the expressions of only isoforms with valid exon 7 splicing junctions. For total expression, no significant difference was found between *SMN1* and *SMN2* (0.281 ± 0.008 vs 0.309 ± 0.009, p=0.078, Chi-squared test). However, significant differences were found between *SMN-converted* and *SMN1/2* (0.226 ± 0.012 vs 0.294 ± 0.002, p=1.75e-07, Chi-squared test), with a 23.0% reduction in expression of *SMN-converted*. In contrast, despite with lower overall expression, *SMN-converted* had 5.93× the expression of *SMN2* (p=2.2e-16, Chi-square test) regarding valid exon 7 splicing, indicating while *SMN-converted* has full functional splicing^65^, its overall expression level is lower (Fig. 5f).

For *AMY2B*, we studied the expressions of duplications when they are translocated to proximal to other *AMY* genes, such as the PAs containing *AMY1* and *AMY2B* at figure 2a. Using GTEx pancreas data, we estimated their expressions as well as other duplications. We found that these translocated *AMY2B* genes had significantly higher expression than other duplications (1.384 ± 0.233 vs −0.275 ± 0.183, p=7.87e-09, Chi-squared test) (Fig. 5g).

## Discussion

New pangenomes present both opportunities and challenges for the study of complex genetic variation: while they reveal the landscape of complex variation, it is challenging to use these sequences to analyze biobank (NGS) cohorts. To enable this, we developed an approach to divide assemblies into pangenome-alleles: sequences that are copy number variable and inherited with low disequilibrium in gene families, and to genotype their copy number in NGS samples.

The use of ctyper genotypes increases the scope of studies on CNVs to include sequence variation between copies. For example, our finding that CNVs reflect two modes of variation: high-identity (and likely recent), and low-identity (ancient and polymorphic) duplications, is based on large cohort ctyper genotypes rather than assembly annotations. As another example, the ctyper genotypes yield tissue-specific expression of paralogs as well as relative contributions to expression of different forms of duplications such as *SMN*.

We investigated the significant improvement of the ANOVA on PAs, whose genotypes reflect underlying sequences with multiple linked variants from known eQTL variants that are bi-allelic single variants. In contrast to PAs, there were either very few or very many eQTLs variants per gene, indicating LD (Supplementary Figure 3) as addressed by fine-mapping^66^, and increasing multiple testing burden^67^. Additionally, there was a greater proportion of variance explained among genes with more CNVs by eQTL variants, possibly explained by indirect association by LD (for example the *HPR* genes, Supplementary Figure 1). Furthermore, as the frequency of CNVs increase, the explained variance by eQTL variants increases (t= 3.80, p-value = 1.6e-04), while the number of eQTL variants decreases (t = −4.79, p-value = 2.1e-06), suggesting that larger effects like CNVs might overshadow the discovery of other variants not in LD. Furthermore, gene expression might not be a linear additive effect of all variants^68^. For example, although *SMN*-converted contains variants that are either from *SMN1* or *SMN2*, its overall expression is lower than both. In this manner, the concept of PAs may have a wider potential for future genome-wide association analysis (including non-CNV genes).

Due to limited sample size, our associations are based on allele-types rather than individual PAs. Different cohort sizes may require different levels of granularity when defining allele-types. For example, the three subtypes of *SMN-converted* showed little difference in expression. Our current classification on allele-types was designed for biobank cohorts, so smaller cohorts may need to test on allele-types that aggregate more PAs. The granularity of genotyping is additionally defined by the length of PA sequences; genotypes using shorter PAs will more accurately reflect NGS samples, while longer sequences can preserve larger phasing and may be preferable in regions with low recombination such as *HLA-DRB*.

Ctyper also has limitations. First, while it is possible to detect CNVs smaller than PA units using ctyper (Supplementary Methods), full support requires additional benchmarking data. Second, ctyper currently does not provide confidence values for genotypes. Finally, although the visualization tool we provide might help in, the high-dimensionality PAs does increase the complexity of interpretation and association analysis.

As new high-quality references become available, we anticipate ctyper to be a useful method for interpreting the association between sequence-resolved CNV and traits at scale.

## Online Methods

### Constructing pangenome allele database

We initiated our study by identifying gene duplicates in pangenome assemblies. Our pangenome cohort was composed of assemblies from the Human Pangenome Reference Consortium (HPRC) (N=92, excluding HG02080 due abundant flagged regions), the Chinese-Pangenome Consortium (CPC) (N=114), the Human Genome Structural Variation Consortium (HGSVC) (N=18, only Pacbio HiFI assemblies were used), two telomere to telomere diploid assemblies (N=4), and reference genomes (GRCh38 including alternative loci and CHM13 T2Tv1). The gene database used for annotation was GENCODE v39 based on the GRCh38 reference genome.

The initial application of this study was on 3,203 genes known to have copy number variation detected by the HPRC and CPC studies.

We organized genes into gene ‘query sets’ where each query set encompassed genes with functional or similar sequence including pseudogenes and genes with distant homologies within the same gene family. The query sets were initially defined based on genes with shared name prefixes, and were used to locate copies of duplicated genes within the pangenome.

Direct sequence alignments might overlook sequences such as small pseudogenes and diverged paralogs, potentially creating biases in our genotyping. To address this, we developed a more sensitive alignment scheme to detect all copies of genes in the pangenome. For each query set, we used low-copy *k*-mers (*k* = 31) that appeared fewer than 255 times in the CHM13 genome, derived from all initial reference genes, to help locate similar genes. We searched for these *k*-mers in each of the pangenome assemblies and references. We then identified *k*-mer hotspots defined as maximal intervals of mapped *k-mers* containing more than 200 *k*-mers within any 1,000-base window within the interval. To aid in mapping small and fragmented pseudogenes, we included an additional criterion to define hotspots: the presence of 50 exonic *k*-mers within the same interval search.

Subsequently, we used BLASTn^1^ to refine the boundaries of each hotspot by aligning all reference genes in this query set to each *k*-mer hotspot extended by 10kb flanking sequences.

The *k*-mer defined hotspots include both individual loci mapped by multiple genes from a query set as well as loci with tandemly duplicated genes multi-mapped by individual genes in a query set. To account for this redundancy, we merged alignments that were less than 10,000 bases apart, causing tandemly duplicated genes to be merged into single loci. To avoid genotyped loci that may be split by recombination, if an intron exceeded 20,000 bases, we divided the locus at the midpoint of the introns. To ensure the overall sequence size was comparable, flanking sequences both upstream and downstream were adjusted to achieve a total length of 15,000 bases. These methods aimed to standardize the size of each sequence to be roughly 30,000 bases, approximating the size of linkage disequilibrium (LD) blocks. The collection of all sequences mapped by a query set are referred to as initial gene-groups.

### Definition of gene-groups and *k-mer* list

Because the initial gene-groups were defined from aligned query sets that potentially arbitrarily grouped genes with unrelated sequences based on name, we used subsequent steps of refinement to exclude unrelated sequences.

Initially, for each genome we extracted all *k*-mers exclusive to aligned locations of the initial gene-groups (hence not found elsewhere in the genome). We also filtered out repetitive *k*-mers with more than two-thirds of the *2*-mers and *3*-mers were redundant, as these were mostly associated with highly repetitive DNA, such as Variable Number Tandem Repeats (VNTRs), microsatellites, and transposable elements. Additionally, we excluded *k*-mers demonstrating a high (>70%) or low (<30%) GC content bias^2^.

Subsequently, we filtered sequences predominantly composed of the *k*-mers removed in the previous step. The remaining sequences were then categorized into subgroups based on the number of shared *k*-mers. This classification was achieved using graph partitioning. Each sequence was represented as a node, and edges were made between node pairs sharing an excess of 500 unique *k*-mers, except for *NBPF* and *ANKRD* genes, for which a higher threshold of 2,000 unique *k*-mers was set to further reduce the sizes of partitions for computational efficiency in later analysis. Each partition represents a singular gene-group, and the list of unique *k*-mers specific to each gene-group was compiled and termed as ‘*k*-mer list’.

As an additional filtration, we filtered out genes from the non-confident regions reported by the HPRC, as well as truncated genes from small scaffolds. The genes included needed to be at least 10,000 base pairs away from both ends of a scaffold, except for sequences from genes taken from the reference genomes located at the telomeres.

### *k*-mers based phylogenetic tree construction

We constructed phylogenetic trees for each gene group based on their *k*-mer composition. Initially, for every gene group, we assembled a *k*-mer matrix, M, that encapsulates all sequences in the gene group. Within this matrix, individual rows represent distinct gene sequences, while each column corresponds to a unique *k*-mer from the *k*-mer list exclusive to the gene group. The matrix cell values are the counts of each *k*-mer present in the respective gene sequence, which is mostly 0 or 1, but occasionally more than 1 when there are low-copy repeated sequences in the gene, or the row represents a tandemly duplicated locus.

The matrix M allows us to measure the concordance between any two sequences, G_i_ and G_j_, by calculating their inner product, denoted as <G_i_ * G_j_> . Consequently, the norm matrix, N = M * M^T^, reflects the *k*-mer concordances for all sequence pairs within the gene group.

We constructed a similarity matrix, S, where S_i,j_ is the cosine similarity of G_i_ and G_j_ representing the sequences. The cosine similarity for any two sequences, G_i_ and G_j_ can be obtained by normalizing the norm matrix N according to the squares of *k*-mer vectors (approximately equal to sequence lengths) of the sequences in question.

Finally, we used the Unweighted Pair Group Method with Arithmetic Mean (UPGMA) algorithm on the similarity matrix S to generate the phylogenetic tree for each gene group.

### Clustering of pangenome alleles into alle-types

We used phylogenetic trees for the annotation and classification of closely related groups of alleles, which we term ‘pangenome allele-types’. The classification of pangenome allele-types is guided by two primary criteria applied across all allele-types:

Homogeneity within allele-types: A allele-type must exhibit near-identical characteristics amongst its members, which is quantified by ensuring the largest *k*-mer distance between any two members does not exceed 155 *k*-mers, which is roughly equivalent to the variation caused by 5 single nucleotide polymorphisms or a structural variation of approximately 95bp, such that allele-types are capable of representing most common variants in about 30kb range.
Distinctiveness of allele-types: Each allele-type must be distinct from its neighboring allele-types. This is measured using a *k*-mer F-statistic score, which must exceed 2 when compared with adjacent allele-types. In cases where allele-types are composed of fewer than three members, the F-statistic may not be reliable; hence, we default this score to 0 for such small allele-types, but change the cutoff of the former criteria to 155 * 3 to detect singleton rare events.

Employing a ‘bottom-up’ recursive approach starting from leaves, we applied these criteria to all allele-types, aiming to identify and report the largest possible near-identical allele-types. These are later used to identify equivalent loci after genotyping.

### Pangenome allele annotation relative to the reference genome

We annotate CNVs events and duplicated alleles in the pangenome assemblies in relative the GRCh38 genome. This requires us to find out the corresponding GRCh38 gene for each pangenome allele. However, this is a known challenging problem of orthology assignment^3^.

First, PAs often align to multiple paralogs on GRCh38, and the gene overlap with their liftover locations may not be the most similar reference gene due to gene conversion and translocation (Figs. 1f and 2a). To address this problem, we designed a method to match PAs to their closest GRCh38 genes based on *k*-mer similarity. For every haplotype, we obtained all pairwise similarities between each of its PAs to each of GRCh38 PAs. We matched PAs to their most similar GRCh38 PAs, starting from the most similar pair, until all PAs were matched or failed to match (had no reference gene with >90% similarity). Secondary redundant matches (match to reference genes that had already been matched) were annotated as duplications (distal).

Second, the former failed to match PAs are likely alleles with large SVs, such as insertion, deletion and local proximal duplications. We attempted to lift them back to GRCh38 using their flanking sequences (100kb either side). Because it is challenging to directly liftover genes in the regions with large segmental duplications, we designed this liftover to be a two-stepped liftover. First, we lifted PAs to the region with the best local alignment coverage, allowing SVs to break alignments into smaller units. Second, we performed a global pairwise alignment between PAs and the lifted region to locate the best aligned gene with the presence of local translocations and tandem duplications (Supplementary Methods).

Third, to annotate the proximal duplications mentioned in the last step as well as to annotate diverged paralogs that failed to match from both prior methods, we annotated PAs regarding the gene transcripts. We aligned all exons from the same gene group to PAs, and based on the exon orders and alignment scores, and determined the optimal combinations of transcripts on each PA (Supplementary Methods). The PAs containing no exons were annotated as introns and PAs containing only transcripts of other non-interested genes were annotated as decoys. Introns and decoys were usually filtered out from analysis and the rest PAs are considered as valid alleles, including pseudogenes that have no intact protein-coding transcripts and putative protein-coding genes with intact protein-coding transcripts.

It is important to note that, because proximal duplication may be highly associated in inheritance and potentially interference with each other functionally such as co-expression (which found between *HP* vs *HPR*, Supplementary Figure 1), and exonic expansion can be found in gene *LPA* and *NBPF,* we treated PAs with proximal duplications as a new type of a single PA, instead of treating them as multiple independent copies of singletons.

### Definition of orthologs and paralogs in the pangenome

Based on annotation results, to illustrate the relation of PAs to their corresponding reference genes regarding orthology and sequence similarities, we classified PAs into four categories, including two types of orthologs and two types of paralogs:

1. Reference alleles are alleles in the same allele-type with GRCh38 alleles, representing the alleles almost identical to the reference sequences.
2. Alternative alleles are orthologs located at the same genomic locus as the reference gene but are distinctly in different allele-types from GRCh38 alleles, including alleles that have a list of small variants in strong linkages or alleles that have large structural variations, such as proximal gene/exon duplications or deletions, as observed in genes like *HPR*, *NBPF*, and the *CYP2D6* (star-alleles) gene.
3. Duplicated paralogs (alleles) consisting of paralogs that have been duplicated to different loci from the reference alleles. Despite being translocated, they retain similarities (>80% in *k*-mers) to the reference alleles. These alleles often reflect large, recent segmental duplications in the genome, including similar paralogs, such as *AMY1A*, *AMY1B*, and *AMY1C*, which are still often considered as the same gene despite their distinct locations.
4. Diverged paralogs (alleles) not only differ in their translocation status but also have sequences that are significantly divergent (<80% in *k*-mers) from reference alleles, such that cannot be simply assigned to a single reference gene. These are typically characterized by highly diverse non-reference paralogs, incomplete gene duplications, and novel processed pseudogenes. An illustrative example of diverged paralogs is found among amylase genes, which indicates a proximal translocation event between *AMY1* and *AMY2B* genes.

### Genotyping NGS sample with ctyper

The goal of ctyper is to select a list of pangenome alleles and determine their individual copy numbers to represent the CNVs of unknown NGS samples. Instead of sequence alignment, our genotyping is based on *k*-mer comparison, which is not only more efficient but also not affected by misalignments that are frequent in the genomic regions enriched in structural variation and repetitive elements. Another advantage is that there is little bias in *k*-mers between high quality long-reads and NGS data^4^, so the *k*-mer data based on assemblies can be applied to predicting NGS data.

The genotyping proceeds per-gene. Given an NGS sample and a *k*-mer matrix M derived from pangenome allele annotation, we generate a vector V for an NGS dataset that includes the counts of each *k*-mer found in the matrix for the NGS sample normalized by the sequencing coverage. We seek to find a vector X of copy-number of each pangenome allele that minimizes the squared distance to the *k*-mer counts we observed in NGS data, e.g. argmin_x_ (‖ M^T^ * X - V ‖). Compared with absolute distance, squared distance is more suitable for the normal-like noise in NGS data^5,6^. Although it is possible to directly obtain an integer solution using mixed-integer linear programing (MILP), this is NP-hard^7^ and can only be used with very few variants/*k*-mers^8,9^. This restricts the use of MILP on the pangenome. The relaxed non-integer solution has an analytic solution which can be efficiently solved for. In essence, the computational problem is akin to a multivariable linear regression. The non-negative least error (NNLS) solution can be further obtained via Lawson-Hanson algorithm^10^.

To make the solution closer to the maximum likelihood estimation, during the regression, we rescaled dimensions of *k*-mers to even their expected uncertainty. Assuming the observation of *k*-mer copy number follows negative binomial distribution with the dispersion small enough to be distinct from Poisson^6^, the expected variance is roughly proportional to the square of observation, thus we weighted the *k*-mer to the square of the reciprocal of their observed copy number. We also applied smaller weights (adjust=0.05) on singleton *k*-mers (observed in only one PAs and not observed in NGS as well) because they are more likely to be sequencing errors.

The last step is referred to as reversed phylogenetic regression. Our analysis (Supplementary Methods) reveals two strong relationships between NNLS and the integer solutions under a phylogenetic relationship. First, the coefficient on each known allele is inversely proportional to its cosine vector distance to the unknown NGS allele. Hence, in a pangenome with diverse representation, the coefficients of NNLS will be mostly located on the genes that are very similar to the unknown gene. Second, when the coefficients are located on genes that are very similar to the unknown gene, the sum of total coefficients will be very close to the sum of its integer copy number.

Based on the solution’s high “convergence” on the phylogenetic tree, we designed a greedy algorithm to efficiently collect non-integer solutions and round it to integer solutions. This algorithm is iterative, employing a bottom-up approach from leaves to root. At each level of the hierarchy, we round the non-integer values to the integer solution with the least overall residual, and propagate the remainder to the next hierarchy. Because at each hierarchy, there are only two remainders from either branch of the tree, this solution is highly efficient.

### Trio analysis

Trio analysis is to determine if the genotype combinations of child-father-mother show possible Mendelian violations. When the copy number of a child is 0, the parents need to be 0 or 1; When the copy number of a child is 1, the parents can not both be 0 or both be 2; When the copy number of a child is 2, the parents both need to be 1 or 2. When the copy number of a child is more than 2, the parents need to have the sum to be greater or equal to this number.

### Leave-one-out comparison of genotyping results to pangenome assemblies

To find out the extent to which the genotyping results can represent the individual small variants on each PA, we aligned PAs in the original assemblies to their corresponding PAs in the genotyping results.

First, the original assembly PAs were one-to-one paired to genotyped PAs. This pairing was finished by a greedy method. We obtained all pairwise similarities in *k*-mer between each pair of the PAs across original assemblies and genotyping results. Starting from the most similar pair, we paired those alleles without replacement and iterated this until all original assemblies PAs are either paired or failed to be paired (has no genotyped PAs with >90% similarity).

Second, the paired PAs were then aligned using global pairwise alignment tool Stretcher^11^ for masked sequences and Locityper^12^ for unmasked sequences. From the global alignments, we obtained the number of mismatched bases in the unmasked region, where the low copy repeat *k*-mers are used in *k*-mer matrices.

### Classification of errors

We classified four types of errors for our benchmarking:

1. False positive: the genotyping results have an additional copy;
2. False negative: the genotyping results have a missing copy;
3. Miss typing: assign a copy to incorrect type;
4. Out of reference: the singleton type among the pangenome and lost reference duringleave-one-out.

### Benchmarking *HLA, KIR* and *CYP2D* genes with public nomenclatures

We benchmarked the results on *HLA* and *CYP2D* genes from all 39 HPRC samples with NGS data from both full-set and leave-one-out analysis. First, we labeled all IPD-IMGT and CYP2D-star annotations of PAs. For *HLA* and *KIR*, we annotated using Immuannot^13^, and for *CYP2D6*, we annotated using Pangu ^14^. Using those annotations, we converted genotyped PAs sequences into public nomenclatures and compared nomenclatures with the annotation results of the assemblies from the same samples. The benchmarking results of *HLA* were compared with T1K with its default settings and the benchmarking results of *CYP2D6* were compared with Aldy with its default settings.

We also benchmarked SNP calling on *CYP2D6*, and compared with Aldy, with its default settings. We took the phased results of Aldy and matched them to their corresponding original PAs. In a range of about 6 kb, where the variants could be found (first SNP reported at chr22:42126309, last SNP reported at chr22:42132374), Aldy genotyped the variants with an F1 score of 85.2%, and ctyper genotyped the variants with an F1 score of 95.7%.

### Total number of duplication events from genotyping results

Based on ctyper’s genotyping results, we calculated the total number of duplication events for each 1kgp sample, excluding 7 samples due to having extreme values different from the population mean by more than five standard deviations. The total number of each reference gene is measured in each genome and compared to GRCh38 chromosomes excluding alternate haplotypes. Each duplication event is called if the genome has more copy number than twice of GRCh38, excluding decoys/introns and sex chromosome genes. The total number of duplication events is reported for each genome. It is important to note that these duplications also included pseudogenes and small exonic fragments besides known protein-coding genes.

### Measuring F-statistic values

Because allele-types may have copy numbers beyond of two and may not be applicable to Fixation index (F_st_), we instead used F-statistic value to measure the population specificity of allele-types. The F-statistic value is based on the F-test, where we obtained the variances of copy numbers within all continental populations (within-group variance), and use it to divide the variances of copy numbers across different populations (between-group variance).

### Relative paralog divergence

Relative paralog divergence (RPD) measures the mean divergences of the paralogs to other alleles, in relative to the mean divergence between only orthologs. RPD was determined for each reference gene and based on the graphic multiple sequence alignments (gMSAs, Supplementary Methods) of PAs assigned to that reference gene as well as ctyper’s genotyping results.

First, the divergence value was determined for each pair of PAs assigned to the same reference gene. It was measured based on the alignment scores of unmasked bases (misalignment and gap open = −4, and gap extend = 0, normalized by total alignment length) from gMSAs.

Second, we obtained mean divergence of the orthologs by averaging divergence values between the two PAs from samples with CN = 2.

Third, we then determined the population median copy numbers for each reference gene, and divided samples into those with additional copy numbers (copy numbers more than the median) and those with no additional copy numbers (copy numbers not more than the median).

It is unreliable to directly distinguish the paralog from orthologs due to complex rearrangements (e.g. Figure 2a). To overcome this limitation and only obtain the divergence values from additional copies, we performed statistical estimations based on large populations. We first estimated the mean divergence values from samples with no additional copy numbers and used it as the unit baseline B. When the population median CN = Y, because there are Y(Y−1)/2 pairs, then the total baseline is B * Y(Y−1)/2, which will be subtracted from total divergence values of samples with duplications, and Y(Y−1)/2 will be subtracted from the total number of pairs (the denominator) as well.

After subtracting the total baseline, the mean paralog divergence value of the additional copies were determined for all samples with additional copy numbers. This mean paralog divergence was then normalized by mean divergence of the orthologs obtained in step two.

### Multi-allelic linkage disequilibrium

Multi-allelic linkage disequilibrium (mLDs) is an analytic continuation of SNP-based bi-allelic linkage disequilibrium to allow computing linkages between multiple genotypes on neighboring loci. When there are only two genotypes on both loci, mLDs equals LD value. When there are more than two genotypes, mLDs measures LDs between each pair of genotypes across different loci, and takes the weighted average of all pairs. This weight is the product of both allele frequencies of the pair.

### Defining gene-units

We represented each gene by the major transcripts from the MANE (Matched Annotation from NCBI and EMBL-EBI^15^) project. Second, individual exons were aligned. Transcripts were recursively clustered together if they overlapped with previously clustered transcripts with more than 98% overall similarity taking the average similarity of all aligned exons from the transcripts. We call these clusters as gene-units. Third, for each gene-unit, we identified all its exons and looked for unique exons that did not overlap with exons from other gene-units. Fourth, we used these unique exons to represent each gene-unit and filtered out gene-units that have no unique exons (2079 out of 2579 filtered genes were known pseudogenes). Lastly, we assigned PAs to each gene-unit if they contain any of the corresponding unique exons with at least 98% similarity.

### Expression correction

For individual tissue analysis, similar to the prior study^16^, we logistically corrected the raw TPMs using tool PEER together with the first three principal components obtained from reported genotypes in chr1^17^. For cross-tissue analysis we corrected raw TPMs using DESeq2^18^.

### Association between CNVs to gene expression

We first associated gene aggregate copy number to expression levels using Pearson correlation (linear-fitting). The p-values and residuals of this fit were recorded. To test if including allele-specific information would improve the correlation, we used the ctyper’s pangnome allele-specific copy numbers to replace the aggregate copy numbers to perform multi-variable linear regression using allele-specific copy numbers as dependent variables and gene expression level as independent variables. We compared the residuals of multi-variable linear regression with residuals from Pearson correlation using F-test, and one-tailed p-values of the reduced residual was reported. Both p-values were corrected by the number of gene-units tested (N=3,224).

### Linear mixed model

We performed linear mixed modeling to measure the individual expression of each allele-type. We used the total gene expression values as the vector of observed dependent variables, different allele-types as the vector of independent fixed variables and the copy numbers from ctyper genotyping results were used as their coefficient matrix. The effect sizes of fixed variables were then solved using ordinary least squares (OLS) regression.

### Alternative expression of allele-types

To determine whether an allele-type has an alternative expression level compared to other allele-types of the same gene, we merged all other allele-types assigned to the same reference gene into a single variable, separating from the allele-type currently being tested. Additionally, we included other factors, such as paralogs that might also influence total expression, as additional parameters to adjust for their potential interference. For allele-types within solvable matrices with more than 10 non-zero expressions, using a linear mixed model and the R lm function^19^, we regressed the expression values to all variables to get their effect sizes. We then compared the effect-size of currently tested allele-type and the effect-size of other allele-types of the same gene using Chi-squared distribution with the linearHypothesis tool^20^. This p-value was then corrected by the number of total allele-types tested (N=18,518).

### Across tissue expression comparison

In order to determine if an allele-type has an alternative most expression tissue compared to other allele-types of the same gene, we merged all other allele-types assigned to the same reference gene into a single variable, separating from the currently tested allele-type. Additionally, we included other factors, such as paralogs that might also influence total expression, as additional parameters to adjust for their potential interference. For allele-types within solvable matrices with more than 10 non-zero expressions, we performed linear mixed models to estimate the gene expression level of each allele-type within each of the 57 tissues in GTEx V8. The tissue with the highest expression level was recorded and compared to the tissue with the second highest expression using the Chi-squared test. We then compared the results between currently tested allele-type and all other allele-types of the same gene to see if they had the different highest expressed tissue. When the highest expressed tissues were different, we tested the p-value of either events happening by combining the p-values from both side as p-combined = p1 + p2 − p1 * p2. This p-value was then corrected by the number of allele-types tested on all 57 tissues (N=776,902).

### ANOVA (Analysis Of Variance) test on gene expression

We first measured the total expression variance for each eQTL gene-unit, filtering out units with per-sample variance less than 0.1 to exclude genes not sufficiently expressed in the Geuvadis cohort. We estimated experimental noise by measuring expression variance between different trials of the same individuals (mean = 10.5% of the total variance) and excluded gene-units with experimental noise exceeding 70% of the total variance, resulting in 639 total gene-units on expression. We applied the one-in-ten rule to restrict the number of variants tested to be not greater than 45 (10% of the sample-size) to avoid over-fitting. We filtered out 18 units involving more than 45 PAs; When there were more than 45 known eQTL variants, we used 45 variants with the lowest p-values. The valid expression variance was obtained by subtracting experimental noise from total expression variance. Using ANOVA, we estimated the explained valid variance and adjusted the results by subtracting a baseline, defined as the mean expression variance explained by permuting the orders of all samples (estimated by the mean of 100 trials). If there are no reported eQTL variants, a value of 0 is used for known eQTL variants.

For paCNV, we further investigated the part of variance explained by gene aggreCNs, applying ANOVA to a random matrix with aggreCN information, such that had randomly assigned allele-types, but with the total copy number equal to the original matrix. We subtracted the variance explained by this random matrix from the total explained variance to obtain the variance explained by allele-type information.

## Supplementary Material

Supplement 1

## Data Availability

Software: https://github.com/ChaissonLab/Ctyper. Allele database and annotations: https://doi.org/10.5281/zenodo.13381931. Benchmarking and analysis code: https://github.com/Walfred-MA/CNVAnalyze.
