# Genotyping sequence-resolved copy-number variations using pangenomes reveals paralog-specific global diversity and expression divergence of gene duplication

**DOI:** 10.1101/2024.08.11.607269

**Published:** 2025-02-20

**Authors:** Walfred Ma, Mark JP Chaisson

**Affiliations:** 1 Quantitative and Computational Biology, University of Southern California, CA, USA.; 2 Norris Comprehensive Cancer Center, University of Southern California, Los Angeles, California, USA

## Abstract

Copy-number variable (CNV) genes are important in evolution and disease, yet sequence variation in CNV genes remains a blindspot in large-scale studies. We present ctyper, a method that leverages pangenomes to produce allele-specific copy numbers with locally phased variants from NGS reads. Benchmarking on 3,351 CNV genes including *HLA*, *SMN*, and *CYP2D6*, and 212 challenging medically-relevant (CMR) genes poorly mapped by NGS, ctyper captures 96.5% of phased variants with ≥99.1% correctness of copy number on CNV genes and 94.8% of phased variants on CMR genes. Applying alignment-free algorithms, ctyper takes only 1.5 hours to genotype a genome on a single CPU. Its results improve predictions of gene expression compared to known eQTL variants. Allele-specific expression quantified divergent expression on 7.94% of paralogs and tissue-specific biases on 4.68% of paralogs. We found reduced expression of *SMN*-2 due to *SMN1*-conversions, potentially affecting spinal muscular atrophy, and increased expression of translocated duplications of *AMY2B*. Overall, ctyper enables biobank-scale genotyping of CNV and CMR genes.

## Introduction

Human genomes are characterized by frequent duplications and deletions, leading to copy number variation (CNV). Up to 10% of protein-coding genes are known to be copy-number variable, showing distinct distributions across human populations^1,2^ and association with traits such as body mass index^3^ and disease including cancer^4^, cardiovascular diseases^5^, and neurodevelopmental disorders^6,7^. While CNVs are infrequent genome-wide, regions of long, low-copy repeats called segmental duplications (SDs) are enriched in genes and are catalysts for recurrent CNVs^8,9^. This leads to diverse gene families such as *TBC1D3*, *NPIP*, and *NBPF*^10,11^. The mechanisms contributing to CNVs, along with the elevated mutations in SDs^12^, result in variation not only in aggregate copy number (aggreCN) but also elevated sequence variation among the copies^12–14^. This variation can influence phenotypes and disease susceptibility^15–17^, including hypertension and type 2 diabetes^18^. In addition, many CNV genes in SDs are found to be human-specific, quickly evolving, and highly associated with brain function^19–21^.

There is scarce information about variation in non-reference gene duplicates, particularly in studies using short-read next-generation sequencing (NGS) data. Existing CNV calling tools detect excess coverage on reference alignments, hiding sequence variants among copies^22^. Furthermore, NGS alignment to a reference genome contains ambiguity and bias^23^. Advances in single-molecule sequencing enabled the assembly of pangenomes from diverse populations^24–26^. Almost all novel sequences found in the pangenome are CNV events^24^, providing sequence-resolved CNVs. Although reference bias may be reduced using graph pangenomes^27^, the variants distinguishing paralogs may be obscured during graph construction^28^. Furthermore, as pangenomes include more samples, diversity among populations, frequent gene conversion, and genome rearrangements present an even greater challenge^12^.

Here, we developed an approach to genotype sequence-resolved copy-number variation, providing copy-number maps with locally phased sequence variants in each copy. Our method, ctyper, uses alignment-free genotyping to call copy-number and allele-specific variants from NGS data leveraging a database of gene sequences derived from pangenome assemblies. This overcomes reference alignment bias and uncovers variation missed from single reference analysis. The efficiency of ctyper enables scaling of this analysis to biobank data.

## Results

### Overview of the genotyping method

We developed a method, ctyper, to genotype sequence-resolved copy-number variation in NGS data leveraging haplotype-resolved assemblies. Ctyper identifies whether an NGS sample shares a genomic segment with an assembly haplotype and determines the copy number of that segment in the sample.

We define these haplotype segments as pangenome alleles (PAs): sequence segments short enough to minimize disruption by recombination, allowing precise sharing with an NGS sample through sufficient haplotype homozygosity^29^ (the probability of two randomly selected haplotypes from a population being identical), yet long enough to capture structural information of genomic sequences. These segments capture genomic information in a phased form, representing structural variation (SVs), gene conversion events, and small variants. In this study, the boundaries of PAs are defined using gene annotations to include all consecutive and proximal exons (separated by <20 kb), with an additional 5 kb of upstream and downstream sequences added as anchors (Methods). The 20 kb cutoff reflects both biological proximities, as most transcription factors operate within this range, and population-level genomic linkage. The PAs typically range from 10 to 100 kb, corresponding to the scale of linkage disequilibrium (LD) blocks^30^, within which most SNPs are in strong linkage and can be treated as single units of inheritance. While PAs generally correspond to a single gene, they can also represent a fraction of a gene flanking long introns to reduce the interference of recombination or, conversely, include proximal duplicated paralogs within the 20 kb boundary.

**Figure 1.**
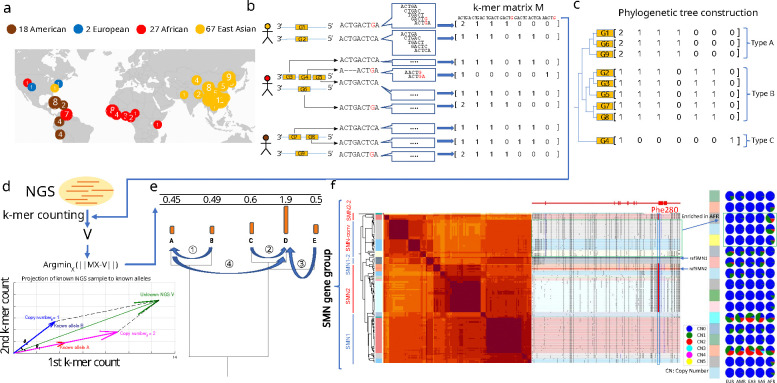
**a,** Demography of the reference pangenome assemblies, HPRC (46 diploid), CPC (57 diploid), HGSVC (9 diploid), T2T-YAO (1 diploid), and CN1(1 diploid), as well as GRCh38 and CHM13. **b,** Construction of pangenome *k*-mer matrices for CNV genes. Each individual gene is represented as a vector of counts of *k*-mers exclusively found among homologous sequences. All copies of genes including paralogs and orthologs are included and integrated as a *k*-mer matrix. **c,** Construction of phylogenetic trees based on *k*-mer matrices. **d,** Schematic of approach to estimate genotypes of alleles using NGS data. The *k*-mers from each matrix are counted in NGS data and normalized by sequencing depth. The normalized *k*-mer counts are projected to all pangenome genes. **e,** Reprojection to an integer solution based on the phylogenetic tree. **f,** An illustrative annotation and genotyping results on *SMN1/2* genes using HPRC samples. All *SMN* genes are categorized into five apparent types and 17 sub-subgroups. *SMN1*/*SMN2* correspond to the most common types of each paralog; *SMN1–2*, a copy of *SMN1* partially converted to *SMN2*; *SMN*-conv: additional converted SMN genes, mostly mapped to the *SMN2* locus, and is found to be enriched in African populations; SMN2–2: a rare outgroup of *SMN2*. The GRCh38 assembly includes *SMN1–2* and *SMN2*. On the right-side of the classification, the phylogenetic tree and heatmap of pairwise similarities are shown along with a mutant plot based on multiple sequence alignment highlighting point differences to *SMN1* in CHM13. Phe-280, the variant found to disrupt the splicing of *SMN2* transcripts is highlighted. Based on genotyping results in 1kg genomes, the pie charts give distributions of copy numbers within each continental population for each subgroup.

The genotyping of PAs avoids ambiguous alignments (i.e., alignments to multiple similar duplicative sequences in the genome) by using alignment-free genotyping, comparing the counts of a list of low-copy *k*-mers (DNA fragments of a fixed length, here *k*=31) of query NGS samples to known PAs. The *k*-mer counts of known pangenome-alleles are represented in matrices. A matrix is constructed for each gene of interest and includes orthologous genes, pseudogenes, and paralogs (Methods). Within each matrix, each row corresponds to a single PA, and columns contain the counts of each *k*-mer (Methods) (Fig. 1a,b).

When genotyping an NGS sample, ctyper first counts all *k*-mers from all matrices in the sample. It then identifies a combination of PAs as well as their allele-specific copy numbers such that their total corresponding *k*-mer counts are the least squared distance to observed *k*-mers counts from the sample. This is achieved by projecting the observed *k*-mer counts into the vector space of each *k*-mer matrix, and using phylogenetic-tree-based rounding to determine integer solutions of copy-numbers (Methods, Figs. 1c–e). The genotyping results are a list of PA-specific copy numbers (paCNVs). These paCNVs provide both the copy number information for each gene of interest and the most likely sequences for individual paralogs and orthologs of that gene.

As an example, the PAs for all *SMN*s (the genes associated with spinal muscular atrophy) contain 178 PAs, including copies of *SMN1* and *SMN2* as well as paralogs that have undergone gene conversion^31^ including genes found on the *SMN2* locus containing the *SMN1* phe-280, the SNP responsible for dysfunctional exon 7 splicing of *SMN2*^32^ (Fig. 1f).

### Pangenome-allele database construction and annotation

We focused on genes previously annotated as CNV^24,26^ among 114 diploid Pacbio-HiFi assemblies from the HPRC, HGSVC, CPC, and two haplotype-phased telomere-to-telomere assemblies^33,34^, and we also included GRCh38 and CHM13^35^. Overall, 3,351 CNV genes were annotated into PAs (Supplementary Table 1).

**Figure 2.**
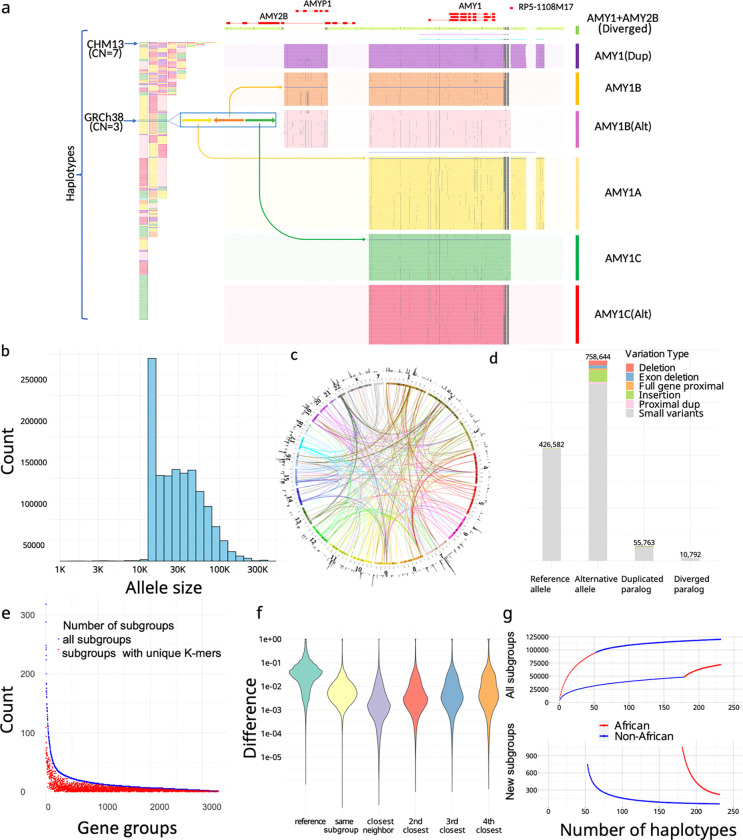
**a,** An overview of amylase 1 pangenome-alleles (PAs). (left) The corresponding order of all *AMY1* PAs on assemblies, which are colored based on their major subgroups (subgroups devoid of SVs >300 bps between members). (right) *AMY1* genes are extracted as PAs as well as their flanking genes and sequences, including an *AMY2B* translocated proximal to *AMY1*, and two pseudogenes: *AMYP1* and *RP5–1108M17*. All PAs are vertically ordered according to the phylogenetic tree and aligned via graphic multiple sequence alignments (gMSA, Supplementary Methods). Homologous sequences are vertically aligned. Mutations are visualized as dots, and large gaps (deletions) are visualized as spaces. Seven major subgroups are categorized including five paralogs and two orthologs. There are no pseudogenes around *AMY1C*, while *AMY1A* has *RP5–1108M17* nearby and *AMY1B* has *AMYP1* nearby. There are alternative versions of *AMY1B and AMY1C*, with sequence substitutions. A new paralog called *AMY1(Dup)* is found primarily on haplotypes with duplications, and has both pseudogenes nearby. The paralog of *AMY1* found with translocated *AMY2B* is called *AMY1+AMY2B*. There are also two rare paralogs (blue and violet) and one singleton ortholog (steel-blue). **b,** The size distribution of PAs on a log density. The minimum size of PAs is 15kb, though smaller alleles may be annotated on alternative haplotypes on GRCh38 and as partial loci when dividing large genes into alleles without recombination. **c**. CIRCOS plot of all PAs. (outer ring) The density of PAs in each megabase on GRCh38. (arcs) Interchromosomal PAs are included in the same groups. **d,** Annotation of PAs according to orthology and variants with respect to GRCh38. Duplicated paralogs are alleles with distal duplications and proximal duplications are included in alternative alleles due to potential interaction with original genes. **e,** Identifiability of highly-similar subgroups by unique *k*-mers. The total number of subgroups (blue), and the number of subgroups that may be identified by paralog-specific *k*-mers (red) are shown for each matrix with a size of at least three. **f,** The distribution of logistic pairwise distances of PAs depending on orthology and phylogenetic relationship. The values shown are average values from all genes. Small neighbor distances are an indicator of the strong representativeness of the current cohort. **g,** Saturation analysis for all subgroups using a recapture mode according to two sorted orders: African genomes considered first, and non-African genomes considered first.

We extracted PAs from all assemblies per gene family, such as amylase genes (Fig. 2a). In total, 1,408,209 PAs were defined and organized into 3,307 matrices. The average PA length was 33 ± 29 kb, and included protein-coding genes (69%), processed pseudogenes (20%), intronic duplications (5%), and decoys (7%). We annotated these PAs based on genomic locations as well as sequence variations (Figs. 2b–c).

Based on genomic locations lifted from references (GRCh38 and CHM13) (Supplementary Methods), PAs were classified as either reference-orthologs or paralogs. Among these, 6,673 PAs contained proximal duplications (duplications within 20 kb of the source gene, included in the same PA), of which 1,646 across 36 genes exhibited “runway duplication”^36^ with at least three duplications, such as the *HPR* locus (Supplementary Figure 1). These proximal tandem duplications were included in a single PA along with their original genes. The multi-gene PAs were considered reference orthologs if any gene copy in them maps to a reference PA. Including neighboring genes in a single unit can represent functional interactions such as co-expression, which we observed in *HP* and *HPR*, for example. Moreover, 164,237 PAs on 6,389 loci were classified as paralogous because they contained duplications on different loci from the source gene (>20kb away) and did not incorporate the source gene. Variation in PAs was annotated by aligning to their corresponding reference locus to identify variants relative to the reference genome (Supplementary Methods).

To reduce genotype dimensionality to accommodate cohort analysis when sample size is limited, we used a phylogenetic tree to merge highly similar neighboring PAs into highly-similar subgroups (Methods). In total, 89,236 highly-similar subgroups were defined and used as the “genotypes” of PAs, analogous to *HLA* nomenclatures (Supplementary Figure 2). Orthologs were classified into reference alleles, defined as alleles within the same highly-similar subgroups as the reference gene, and alternative alleles, which fall outside those subgroups.

We distinguished paralogous PAs that were structurally different from their corresponding reference sequences because they may potentially contain novel sequences recalcitrant to analysis using a single reference genome. Previously defined paralogous PAs that were similar (≥80% k-mer similarity) to their corresponding reference locus were labeled as duplicative paralogs, and remaining lower identity paralogous PAs were labeled as diverged paralogs. In total, 10,792 novel diverged paralogs from 2,734 subgroups were identified across 333 matrices (Fig. 2d). For example, some amylase PAs include paralogs for both *AMY1* and *AMY2B*, so they have low k-mer similarity with reference loci and were annotated as diverged paralogs (Fig. 2a).

### Pangenome alleles and highly-similar subgroups capture unique aspects of population diversity

To show pangenome alleles capture unique aspects of genomic information that cannot be replicated by other genomic representations, we compared the paCNVs to other representations of CNVs with lower resolution of variants: copy-numbers of reference genes^1,36^, single unique nucleotide *k*-mers^1,36,37^ (SUNKs), and large haplotype sequences^13,38–40^. Our analysis demonstrates that using PAs captures 94.7% of phased genetic variation in duplicated genes within the current cohort when compared to reference genes. Additionally, it highlights that nearby SUNK markers are poor proxies for PAs (Fig. 2e), with weak linkage to large haplotype structures (Methods).

Moreover, we evaluated the population diversity after merging PAs into highly similar subgroups. We found that even with largely reduced dimensions, highly-similar subgroups can still capture more than 80% of the total population variation. We also performed saturation analysis^41,42^ to estimate the percentage of the unknown highly-similar subgroups in each new genome and showed that the current cohort represents 98.7% highly-similar subgroups in non-Africans and 94.9% in Africans (Methods) (Fig. 2f).

### Genotyping Pangenome-alleles among NGS samples and benchmarking results

**Figure 3.**
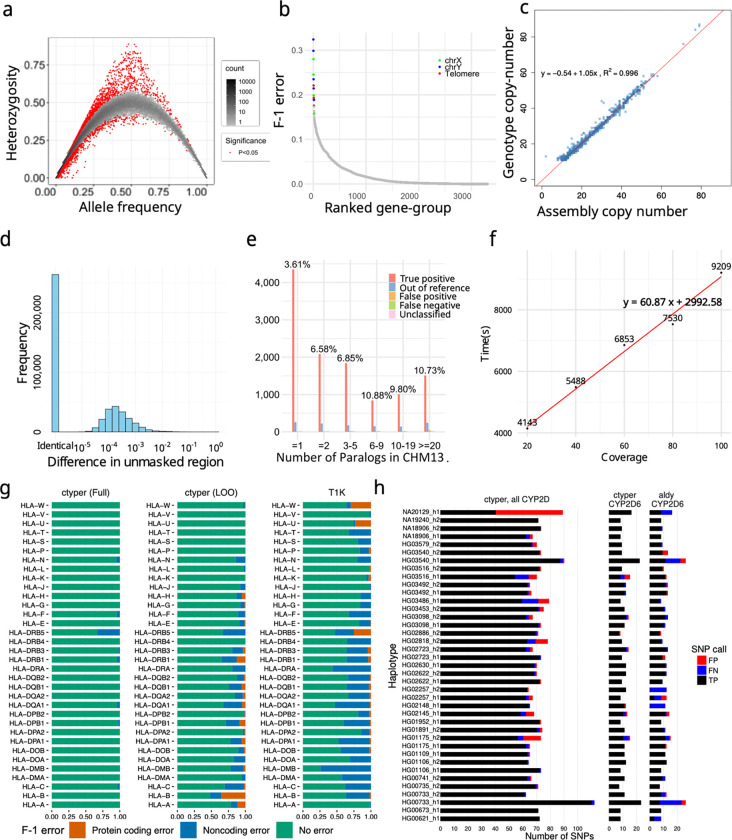
**a,** Hardy-Weinberg equilibrium of genotyping results on 1kgp unrelated samples. **b,** Genotype concordance of genotyping results on 1kgp trios, ordered by F-1 error. The matrices with F-1 error of more than 15% were labeled by genomic location. **c,** Copy number comparison between assemblies and genotyping results on 39 HPRC samples. **d,** Sequence differences between genotyped and original alleles during leave-one-out test using on Stretcher pairwise alignment of nonrepetitive sequences. **e,** Detailed leave-one-out comparison in the diploid T2T genome CN1. The results are categorized regarding the number of paralogs in CHM13 to show performances on different levels of genome complexity and the main sources of errors. **f** Runtime of ctyper on CN1 on all loci for varying coverage. **g,** Benchmarking of *HLA* genotyping using ctyper on full, and leave-one-out (LOO) databases, compared with T1K on 31 *HLA* genes. **h,** Benchmarking of *CYP2D* annotation on all *CYP2D* genes and *CYP2D6* exclusively.

We applied ctyper to genotype NGS samples within the 1000 Genomes Project (1kgp), including 2,504 unrelated individuals and 641 offspring. Accuracy was measured using Hardy-Weinberg Equilibrium (HWE), trio concordance (Supplementary Table 2), and comparison to reference assemblies, excluding intronic/decoy PAs (Methods). There were significant HWE violations (p < 0.05) for 0.75% of highly-similar subgroups after excluding sex chromosomes and setting the maximum copy number to two (Fig. 3a). There were 27 matrices having >15% highly-similar subgroups with significant disequilibrium, which were mostly small genes (median = 4,564 bp) with few unique *k*-mers (Supplementary Table 3). The average F-1 score for trio concordance was 97.58% (Fig. 3b), while 18 matrices had high discordance (>15%), primarily for subtelomeric genes or on sex chromosomes (Supplementary Table 4).

We assessed copy-number accuracy in the most challenging and highly duplicated gene families, such as amylase, *NBPF*, *NOTCH*, *GOLGA*, and *TBC1D3*, by comparing genotyped copy numbers of 39 HPRC samples against their corresponding assemblies. For each sample, we benchmarked on matrices from these gene families where the corresponding assembly had more than 10 PAs and no low-confidence sequences. The results showed a 0.2% false-negative rate and a 2.4% false-positive rate, with the higher false-positive rate likely explained by unassembled genes in the assemblies (Fig. 3c). In addition, including the remaining fewer CNV genes with matrices without filtered sequences, the copy number between genotype and assemblies remain highly correlated (***ρ***=0.996, Pearson correlation).

We then assessed how well the genotyped alleles reflect the sample assembly using 39 HPRC samples having both NGS and assemblies. Each sample was genotyped with the full database (full-set) or the database excluding its corresponding PAs (leave-one-out). We used a matching script to assign the genotyped PAs to the corresponding assembly (Methods), excluding intron/decoys and sequences with <1kb unrepetitive bases, and measured the similarity between the genotyped allele and assigned query using global alignment^43,44^. We performed a similar analysis treating the closest neighbor from the database to each assembly PA as the correct genotyped locus. Across samples, 2.9% of PAs from the leave-one-out assembly and 1.0% of PAs from full-set could not be paired, which is primarily due to mistyping, assembly error or copy number error. Using the full-set, paired PAs have 0.36 mismatches per 10kb, with 93.0% having no mismatches on less repetitive regions. The leave-one-out had 2.7 mismatches per 10kb on less repetitive regions, which has 1.2 additional mismatches per 10kb from the optimal solutions (closest neighbors), and 57.3% alleles had no mismatches, and 77.0% were mapped to the optimal solution (Fig. 3d). The leave-one-out results were 96.5% closer to the original PAs compared to the closest GRCh38 gene at 79.3 mismatches per 10kb.

To isolate sources of errors in cases of misassembled duplications, we directly compared leave-one-out genotyping results to a telomere-to-telomere phased assembly, filtering out intronic/decoy sequences. The sample genotypes had 11,627 correctly matched subgroups, 599 (4.8%) mistyped to other subgroups, 131 out-of-reference (1.1%), 127 false-positive (0.5% F-1), 93 false-negative (0.4% F-1) for a total F-1 error of 6.7% (Methods) (Fig. 3e), showing a 3% increase in mistypes on this genome compared to trio discordance and copy number agreement at 99.1% with even potential mis-assemblies.

The computational requirements are sufficient for biobank analysis. The average runtime for genotyping 3,307 genes at 30x coverage was 80.2 minutes (1.0 min/1✕coverage for sample preprocessing, and 0.9 s/gene for genotyping) on a single core (Fig. 3f) using ~20GB RAM, with support for parallel processing.

We compared benchmarking results on *HLA*, *KIR*, and *CYP2D6* to the locus-specific methods T1K^45^ and Aldy^46^, regarding their F-1 values. For 31 *HLA* genes, ctyper reached 98.9% accuracy in predicting all four fields of *HLA* nomenclature^47,48^ against the full-set and 86.3% among the leave-one-out, while T1K had 70.8%. Regarding protein-coding products (first two fields), ctyper reached 99.98% accuracy against the full-set (with 99.9% copy-number F1-correctness) and 96.5% (with 99.5% copy-number F1-correctness) among the leave-one-out, and T1K had 97.2% (Fig. 3g). For 14 *KIRs*, ctyper reached 98.5% accuracy of predicting full fields against the full-set and 70.6% among leave-one-out, while T1K only has 32.0% due to the limited database. Regarding protein-coding products (first three digits), ctyper reached 99.2% against the full-set (with 99.9% copy-number F1-correctness) and 88.8% among leave-one-out (with 99.2% copy-number F1-correctness), while T1K had 79.6% (Supplementary Figure 3). Benchmarking *CYP2D6* star annotation based on assemblies^49^, ctyper reached 100.0% against the full-set and 83.2% among leave-one-out, compared to 80.0% accuracy using Aldy (Fig. 3h). The SNP variants inferred by ctyper genotypes had a 100.0% F1-score against the full-set and 95.7% among leave-one-out, compared to 85.2% using Aldy. Genotyping results on *HLA* and *KIR* can be found in Supplementary Table 5 and detailed benchmarking on accuracies, specificity and copy number can be found in Supplementary Table 6.

We compared genotyping results on *HLA* to a contemporary developed method works on challenging genes also based on pangenomes, Locityper^44^. Locityper is slightly better at predicting all four fields, including non-coding variants (leave-one-out: Locityper 87.9% vs. ctyper 86.3%; full-set: Locityper 99.5% vs. ctyper 98.9%), while ctyper is slightly better on the first two fields of protein-coding products (leave-one-out: Locityper 94.0% vs. ctyper 96.5%; full-set: Locityper 99.5% vs. ctyper 99.98%). If only run on 28 *HLA* genes, using one thread, ctyper takes 35 seconds (1.1s for genotyping) on genotyping all *HLA*s. As a comparison, the Locityper genotyping of *HLA* genes required about 8.5 minutes using 8 threads (4 minutes spent genotyping). Separating out the genotyping runtime from read preprocessing (which may be amortized across multiple loci), for a 218✕ speedup in genotyping.

Finally, we used ctyper to genotype 273 challenging medically relevant genes^50^, including 61 genes included in the 3,307 CNV genes and 212 non-CNV CMR genes. Unrepetitive (unmasked) regions had 0.29 mismatches per 10kb against the full-set, 99.7% closer to the reference genome, and 4.9 mismatches per 10kb against leave-one-out, 94.8% closer to the reference genome (Supplementary Figures 4–6). Including repeat-masked low-complexity sequences (eg. VNTRs), there were 10.5 mismatches per 10kb against the full-set, and 74.7 mismatches per 10kb among leave-one-out (Supplementary Figures 7–9). Compared with Locityper in the full-set, ctyper has a lower average mismatch rate (0.011% vs. 0.015%) and works on more genes (273 vs 259), but Locityper has more exact matches (81.9% vs. 72.7%) (Supplementary Table 7 and Supplementary Figure 10). This discrepancy is likely due to ctyper’s limited access to low-complexity intronic regions using k-mers.

### Sequence level diversity of CNVs in global populations

**Figure 4.**
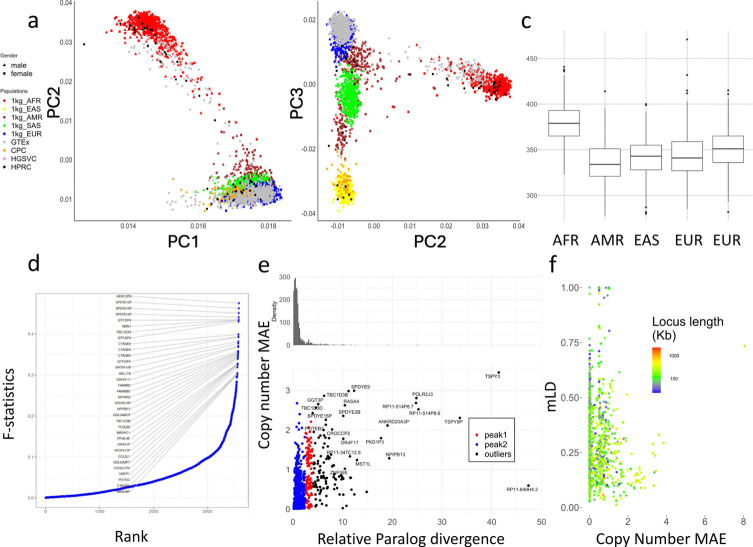
**a,b**. PCA of allele-specific copy numbers on genotype results and assembly annotations. **c,** Distribution of total autosomal gene copy numbers among 2504 unrelated 1kgp samples. **d,** Population differentiation measured by F-statistics of subgroups among different continental populations. The genes with a subgroup with an F-statistic more than 0.3 are labeled. **e,** Copy number and relative paralog divergence. Based on our genotyping results on 2504 unrelated 1kgp genomes, for genes found to be CNV to the population median in more than 20 samples, we determined the average aggregate copy number difference (MAE) between individuals and estimated the average paralog differences relative to orthologs difference. **f,** Multi-allelic linkage disequilibrium between pairs of CNV genes less than 100kb apart. The larger MAE value of each pair is used for the x-axis values. The total locus length denotes the length from the beginning of the first gene to the end of the last gene.

We used principal component analysis (PCA) to examine the population structure of PA genotypes on 2,504 unrelated 1kgp samples, 879 Genotype-Tissue Expression (GETx) samples, and 105 diploid assemblies (excluding HGSVC due to lower coverage) (Figs. 4a,b). Following standard population analysis, rare subgroups (<0.05 allele frequency) were excluded, and copy numbers were limited to 10 to balance the weights of PCs. The 1kgp, GETx, and genome assemblies were clustered by population as opposed to the data source, suggesting little bias between genotyping and assembly, or across NGS cohorts.

The top 0.1% highest weighted subgroups on PC1 have an average aggregate copy number (here labeled aggreCN) variance of 26.33, compared to an overall of 4.00 (p-value=1.11e-16, F-test). Similarly, PC2 and PC3 have mean aggreCN variance of 19.73 and 7.20, suggesting CNVs are weakly associated with sequence variants. Furthermore, PC1 is the only PC that clustered all samples into the same sign with a geographic center away from 0, suggesting it corresponds to modulus variance (hence aggreCN) if treating samples as vectors of paCNVs. Meanwhile, PC2 and PC3 were similar to the PCA plots based on SNP data on global samples^51^, suggesting they are associated with the sequence diversity of CNV genes. The total number of duplications is elevated in African populations (Fig. 4c), reflected in the order of PC1 (Fig 4a).

We examined ctyper genotypes to measure the extent to which duplications show population specificity. We used the F-statistic, a generalization of the F_st_ that accommodates more than two genotypes (Methods), to test the differences in distributions across five continental populations (Fig. 4d). In total, 4.4% (223/5,065) of duplicated subgroups showed population specificity (F-statistic > 0.2, Supplementary Table 8). The subgroups with the highest F-statistic (0.48) contain duplications of the *HERC2P9* gene that is known to have population differentiation^9,52^. Another example is a converted copy of *SMN2* annotated as a duplication of *SMN1* that is enriched in African populations (F-statistic=0.43).

We then measured whether duplicated genes were similar or diverged from reference copies, indicating recent or ancient duplications, and providing a measure of reference bias from missing paralogs. We constructed multiple sequence alignments (Methods) for sequences of each matrix, and measured the pairwise differences at non-repetitive sequences. We determined the average paralog divergence relative to ortholog divergence (Methods), which we refer to as relative paralog divergence (RPD). We also measured diversity by the mean absolute error (MAE) of the gene copy number in the populations (Fig. 4e). Based on RPD, using Density-Based Spatial Clustering of Applications with Noise^53^, we identified two peaks at 0.71 and 3.2, with MAE centers at 0.18 and 0.93. The first peak indicates genes with rare and recent CNVs, while the second peak indicates more divergent and common CNVs, often CNVs that may be inherited as different structural haplotypes. For example, *AMY1A* has a high RPD at 3.10 because of the truncated duplications of *AMY1A* (blue gene annotations in Fig. 2a). These results are consistent with ancient bursts of duplications in humans and primate ancestors^54^.

We next studied the haplotype linkage of PAs to investigate the levels of recombination at different loci. We determined multi-allelic linkage disequilibrium (mLDs) between PAs using the 1kg genotypes^55^ (Methods) for 989 subgroups that were adjacent and less than 100kb apart on GRCh38 (Fig. 4f), and found the average within each matrix. Among all mLDs, there was a strong negative rank correlation between MAEs of the copy number and mLD (***ρ***=−0.24, p-value=3.4e-15, Spearman’s rank), which is stronger than the rank correlation between mLDs and total locus length (***ρ***=−0.21, p-value = 1.5e-11, Spearman’s rank), suggesting a reduced haplotype linkage on genes with frequent CNVs. The lowest mLD=0.013 was found on *FAM90*, a gene with frequent duplications and rearrangements^56^. Not surprisingly, the 29 highest loci (mLDs > 0.7) are enriched in the sex chromosomes (N=19). Furthermore, *HLA-B* and *HLA-DRB*, had mLD >0.7 and only copy-number variation by deletion. The *HLA-DRB* deletions were only apparent after correcting HLA-specific coverage bias (Supplementary Methods). In agreement with our prior diversity analysis based on long-read data, the amylase locus has a value of 0.293 due to recombination.

### Expression quantitative trait locus (eQTLs) on pangenome alleles

To investigate the expression impact of paCNVs, we performed eQTL analysis in the Geuvadis^57^ and the GTEx^58^ cohorts. There were 4,512 genes that could be uniquely mapped in RNA-seq alignments. An additional 44 genes such as *SMN1/2* and *AMY1A/1B/1C* do not have unique exonic sequences (Methods, Supplementary Table 9), and have indistinguishable transcription products. Their expression was analyzed by pooling among all copies.

**Figure 5.**
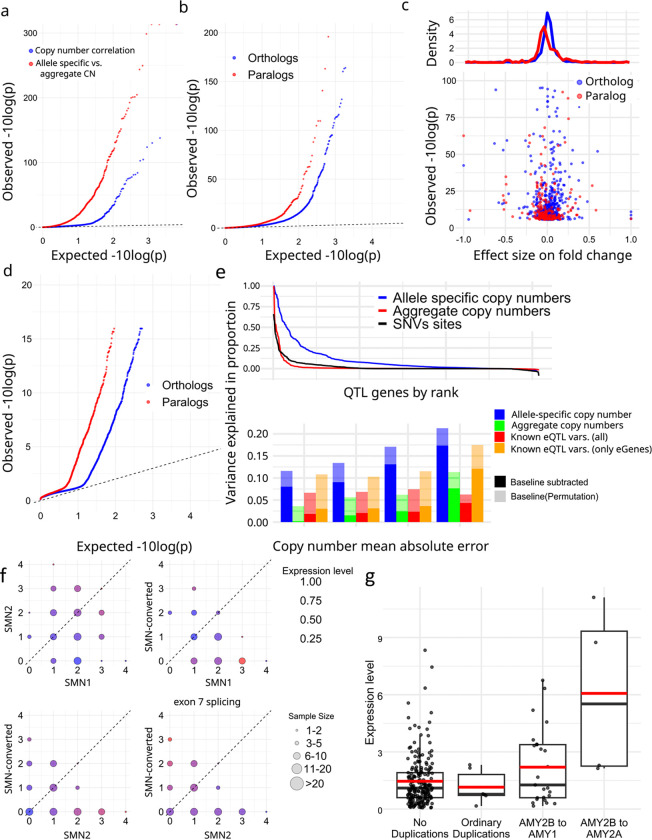
**a.** Q-Q plot of association of aggregate (*blue*) and the improvement of allele-specific over aggregate (*red*) copy numbers to gene expression in Geuvadis samples. **b,** Comparative gene expression of orthologs (*blue*) and paralogs (*red*). **c,** Fold change effect size of all alternative expressions. For all subgroups found to be significant, the fold changes as well as p-values were shown. **d,** Q-Q plot of preferential tissue expression of orthologs and paralogs. **e,** (top), Model evaluation for PAs representing gene expression diversity. (bottom) Quantification of variance explained by different representations of genomic diversity: full paCNV genotypes, aggregate copy number, and known eQTLs variants. **f,** Case study on *SMN* genes showing decreased gene expression on converted *SMN*. The average expression level in PEER-corrected Geuvadis samples is shown under different copy numbers of *SMN1*, *SMN2*, and converted *SMN*. Transcript levels are the total coverage of all isoforms, and the exon 7 splicing level is measured by counting isoforms with a valid exon 7 splicing junction. **g,** Case study on amylase genes showing increased gene expression on translocated *AMY2B* using PEER-corrected GTEx pancreas data.

We corrected expression bias using PEER^59^ with the first three PCs from reported genotypes^60^, and performed association analyses with paCNVs. After merging paCNs to aggreCNs, 5.5% (178/3,224) of transcripts showed significance (corrected-p = 1.6e-0.5, Pearson-correlation) as previously observed^36^. We then tested whether using paCNVs would provide a stronger fit by updating the aggreCNs with individual paCNVs and performing multivariable linear regression on expression (Methods). There were significant improvements in fitting for 890 transcripts (27.6%) (corrected p=1.6e-05, one-tailed F-test) (Fig. 5a).

The improved fit could be explained by non-uniform expressions of different alleles producing the same transcripts. To test this, we used a linear mixed model (LMM, Methods)^61,62^ to regress total expression to individual subgroups and estimate allele-specific expression, then compared these values to other subgroups of the same matrix (Supplementary Table 10). For subgroups within solvable matrices with >10 samples, we found that 7.94% (150/1,890) paralogs and 3.28% of (546/16,628) orthologs had significantly different expression levels (corrected with sample size = number of paralogs + orthologs, corrected-p = 2.7e-06, Chi-squared test, Fig. 5b). Overall, paralogs are found to have reduced expression (Fig. 5c), consistent with previous findings on duplicated genes^63^.

The granularity of PA genotypes enabled testing for preferential expression of paralogs among tissues. After correcting raw TPMs using DESeq2^64^, we compared expression in 57 tissues in the GTEx samples using LMMs to estimate the expression levels on each tissue (Methods, Supplementary Table 11). There was alternative tissue specificity for 132 of 2,820 paralogs (4.68%) and 225 of 19,197 orthologs (1.17%) (corrected-p = 6.4e-08, union of two Chi-squared tests, Methods, Fig. 5d).

Additionally, we used analysis of variance (ANOVA) to estimate the proportion of expression variance explained by paCNVs using PEER-corrected Geuvadis, and compared it to a model based on known SNPs, indel, and SV eQTL variants^65^ (Methods). As expected, the highly granular paCNVs explain the most variance: on average, 10.3% (14.3% including baseline). In contrast, 58.0% of transcripts are eGenes with known eQTL variants that explained valid variance by 2.14% (1.60% considering experimental noise, in agreement with a previous estimate of 1.97%^66^). On average, 1.98% of the variance was explained by aggreCNs, and 8.58% by subgroups information. When combining both paCNVs and known eQTL sites, 10.4% (19.0% including baseline) of the valid variance was explained (Fig. 5e).

We examined *SMN* and *AMY2B* genes as case studies due to their importance in disease and evolution^32,67^. The *SMN* genes were classified into three categories: *SMN1*, *SMN2*, and *SMN-converted*. We estimated the total expression of all transcripts and the expression of only isoforms with valid exon 7 splicing junctions. For total expression, no significant difference was found between *SMN1* and *SMN2* (0.281 ± 0.008 vs 0.309 ± 0.009, p=0.078, Chi-squared test). However, significant differences were found between *SMN-converted* and *SMN1/2* (0.226 ± 0.012 vs 0.294 ± 0.002, p=1.75e-07, Chi-squared test), with a 23.0% reduction in expression of *SMN-converted*. In contrast, despite with lower overall expression, *SMN-converted* had 5.93✕ the expression of *SMN2* (p=2.2e-16, Chi-squared test) regarding valid exon 7 splicing, indicating while *SMN-converted* has full functional splicing^68^, its overall expression level is lower (Fig. 5f).

For *AMY2B*, we studied the expressions of duplications when they are translocated to proximal to other *AMY* genes, such as the PAs containing *AMY1* and *AMY2B* in Fig. 2a. Using PEER-corrected GTEx pancreas data, we estimated their expressions as well as other duplications. We found that these translocated *AMY2B* genes had significantly higher expression than other duplications (1.384 ± 0.233 vs −0.275 ± 0.183, p=7.87e-09, Chi-squared test) (Fig. 5g).

## Discussion

New pangenomes present both opportunities and challenges for the study of complex genetic variation: while they reveal the landscape of complex variation, it is challenging to use these sequences to analyze biobank (NGS) cohorts. To enable this, we developed an approach to divide assemblies into pangenome-alleles: sequences that are copy number variable and inherited with low disequilibrium in gene families, and to genotype their copy number in NGS samples.

The use of ctyper genotypes increases the scope of studies on CNVs to include sequence variation between copies. For example, our finding that CNVs reflect two modes of variation: highly similar (and likely recent), and low-identity (ancient and polymorphic) duplications, is based on large cohort ctyper genotypes rather than assembly annotations. As another example, the ctyper genotypes yield tissue-specific expression of paralogs as well as relative contributions to the expression of different forms of duplications such as *SMN*.

We investigated the significant improvement of the ANOVA on PAs, whose genotypes reflect underlying sequences with multiple linked variants from known eQTL variants that are bi-allelic single variants. In contrast to PAs, there were either very few or very many eQTLs variants per gene, indicating LD (Supplementary Figure 11) as addressed by fine-mapping^69^, and increasing multiple testing burden^70^. Additionally, there was a greater proportion of variance explained among genes with more CNVs by eQTL variants, possibly explained by indirect association by LD (for example the *HPR* genes, Supplementary Figure 1). Furthermore, as the frequency of CNVs increases, the explained variance by eQTL variants increases (t= 3.80, p-value = 1.6e-04, Pearson’s correlation), while the number of eQTL variants decreases (t = −4.79, p-value = 2.1e-06, Pearson’s correlation), suggesting that larger effects like CNVs might overshadow the discovery of other variants not in LD. Furthermore, gene expression might not be a linear additive effect of all variants^71^. For example, although *SMN*-converted contains variants that are either from *SMN1* or *SMN2*, its overall expression is lower than both. In this manner, the concept of PAs may have a wider potential for future genome-wide association analysis (including non-CNV genes).

Due to the limited sample size, our associations are based on subgroups rather than individual PAs. Different cohort sizes may require different levels of granularity when defining subgroups. For example, the three subtypes of *SMN-converted* showed little difference in expression. Our current classification on highly-similar subgroups was designed for biobank cohorts, so smaller cohorts may need to test on subgroups that aggregate more PAs. The granularity of genotyping is additionally defined by the length of PA sequences; genotypes using shorter PAs will more accurately reflect NGS samples, while longer sequences can preserve larger phasing and may be preferable in regions with low recombination, such as *HLA-DRB*.

Ctyper also has limitations. First, while it is possible to detect CNVs smaller than PA units using ctyper (Supplementary Methods), full support requires additional benchmarking data. Second, ctyper currently does not provide confidence values for genotypes. Finally, although the visualization tool we provide might help, the high-dimensionality PAs do increase the complexity of interpretation and association analysis.

As new high-quality references become available, we anticipate ctyper to be a useful method for interpreting the association between sequence-resolved CNV and traits at scale.

## Online Methods

### Constructing pangenome allele database

We initiated our study by identifying gene duplicates in pangenome assemblies. Our pangenome cohort was composed of assemblies from the Human Pangenome Reference Consortium (HPRC) (N=92, excluding HG02080 due to abundant flagged regions), the Chinese-Pangenome Consortium (CPC) (N=114), the Human Genome Structural Variation Consortium (HGSVC) (N=18, only Pacbio HiFI assemblies were used), two telomere to telomere diploid assemblies (N=4), and reference genomes (GRCh38 including alternative loci and CHM13 T2Tv1). The gene database used for annotation was GENCODE v39 based on the GRCh38 reference genome.

The initial application of this study was on 3,203 genes known to have copy number variation detected by the HPRC and CPC studies.

We organized genes into gene ‘query sets’ where each query set encompassed genes with functional or similar sequences including pseudogenes and genes with distant homologies within the same gene family. The query sets were initially defined based on genes with shared name prefixes, and were used to locate copies of duplicated genes within the pangenome.

Direct sequence alignments might overlook sequences such as small pseudogenes and diverged paralogs, potentially creating biases in our genotyping. To address this, we developed a more sensitive alignment scheme to detect all copies of genes in the pangenome. For each query set, we used low-copy *k*-mers (*k* = 31) that appeared fewer than 255 times in the CHM13 genome, derived from all initial reference genes, to help locate similar genes. We searched for these *k*-mers in each of the pangenome assemblies and references. We then identified *k*-mer hotspots defined as maximal intervals of mapped *k-mers* containing more than 200 *k*-mers within any 1,000-base window within the interval. To aid in mapping small and fragmented pseudogenes, we included an additional criterion to define hotspots: the presence of 50 exonic *k*-mers within the same interval search.

Subsequently, we used BLASTn^1^ to refine the boundaries of each hotspot by aligning all reference genes in this query set to each *k*-mer hotspot extended by 5,000 base upstream and downstream flanking sequences.

The *k*-mer-defined hotspots include both individual loci mapped by multiple genes from a query set as well as loci with tandemly duplicated genes multi-mapped by individual genes in a query set. To account for this redundancy, we merged alignments that were less than 10,000 bases apart (together with 5,000 upstream and downstream flanking sequences, this merges genes within 20,000 bases distance), causing tandemly duplicated genes to be merged into single loci. To avoid genotyped loci that may be split by recombination, if an intron exceeded 20,000 bases, we divided the locus at the midpoint of the introns. To ensure the overall sequence size was comparable, flanking sequences both upstream and downstream were adjusted to achieve a total length of 15,000 bases. These methods aimed to standardize the size of each sequence to be roughly 30,000 bases, approximating the size of linkage disequilibrium (LD) blocks. The collection of all sequences mapped by a query set is referred to as initial matrix-sequences.

### Determine homology sequences and low-copy *k-mer* lists

Because the initial matrix-sequences were defined from aligned query sets that potentially arbitrarily grouped genes with unrelated sequences based on name, we used subsequent steps of refinement to exclude unrelated sequences.

Initially, for each genome, we extracted all *k*-mers exclusive to aligned locations of the matrix-sequences (hence not found elsewhere in the genome). We also filtered out repetitive *k*-mers with more than two-thirds of the *2*-mers and *3*-mers redundant, as these were mostly associated with highly repetitive DNA, such as Variable Number Tandem Repeats (VNTRs), microsatellites, and transposable elements. Additionally, we excluded *k*-mers demonstrating a high (>70%) or low (<30%) GC content bias^2^.

Subsequently, we filtered sequences predominantly composed of the *k*-mers removed in the previous step. As an additional filtration, we filtered out genes from the non-confident regions reported by the HPRC, as well as truncated genes from small scaffolds. The genes included needed to be at least 10,000 base pairs away from both ends of a scaffold, except for sequences from genes taken from the reference genomes located at the telomeres.

The remaining sequences were then categorized into partitions based on the number of shared *k*-mers. This classification was achieved using graph partitioning. Each sequence was represented as a node, and edges were made between node pairs sharing an excess of 500 low-copy *k*-mers, except for *NBPF* and *ANKRD* genes, for which a higher threshold of 2,000 low-copy *k*-mers was set to further reduce the sizes of partitions for computational efficiency in later analysis. Each partition represents a singular matrix, and the list of low-copy *k*-mers specific to each matrix was compiled and termed as ‘*k*-mer list’.

### *k*-mers based phylogenetic tree construction

Initially, for every partition, we assembled a *k*-mer matrix, M, that encapsulates all sequences in the matrix. Within this matrix, individual rows represent distinct gene sequences, while each column corresponds to a unique *k*-mer from the *k*-mer list exclusive to the matrix. The matrix cell values are the counts of each *k*-mer present in the respective gene sequence, which is mostly 0 or 1, but occasionally more than 1 when there are low-copy repeated sequences in the gene, or the row represents a tandemly duplicated locus.

The matrix M allows us to measure the concordance between any two sequences, G_i_ and G_j_, by calculating their inner product, denoted as <G_i_ * G_j_> . Consequently, the norm matrix, N = M * M^T^, reflects the *k*-mer concordances for all sequence pairs within the matrix.

We constructed a similarity matrix, S, where S_i,j_ is the cosine similarity of G_i_ and G_j,_ representing the sequences. The cosine similarity for any two sequences, G_i_ and G_j_ can be obtained by normalizing the norm matrix N according to the squares of *k*-mer vectors (approximately equal to sequence lengths) of the sequences in question.

Finally, we used the Unweighted Pair Group Method with Arithmetic Mean (UPGMA) algorithm on the similarity matrix S to generate the phylogenetic tree for each partition.

### Clustering of pangenome alleles into highly-similar subgroups

With each group of sequences we build a matrix for, we used phylogenetic trees for the annotation and classification of closely related groups of alleles, which we term ‘highly-similar subgroups’. The classification of highly-similar subgroups is guided by two primary criteria applied across all subgroups:

Homogeneity within subgroups: A subgroup must exhibit near-identical characteristics amongst its members, which is quantified by ensuring the largest *k*-mer distance between any two members does not exceed 155 *k*-mers, which is roughly equivalent to the variation caused by 5 single nucleotide polymorphisms or a structural variation of approximately 95bp, such that subgroups are capable of representing most common variants in about 30kb range.

Distinctiveness of subgroups: Each subgroup must be distinct from its neighboring subgroups. This is measured using a *k*-mer F-statistic score, which must exceed 2 when compared with adjacent subgroups. In cases where subgroups are composed of fewer than three members, the F-statistic may not be reliable; hence, we default this score to 0 for such small subgroups, but change the cutoff of the former criteria to 155 * 3 to detect singleton rare events.

Employing a ‘bottom-up’ recursive approach starting from leaves, we applied these criteria to all subgroups, aiming to identify and report the largest possible highly-similar subgroups. These are later used to identify equivalent loci after genotyping.

### Pangenome allele annotation relative to the reference genome

We annotate CNV events and duplicated alleles in the pangenome assemblies relative to the GRCh38 genome. This requires us to find out the corresponding GRCh38 gene for each pangenome allele. However, this is a known challenging problem of orthology assignment^3^.

First, PAs often align to multiple paralogs on GRCh38, and the gene overlaps with their liftover locations may not be the most similar reference gene due to gene conversion and translocation (Figs. 1f and 2a). To address this problem, we designed a method to match PAs to their closest GRCh38 genes based on *k*-mer similarity. For every haplotype, we obtained all pairwise similarities between each of its PAs to each of GRCh38 PAs. We matched PAs to their most similar GRCh38 PAs, starting from the most similar pair, until all PAs were matched or failed to match (had no reference gene with >90% similarity). Secondary redundant matches (match to reference genes that had already been matched) were annotated as duplications (distal).

Second, the formerly failed-to-match PAs were likely alleles with large SVs, such as insertion, deletion and local proximal duplications. We attempted to lift them back to GRCh38 using their flanking sequences (100kb on either side). Because it is challenging to directly liftover genes in the regions with large segmental duplications, we designed this liftover to be in two steps. First, we lifted PAs to the region with the best local alignment coverage, allowing SVs to break alignments into smaller units. Second, we performed a global pairwise alignment between PAs and the lifted region to locate the best-aligned gene with the presence of local translocations and tandem duplications (Supplementary Methods).

Third, to annotate the proximal duplications mentioned in the last step as well as to annotate diverged paralogs that failed to match from both prior methods, we annotated PAs regarding the gene transcripts. We aligned all exons from the same matrix to PAs, and based on the exon orders and alignment scores, and determined the optimal combinations of transcripts on each PA (Supplementary Methods). The PAs containing no exons were annotated as introns and PAs containing only transcripts of other non-interested genes were annotated as decoys. Introns and decoys were usually filtered out from analysis and the remaining PAs were considered as valid alleles, including pseudogenes that have no intact protein-coding transcripts and putative protein-coding genes with intact protein-coding transcripts.

It is important to note that because proximal duplications may be highly associated with inheritance and potentially interference with each other functionally, such as co-expression (which was found between *HP* vs *HPR*, Supplementary Figure 1), and exonic expansion can be found in gene *LPA* and *NBPF,* we treated PAs with proximal duplications as an alternative type of orthology PA, instead of treating them as multiple independent copies of singletons.

### Definition of orthologs and paralogs in the pangenome

Based on annotation results, to illustrate the relation of PAs to their corresponding reference genes regarding orthology and sequence similarities, we classified PAs into four categories, including two types of orthologs and two types of paralogs:

1. Reference alleles are alleles in the same subgroup with GRCh38 alleles, representing the alleles almost identical to the reference sequences.
2. Alternative alleles were orthologs located at the same genomic locus as the reference gene but are distinctly in different subgroups from GRCh38 alleles, including alleles that have a list of small variants in strong linkages or alleles that have large structural variations, such as proximal gene/exon duplications or deletions, as observed in genes like *HPR*, *NBPF*, and the *CYP2D6* (star-alleles) gene.
3. Duplicated paralogs (alleles) consisting of paralogs that have been duplicated to different loci from the reference alleles. Despite being translocated, they retain similarities (>80% in *k*-mers) to the reference alleles. These alleles often reflect large, recent segmental duplications in the genome, including similar paralogs, such as *AMY1A*, *AMY1B*, and *AMY1C*, which are still often considered the same gene despite their distinct locations.
4. Diverged paralogs (alleles) not only differ in their translocation status but also have sequences that are significantly divergent (<80% in *k*-mers) from reference alleles, such that cannot be simply assigned to a single reference gene. These were typically characterized by highly diverse non-reference paralogs, incomplete gene duplications, and novel processed pseudogenes. An illustrative example of diverged paralogs is found among amylase genes, which indicates a proximal translocation event between *AMY1* and *AMY2B* genes.

### Comparison of pangenome alleles to other genomic representations

First, we characterized the information gained by representing a genome as paCNVs compared to copy-numbers of reference alleles. For each PA, we used the nearest neighbor in our pangenome database as a proxy for the optimal genotyping results of samples containing that PA, and its closest GRCH38 genes for comparison of single-reference-based CNV. The nearest neighbor demonstrated an average 94.7% reduction in differences compared to GRCh38 matches; 57.3% had identical nearest neighbors showing common paCNVs alleles.

We then assessed the proportion of subgroups identifiable by *k*-mers uniquely shared by all their members, analogous to SUNKs. Only 38.8% of subgroups (with at least three members) contain such *k*-mers (Fig. 2e). For example, no SUNKs exist between *SMN1*, *SMN2* and *SMN-converted* due to gene conversion (Fig. 1f). However, there are unique combinations of *k*-mers used by ctyper genotyping.

We investigated the extent to which pan-genome alleles are linked to large haplotype structures. We found out that this linkage drops quickly at larger scales due to allelic variation and recombination creates unique combinations that cannot be represented during leave-one-out analysis. Using amylase genes as an example, there were 40% (90/226) of haplotypes that could not be represented, particularly those with greater copies than GRCh38 (45/67). When all PAs devoid of SV were combined into a single large subgroup, 20% (46/226) of haplotypes remained singleton, especially those with additional copies (26/67). Furthermore, many subgroups, such as the novel PAs containing both *AMY1* and *AMY2B* in proximity, are found within different structural haplotypes (Fig. 2a). While such issues may be mitigated by a larger pangenome, genotyping at the level of PA increases the ability to identify the genetic composition of an NGS sample at highly variable multicopy gene loci.

### Justification of highly-similar subgroups in representing population diversity

We characterized highly-similar subgroups to justify if they can well capture sufficient population diversity. We found the average pairwise *k*-mer cosine similarity is 98.8% within each highly-similar subgroup, compared to the average 94.2% to their corresponding reference, showing 5.03✕ in reduction, noting one base change adds up to *k* different *k*-mers. Between two phylogenetically neighboring subgroups having at least three members each, the between-type variance is 6.03✕ greater than the within-type variance, showing a strong hierarchy structure, and most genetic diversity may be represented using a small number of subgroups. Both criteria suggest that about 80% of total population variation could be represented by highly-similar subgroups.

Then, we performed saturation analysis using a recapture model^41,42^ to estimate the extent to which the current cohort represents all possible subgroups among worldwide populations. This estimates the average number of novel subgroups within each new genome at increasing cohort sizes. Among the current cohort, each new African genome has 221 of 4363 (5.1%) novel subgroups, and non-Africans have 56 of 4358 (1.3%).

### Genotyping NGS sample with ctyper

#### Initial solution based on matrix solution

The goal of ctyper is to select a list of pangenome alleles and determine their individual copy numbers to represent the CNVs of unknown NGS samples. Instead of sequence alignment, our genotyping is based on *k*-mer comparison, which is not only more efficient but also not affected by misalignments that are frequent in the genomic regions enriched in structural variation and repetitive elements. Another advantage is that there is little bias in *k*-mers between high-quality long-reads and NGS data^4^, so the *k*-mer data based on assemblies can be applied to predicting NGS data.

The genotyping proceeds per gene. Given an NGS sample and a *k*-mer matrix M derived from pangenome allele annotation, we generate a vector V for an NGS dataset that includes the counts of each *k*-mer found in the matrix for the NGS sample normalized by the sequencing coverage. We seek to find a vector X of the copy-number of each pangenome allele that minimizes the squared distance to the *k*-mer counts we observed in NGS data, e.g. argmin_x_ (ǁ M^T^ * X − V ǁ). Compared with absolute distance, squared distance is more suitable for the normal-like noise in NGS data^5,6^. Although it is possible to directly obtain an integer solution using mixed-integer linear programing (MILP), this is NP-hard^7^ and can only be used with very few variants/*k*-mers^8,9^. This restricts the use of MILP on the pangenome. The relaxed non-integer solution has an analytic solution that can be efficiently solved. In essence, the computational problem is akin to a multivariable linear regression.

To make the solution closer to the maximum likelihood estimation, during the regression, we rescaled the dimensions of *k*-mers to even their expected uncertainty. Assuming the observation of *k*-mer copy number follows a negative binomial distribution with the dispersion small enough to be distinct from Poisson^6^, the expected variance is roughly proportional to the square of observation, thus we weighted the *k*-mer to the square of the reciprocal of their observed copy number. We also applied smaller weights (adjust=0.05) on singleton *k*-mers (observed in only one PAs and not observed in NGS as well) because they are more likely to be sequencing errors.

#### Integer solution based on reversed phylogenetic rounding

The next step is what we named reversed phylogenetic rounding. Initial linear regression often yields solutions in the form of small floating-point values, where the alleles with the highest coefficients are not necessarily those closest to query genes. However, as shown by mathematical analysis (Supplementary Methods), there are relationships between the initial least-error solution and the true integer solutions under a phylogenetic framework:

1. Non-negative solutions: Without uncertainty in predicting k-mer copy numbers, the least-error solution should be non-negative. Therefore, we obtain a non-negative least-error (NNLS) solution via Lawson-Hanson algorithm^10^.
2. Total copy number estimation: The sum of the initial solutions should approximate the total number of true integer solutions, allowing us to estimate the total gene copy numbers in the querying sample.
3. Fractality of least square error solution on phylogenetic tree: We should if a solution is the least square error solution of the tree, it is also the least square error solution within each clade, allowing greedy method to perform.
4. Phylogenetic position prediction: On a binary phylogenetic tree, the branch with a shorter vector distance to the genes in the querying sample will have a larger sum of coefficients (inversely proportional to distance). This relationship enables us to predict the phylogenetic position of each gene in the querying sample.
5. Large database effect: In large databases, having more genes highly similar to query genes tends to distribute the total coefficients across them, resulting in smaller individual coefficients. However, the total sum of these coefficients increases, improving the precision of phylogenetic position prediction, and this effect does not plateau.
6. We also show that even with sequencing uncertainties for NGS at about 30-fold coverage, the model’s precision remains, and it is not the bottleneck of the model.

Given the high “convergence” and fractality of the solution on the phylogenetic tree in large databases, we developed a greedy algorithm to efficiently convert non-integer solutions into integer solutions. This iterative algorithm follows a bottom-up approach, starting from the leaves and progressing toward the root. At each hierarchical level, non-integer values are rounded to the nearest integer solution that minimizes the overall residual, while any remainder is propagated to the next level. Because at each level of the hierarchy, there are only two remainders from either branch of the tree, this solution is highly efficient. The pseudo-code for this algorithm (naive version and optimized version) is provided in the Supplementary Methods.

### Trio analysis

Trio analysis is to determine if the genotype combinations of the child-father-mother show possible Mendelian violations. When the copy number of a child is 0, the parents need to be 0 or 1; When the copy number of a child is 1, the parents can not both be 0 or both be 2; When the copy number of a child is 2, the parents both need to be 1 or 2. When the copy number of a child is more than 2, the parents need to have the sum to be greater or equal to this number.

### Leave-one-out comparison of genotyping results to pangenome assemblies

To find out the extent to which the genotyping results can represent the individual small variants on each PA, we aligned PAs in the original assemblies to their corresponding PAs in the genotyping results.

First, the original assembly PAs were paired one-to-one with genotyped PAs by a greedy method. We obtained all pairwise similarities in *k*-mer between each pair of the PAs across original assemblies and genotyping results. Starting from the most similar pair, we paired those alleles without replacement and iterated this until all original assemblies PAs were either paired or failed to be paired (has no genotyped PAs with >90% similarity, considered as copy number).

Second, the paired PAs were then aligned using the global pairwise alignment tool Stretcher^11^ for masked sequences and Locityper^12^ for unmasked sequences. From the global alignments, we obtained the number of mismatched bases in the unmasked region, where the low copy repeat *k*-mers are used in *k*-mer matrices. We compared ctyper’s performance with Locityper regarding unmasked sequences using the full set of databases, and Locityper’s results were obtained from their uploaded zenodo tables.

### Classification of errors

We classified four types of errors for our benchmarking:

1. False positive: the genotyping results have an additional copy;
2. False negative: the genotyping results have a missing copy;
3. Miss typing: assign a copy to incorrect type;
4. Out of reference: the singleton type among the pangenome and lost reference during leave-one-out.

### Benchmarking *HLA, KIR* and *CYP2D* genes with public nomenclatures

We benchmarked the results on *HLA* and *CYP2D* genes from all 39 HPRC samples with NGS data from both full-set and leave-one-out analyses. First, we labeled all IPD-IMGT and CYP2D-star annotations of PAs. For *HLA* and *KIR*, we annotated using Immuannot^13^, and for *CYP2D6*, we annotated using Pangu^14^. Using those annotations, we converted genotyped PAs sequences into public nomenclatures and compared nomenclatures with the annotation results of the assemblies from the same samples. The benchmarking results of *HLA* were compared with T1K with its default settings and the benchmarking results of *CYP2D6* were compared with Aldy with its default settings.

We also benchmarked SNP calling on *CYP2D6*, and compared with Aldy, with its default settings. We took the phased results of Aldy and matched them to their corresponding original PAs. In a range of about 6 kb, where the variants could be found (first SNP reported at chr22:42126309, last SNP reported at chr22:42132374), Aldy genotyped the variants with an F1 score of 85.2%, and ctyper genotyped the variants with an F1 score of 95.7%.

We ran T1K for HLAs with commands: “run-t1k --abnormalUnmapFlag -t 12 --preset hla-wgs --alleleDigitUnits 15 --alleleDelimiter : -c hlaidx_dna_coord.fa -f hlaidx_dna_seq.fa -b $CramFile -o $Output”, and ran T1K for KIRs with commands: “run-t1k --abnormalUnmapFlag -t 12 --preset kir-wgs --alleleDigitUnits 15 --alleleDelimiter : -c kiridx_dna_coord.fa -f kiridx_dna_seq.fa -b $CramFile -o $Output”. We ran Aldy on CYP2D6 with the command: “aldy genotype -p wgs -g CYP2D6 $CramFile --reference $HG38 --output $Output”.

### Total number of duplication events from genotyping results

Based on ctyper’s genotyping results, we calculated the total number of duplication events for each 1kgp sample, excluding 7 samples due to having extreme values different from the population mean by more than five standard deviations. The total number of each reference gene is measured in each genome and compared to GRCh38 chromosomes excluding alternate haplotypes. Each duplication event is called if the genome has more copy number than twice that of GRCh38, excluding decoys/introns and sex chromosome genes. The total number of duplication events is reported for each genome. It is important to note that these duplications also included pseudogenes and small exonic fragments besides known protein-coding genes.

### Measuring F-statistic values

Because subgroups may have copy numbers beyond two and may not be applicable to the Fixation index (F_st_), we instead used the F-statistic value to measure the population specificity of subgroups. The F-statistic value is based on the F-test, where we obtained the variances of copy numbers within all continental populations (within-group variance), and used it to divide the variances of copy numbers across different populations (between-group variance).

### Relative paralog divergence

Relative paralog divergence (RPD) measures the mean divergences of the paralogs to other alleles, in relative to the mean divergence between only orthologs. RPD was determined for each reference gene and based on the graphic multiple sequence alignments (gMSAs, Supplementary Methods) of PAs assigned to that reference gene as well as ctyper’s genotyping results.

First, the divergence value was determined for each pair of PAs assigned to the same reference gene. It was measured based on the alignment scores of unmasked bases (misalignment and gap open = −4, and gap extend = 0, normalized by total alignment length) from gMSAs.

Second, we obtained the mean divergence of the orthologs by averaging divergence values between the two PAs from samples with CN = 2.

Third, we then determined the population median copy numbers for each reference gene, and divided samples into those with additional copy numbers (copy numbers more than the median) and those with no additional copy numbers (copy numbers not more than the median).

It is unreliable to directly distinguish the paralog from orthologs due to complex rearrangements (e.g. Figure 2a). To overcome this limitation and only obtain the divergence values from additional copies, we performed statistical estimation based on large populations. We first estimated the mean divergence values from samples with no additional copy numbers and used it as the unit baseline B. When the population median CN = Y, because there are Y(Y−1)/2 pairs, then the total baseline is B * Y(Y−1)/2, which will be subtracted from total divergence values of samples with duplications, and Y(Y−1)/2 will be subtracted from the total number of pairs (the denominator) as well.

After subtracting the total baseline, the mean paralog divergence value of the additional copies was determined for all samples with additional copy numbers. This mean paralog divergence was then normalized by the mean divergence of the orthologs obtained in step two.

### Multi-allelic linkage disequilibrium

Multi-allelic linkage disequilibrium (mLDs) is an analytic continuation of SNP-based bi-allelic linkage disequilibrium to allow computing linkages between multiple genotypes on neighboring loci. When there are only two genotypes on both loci, mLDs equal LD value. When there are more than two genotypes, mLDs measure LDs between each pair of genotypes across different loci, and take the weighted average of all pairs. This weight is the product of both allele frequencies of the pair.

### Defining transcripts for expression analysis

We represented each gene by the major transcripts from the MANE (Matched Annotation from NCBI and EMBL-EBI^15^) project. Second, individual exons were aligned. Transcripts were recursively clustered together if they overlapped with previously clustered transcripts with more than 98% overall similarity taking the average similarity of all aligned exons from the transcripts. We consider these clusters as the same transcript. Third, for each transcript, we identified all its exons and looked for unique exons that did not overlap with exons from other transcripts. Fourth, we used these unique exons to represent each transcript and filtered out transcripts that have no unique exons (2079 out of 2579 filtered genes were known pseudogenes). Lastly, we assigned PAs to each transcript if they contained any of the corresponding unique exons with at least 98% similarity.

### Expression correction

For individual tissue analysis, similar to the prior study^16^, we logistically corrected the raw TPMs using the tool PEER together with the first three principal components obtained from reported genotypes in chr1^17^. For cross-tissue analysis, we corrected raw TPMs using DESeq2^18^.

### Association between CNVs to gene expression

We first associated gene aggregate copy number to expression levels using Pearson correlation (linear fitting). The p-values and residuals of this fit were recorded. To test if including allele-specific information would improve the correlation, we used the ctyper pangnome allele-specific copy numbers to replace the aggregate copy numbers to perform multi-variable linear regression using allele-specific copy numbers as dependent variables and gene expression level as independent variables. We compared the residuals of multi-variable linear regression with residuals from Pearson correlation using the F-test, and one-tailed p-values of the reduced residual was reported. Both p-values were corrected by the number of transcripts tested (N=3,224).

### Linear mixed model

We performed linear mixed modeling to measure the individual expression of each subgroup. We used the total gene expression values as the vector of observed dependent variables, different subgroups as the vector of independent fixed variables and the copy numbers from ctyper genotyping results were used as their coefficient matrix. The effect sizes of fixed variables were then solved using ordinary least squares (OLS) regression.

### Alternative expression of subgroups

To determine whether a subgroup has an alternative expression level compared to other subgroups of the same gene, we merged all other subgroups assigned to the same reference gene into a single variable, separating from the subgroup currently being tested. Additionally, we included other factors, such as paralogs that might also influence total expression, as additional parameters to adjust for their potential interference. For subgroups within solvable matrices with more than ten non-zero expressions, using a linear mixed model and the R lm function^19^, we regressed the expression values to all variables to get their effect sizes. We then compared the effect size of the currently tested subgroup and the effect size of other subgroups of the same gene using Chi-squared distribution with the linearHypothesis tool^20^. This p-value was then corrected by the number of total subgroups tested (N=18,518).

### Across tissue expression comparison

In order to determine if a subgroup has an alternative most expression tissue compared to other subgroups of the same gene, we merged all other subgroups assigned to the same reference gene into a single variable, separating from the currently tested subgroup. Additionally, we included other factors, such as paralogs that might also influence total expression, as additional parameters to adjust for their potential interference. For subgroups within solvable matrices with more than 10 non-zero expressions, we performed linear mixed models to estimate the gene expression level of each subgroup within each of the 57 tissues in GTEx V8. The tissue with the highest expression level was recorded and compared to the tissue with the second highest expression using the Chi-squared test. We then compared the results between the currently tested subgroup and all other subgroups of the same gene to see if they had a different highest expressed tissue. When the highest expressed tissues were different, we tested the p-value of either event by combining the p-values from both sides as p-combined = p1 + p2 − p1 * p2. This p-value was then corrected by the number of subgroups tested on all 57 tissues (N=776,902).

### ANOVA (Analysis Of Variance) tests on gene expression

We first measured the total expression variance for each eQTL transcript, filtering out units with per-sample variance less than 0.1 to exclude genes not sufficiently expressed in the Geuvadis cohort. We estimated experimental noise by measuring expression variance between different trials of the same individuals (mean = 10.5% of the total variance) and excluded transcripts with experimental noise exceeding 70% of the total variance, resulting in 639 total transcripts on expression. We applied the one-in-ten rule to restrict the number of variants tested to be not greater than 45 (10% of the sample size) to avoid over-fitting. We filtered out 18 units involving more than 45 PAs; When there were more than 45 known eQTL variants, we used 45 variants with the lowest p-values. The valid expression variance was obtained by subtracting experimental noise from the total expression variance. Using ANOVA, we estimated the explained valid variance and adjusted the results by subtracting a baseline, defined as the mean expression variance explained by permuting the orders of all samples (estimated by the mean of 100 trials). If there were no reported eQTL variants, a value of 0 was used for known eQTL variants.

For paCNV, we further investigated the part of variance explained by gene aggreCNs, applying ANOVA to a random matrix with aggreCN information, with randomly assigned subgroups, but with the total copy number equal to the original matrix. We subtracted the variance explained by this random matrix from the total explained variance to obtain the variance explained by subgroup information.

## Supplementary Material

Supplement 1

Supplement 2

Supplement 3

Supplement 4

## Data Availability

Software: https://github.com/ChaissonLab/Ctyper. Allele database and annotations: https://doi.org/10.5281/zenodo.13381931, and the construction code: https://github.com/Walfred-MA/PATs. Benchmarking and analysis code: https://github.com/Walfred-MA/CNVAnalyze.
