# Supplementary material for: Genotyping sequence-resolved copy-number variations using pangenomes reveals paralog-specific global diversity and expression divergence of gene duplication
Source: bioRxiv. 2025 Feb 20:2024.08.11.607269. Originally published 2024 Aug 11. Preprint. [Version 5] doi: 10.1101/2024.08.11.607269 (PMC11326217; doi:10.1101/2024.08.11.607269)
Supplement: Supplementary file 1 [file media-1.pdf]

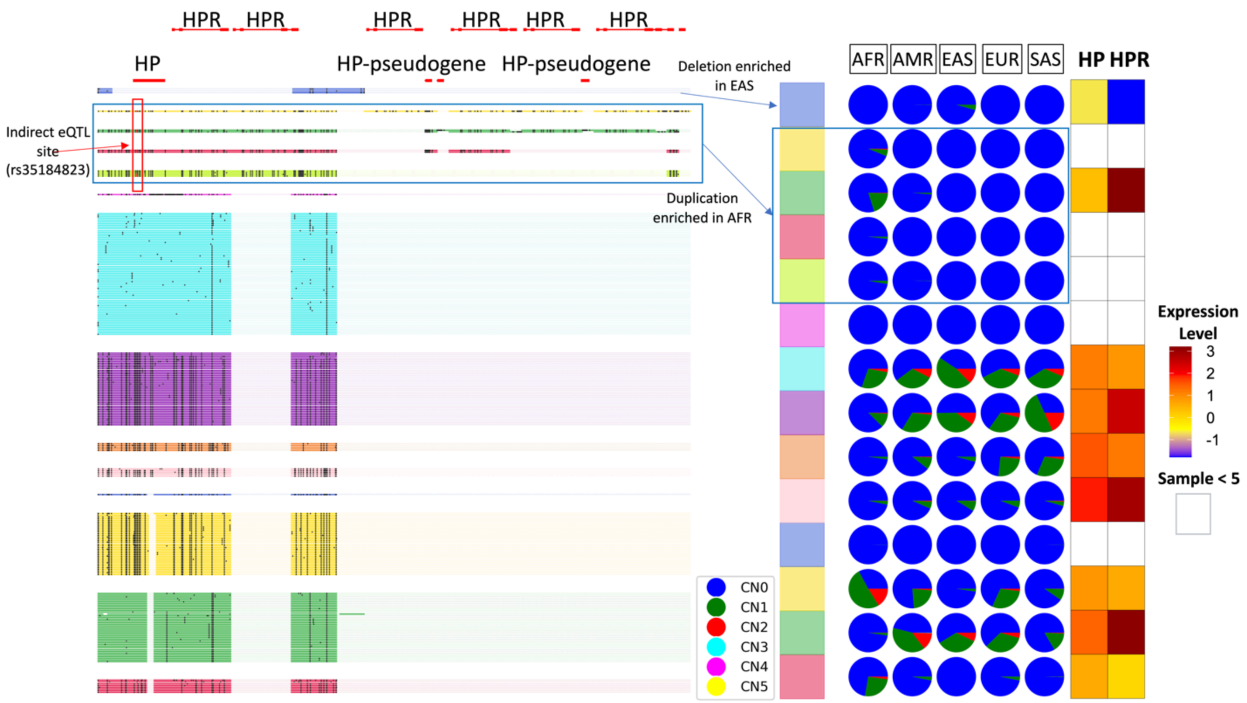

Supplementary Figure 1. Comprehensive case study on *HPR* and *HP* genes. Left: the mutant plot for all PAs on the *HPR* and *HP* locus based on graphic MSA; The proximal duplication and an SNP associated with proximal duplications are annotated (rs35184823, reported by GETx). Middle: the genotyping results are based on 2504 unrelated 1kpg samples. Each pie chart represents the copy number distribution of each subgroup in each population. Right: the expression level for each subgroup estimated from the genotyping results of GETx samples and published GETx whole blood TPM, using a linear-mixed-model. The subgroups found in fewer than 5 samples are excluded. Co-expression has been found between *HPR* and *HP* (t = 24.089, p-value = 6.2e-78, Pearson correlation).

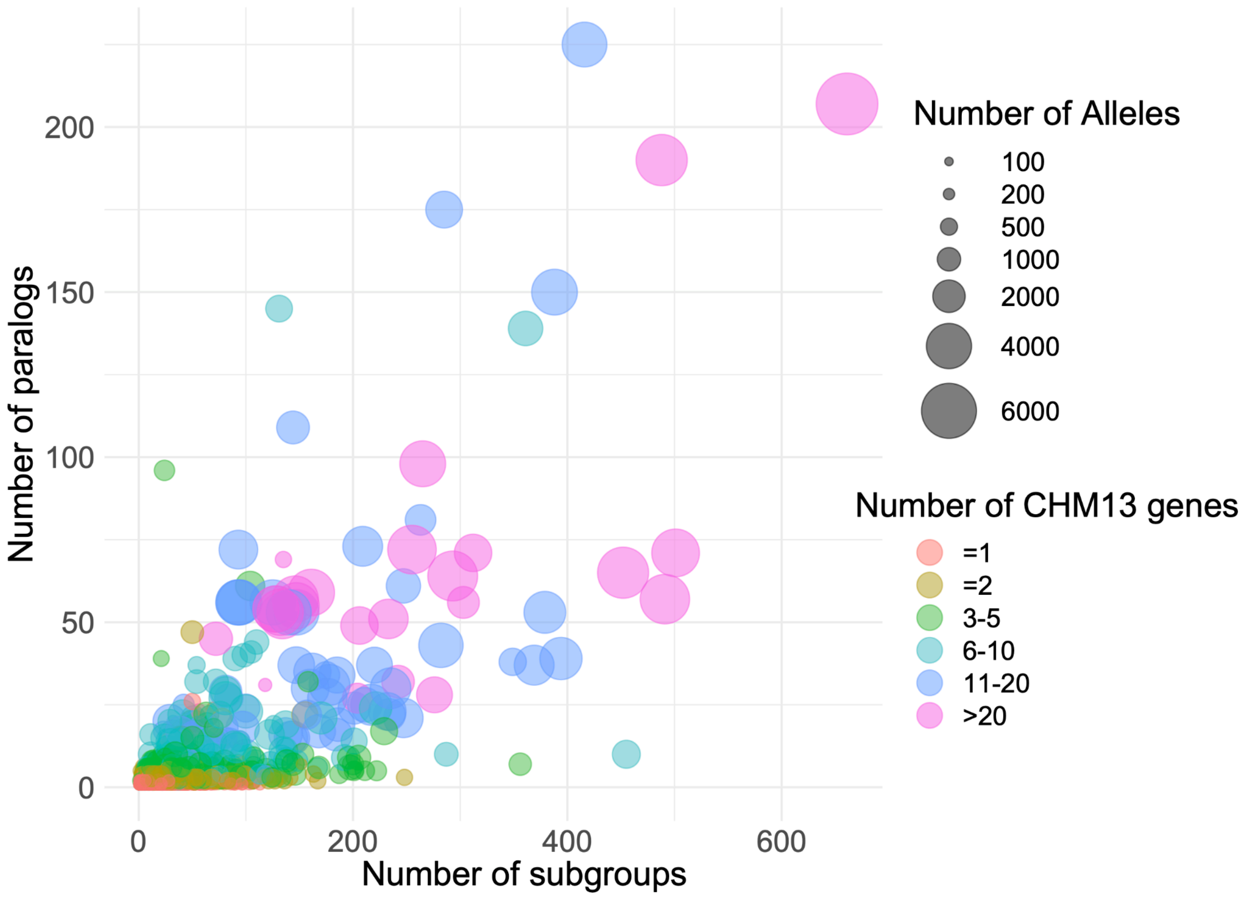

Supplementary Figure 2. The composition of all matrices. Each dot represents each of 3,307 matrices. The correlation has been found between the number of alleles and the number of subgroups (t=54.6, p-value = 1e-179), but stronger between the number of paralogs and the number of subgroups (t=72.9, p-value = 1e-219).

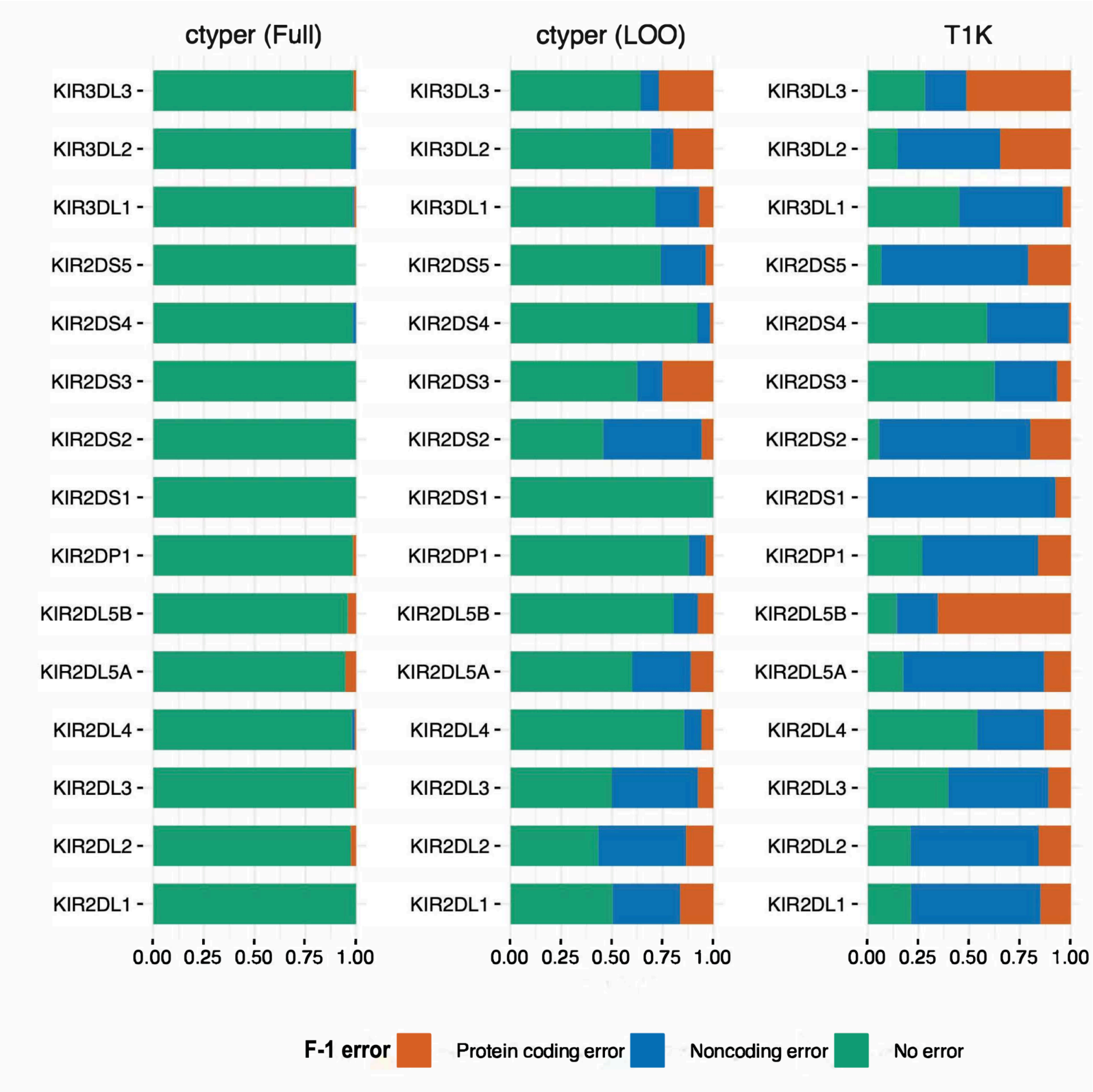

Supplementary Figure 3. Benchmarking of KIR genes using the full-set and leave-one-out databases. Ground truth annotations are given from Immunotyper on assemblies. Protein coding errors are errors on the first three digits and noncoding errors are errors on the remaining digits.

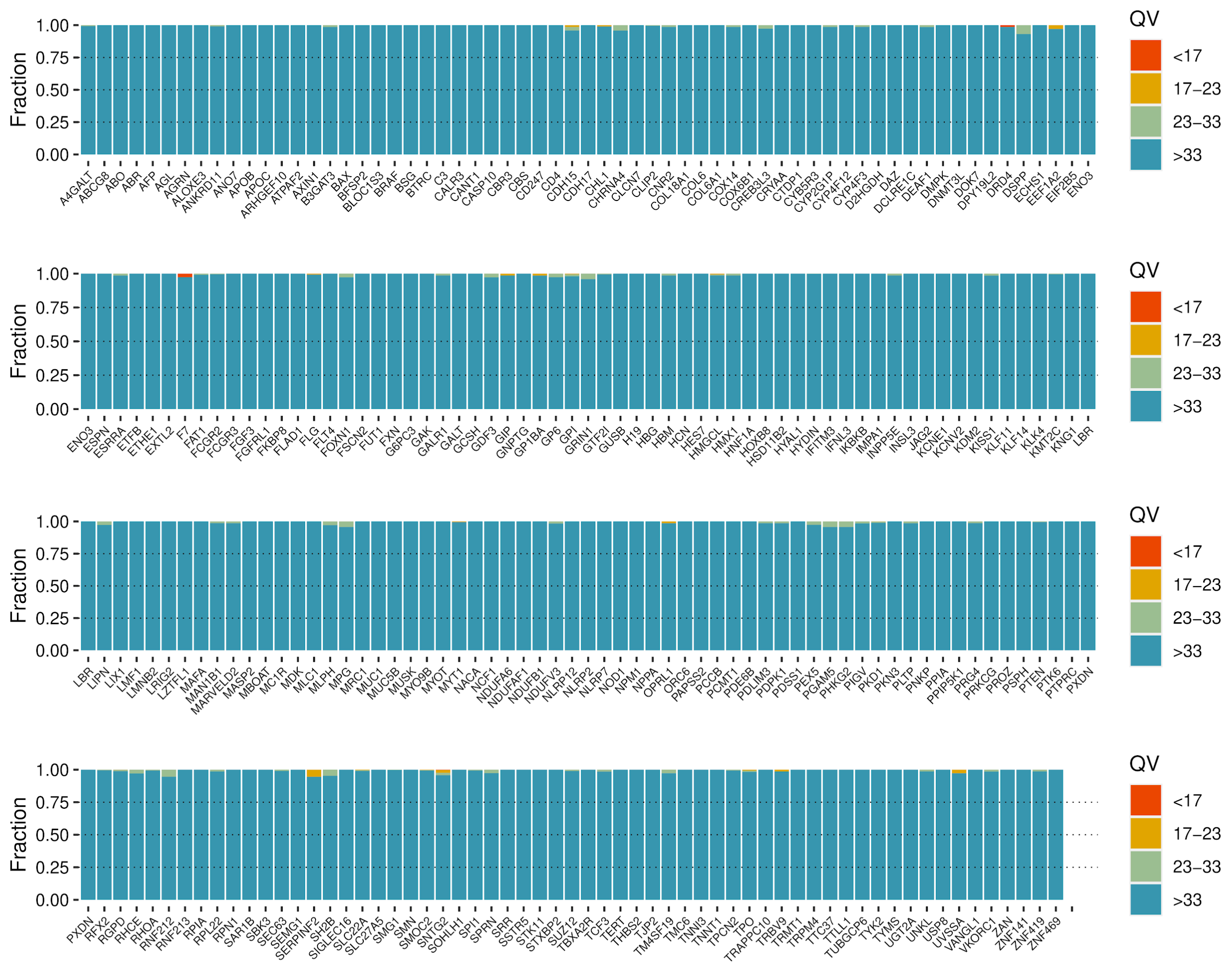

Supplementary Figure 4. Challenging medically relevant (CMR) genes benchmarked against the full genome database using stretcher alignment to ground truth, with accuracy measured on nonrepetitive sequences.

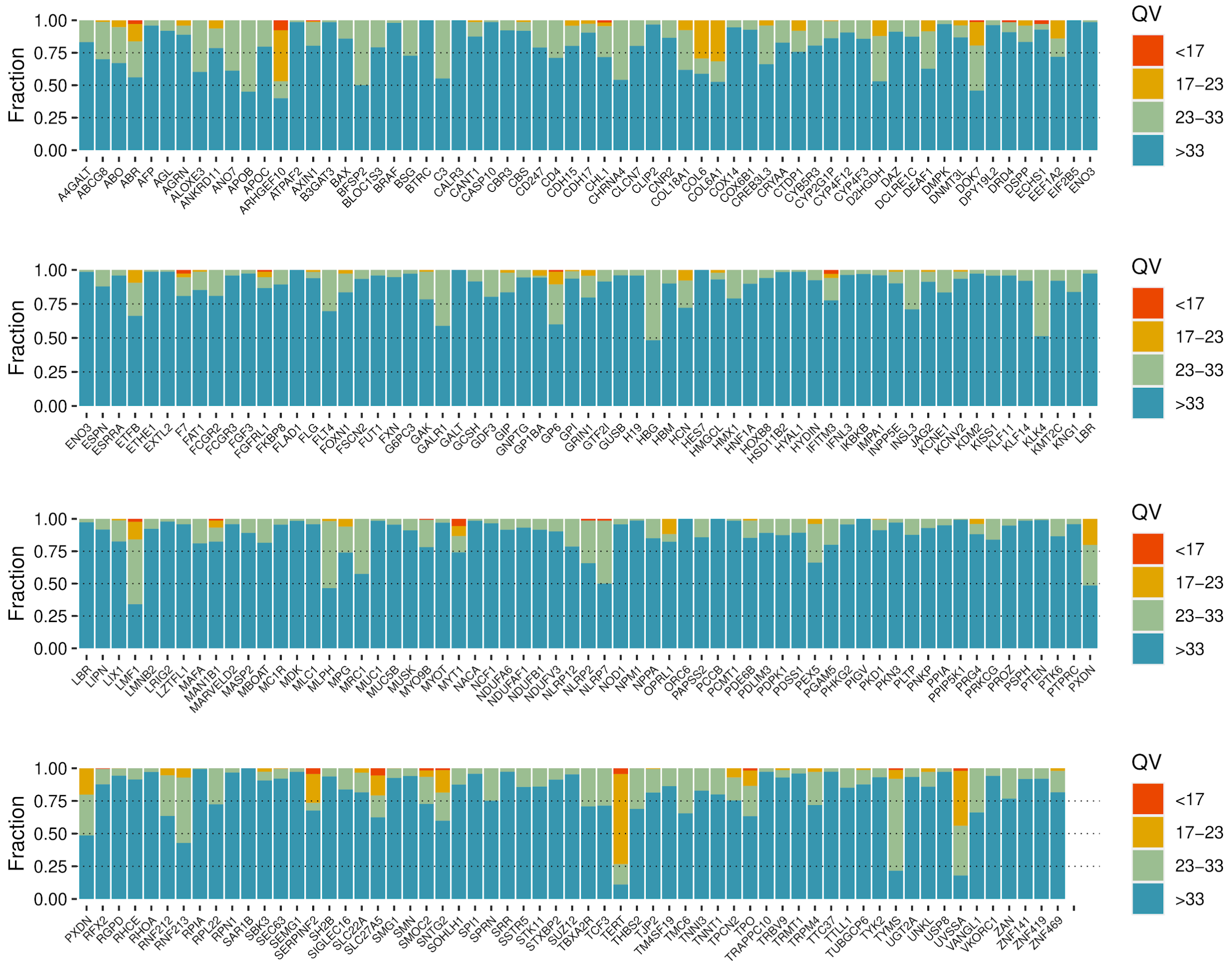

Supplementary Figure 5. CMR genes benchmarked using leave-one-out. Sequences were compared against ground truth using Stretcher, with accuracy measured on non-repetitive sequences.

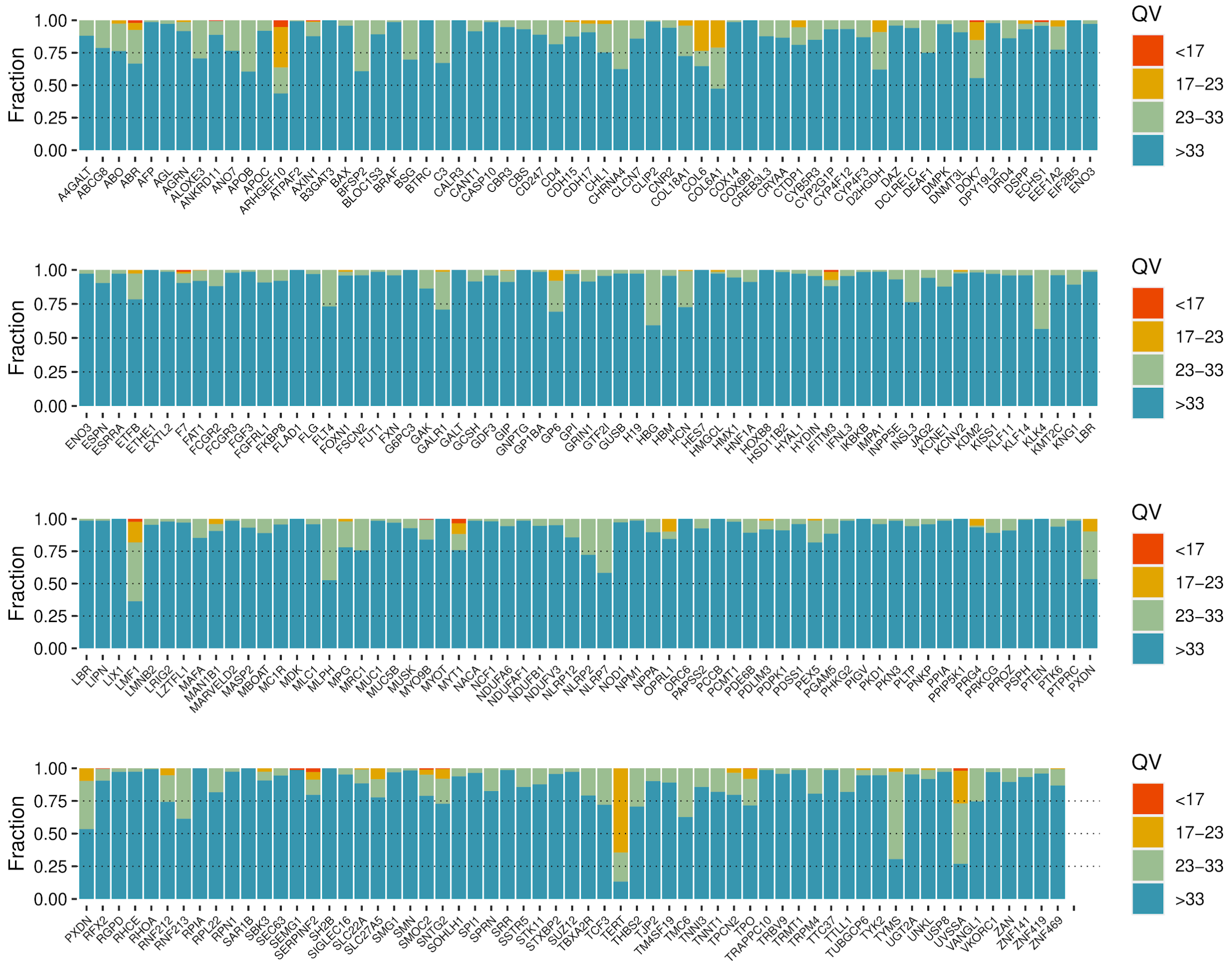

Supplementary Figure 6. Accuracy of the nearest-neighbor (determined by *k-mers*) in the pangenome to the ground truth for CMR genes. This reflects the sequence with the closest similarity to the ground truth, and the upper-bound for accuracy of leave-one-out tests.

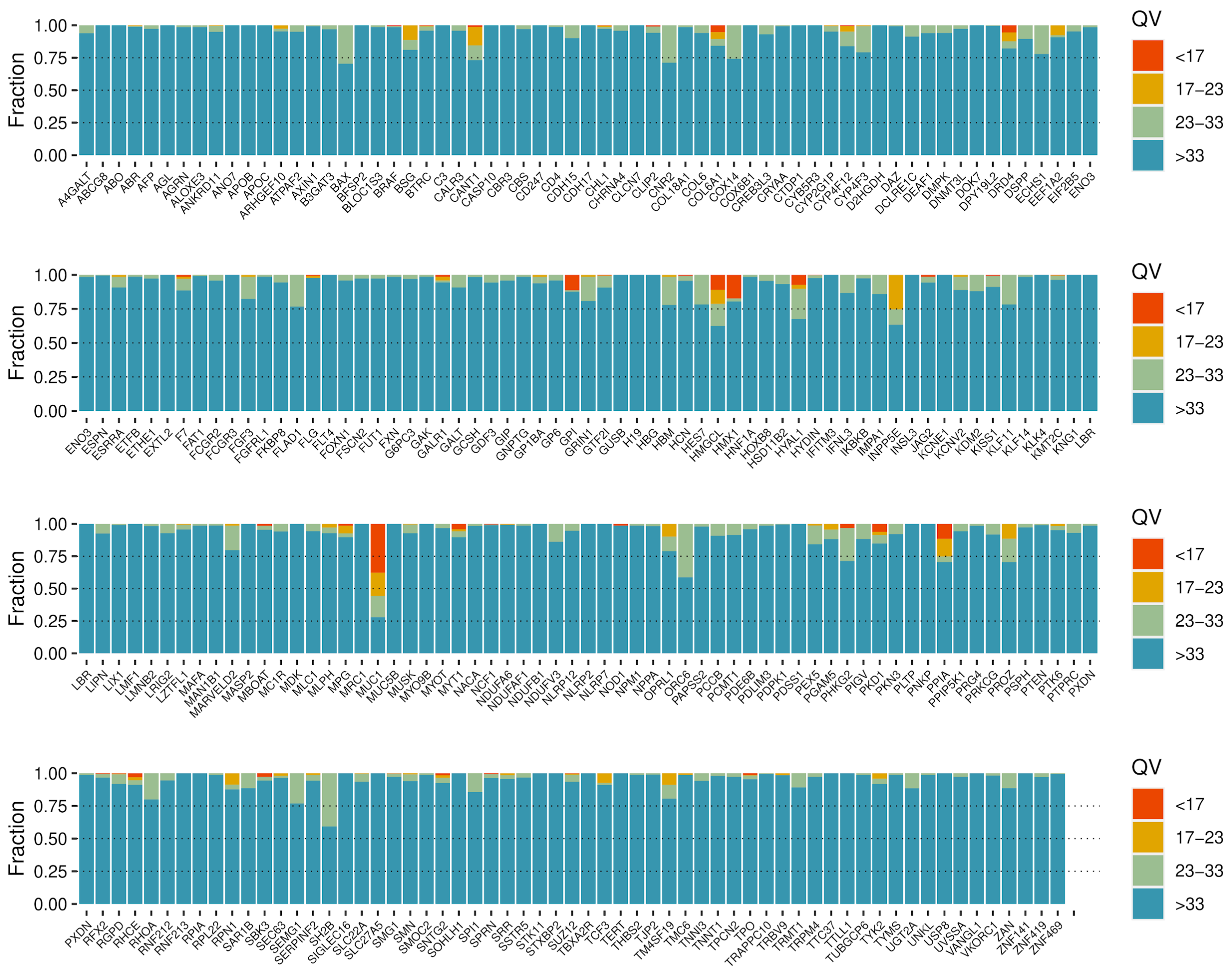

Supplementary Figure 7. Accuracy of ctyper genotyping on the full database including repetitive sequences.

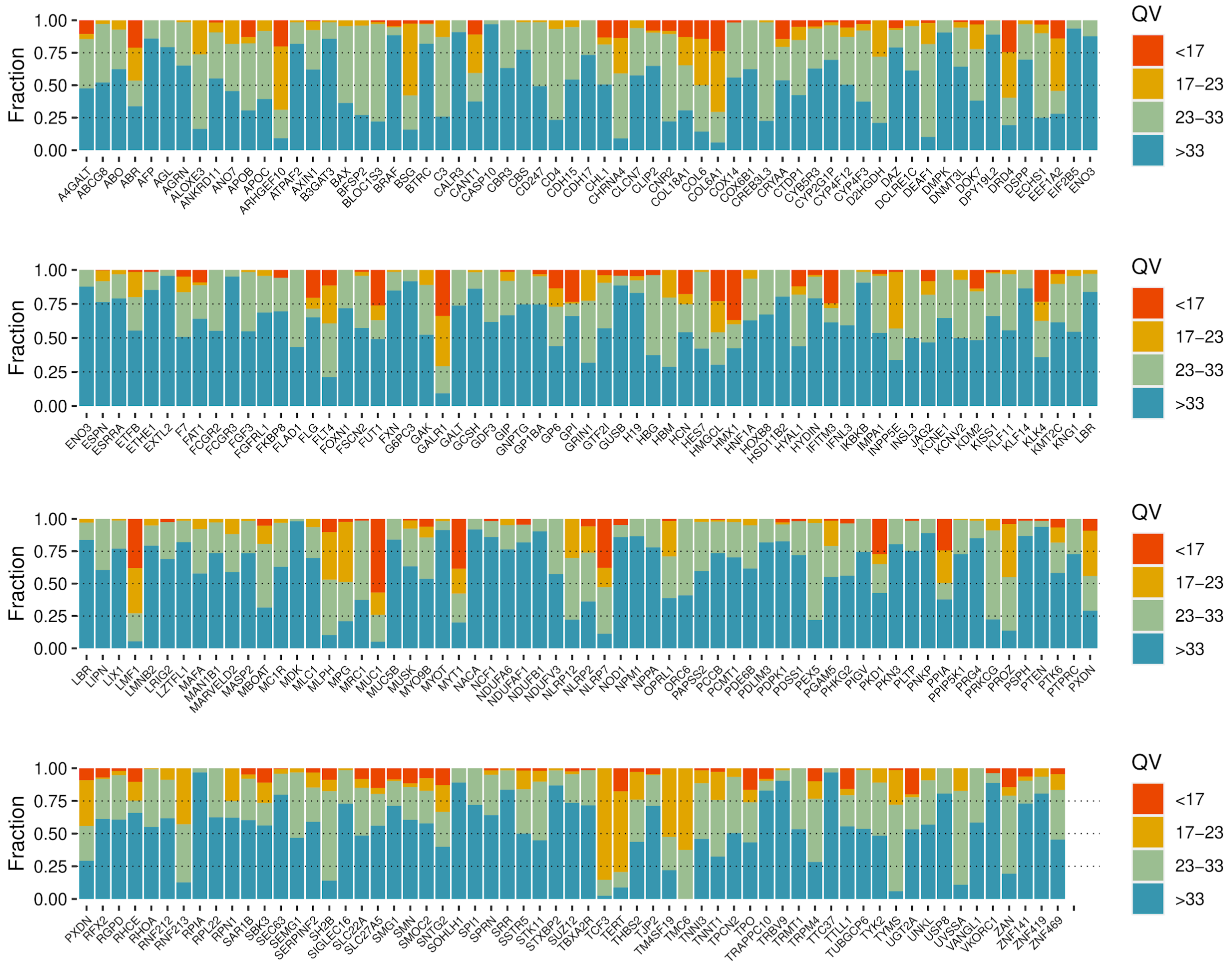

Supplementary Figure 8. Accuracy of ctyper genotyping on the leave-one-out database including repetitive sequences.

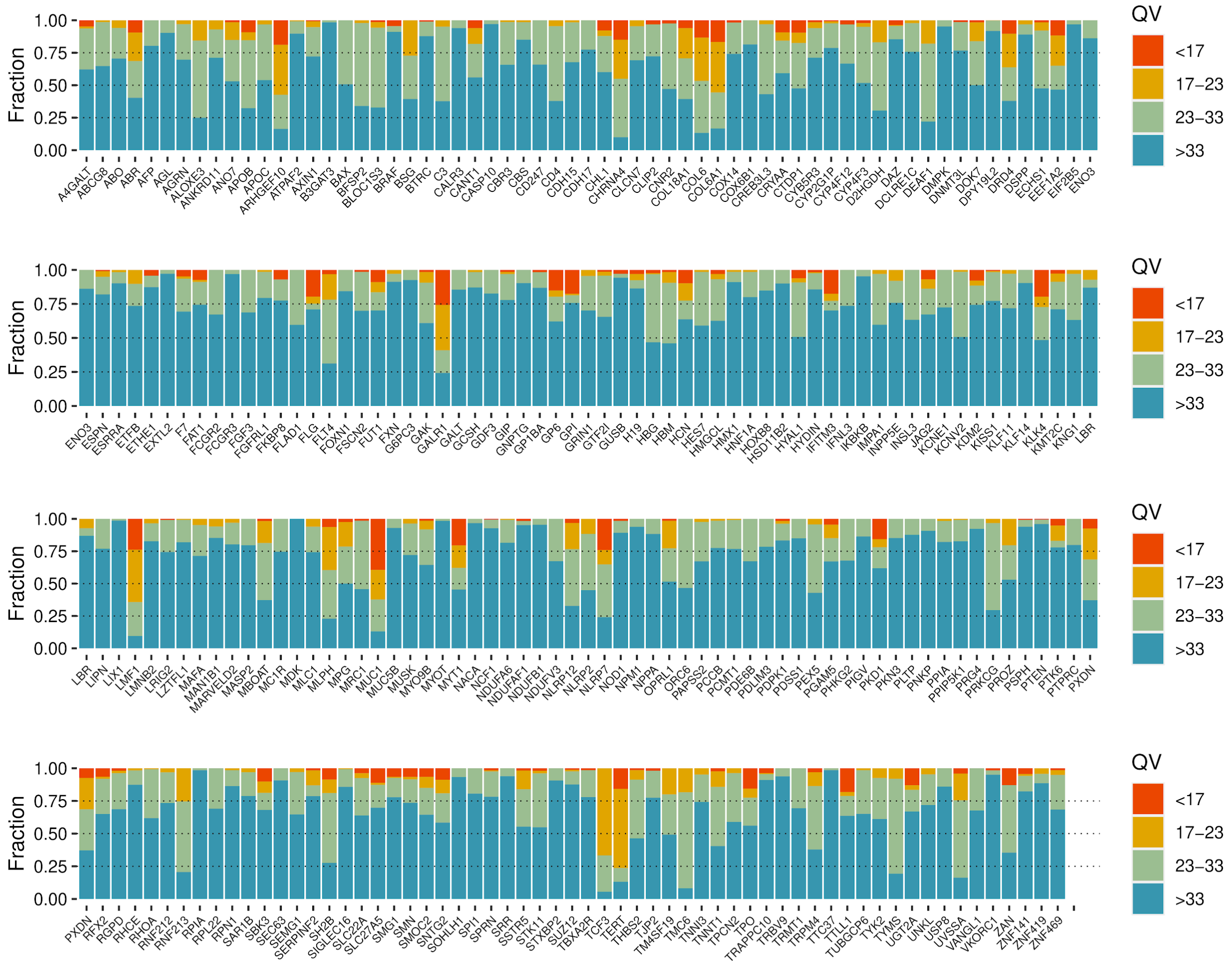

Supplementary Figure 9. Distance from the nearest neighbor (determined by *k-mers*) to the ground truth including repetitive sequences.

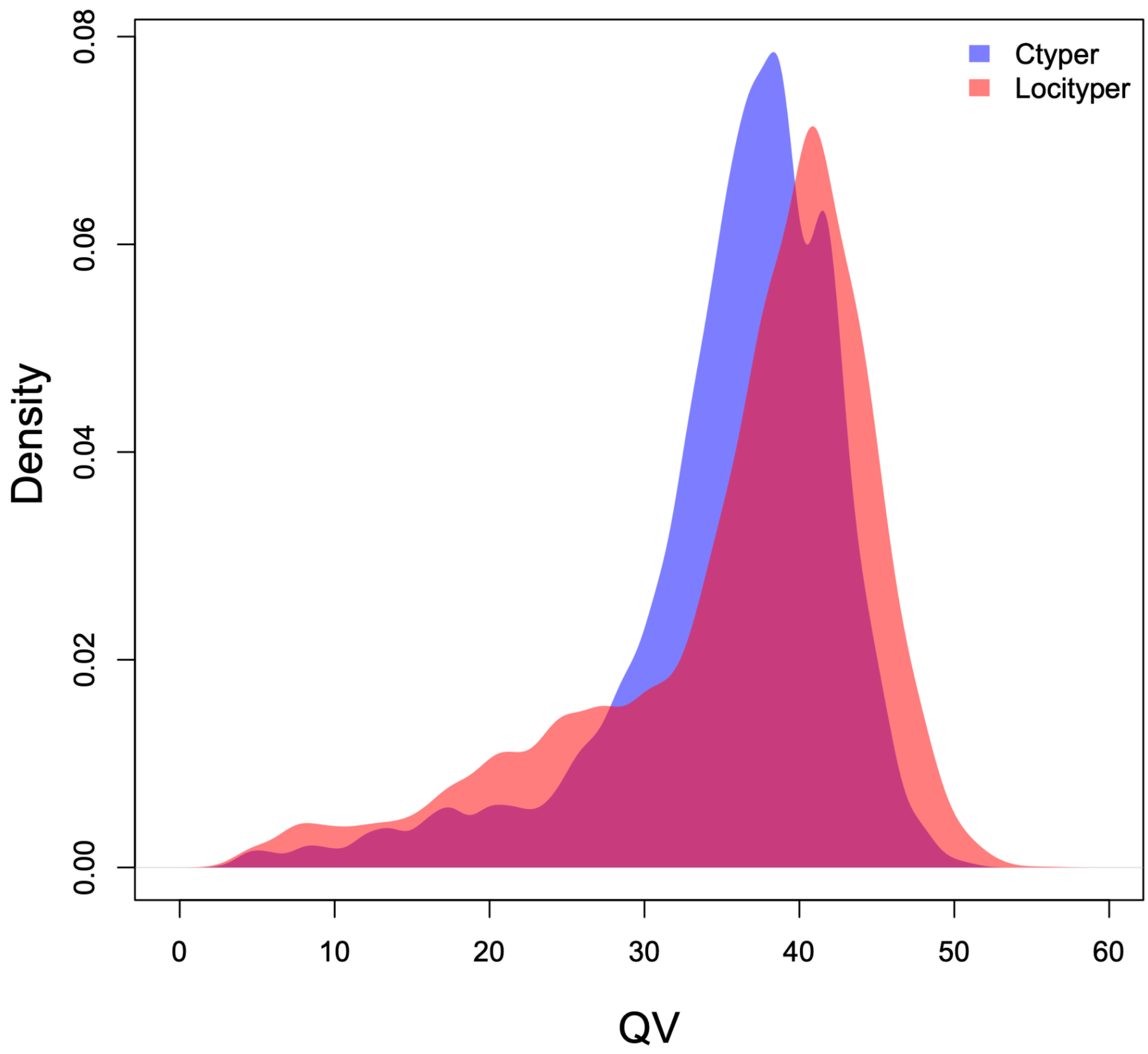

Supplementary Figure 10. Comparison of genotyping qualities between ctyper and Locityper on CMR genes. The genotyping results are based on the full-set (including original genes from assemblies of the same sample). The Locityper results were retrieved from https://zenodo.org/records/12607584. Both QV results were based on the aligner included in Locityper.

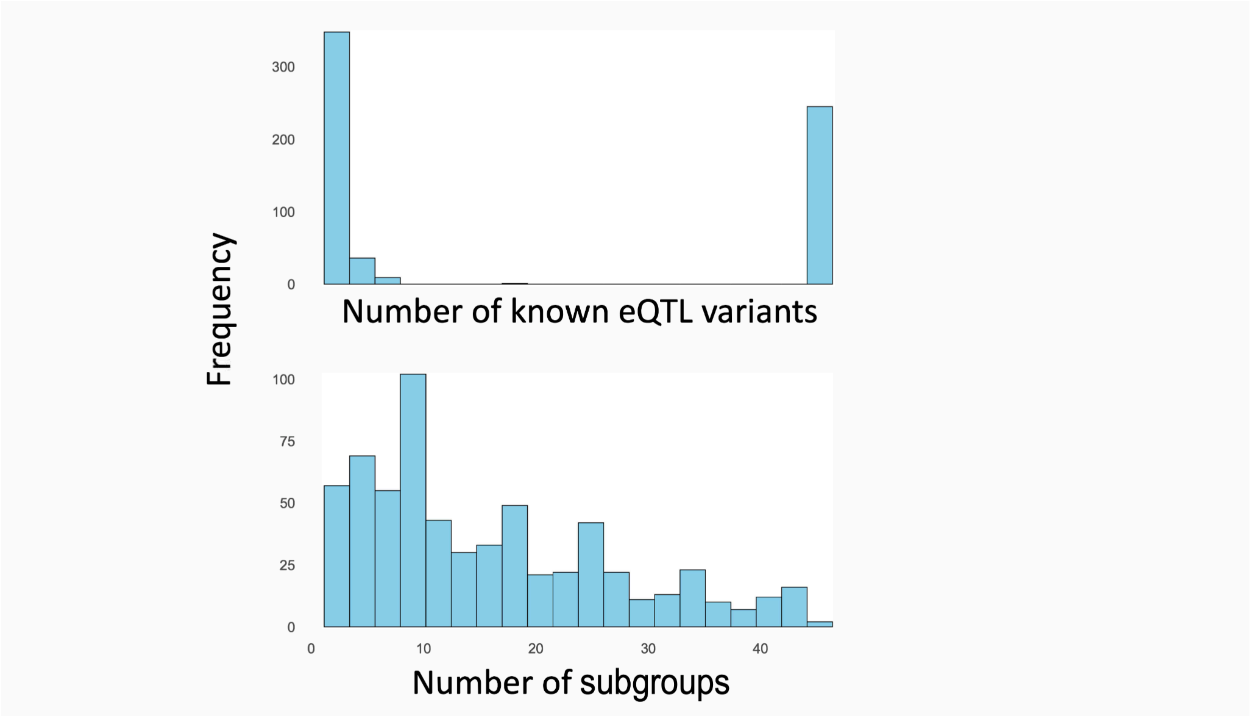

Supplementary Figure 11. The distribution of numbers of known eQTL variants (cis) and numbers of subgroups involved in gene expression. The values are based on 639 QTL transcripts used. The transcripts with more than 45 eQTL variants are capped at 45.
