# Supplementary material for: Genotyping sequence-resolved copy-number variations using pangenomes reveals paralog-specific global diversity and expression divergence of gene duplication
Source: bioRxiv. 2025 Feb 20:2024.08.11.607269. Originally published 2024 Aug 11. Preprint. [Version 5] doi: 10.1101/2024.08.11.607269 (PMC11326217; doi:10.1101/2024.08.11.607269)
Supplement: Supplementary file 2 [file media-2.pdf]

Analysis of the Relationship Between True Integer Results and Least-Error Non-Integer Solution

Contents

0.1 Formulation of genotyping 1
0.2 Initial non-integer solution 2
0.3 Analysis on precision of copy number 3
0.4 Analysis on precision of sequence based on tree 7
0.5 Analysis on the tolerance of sequencing uncertainty 15
0.6 Pseudo-code 18

0.1 Formulation of genotyping

Our genotyping is looking for a combination of known pangenome alleles to represent the genotypes of the query sample for each group of genes sharing homologies. Here, we represent this genotyping in mathematically way as:

$$X^* = \arg \min_{X^*, X_i^* \in \mathbb{Z}_{\geq 0}} \{\|X^* \times M - V\|\},$$

The following definitions are used,

X^*	$1 \times n$ vector, represents the combination of known pangenome alleles, and X_i^*, $X_i^* \in \mathbb{Z}_{\geq 0}$, represents the non-negative integer copy number of the i-th known allele.
M	An full rank $n \times m, m \gg n$ non-negative integer matrix, represents all known alleles in their k-mer form.
$M_{i,:}$	The i-th row of M, represents the i-th known allele.
$M_{:,j}$	The j-th column of M, represents the copy number of the j-th k-mer in each of the known alleles.
V	An $1 \times m$ vector, represents the observed counts of k-mers in M after normalization by sequencing coverage.
$(V_1, V_2, \ldots, V_n)$	Let V_i be $1 \times m$ vectors denoting the k-mer counts of individual alleles in the query sample, ignoring the uncertainty in the sequencing, then $V = \sum_{i=1}^{n} V_i$.

Thus, this solution is analogous to integer non-negative least squares regression, which is NP-hard for general solutions. However, we are showing we can have a good approximation with these constrains on M:

① This analysis assumes independent phylogenetic processes (e.g. perfect phylogeny), hence for any edge vectors E_i and E_j, $E_i * E_j = 0$.
This constraint is for the phylogenetic structure of alleles.
Gene conversions and recombinations might be an exception to this constrain. However, in practice, there is almost no "linearly perfect" conversion (such that $A + B \approx C + D$), thus those conversion events will show up close to the root of the phylogenetic tree and have little interference on the genotyping.

② A sufficient number of k-mer counts are not repetitive (e.g. < 2), hence for any allele's k-mer vector v such that v_j is the count of jth k-mer in this allele, we have set U_v to be the set of non-repetitive k-mers for allele v:
$U_v := \{j \in 1...m | v_j = 1, \text{ and } M_{i,j} \in \{0, 1\} \text{ for any i = 1...n}\}, |U_v| \not\ll \|v\|$.
This constraint ensures each row represents a single allele, instead of summations of multiple alleles.

③ For any allele in the query sample V_i, it shares high homology with at least one known allele in M, hence $\forall V_i, \min(\sin\langle V_i, M_{j,:}\rangle)_{j=1...m} \leq \epsilon$, where $\epsilon \ll 1$.
This constraint is to make sure we only genotype relevant genes. This constraint can hold true, considering humans are 99.9% genetically similar in general, and is more likely with increasing pangenome sizes.

④. The allele vector scales are not significantly different, satisfying $\frac{\|a\|}{\|b\|} \leq r$ for any pair of $a, b \in \{M_{i,:}, i = 1...n\} \cup \{v | \ v \text{ is any allele in query sample}\}$, where r is a finite value, $1 \leq r \ll \frac{1}{\sqrt[4]{max(\sin\langle a, b\rangle)}}$.
This constrain is make sure each row represents a single allele, instead of such as a small fragment or a single k-mer.

0.2 Initial non-integer solution

Considering first relax as a multivariable linear regression formulation, the solution minimizes the squared distance between observed and predicted values:

$$X = \arg\min_X \{\|X \times M - V\|\},$$

where X is the $1 \times n$ vector of copy numbers for each pangenome allele, V represents the observed $1 \times m$ k-mer counts from query sample, and M is the k-mer matrix acting as the coefficient matrix,

Since M is non-singular, the regression solution can be expressed as:

$$X = V \times M^+,$$

where M^+ is the $m \times n$ Moore–Penrose pseudoinverse of M.

Suppose M is an $n \times m$ matrix, where $m \gg n$ (with m representing number of k-mers and n representing number of alleles). Let $V_1, V_2, \ldots, V_n$ be $1 \times m$ vectors denoting the k-mer counts of individual alleles in the unknown NGS sample. Thus:

$$V = \sum_{i=1}^{n} V_i,$$

the total k-mer counts across all alleles. Substituting into the regression solution:

$$X = V \times M^+ = \sum_{i=1}^{n} \left(V_i \times M^+ \right).$$

This shows that the regression is additive, that is, the projection of the total counts V is the sum of the projections of the individual allele counts V_i.

By association, the regression result $X = V \times M^+ = \sum_{i=1..n}(V_i \times M^+)$. As a result, if the k-mer counts of any arbitrary allele v have high confidence to be projected to its correct genotype, the sum of all alleles V will also have high confidence to be projected to all correct genotypes without the need of phasing the reads. Hence, we only discuss the precision for this arbitrary individual allele vector v.

For an individual $1 \times m$ allele vector v, the projection is given by:

$$x = v \times M^+ = (x_1, x_2, \ldots, x_n),$$

where x_i is the projected coefficient for the i-th allele. By definition:

$$x = \arg\min_x \{\|x \times M - v\|\}$$

Expanding the projection using the rows M_i of M (where M_i denotes the k-mer counts for the i-th allele):

$$x \times M = \sum_{i=1}^{n} x_i \times M_i$$

0.3 Analysis on precision of copy number

Here, we analyze the bound of aggregate copy number to show that

$$1 - \sum_{i=1}^{n} x_i \ll 1, \text{Hence, } \sum_{i=1}^{n} x_i \approx 1$$

In this section, we define $R_a(b)$ as the rejection of $\mathbf{b}$ on $\mathbf{a}$, and we define $P_a(b)$ as the projection of $\mathbf{b}$ on $\mathbf{a}$.

Corollary 1. Under independent phylogenetic processes, the rejection of v does not counteract via summation. Hence, $\sum x_i \cdot \left\|R_v(M_i)\right\|^2 \leq \left\|\sum x_i \cdot R_v(M_i)\right\|^2$

Proof. Let Ω_i denote the set of edges of a phylogeny from the root to an arbitrary allele i corresponding to row M_i (each edge is a $1 \times m$ vector reflecting variants shared by descendent alleles; $M_i = \sum_{e \in \Omega_i} e$), Ω_j denote all phylogeny edges for another row M_j, and Ω_v denote the set of all edges for v in the phylogeny.

$$
\begin{aligned}
\left\|x_i \cdot R_v(M_i) + x_j \cdot R_v(M_j)\right\|^2 = & {x_i}^2 \sum_{e \in \Omega_i \setminus \Omega_j \setminus \Omega_v} \|e\|^2 \\
& + {x_j}^2 \sum_{e \in \Omega_j \setminus \Omega_i \setminus \Omega_v} \|e\|^2 \\
& + (x_i + x_j)^2 \sum_{e \in \Omega_j \cap \Omega_i \setminus \Omega_v} \|e\|^2 \\
& \geq \|x_i \cdot R_v(M_i)\|^2 + \|x_j \cdot R_v(M_j)\|^2
\end{aligned}
$$

Extending this to additional alleles:

$$
\sum x_i \cdot \left\|R_v(M_i)\right\|^2 \leq \left\|\sum x_i \cdot R_v(M_i)\right\|^2
$$

Let $M_{\min}$ represent the row of the closest neighbor of v in M, $M_{\min} = \arg\min_{M_i} (\sin\langle M_i, v\rangle)$, define $Sin_{min} := \sin\langle M_{\min}, v\rangle \leq \epsilon \ll 1$ and $R_{\min} := Sin_{min} \cdot \|M_{\min}\|$.

We then use Corollary 1 to analyze the rows with large rejection module, in order to show:

Corollary 2. The sum of coefficients of rows with large rejection to v are negligible, and $\sum_{\sin\langle M_i, v\rangle > \lambda \cdot Sin_{min}} x_i \ll 1$.

For rows with large rejection values (M_i satisfying $\sin\langle M_i, v\rangle \geq \lambda \cdot S_{min}$), we pick $\lambda = \frac{r}{\sqrt[4]{Sin_{min}}} \gg r^2 \geq r$, and $\ll \frac{1}{\sqrt{Sin_{min}}}$ (r is defined in Constraint 4), since $(x_1, x_2, \ldots, x_n)$ is the least-squares (LSE) solution, we show:

$$
\begin{aligned}
\sum_{\sin\langle M_i, v\rangle > \lambda \cdot S_{min}} x_i \cdot \|R_v(M_i)\|^2 \leq & \sum_{i=1}^{n} x_i \cdot \|R_v(M_i)\|^2 \\
& \leq R_{\min}, \\
& \text{because, } \|R_v(M_i)\| = \|M_i\| \cdot \sin\langle M_i, v\rangle \ \geq \frac{1}{r}\|M_{\min}\| \cdot \lambda S_{min} = \frac{\lambda}{r} R_{\min}.
\end{aligned}
$$

Thus:

$$\sum_{\sin\langle M_i,v\rangle > \lambda\cdot Sin_{min}} x_i \leq \frac{r}{\lambda} \ll 1 \quad \text{(negligible).}$$

Now we analyze the remaining rows with small rejections.

Corollary 3: Rows with small rejection has the projection to $v \approx \|v\|$. Hence, $\|P_v(M_i)\| \approx \|v\|$ as $\sin\langle M_i, v\rangle \leq \lambda \cdot Sin_{min}$.

Let S_i denote the set of all k-mers of M_i, and S_v denote the set of all k-mers of v:

Recall $U_v := \{j \in 1...m | v_j = 1$, and $M_{i,j} \in \{0, 1\}$ for any i = 1...n$\}$, the rejections on the columns corresponding to P:

$$\begin{aligned}
&R_v^2(M_i)\\
&\quad = \|M_i - P_v(M_i)\|^2\\
&\quad = \|M_i - \frac{\|M_i\| \cos\langle M_i, v\rangle}{\|v\|} v\|^2\\
&\quad = \sum_j^m (M_{i,j} - \frac{\|M_i\| \cos\langle M_i, v\rangle}{\|v\|} v_j)^2\\
&\quad \geq \sum_{j\in U_v}^m (M_{i,j} - \frac{\|M_i\| \cos\langle M_i, v\rangle}{\|v\|} v_j)^2\\
&\quad \geq \sum_{j\in U_v} (M_{i,j} - \frac{\|M_i\|}{\|v\|} v_j)^2 \text{ , Dividing } M_{i,j} \text{ into matching and mismatching components:}\\
&\quad \geq (1 - \frac{\|M_i\|}{\|v\|})^2 \cdot |U_v \cap \{j|M_{i,j} = 1\}| + (\frac{\|M_i\|}{\|v\|})^2 \cdot (|U_v \cap \{j|M_{i,j} = 0\}|)\\
&\quad \geq (1 - \frac{\|M_i\|}{\|v\|})^2 \cdot |U_v \cap \{j|M_{i,j} = 1\}| + (\frac{\|M_i\|}{\|v\|})^2 \cdot (|U_v| - |U_v \cap \{j|M_{i,j} = 1\}|)
\end{aligned}$$

Since $R_v^2(M_i) = \|M_i\|^2 \cdot \sin^2\langle M_i, v\rangle$, then for the second term on the right side:

$$\begin{aligned}
&(\frac{\|M_i\|}{\|v\|})^2 \cdot (|U_v| - |U_v \cap \{j|M_{i,j} = 1\}|) \leq \|M_i\|^2 \cdot \sin^2\langle M_i, v\rangle\\
&\iff |U_v| - |U_v \cap \{j|M_{i,j} = 1\}| \leq v^2 \cdot \sin^2\langle M_i, v\rangle \leq v^2\lambda^2 Sin_{min}^2\\
&\iff \frac{1}{v^2}|U_v \cap \{j|M_{i,j} = 1\}| \geq \frac{|U_v|}{v^2} - \lambda^2 Sin_{min}^2 \geq \frac{|U_v|}{v^2} - Sin_{min} \gtrapprox \frac{|U_v|}{v^2}
\end{aligned}$$

Apply this inequality for the first term on the right side:

$$
\begin{aligned}
&(1-\frac{\|M_i\|}{\|v\|})^2 \cdot |U_v \cap \{j|M_{i,j}=1\}| \leq R_v^2(M_i) \\
&\iff (\|v\|-\|M_i\|)^2 \cdot \frac{1}{v^2}|U_v \cap \{j|M_{i,j}=1\}| \leq R_v^2(M_i) \\
&\iff (\|v\|-\|M_i\|)^2 \lessapprox \frac{R_v^2(M_i)}{|U_v|}v^2 \ll v^2 \\
&\iff \|M_i\| \approx \|v\|
\end{aligned}
$$

Additionally,

$$
\sin\langle M_i, v\rangle \leq \lambda \cdot Sin_{min} \ll 1 \iff \cos\langle M_i, v\rangle \approx 1
$$

Hence, for the remaining rows with small rejections ($\sin\langle M_i, v\rangle \leq \lambda \cdot S_{min}$), we have:

$$
\|P_v(M_i)\| = \|M_i\| \cos\langle M_i, v\rangle \approx \|v\| \cos\langle M_i, v\rangle \approx \|v\|
$$

Corollary 4. The total projection of rows with small rejections on v is approximate to v. Hence, $\left\| P_v\big(\sum_{\sin\langle M_i,v\rangle \leq \lambda \cdot S_{min}} x_i \cdot M_i\big) - v \right\| \ll \|v\|$.

Proof. Since $(x_1, x_2, \ldots, x_n)^\top$ is the least-squares (LSE) solution:

$$
\big\|P_v(\sum_i^n x_i M_i - v)\big\| \leq R_{min}
$$

Splitting the left side to $\big\|P_v(\sum_{\sin\langle M_i,v\rangle \leq \lambda \cdot S_{min}} x_i M_i - v)\big\|$, and $\big\|P_v(\sum_{\sin\langle M_i,v\rangle > \lambda \cdot S_{min}} x_i M_i)\big\|$,

$$
\begin{aligned}
&\big\|P_v(\sum_{\sin\langle M_i,v\rangle \leq \lambda \cdot S_{min}} x_i M_i - v)\big\| \\
&\leq \big\|P_v(\sum_{\sin\langle M_i,v\rangle > \lambda \cdot S_{min}} x_i M_i)\big\| + \big\|P_v(\sum_i^n x_i M_i - v)\big\| \\
&\leq (\sum_{\sin\langle M_i,v\rangle > \lambda \cdot S_{min}} x_i)\big\|P_v(max(M_i))\big\| + R_{min} \\
&\leq (\sum_{\sin\langle M_i,v\rangle > \lambda \cdot S_{min}} x_i)\big\|P_v(max(M_i))\big\| + R_{min} \\
&\text{since } \big\|P_v(max(M_i))\big\| \leq \big\|max(M_i)\big\| \leq r\big\|v\big\|, \\
&\text{and } \sum_{\sin\langle M_i,v\rangle > \lambda \cdot S_{min}} x_i \leq \frac{r}{\lambda} \text{ from Corollary 2,} \\
&\leq \frac{r^2}{\lambda}\big\|v\big\| + R_{min} \\
&\ll \big\|v\big\|
\end{aligned}
$$

Corollary 5. The coefficients of rows with small rejections has the sum approximate to 1. Hence, $\sum_{\sin\langle M_i,v\rangle\leq\lambda\cdot S_{min}} x_i \approx 1$.

$$
\begin{aligned}
&\|P_v(\sum_{\sin\langle M_i,v\rangle\leq\lambda\cdot S_{min}} x_i M_i - v)\| = \|\sum_{\sin\langle M_i,v\rangle\leq\lambda\cdot S_{min}} x_i \frac{\|P_v(M_i)\|}{\|v\|} - 1\| \cdot \|v\| \ll \|v\| \\
&\iff \|\sum_{\sin\langle M_i,v\rangle\leq\lambda\cdot S_{min}} x_i \frac{\|P_v(M_i)\|}{\|v\|} - 1\| \ll 1 \\
&\iff \|\sum_{\sin\langle M_i,v\rangle\leq\lambda\cdot S_{min}} x_i \frac{\|P_v(M_i)\|}{\|v\|}\| \approx 1 \\
&\text{with Corollary 3, } \frac{\|P_v(M_i)\|}{\|v\|} \approx 1 \\
&\iff \|\sum_{\sin\langle M_i,v\rangle\leq\lambda\cdot S_{min}} x_i\| \approx 1
\end{aligned}
$$

Combining Corollary 2:

$$\left(\sum_{\sin\langle M_i,v\rangle>\lambda\cdot S_{min}} x_i + \sum_{\sin\langle M_i,v\rangle\leq\lambda\cdot S_{min}} x_i\right) \approx 1,$$

Hence:

$$\sum_{i=1}^{n} x_i \approx 1,$$

This shows that we have a good approximation of the correct number of alleles.

0.4 Analysis on precision of sequence based on tree

We analyze the precision on a binary phylogenetic tree. Using the closest neighbor location on tree as the true value, the precision on the tree is to show the discrepancy between the relative copy number we estimated from LSE regression and the true value, on each clade of the tree. Hence, on the clade C and its parent node N, the relative error: $|\frac{\sum_{i\in C} x_i}{\sum_i^n x_i} - t|$, where $t = 1$ if the closest neighbor of $v \in C$ else 0.

First, we demonstrate the fractality of the least square error solution on the phylogenetic tree. Hence:

Corollary 6: When x is the least square error regression of v on M. For any clade C of the phylogenetic tree, its corresponding coefficient $(x_i|i \in C)$ is also the least square error regression of $(\sum_{i\in C} x_i)v$ on submatrix $M'_{i\in C,:}$, hence regardless the rest of the tree, $\forall i \in C, x_i = k * x'_i$, where $k = \sum_{i\in C} x_i$, $x := \arg\min_X(\|XM - v\|)$, and $x' := \arg\min_X(\|XM_{C,:} - v\|)$. It means $\frac{(x_i|i\in C)}{\|(x_i|i\in C)\|}$ is a function of clade structure C.

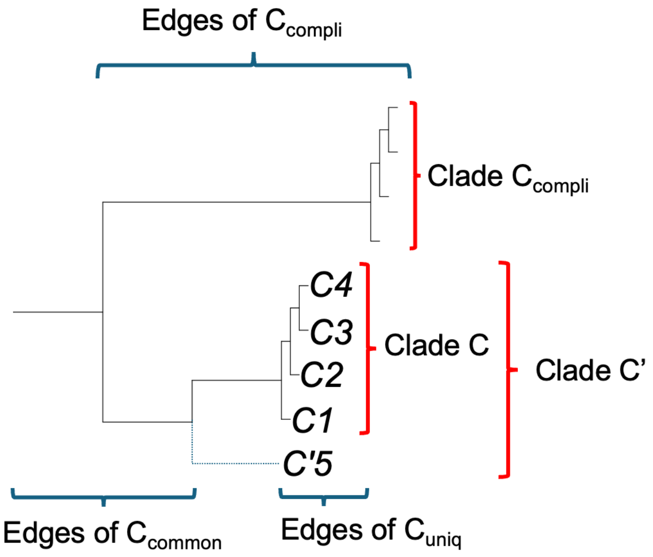

Figure 1: Diagram of the tree structure and three partitions of edges.

Define the following:

e	Denote any edge in the tree
$I_{i,e}$	Be 1 if e is on the path from the root to the ith allele of the tree, otherwise 0.
$R_a(b)$	The rejection of vector b on a.

Furthermore,

$$\text{x} := \arg\min_X(\|XM - v\|), \text{ the solution of the global LSE}$$

$$x' := \arg\min_X(\|XM' - v\|), \text{ the solution of the local LSE on only rows of clade } C$$

$$R := min(\|XM - v\|)_{X\geq 0}, \text{ the regression residual of the global LSE solution } x$$

$$R' := min(\|XM' - v\|)_{X\geq 0}, \text{ the regression residual of the local LSE solution } x'$$

We partition all edges of the tree into three parts as shown in Figure 1:

C_{common}	The common edges of all leaves in C (note: the edges of their common ancestor)
C_{uniq}	Other edges in of C.
C_{compli}	Complementary of C in T, representing edges only in other clades.

For the global LSE solution x, for residual R on each of three partitions of the tree, based on Corollary 1,

$$\begin{aligned} R^2 = & \sum_{t,e_t \in C_{common}} \|R_{e_t}(v)\|^2 \sum_i^n x_i I_{i,e_t} \\ & + \sum_{j,e_j \in C_{uniq}} \|R_{e_j}(v)\|^2 \sum_i^n x_i I_{i,e_j} \\ & + \sum_{k,e_k \in C_{compli}} \|R_{e_k}(v)\|^2 \sum_i^n I_{i,e_k} \end{aligned}$$

Because $I_{i,e_t} = 1, e_t \in C_{common}$ holds for alleles in C,

$$\begin{aligned} = & (\sum_{i,e_t \in C_{common}} x_i) * \|e_i\|^2 |C| \\ & + \sum_{j,e_j \in C_{uniq}} \|R_{e_j}(v)\|^2 \sum_i^n x_i I_{i,e_j} \\ & + \sum_{k,e_k \in C_{compli}} \|R_{e_k}(v)\|^2 \sum_i^n x_i I_{i,e_k} \end{aligned}$$

Also based on tree structure $I_{i,e_j} = 0, e_t \in C_{uniq}$ for any $i \notin C$ and conversely $I_{i,e_k} = 0, e_t \in C_{compli}$ for any $i \in C$,

$$\begin{aligned} = & (\sum_{i,e_t \in C_{common}} x_i) * \|e_i\|^2 |C| \\ & + \sum_{j,e_j \in C_{uniq}} \|R_{e_j}(v)\|^2 \sum_{i \in C} x_i I_{i,e_j} \\ & + \sum_{k,e_k \in C_{compli}} \|R_{e_k}(v)\|^2 \sum_{i \notin C} x_i I_{i,e_k} \end{aligned}$$

So, while fixing $(\sum_{i \in C} x_i)$,

$$\sum_{j,e_j \in C_{uniq}} \|R_{e_j}(v)\|^2 \sum_{i \in C} x_i I_{i,e_j} \text{ , and, } \sum_{k,e_k \in C_{compli}} \|R_{e_k}(v)\|^2 \sum_{i \notin C} x_i I_{i,e_k} \text{ are independent.}$$

Solve the 2nd and 3rd terms independently:

$$x = \arg\min_x(R) = \arg\min_x(\sum_{j,e_j \in C_{uniq}} \|R_{e_j}(v)\|^2 \sum_{i \in C} x_i I_{i,e_j}) \cap \arg\min_x(\sum_{k,e_k \in C_{compli}} \|R_{e_k}(v)\|^2 \sum_{i \notin C} x_i I_{i,e_k})$$

Similarly, for local solution residual R', which is not performed on C_{compli},

R'^2, based on Corollary 1,

$$= \sum_{t,e_t \in C_{common}} \|R_{e_t}(v)\|^2 \sum_{i \in C} x_i I_{i,e_t} + \sum_{j,e_j \in C_{uniq}} \|R_{e_j}(v)\|^2 \sum_{i \in C} x_i I_{i,e_j}$$

$$= (\sum_{i,e_t \in C_{common}} x_i) * \|e_i\|^2 |C| + \sum_{j,e_j \in C_{uniq}} \|R_{e_j}(v)\|^2 \sum_{i}^{n} x_i I_{i,e_j}$$

when fixing $\sum_{i \in C} x_i$,

$$x' = \arg\min_x(R') = \arg\min_x(\sum_{j,e_j \in C_{uniq}} \|R_{e_j}(v)\|^2 \sum_{i}^{n} x_i I_{i,e_j})$$

Hence, $R = \arg\min_x(R) \implies R' = \arg\min_x(R')$,

Corollary 7, With additional alleles in C, the total projection of v on C corresponding to the LSE solution, $\sum_{i \in C} x_i P_{M_i}(v)$, will increase, Hence, $C \subset C' \implies \sum_{i \in C'} x'_i P_{M_i}(v) \leq \sum_{i \in C} x_i P_{M_i}(v)$, where $x := \arg\min_x(\|XM_{C,:} - v\|)$, and $x' := \arg\min_x(\|XM_{C',:} - v\|)$.

We define :

$M' := M_{(C' \cup C_{compli}),:}$
$R^2_{M'}$:= the total regression of performing local LSE independently. Based on Corollary 6, $\min(R^2_{M'})$ is the global LSE solution.
$x := \arg\min_x(\|XM - v\|)$
$x' := \arg\min_x(\|XM' - v\|)$
$P_C := \sum_{i \in C} P_{M_i}(v) x_i$
$P_{C'} := \sum_{i \in C'} P_{M_i}(v) x'_i$
$R_{C'} := \sum_{i \in C'} R_{M_i}(v) x'_i$
$R_{C'_{compli}} := \sum_{i \in C_{compli}} R_{M_i}(v) x'_i$
$R_{v,C} := R_{M_{C,:}}(v),$ the residual of projecting v on $M_{C,:}$.
$R_{v,C'} := R_{M_{C',:}}(v),$ the residual of projecting v on $M_{C',:}$.
$R_{v,C_{compli}} := R_{M_{C_{compli},:}}(v),$ the residual of projecting v on $M_{C_{compli},:}$.

Based on Corollary 6, $R_{C'} : R_{v,C'} = P_{C'} : \|v\|, \implies$

$$R_{C'} = \frac{P_{C'}}{\|v\|} R_{v,C'} \text{ and similarly,}$$

$$R_{C'_{compli}} = \frac{\|v\| - P_{C'}}{\|v\|} R_{v,C_{compli}} \implies$$

$$R^2_{M'} = \frac{P^2_{C'}}{v^2} R^2_{v,C'} + \frac{(\|v\| - P_{C'})^2}{v^2} R^2_{v,C_{compli}} \implies$$

$$v^2 \frac{\partial R^2_{M'}}{\partial P^2_{C'}} = 2P^2_{C'} R^2_{v,C'} + 2(P_{C'} - \|v\|) R^2_{v,C_{compli}} \implies$$

when $R^2_{M'}$ is the minimum value, $P^2_{C'} = \frac{R^2_{v,C_{compli}}}{R^2_{v,C'} + R^2_{v,C_{compli}}} \|v\|,$

similarly, $P^2_C = \frac{R^2_{v,C_{compli}}}{R^2_{v,C} + R^2_{v,C_{compli}}} \|v\|$

because $C \subset C', R^2_{C'} \leq R^2_C,$ so,

$$P'_C = \frac{R^2_{C_{compli}}}{R^2_{C'} + R^2_{C_{compli}}} \|v\| \geq \frac{R^2_{C_{compli}}}{R^2_C + R^2_{C_{compli}}} \|v\| = P_C$$

Finally, we perform numerical analysis for the precision on any ancestor node N of v's closest neighbor u in M. We let $A' :=$ the child node of N that is either u or ancestor node of u. $B :=$ the another child node of N. Then the relative precision on A is $1 - \frac{\sum_{i \in A} x_i}{\sum_{i \in N} x_i}$.

Use Corollary 7, we relax the analysis on clades of only close neighbors of u, comparing it to other clades regarding the coefficients in x.

To simplify analysis, we normalize the scales of all rows of M to create a new matrix m, such $\forall i\,, R_v(m_i) = \|v\|$, and hence $m := diag(\frac{\|v\|}{P_v(M_i)}) \times M$. We use x and its derived symbols to denote the solutions on m, and y and its derive symbols to denote the solutions on M, we have $y = x \times diag(\frac{\|v\|}{P_v(M_i)})$. Based on Corollary 3, for close neighbors of v, we consider $\frac{\|v\|}{P_v(M_i)} \approx 1$, and $diag(\frac{\|v\|}{P_v(M_i)}) \approx I$, hence $y \approx x$. Thus we do not distinguish y and x in our following analysis.

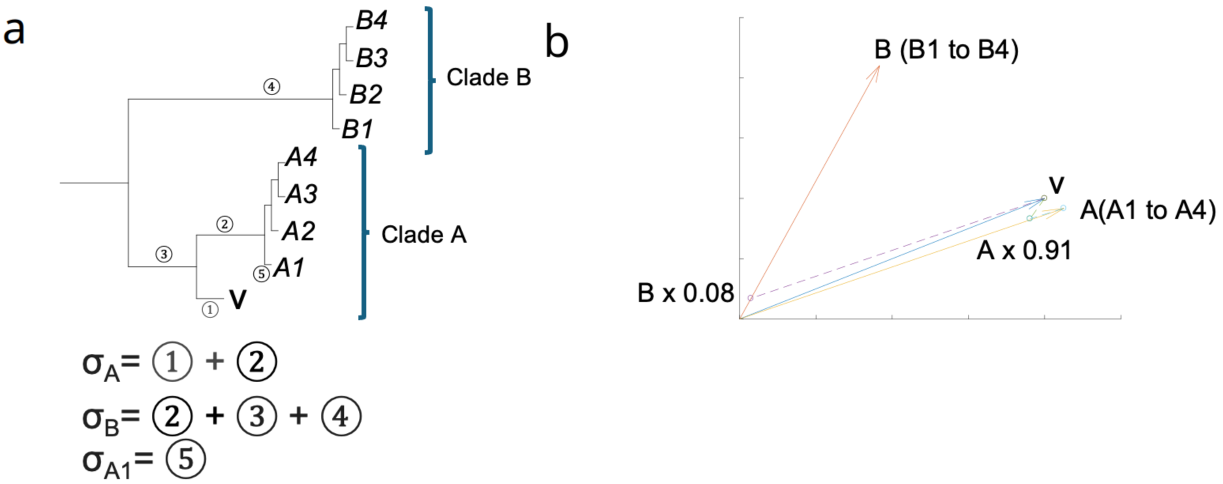

Figure 2: a. Tree structure of query allele v and two clades. b. The example projection of v on span$(A \cup B)$

The following definitions relate to the projection shown in Figure 2.

$S_A := \{x | x \in A, \text{and for for any another allele, } y, \text{ such that } R_v x * R_v y \approx 0 \ \}$
note: A only includes very similar alleles with v, only has rare variants and sequencing noise.
$S_B := \{i | m_i \text{ corresponds to all offspring of B}\}$
$A_i :=$ the index of vector of i th allele of S_A in m, and $i \in \{1...|S_A|\}$,
$B_i :=$ the index of vector of i th allele of S_B in m, and $i \in \{1...|S_B|\}$,
$m_A := m_{S_A,:}$, submatrix that index of rows correspond to S_A
$m_B := m_{S_B,:}$, submatrix that index of rows correspond to S_B
$m' := m_{S_A \cup S_B,:}$, submatrix that index of rows correspond to S_A and S_B
$\bar{m}_A := \frac{1}{|S_A|} \sum_{\mathbf{i} \in S_A} \mathbf{a}$, the mean of all m_{A_i}
$\bar{m}_B = \frac{1}{|S_B|} \sum_{\mathbf{i} \in S_B}, \mathbf{a}$, the mean of all m_{B_i}
$x_{A_i} := A_i$ th element of the LSE solution X
$x_{B_i} := B_i$ th element of the LSE solution X
$x_A := \sum_{i \in S_A} x_i$
$x_B := \sum_{i \in S_B} x_i$

The following distances (rejection) are defined:

$$\begin{aligned}
\text{Distance from } v \text{ to the mean of group } A: &\quad \sigma_A := \bar{m}_A - v, \\
\text{Distance from } v \text{ to the mean of group } B: &\quad \sigma_B := \bar{m}_B - v, \\
\text{Distance from } A_i \text{ to the mean of group } A: &\quad \sigma_{A_i} := m_{A_i} - \bar{m}_A, \\
\text{Distance from } B_i \text{ to the mean of group } B: &\quad \sigma_{B_i} := m_{B_i} - \bar{m}_B, \\
\text{Distance of common variants between groups, } A \text{ and } B,: &\quad \hat{\sigma}_B : \sigma_B - \sigma_A
\end{aligned}$$

Now, we analyze the precision of x, substituting these definitions into the projection optimization:

$$x = \arg\min_x \left\{ \left\| \sum_{A_i \in A} x_{A_i} \times (\sigma_{A_i} + \sigma_A) + \sum_{B_i \in B} x_{B_i} \times (\sigma_{B_i} + \sigma_B) \right\| \right\}.$$

Because $\sigma_A, \sigma_{A_1}, \ldots, \sigma_{A_n}, \sigma_{B_1}, \ldots, \sigma_{B_n}, \hat{\sigma}_B$ correspond to independent phylogenetic processes (non-recurrent mutations), they can be considered to be approximately orthogonal vectors ($\perp$) to each other. Thus, the optimization problem can be expressed as follows:

Continuing from the previous step, we have the following:

$$
\begin{aligned}
x &= \arg\min_x \left\{ \left\| \sum_{A_i \in A} x_{A_i} \cdot (\sigma_{A_i} + \sigma_A) + \sum_{B_i \in B} x_{B_i} \cdot (\sigma_{B_i} + \sigma_B) \right\| \right\} \\
&= \arg\min_x \left\{ \left\| \sum_{A_i \in A} \left(x_{A_i} \cdot \sigma_{A_i} \right) + \sum_{A_i \in A} \left(x_{A_i} \cdot \sigma_A \right) \right.\right. \\
&\quad \left.\left. + \sum_{B_i \in B} \left(x_{B_i} \cdot \sigma_{B_i} \right) + \sum_{B_i \in B} \left(x_{B_i} \cdot \hat{\sigma}_B \right) + \sum_{B_i \in B} \left(x_{B_i} \cdot \sigma_A \right) \right\| \right\} \\
&= \arg\min_x \left\{ \left\| \sum_{A_i \in A} \left(x_{A_i} \cdot \sigma_{A_i} \right) + \left(\sum_{A_i \in A} x_{A_i} + \sum_{B_i \in B} x_{B_i} \right) \cdot \sigma_A \right.\right. \\
&\quad \left.\left. + \sum_{B_i \in B} \left(x_{B_i} \cdot \sigma_{B_i} \right) + \sum_{B_i \in B} \left(x_{B_i} \cdot \hat{\sigma}_B \right) \right\| \right\}
\end{aligned}
$$

Substituting $\sum_{A_i \in A} x_{A_i}$ with x_A and $\sum_{B_i \in B} x_{B_i}$ with x_B, we can rewrite:

$$
\begin{aligned}
x = \arg\min_x \left\{ \left\| \sum_{A_i \in A} \left(x_{A_i} \cdot \sigma_{A_i} \right) + (x_A + x_B) \cdot \sigma_A \right.\right. \\
\left.\left. + \sum_{B_i \in B} \left(x_{B_i} \cdot \sigma_{B_i} \right) + x_B \cdot \hat{\sigma}_B \right\| \right\}
\end{aligned}
$$

Expanding and squaring norm terms, and using orthogonality, the squared norm becomes:

$$
\begin{aligned}
x = \arg\min_x \left\{ (x_A + x_B)^2 \cdot \|\sigma_A\|^2 + x_B^2 \cdot \|\hat{\sigma}_B\|^2 \right. \\
\left. + \sum_{A_i \in A} x_{A_i}^2 \cdot \|\sigma_{A_i}\|^2 + \sum_{B_i \in B} x_{B_i}^2 \cdot \|\sigma_{B_i}\|^2 \right\}
\end{aligned}
$$

Because $\|\sigma_{B_i}\|^2 \ll \|\hat{\sigma}_B\|^2$, the contributions from σ_{B_i} are negligible. Hence:

$$
\sum_{B_i \in B} \left(x_{B_i}^2 \cdot \|\sigma_{B_i}\|^2 \right) \ll \sum_{B_i \in B} \left(x_{B_i}^2 \cdot \|\hat{\sigma}_B\|^2 \right) \ll x_B^2 \cdot \|\hat{\sigma}_B\|^2.
$$

The optimization reduces to:

$$
\begin{aligned}
x &\approx \arg\min_x \left\{ (x_A + x_B)^2 \cdot \|\sigma_A\|^2 + x_B^2 \cdot \|\hat{\sigma}_B\|^2 + \sum_{A_i \in A} x_{A_i}^2 \cdot \|\sigma_{A_i}\|^2 \right\} \\
x &\approx \arg\min_x \left\{ x_B^2 \cdot \|\hat{\sigma}_B\|^2 + \sum_{A_i \in A} x_{A_i}^2 \cdot \|\sigma_{A_i}\|^2 \right\},
\end{aligned}
$$

By applying the Cauchy–Schwarz inequality, the solution satisfies:

$$x_{A_1} : x_{A_2} : \cdots : x_B \approx \frac{1}{\|\sigma_{A_1}\|} : \frac{1}{\|\sigma_{A_2}\|} : \cdots : \frac{1}{\|\hat{\sigma}_B\|}.$$

Thus, the ratio of contributions is:

$$x_A : x_B \approx \left(\sum_{A_i \in A} \frac{1}{\|\sigma_{A_i}\|} \right) : \frac{1}{\|\hat{\sigma}_B\|}.$$

Hence, the relative error is:

$$1 - \frac{x_A}{x_A + x_B} = \frac{1}{\frac{x_A}{x_B} + 1} \approx \frac{1}{\frac{\left(\sum_{A_i \in A} \frac{1}{\|\sigma_{A_i}\|}\right)}{\frac{1}{\|\hat{\sigma}_B\|}} + 1}.$$

For a sufficiently large and unbiased sample set, where all alleles follow phylogenetic relationships, we can consider that $\frac{1}{\|\hat{\sigma}_B\|}$ converges to a positive constant as the sample size increases, given that $\|\hat{\sigma}_B\|$ also converges to a positive constant.

In contrast, the term $\sum_{A_i \in A} \frac{1}{\|\sigma_{A_i}\|}$ diverges to positive infinity as the distance to the closest neighbor, $\min_{A_i \in A} \|\sigma_{A_i}\|$, approaches zero. Consequently, x_A will converge to 1, which represents the correct integer solution.

Use Corollary 7, we show the precision will not be worse when adding additional less close alleles in C. So for any ancestor node of C, its precision will not be worse than C.

This indicates that the model exhibits high precision in detecting known alleles that are phylogenetically close to the unknown allele with a sufficient database. Furthermore, the power of the model increases as the sample size grows, enabling better detection and accuracy with larger datasets.

In addition, this also shows when there is no uncertainty in the sequencing regarding counts of k-mers, the LSE solution X should be non-negative.

0.5 Analysis on the tolerance of sequencing uncertainty

To simplify this analysis, although exception exists, we assume the observation of all k-mers follows normal distribution (approximated from Poisson, hence variance equals the mean, and σ^2 is normalization factor based on sequencing coverage) and independent and identically distributed (iid). Thus $\hat{v}$, the observation of any $m \times 1$ vector v,

$$\hat{v} \overset{\approx}{\sim} \mathcal{N}(v, \sigma^2 \text{diag}(v))$$

Now we show for a large m, the uncertainty in the project can be ignored but unfortunately, the uncertainty in the rejection still needs to be considered.

$P_v(\hat{v}) = \frac{vv^T}{v^2}\hat{v},$

$\stackrel{\approx}{\sim} N\left(v, \sigma^2 \frac{vv^T}{v^2} \operatorname{diag}(v) \frac{v^T v}{v^2}\right),$

because $\sigma^2 \ll 1,$ and we consider most k-mers to be low in copy number, hence $max(v_i) \ll m$ and $\sigma^2 \operatorname{trace}\left(\frac{vv^T}{v^2} \operatorname{diag}(v) \frac{vv^T}{v^2}\right) \ll \sigma^2 trace(\operatorname{diag}(v)) \ll \|v\|,$

$\implies P_v(\hat{v}) \stackrel{p}{\approx} v.$

This means for our analysis on copy numbers still holds true if $1 \leq r \ll \frac{1}{\sqrt[4]{max(\sin\langle a,b\rangle) + max(R_v(\hat{v}), v=V_1 \ldots V_n)}}$ holds.

Now we move to rejection

$$
\begin{aligned}
R_v(\hat{v}) &= \hat{v} - P_v(\hat{v}) \\
&= \hat{v}(I - \frac{vv^T}{v^2}) \\
&\stackrel{\approx}{\sim} N\left(0, \sigma^2 (I - \frac{vv^T}{v^2}) \operatorname{diag}(v) (I - \frac{v^T v}{v^2})\right) \\
&\implies E(R_v^2(\hat{v})) \approx \sigma^2[trace(diag(v)) + trace(\frac{vv^T}{v^2} \operatorname{diag}(v) \frac{v^T v}{v^2}) - 2 * trace(\frac{vv^T}{v^2} \operatorname{diag}(v))]
\end{aligned}
$$

Consider most k-mers to be low in copy number, $\ll m$, thus,

$trace(\frac{vv^T}{v^2} \operatorname{diag}(v))$ and

$trace(\frac{vv^T}{v^2} \operatorname{diag}(v) \frac{v^T v}{v^2}) \ll trace(\operatorname{diag}(v)),$

so, $E(R_v^2(\hat{v})) \approx \sigma^2 trace(\operatorname{diag}(v))$

Thus, considering the degrees of freedom in m are large,

$R_v^2 \stackrel{\approx}{\sim} \frac{\sigma^2 trace(diag(v))}{m} \chi^2(m)$

$\stackrel{\approx}{\sim} N\left(\sigma^2 trace(diag(v)), 2\sigma^2 trace(diag(v))\right)$

Using the Delta method on first order derivative,

$\frac{Var(\|R_v\|)}{Var(\|R_v\|^2)} \approx (\frac{dE(\|R_v\|)}{dE(\|R_v\|^2)})^2,$ so, $Var(\|R_v\|) \approx \frac{1}{2}$

$\|R_v\| \stackrel{\approx}{\sim} N\left(\sigma\sqrt{trace(diag(v))}, \frac{1}{2}\right)$, consider $\frac{1}{2} \ll \sigma\sqrt{trace(diag(v))}$

$\stackrel{p}{\approx} \sigma\sqrt{trace(diag(v))}$

This means the uncertainties in sequencing will cause non-negligible residuals in regression. Now we demonstrate its interference on the genotyping precision.

Because of Corollary 6, we restrict our analysis on the edges in either clade A or B, which also makes $x_A : x_B$ to be the LSE solution.

We give these definitions:

$M'_{A,:}$ the derive of M_A with columns for k-mers only in clade $A \cup B$

$M'_{B,:}$ the derive of M_B with columns for k-mers only in clade $A \cup B$

$\sigma_{A,seq} := \hat{v}$ with columns for k-mers only in clade σ_A

Recall our reasoning in Corollary 7 and numeric analysis, without uncertainty in sequencing, we have

$$x_A : x_B \approx \frac{1}{\|R_A(v)\|} : \frac{1}{\|R_B(v)\|} \approx \left(\frac{1}{\frac{1}{\sum_{A_i \in A} \frac{1}{\|\sigma_{A_i}\|}}} \right) : \left(\frac{1}{\|\hat{\sigma}_B\|} \right)$$

Now we refine with uncertainty in sequencing of v. We consider sequencing error does not "create" any k-mers that does not exists, we have:

$$\|R_{M'_{B,:}}(v')\| = \|R_{M_{B,:}}(v)\|$$
$$\|R_{M'_{A,:}}(v')\| = \sqrt{R^2_{M_{A,:}}(v) + R^2_{\sigma_A}(\sigma_{A,seq})}$$

From the above, we have, $\|R_{\sigma_A}\| \stackrel{\approx}{\sim} N\left(\sigma \cdot \sqrt{trace(diag(\sigma_A))}, \frac{1}{2}\right) \| \stackrel{p}{\approx} \sigma \cdot \sqrt{trace(diag(\sigma_A))}$

and $trace(diag(\sigma_A))$ is the total number of k-mers v has, which should be relative to σ_A^2, let it to be $k \cdot \sigma_A^2$, where $k = \frac{trace(diag(\sigma_A))}{\sigma_A^2}$. When most k-mers are either 0 or 1, $k \approx 1$, most k-mers are 0 or 2, $k \approx \frac{1}{2}$.

So when consider uncertainty in sequencing, the updated the ratio of coefficients is.

$$E(x_A : x_B) \approx \left(\frac{1}{\sqrt{R^2_{\sigma_A} + (\frac{1}{\sum_{A_i \in A} \frac{1}{\|\sigma_{A_i}\|}})^2}} \right) : \left(\frac{1}{\sqrt{R^2_{\sigma_A} + \hat{\sigma}^2_B}} \right)$$

it is natural to consider $\frac{1}{\sum_{A_i \in A} \frac{1}{\|\sigma_{A_i}\|}} \ll \|R_{\sigma_A}\|$

$$\approx \left(\frac{1}{\|R_{\sigma_A}\|} \right) : \left(\frac{1}{\sqrt{R^2_{\sigma_A} + \hat{\sigma}^2_B}} \right)$$

if also to consider $\|R_{\sigma_A}\| \ll \|\hat{\sigma}_B\|$,

$$x_B : x_A \approxeq N \left(\frac{\sqrt{k}\sigma\|\sigma_A\|}{\|\hat{\sigma}_B\|}, \frac{1}{2\|\hat{\sigma}_B\|} \right)$$

$$\overset{p}{\approx} \frac{\|\sigma_A\|}{\|\hat{\sigma}_B\|}\sqrt{k}\sigma$$

At a high level, this could be interpreted as residuals from NGS sequencing vs residuals from population diversity.

So when the standard deviation of a single k-mer σ is small, $x_A : x_B$ will act like no uncertainty, but unfortunately at high σ, this model will lose precision. When there are only a single or very few alleles, since in general this does not change the size comparison relationship of x_A, x_B, it might not affect the rounding results. However, when there are many copies of alleles in the query samples, x_B might exceed 0.5 and cause rounding error. Thus correctly genotyping high copy number gene families does require sufficient coverage to reduce σ. Fortunately, via performing independent copy number estimation (rounding) for each k-mer, σ can be largely reduced, which we implemented. Moreover, k decreases as gene copy number increases, which sort of reduces this high copy number noise from $O(n)$ to $O(\sqrt{n})$, where n is the allele copy number.

In the most common simple situation, where $\sigma_A \approx \hat{\sigma}_B$, most k-mers are 0 or 1 in each allele and $\sigma \approx \sqrt{P_{\text{WrongRounding}}(1 - P_{\text{WrongRounding}})} \approx \sqrt{\phi(2.5)(1 - \phi(2.5))} \approx 0.1$, where ϕ is cumulative density function of normal distribution, after for standard 30 coverage NGS after performing independent rounding for k-mers, the model ideally can maintain precision for $k < 25$, and having about 1 error for $k < 100$. This implies this model in the ideal situation works on almost all high-copy number genes.

0.6 Pseudo-code

Based on the previous mathematical reasoning, we can conclude an algorithm:

Algorithm 1: Naive recursive function to round coefficients to the most likely closest neighbors, starting from the root of the tree.

```
Function RoundingtoNeighbor(node):
    Input  : node
    Output: node
    if node has no child then
        return node;
    LeftChild ← RoundingtoNeighbor(node.LeftChild)    // Recursive to the
     left child RightChild ← RoundingtoNeighbor(node.RightChild)
     // Recursive to the right child
    if abs(LeftChild.remainder) > abs(RightChild.remainder)) then
        LeftChild.remainder += RightChild.remainder;
        LeftChild.round += ⌊LeftChild.remainder + 0.5⌋;
        LeftChild.remainder −= ⌊LeftChild.remainder + 0.5⌋;
        return LeftChild;
    else
        RightChild.remainder += LeftChild.remainder;
        RightChild.round += ⌊RightChild.remainder + 0.5⌋;
        RightChild.remainder −= ⌊RightChild.remainder + 0.5⌋;
        return RightChild;
    end
```

However, real-world data often deviate from ideal assumptions (e.g., perfect phylogeny, complete independence of k-mers). Based on our previous mathematical reasoning, we apply the following optimizations:

1. We perform discretization to reduce variance by performing rounding for each k-mer. By central limit theorem, this shall not cause bias and our reasoning still holds true, but with reduced overall σ.

2. Non-negative least-squares (NNLS) rounding. Given the uncertainty in predicting k-mer copy numbers, a non-negative solution minimizes overall error. Hence, we use an NNLS approach for rounding the coefficients. Unfortunately, this is heuristic and it is still unknown to us why initial solutions often have negative terms. One hypothesis could be the k-mers can not really independent at even large scale due to library preparations. Because this is heuristic, we do not predict it overlap impact, but since it bounds the range of the solution, this is at least a safer choice.

3. Projections instead of simple summations. As shown in Corollary 4, the total projections on query genes also approximates the query genes. Furthermore, using projections helps reducing the impact of varying allele sizes. This is more robust than merely summing

coefficients, and can improve copy number call.

4. Choosing the minimal global residual. Rather than selecting the clade with the larger coefficient, we pick the one yielding the smaller global residual. This minimizes the impact of imperfect solution of NNLS, which is not a deterministic solution. This can improve the sensitivity in genotyping.

These optimizations result in this following updated pseudo-code:

Algorithm 2: Updated recursive function based on optimizations.

```
Function RoundingtoNeighbor(node, residual):
    Input  : node, residual
    Output: Updated node, residual
    if node has no child then
        return node, residual;
    LeftChild, residual ← RoundingtoNeighbor(node.LeftChild, residual)
     // Recurse on the left child
     RightChild, residual ← RoundingtoNeighbor(node.RightChild, residual)
     // Recurse on the right child
    Left_to_Right ← Proj(LeftChild, RightChild, RightChild.remainder)
     // Project left remainder to the right
     Right_to_Left ← Proj(RightChild, LeftChild, LeftChild.remainder)
     // Project right remainder to the left
    Left_to_Right_residual ← newResid(RightChild, Left_to_Right, residual)
     // Global residual for left-to-right projection
     Right_to_Left_residual ← newResid(LeftChild, Right_to_Left, residual)
     // Global residual for right-to-left projection
    if (Left_to_Right_residual)^2 < (Right_to_Left_residual)^2 then
        LeftChild.remainder += Left_to_Right;
        LeftChild.round += ⌊LeftChild.remainder + 0.5⌋;
        LeftChild.remainder −= ⌊LeftChild.remainder + 0.5⌋;
        return LeftChild, Left_to_Right_residual;
    else
        RightChild.remainder += Right_to_Left;
        RightChild.round += ⌊RightChild.remainder + 0.5⌋;
        RightChild.remainder −= ⌊RightChild.remainder + 0.5⌋;
        return RightChild, Right_to_Left_residual;
    end
Function newResid(node, increment, residual):
    Input  : node, increment, residual
    Output: Updated residual
    new_residual ← residual − (increment × node.vector);
    return new_residual;
Function Proj(child1, child2, coef):
    Input  : child1 node, child2 node, coefficient
    Output: Projected value
    project ← child1.vector × child2.vector^T × child2.vector//(child2.vector)^2;
    return coef × project;
```
