# Supplementary material for: Genotyping sequence-resolved copy-number variations using pangenomes reveals paralog-specific global diversity and expression divergence of duplicated genes
Source: bioRxiv. 2024 Oct 15:2024.08.11.607269. Originally published 2024 Aug 11. Preprint. [Version 3] doi: 10.1101/2024.08.11.607269 (PMC11326217; doi:10.1101/2024.08.11.607269)
Supplement: Supplementary file 1 [file media-1.pdf]

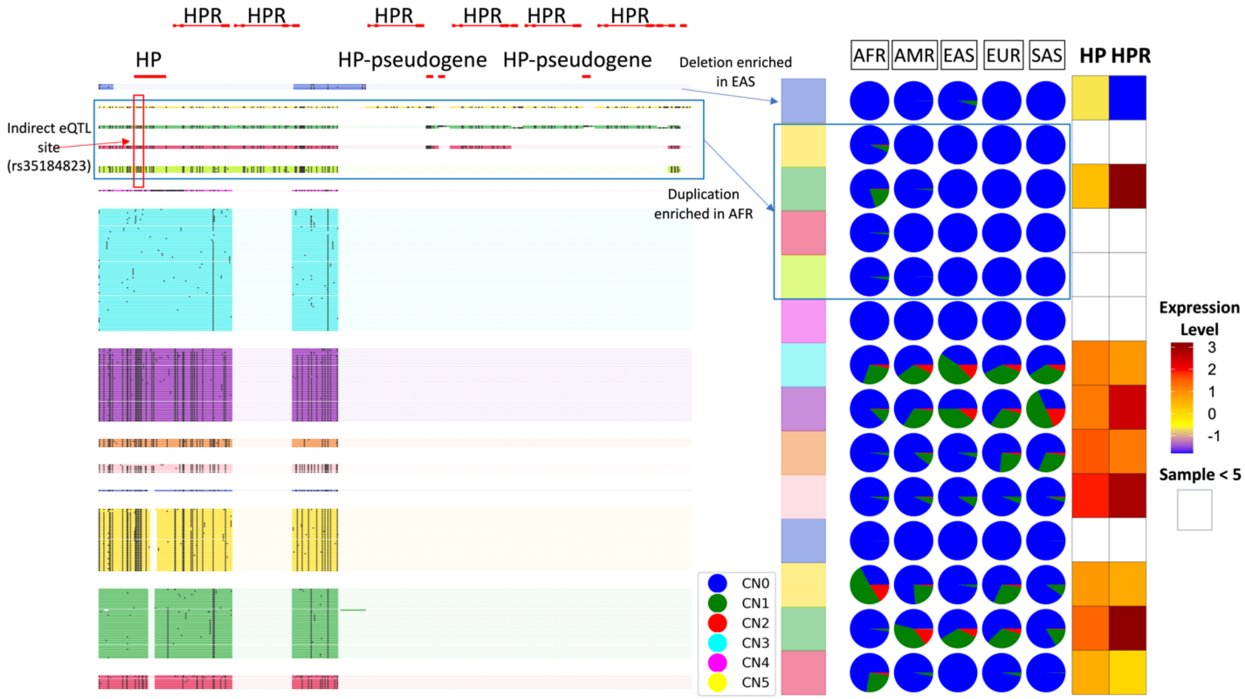

Supplementary Figure 1. Comprehensive case study on *HPR* and *HP* genes. Left: the mutant plot for all PAs on the *HPR* and *HP* locus based on graphic MSA; Proximal duplication and a SNP associated with proximal duplications are annotated (rs35184823, reported by GETx). Middle: the genotyping results based on 2504 unrelated 1kpg samples. Each pie chart represents the copy number distribution of each allele-type in each population. Right: the expression level for each allele-type estimated from the genotyping results of GETx samples and published GETx whole blood TPM, using linear-mixed-model. The allele-types found in less than 5 samples are excluded. Co-expression has been found between *HPR* and *HP* (t = 24.089, p-value = 6.2e-78, pearson correlation).

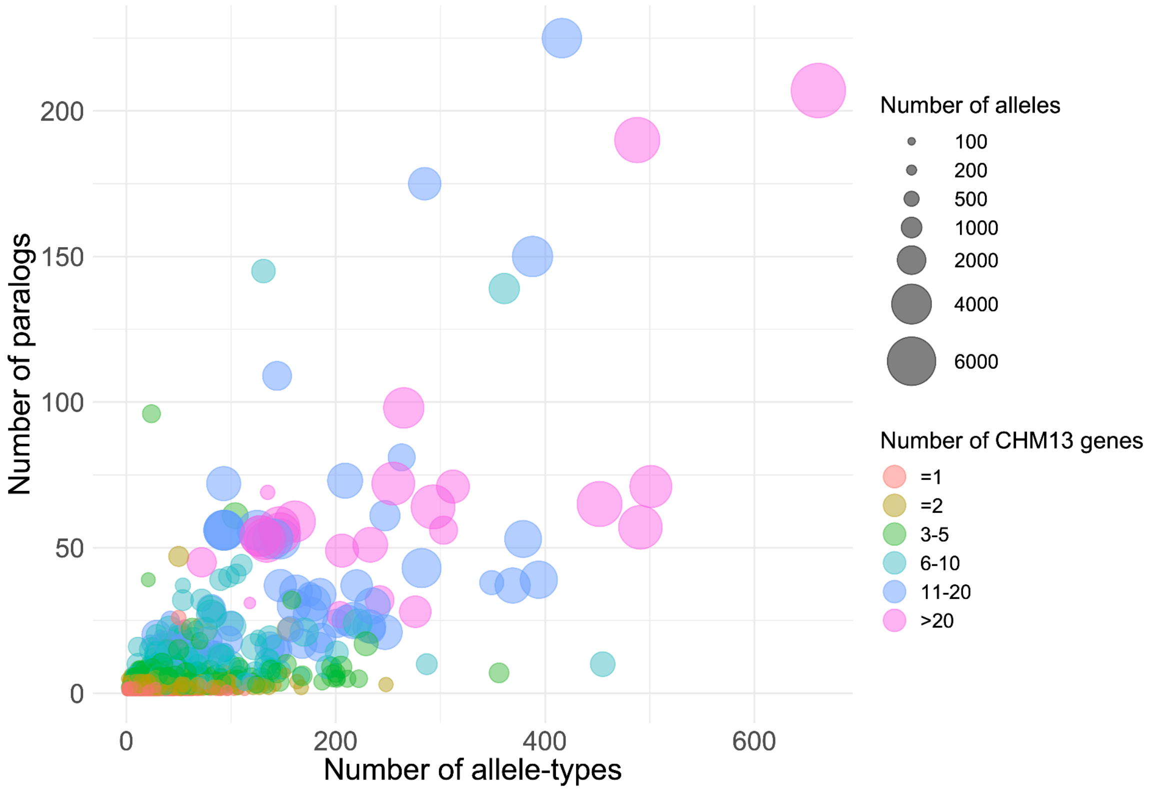

Supplementary Figure 2. The characteristics of all gene groups. Each dot represents each of 3,307 gene-groups. The correlation has been found between number of alleles and number of allele-types (t=54.6, p-value = 1e-179), but stronger between number of paralogs and number of allele-types (t=72.9, p-value = 1e-219).

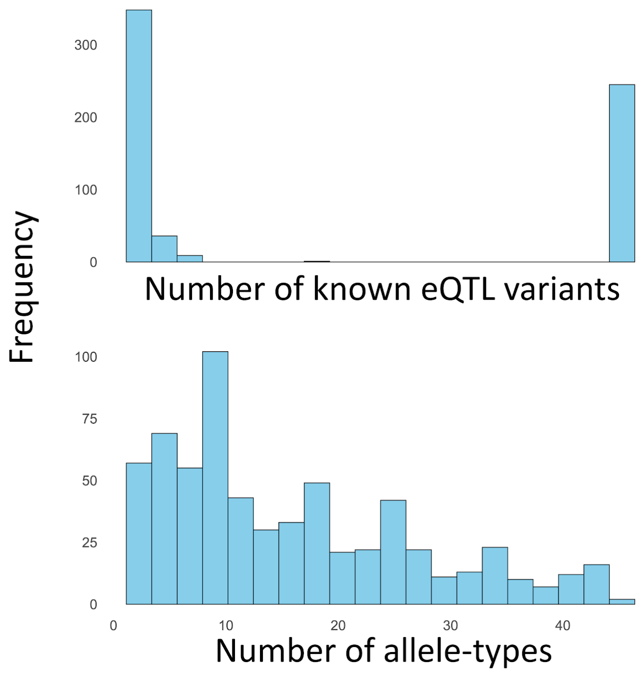

Supplementary Figure 3. The distribution of numbers of known eQTL variants (cis) and numbers of allele-type involved in gene expression. The values are based on 639 QTL units used. The units with more than 45 eQTL variants are capped at 45.

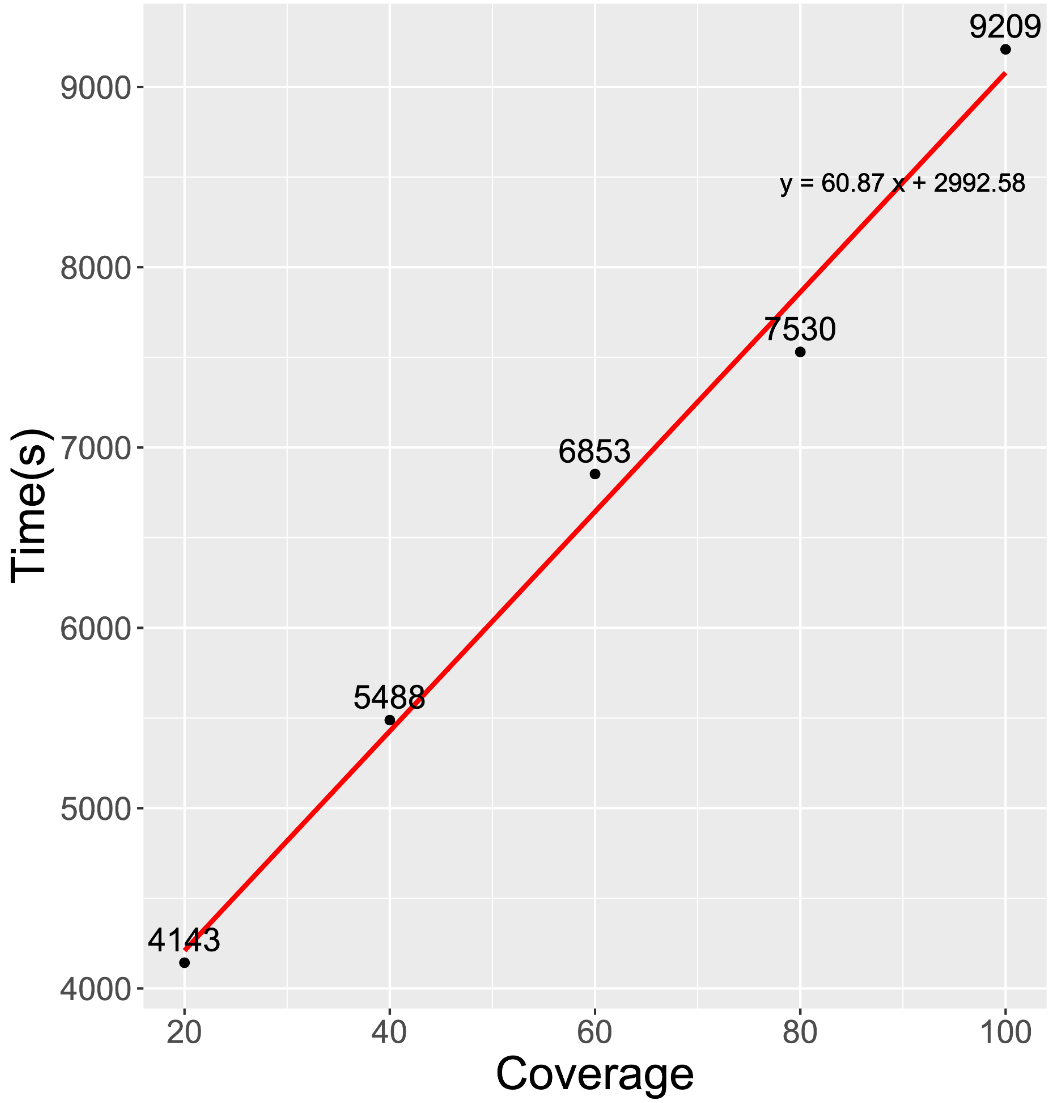

Supplementary Figure 4. The time cost of ctyper on NGS data at different coverages. The same NGS data was downsampled into different coverages and converted to standard cram files. We ran all coverages independently with one thread CPU (AMD EPYC 7513 32-Core Processor) and 16G RAM. We also provide a multiple thread function to let Ctyper be able to process multiple files in parallel using shared memory.

Supplementary methods

1 Construction and visualization of graphic multiple sequence alignment

Representing rearrangement, tandem duplication, and translocation events using progressive multiple-sequence alignment is challenging and computationally intensive, particularly for large sets of sequences due to the $O(N^2)$ number of pairwise alignments. To address these challenges, we developed a method that constructs a pseudo multiple-sequence alignment using graph-based alignment, which can be achieved with linear complexity.

Unlike traditional genomic graphs, in this graph alignment each vertex represents a structural variation breakpoint and each edge represents the sequence segment between these breakpoints. On each edge, we merge a collection of sequence segments from the query sequences that have high sequence identity and no large (>300 bp) structural variants. With all segments represented or aligned on edges, each query sequence corresponds to a path within this graph.

1.1 Finding the minimum sufficient set of segments to represent all sequences

Given a list of query sequences, we define a collection of segments as a reference set if all query sequences can be fully covered by combinations of local alignments on this collection, provided the alignments have high identity (95%). We call this collection of segments as the pan-gene sequence graph.

The first step in forming a graph-based multiple sequence alignment is to identify a reference set of minimal size. This process begins by initializing the reference set with the largest allele from GRCh38. Subsequently, the remaining sequences are iteratively locally aligned to the reference set. Local alignments with a similarity greater than 95%, spanning at least 300 bases, and containing a minimum of 100 shared k-mer are considered valid. Any portion of the query sequences exceeding 300 bases that lacks a valid alignment is deemed uncovered and is added to the reference set.

After assembling the reference set, we post-process it to identify and exclude internal repeats longer than 300 bases and with 95% identity. Specifically, regions within the reference set that align to any upstream location or prior sequences are removed to prevent redundancy.

1.2 Identifying alignment breakpoints on query sequences to construct pan-gene alignment graph

We then align each query sequence to the reference set to identify structural variant breakpoints. Sequences are mapped to the reference set, retaining alignments over 300 bases and 95% identity. The starting and ending points of alignments are recorded on each reference set sequence, and breakpoints are defined as clusters of alignment endpoints within 50 bps.

The vertices of the graph are then defined by the sequences' endpoints and an enumeration of breakpoints. Edges are established based on the reference set segments between corresponding endpoints or breakpoints of the vertices. Consequently, each query sequence correspond to a path in the graph, which can then be used as a template to align the query sequence with high identity and no structural variation. We refer to this as a sequence path.

We construct a directed graph using all sequence paths, along with a start node (S) that connects the start points of all sequence paths, and an end node (E) that connects the end points of all sequence paths. This graph is referred to as the pan-gene alignment graph.

1.3 Linearization of the pan-gene alignment graph

First, in order to represent the repetitive sequences in the graph, if a sequence path contains multiple visits of a same node (cycle), we will create new nodes for the redundant visits. For example, the second occurrence of the breakpoint $B1$ on any query sequence will be renamed to $B1_2$.

We then record all breakpoints and aim to sort them into a list that has the best overall agreement with the orders in all sequence paths.

1. Starting from the start node S, we propagate to its most frequent unvisited successor across all sequence paths.

2. We keep propagating and record every new node we visit to the list at the location right after the last visited node, which is important when we re-visit the same node again.
   If all successors of a node have already been visited, the propagation moves to the end node E, if connected, or returns to the last visited node.

3. When we reach the end node, we re-propagate it to start node and repeat the cycle until all node has been visited.

The resulting list determines the order of linearization. Using this ordered list of nodes, we identify the corresponding edges (if any) and arrange the associated segments in the determined order. We then assemble these segments to create a template, referred to as the global linear template.

1.4 Pairwise global alignments to determine and visualize small variants

To enhance alignment quality, we re-align each query sequence to the pan-gene linear template. We extract the segments from each query sequence that mapped to the template and assembled them into what we refer to as a local linear template. Global pairwise alignments are then performed between each sequence and its corresponding local linear template. These alignments are subsequently transferred back to the pan-gene linear template. For segments of sequences that are not included to the local linear template, gaps are introduced.

We then assemble all pairwise alignments onto the pan-gene linear template, ensuring that sequence lengths were uniform at locations with insertions or deletions (indels). The result is a graphic multiple sequence alignment (MSA).

Variants within each sequence were readily identified and annotated based on the alignments. These variants can be visualized as a mutant plot, providing a clear and informative overview of genetic diversity, including both structural variations and small variants, across different sequences.

2 *k*-mer counting, normalization and bias correction in NGS data

During genotyping of NGS samples, we count all k-mers in each gene group's k-mer list. Specifically, we encode 31-mers as 64-bit integers for hashing purposes. We selected approximately one million random unique k-mers from exons of protein-coding genes to establish background sequencing coverage, which we refer as background k-mers. The average copy numbers of those background k-mers is determined as the k-mers coverage. We normalize all copy numbers of k-mers by this coverage to obtained the initial k-mers vectors of NGS samples.

We found that certain genomic regions, such as the *HLA-DRB* gene—previously identified as the most problematic genes in other study[3]—exhibited strong batch effects. The bias overall increases the coverage of NGS data in this region and might potentially cause false positive calling of duplications and false negative of detecting deletions.

To address this, we provide a function for bias correction. We benchmarked individual k-mer in NGS data against long-read assemblies of the same sample, which served as the golden standard for true copy number determination. To identify k-mer with the lowest confidence, we calculated a posterior p-value for each k-mer' occurrence based on the Poisson distribution of ground-truth copy numbers in each NGS datasets. The k-mer with more than 15% of samples (6 out of 40) exhibiting a p-value below 0.05 were flagged. We assessed the average ratio discrepancies between ground-truth

and observed copy numbers across 40 NGS samples, applying ratio corrections to the k-mer with low confidence.

The bias correction we performed largely reduced the Hardy Weinberg disequilibrium of *HLA-DRB* genotyping, from 34.3% (48 out of 140) to no Hardy-Weinberg disequilibrium.

After background normalization and bias correction, a k-mer vector V is obtained, denoting the most likely integer copy numbers of all k-mer in the original genome.

However, the underlying cause of this bias remains to be investigated, and we observed this bias in both cell-line and non-cell line NGS samples.

3 Detection of novel partial duplication and deletion below the scale of pangenome-alleles

To detect novel partial gene duplications and deletions that have not been previously observed in the pangenome cohorts and are of much smaller scale than pangenome alleles, we designed an auxiliary function in ctyper that utilizes k-mer coverages.

Recall that all pangenome alleles (PAs) are included in the graphic MSAs by the minimum sufficient sets of segments. We annotated the k-mer that locate at exclusive positions (i.e., k-mer found only in one segment and at a single position on that segment) and record these in our database.

After completing genotyping, we calculate the coverage ratio for each k-mer using the genotyping solution. The coverage ratio is defined as the ratio between the expected k-mer count from genotyping and the observed k-mer count in NGS data. We average the coverage ratios within each window on each segment (default window size = 60). This coverage can be further averaged over larger scales; we recommend using a scale of at least 3K.

These coverage patterns can also be visualized as a dot plot, which allows us to identify and visualize fragmented gene duplication and deletion events.

However, due to the rarity and difficulty of detecting novel events, benchmarking this results is challenging. It is also often difficult to distinguish real events from misassemblies, making comprehensive benchmarking infeasible. Nonetheless, it is estimated to reduce discrepancies the genotyping results from *de novo* assembly (CN1) by 7% for events larger than 3K.

4 Two step alignments for better lift-over of CNV genes

Our phylogenetic-based annotation and classification relied on sequence similarities among different pangenome alleles and were independent of their specific genomic locations. However, understanding these locations was important for distinguishing between paralogs and orthologs, especially in cases involving highly mutated sequences.

While the Human Pangenome Reference Consortium (HPRC) and the Human Genome Structural Variation Consortium (HGSVC) had previously conducted their own lift-over processes and analyses, these efforts may not be sufficient for accurate ortholog identification. The limitation was largely due to the fact that copy number variable genes often reside in segmental duplication regions, which are susceptible to structural variations and repetitive sequences. Alignments on these genes often result in fragmented alignments and multiple high-confidence alignments, presenting significant challenges for conventional sequence aligners.

To overcome the challenge, we developed a highly sensitive, multi-step alignment pipeline that allowed for the alignment and lift-over of sequences through a series of fragmented local alignments.

For the lift-over, we utilized the CHM13 T2T and GRCh38 genomes, including all annotated alternative loci, as templates. We constructed a BLAST database encompassing all these references.

Each lifted sequence was extended by 10,000 base pairs (bps) upstream and downstream to serve as anchor sequences. These sequences, along with the anchors, were then aligned using the local alignment tool BLASTn[1], selecting alignments with over 90% similarity and lengths exceeding 500 bps.

We first merged alignments located on the same reference chromosome within a 10,000 bp range, forming what we refer to as alignment clusters. For each cluster, we created a pseudo-global alignment. For each query region, we selected the local alignment with the highest local alignment score (giving

priority to higher-ranked alignments in case of score ties) and concatenated these to form a pseudo-global alignment. This approach provides a spliced global alignment between the query and the loci corresponding to each alignment cluster, optimizing the total alignment score.

To calculate a global alignment score for each locus within an alignment cluster, we assigned a score of +1 for each matched base and -4 for each mismatched base. Gaps in the query were penalized with a score of -3, plus an additional penalty equal to the gap length. The global score was the sum of these individual scores. Loci with a score greater than zero were considered valid. Queries without valid loci were considered unmapped, and for those with multiple valid loci, the one with the highest score was selected as the most likely location.

However, local tandem duplications and translocations on the query or mapped locus can obscure the precise location of the query. To address this challenge, we performed an additional pairwise global alignment using Stretcher[2] between the query and the mapped locus. This approach allowed us to more accurately pinpoint and annotate the query's location based on the results of the Stretcher alignment.

5 Structural variants (SV) calling based on global pairwise alignment

Annotation of SVs on PAs is important to understand the genetic architecture and global diversity. Using pairwise global alignments, we called SVs on PAs relative the their corresponding reference genes (most similar reference genes if > 90%, else are gene overlap with the lift-over location). Three types of SVs are called, including: deletion, insertion and duplication.

Based on pairwise global alignment, we searched for the gaps larger than 20bps on both query and reference sequences. To avoid large gaps were scattered by small fragments within it, we also merged all gaps within less than 20 bps in distance.

The gaps on query sequences will be annotated as deletions and gaps on reference sequences will be annotated as insertions. If more than 50% of the inserted sequence can be aligned to elsewhere on the reference sequences itself, it will be re-annotated as duplication.

We only annotated SVs with more than 300bps unmasked bases.

6 Determining the optimal transcripts based on exon alignments

Aligning transcripts to each pangenome-allele is essential for annotating diverged paralogs that differ from reference genes in both genomic location and sequence. This annotation is also important for detecting local duplication, distinguishing full gene duplications from exon expansions, and identifying pseudogenes with incomplete transcripts.

We first align all reference exons within the gene group to the pangenome-allele, using only exons with more than 90% similarity to the reference exons. We then assemble the aligned exons into transcripts.

We consider a transcript to be validly assembled if:

1. It contains no redundant exons, so any duplicated exons are treated as new units.

2. It maintains the correct exon order, with no rearrangements.

3. Its overall alignment score equals the total alignment score of all its exons, with a penalty of -100.0 for each missing exon.

Using a greedy strategy, we independently find the highest possible alignment score for each involved transcript in each iteration.

To find the highest possible alignment score, we applied a dynamic programming scheme to determine the global optimal choice for each exon:

1. We let $E_{i,j}$ denote the decision where, at the ith exon on the whole contig, with the last exon on the transcript is its jth exon in the reference.

2. We can conclude this relationship between solutions:

$$E_{i,j} = max \begin{cases} E_{i-1,j-1} & \text{If not include this exon into transcripts,} \\ \max\{E_{i-1,x} \mid x < a\} + S_i + 100 & \text{If include this exon into transcripts,} \\ & S_i \text{ is the alignment score of the } i^{\text{th}} \text{ exon on the whole contig,} \\ & \text{and } a \text{ is the index number of this exon in the reference gene.} \end{cases} \quad (1)$$

3. Start from $E_{0,0} = -100 * \text{exon number}$, and end at the last exon on the contig.

After we finish this iteration for all involved transcripts, we choose the one with the highest score with prior on protein-coding transcripts (scores of protein-coding transcripts will increase by 10 times). We extract this transcript and remove all exons in this transcript from future computation, and continue the next iteration until no transcript could be found.

7 Analysis on the relationship between true integer results and least error non-integer solution

The initial solutin of our genotyping is akin to a akin to a multivariable linear regression, as we stated in methods. Considering the multivariable linear regression solution: $X = argmin_X\{\parallel X \times M - V \parallel\})$, We seek to find a vector X of copy-number of each pangenome allele that minimizes the squared distance to the k-mer counts V we observed in NGS data, using the k-mer matrix as the coefficient matrix.

The regression can be solved with matrix multiplication such that $X = V \times M^+$, where M^+ is the Moore-Penrose pseudoinverse of M.

Suppose the M^T to be $m \times n$ (m k-mer and n alleles, where m >> n), Let $V_1, V_2...V_n$ be m×1 vectors that denote the k-mer counts of individual unknown alleles in the unknown NGS sample. Thus $V = \sum_{i=1..n} V_i$, the sum of all unknown alleles.

By the rule of the association, the regression result $X = V \times M^+ = \sum_{i=1..n}(V_i \times M^+)$. As a result, if the k-mer counts of any arbitrary allele v has high confidence to be projected to its correct genotype, the sum of all alleles V will also have high confidence to be projected to all correct genotypes without the need of phasing the reads. Hence we only discuss the precision for this arbitrary individual allele vector v.

When we are projecting a vector v to known alleles in the data set, let the projection result $x = v \times M^+ = (x_1, x_2...x_n)$, where x_i is the projected coefficient on the ith allele. Because we have
$x = argmin_x\{\parallel x \times M - v \parallel\}$,

$x = argmin_x\{\parallel \sum_{i=1}^{n} \ x_i \times (G_i - \frac{v}{\sum_{i=1..n} x_i}) \ \parallel\}$, similar above, G_i is the ith row in the M, denotes the counts of k-mer in ith allele,.

Because they are all similar alleles, $G_i \approx v$, so $x \times M = \sum_{i=1}^{n} x_i \times G_i \approx (\sum_{i=1}^{n} x_i) * v$, and obviously we have solution on $(\sum_{i=1}^{n} x_i) = 1$ for $x = argmin_x\{\parallel (\sum_{i=1}^{n} x_i) * v - v \parallel\}$, which means we have a good approximation on the correct number of alleles.

We apply $(\sum_{i=1}^{n} x_i) = 1$, getting $x = argmin_x\{\parallel \sum_{i=1}^{n} \ x_i \times (G_i - v) \ \parallel\}$

Let all alleles be either clade into A or B on the binary tree, let G_{A_i} to be the k-mer vector of gene A_i, and G_{B_i} to be the k-mer vector of gene B_i,

We denote:
The mean distance from v to mean of group A, $\sigma_A := G_{\bar{A}} - v$

The mean distance from v to mean of group B, $\sigma_B := G_{\bar{B}} - v$
The distance from A_i to mean of group A, $\sigma_{A_i} := G_{A_i} - \sigma_A$,
The distance from B_i to mean of group B, $\sigma_{B_i} := G_{B_i} - \sigma_B$,

Let $\sigma_B := \sigma_A + \sigma_{\hat{B}}$, where $\sigma_{\hat{B}} := \sigma_B - \sigma_A$, it is the distance from group B to group A.
Let $x_A = \sum_{A_i \in A} x_i$, and $x_B = \sum_{B_i \in A} x_i$

$x = argmin_x\{ \| \sum_{i=1}^{n} \ x_i \times (G_i - v) \ \| \}$
$= argmin_x\{ \ \| \sum_{A_i \in A} \ (x_{A_i} \times (G_{A_i} - v)) \ + \sum_{B_i \in B} \ (x_{B_i} \times (G_{B_i} - v)) \ \| \ \}$
$= argmin_x\{ \ \| \sum_{A_i \in A} x_{A_i} \times \ (\sigma_{A_i} + \sigma_A) + \ \sum_{B_i \in B} x_{B_i} \times \ (\sigma_{B_i} + \sigma_B) \| \}$

Because $\sigma_A, \sigma_{A_1} .... \sigma_{A_n}, \sigma_{B_1} .... \sigma_{B_n}, \sigma_{\hat{B}}$ correspond to independent phylogeny processes, they may be considered as close to orthogonal vectors and $\perp$ to each other. Thus:

$$
\begin{aligned}
x = argmin_x\{ \ \| \sum_{A_i \in A} x_{A_i} \times \ (\sigma_{A_i} + \sigma_A) + \sum_{B_i \in B} x_{B_i} \times \ (\sigma_{B_i} + \sigma_B) \| \} \\
= argmin_x\{ \| \sum_{A_i \in A} (x_{A_i} \times \sigma_{A_i}) + \sum_{A_i \in A} (x_{A_i} \times \sigma_A) + \\
\sum_{B_i \in B} (x_{B_i} \times \ \sigma_{B_i}) + \sum_{B_i \in B} (x_{B_i} \times \ \sigma_{\hat{B}}) + \sum_{B_i \in B} (x_{B_i} \times \sigma_A) \| \} \\
= argmin_x\{ \| \sum_{A_i \in A} (x_{A_i} \times \sigma_{A_i}) + (\sum_{A_i \in A} v_{A_i} + \sum_{B_i \in B} x_{B_i}) \times \sigma_A + \\
\sum_{B_i \in B} (x_{B_i} \times \ \sigma_{B_i}) + \sum_{B_i \in B} (x_{B_i} \times \ \sigma_{\hat{B}}) \| \} \\
= argmin_x\{ [\sum_{A_i \in A} (v_{A_i} \times \sigma_{A_i}) + (\sum_{A_i \in A} x_{A_i} + \sum_{B_i \in B} x_{B_i}) \times \sigma_A + \\
\sum_{B_i \in B} (x_{B_i} \times \ \sigma_{B_i}) + \sum_{B_i \in B} (x_{B_i} \times \ \sigma_{\hat{B}}) \ ]^2 \} \\
= argmin_x\{ (x_A + x_B)^2 \ \times \ \sigma_A^2 + \ x_B^2 \times \sigma_{\hat{B}}^2 + \sum_{A_i \in A} (x_{A_i}^2 \times \sigma_{A_i}^2) + \sum_{B_i \in B} (x_{B_i}^2 \times \sigma_{B_i}^2) \}
\end{aligned}
$$

Because $\sigma_{B_i}^2 << \sigma_{\hat{B}}^2$, so

$$
\sum_{B_i \in B} (x_{B_i}^2 \times \sigma_{B_i}^2) << \sum_{B_i \in B} (x_{B_i}^2 \times \sigma_{\hat{B}}^2) << x_B^2 \times \sigma_{\hat{B}}^2
$$

$$
\begin{aligned}
x \approx argmin_x\{ (x_A + x_B)^2 \ \times \ \sigma_A^2 + \ x_B^2 \times \sigma_{\hat{B}}^2 + \sum_{A_i \in A} (x_{A_i}^2 \times \sigma_{A_i}^2) \} \\
x \approx argmin_x\{ v_B^2 \times \ \sigma_{\hat{B}}^2 + \sum_{A_i \in A} (x_{A_i}^2 \times \sigma_{A_i}^2) \}, where(\sum_{A_i \in A} V_{A_i}) + x_B = 1
\end{aligned}
$$

By the Cauchy–Schwarz inequality,
$x_B^2 \times \ \sigma_{\hat{B}}^2 + \sum_{A_i \in A} (x_{A_i}^2 \times \sigma_{A_i}^2)$ has the minimum value when:

$x_{A_1} : x_{A_2} .... : x_B = \frac{1}{\|\sigma_{A_1}\|} : \frac{1}{\|\sigma_{A_2}\|} : .... \frac{1}{\|\sigma_{\hat{B}}\|}$
$x_A : x_B = (\sum_{A_i \in A} \frac{1}{\|\sigma_{A_1}\|}) : \frac{1}{\|\sigma_{\hat{B}}\|}$

For a sufficient sample set without bias and all alleles follow phylogenetic relationships, we might consider that $\frac{1}{\|\sigma_{\hat{B}}\|}$ will coverage to a positive constant too as the sample size increases as $\sigma_{\hat{B}}$ converges to a positive constant. On the other hand, $(\sum_{A_i \in A} \frac{1}{\|\sigma_{A_1}\|})$ diverges to positive infinite as the distance

to the closest neighbor $\min_{A_i \in A} \|\sigma_{A_1}\|$ converges to 0. Hence, x_A will converge to 1, the correct integer solution.

This means the model will have a good precision to detect known alleles close to the unknown allele close and have a increasing power with the increase of the data size.

References

[1] S.F. Altschul, W. Gish, W. Miller, E.W. Myers, and D.J. Lipman, *Basic local alignment search tool.* Journal of Molecular Biology, Vol. 215, No. 3, pp. 403-410, October 1990. doi:10.1016/S0022-2836(05)80360-2.

[2] P. Rice, I. Longden, and A. Bleasby, *EMBOSS: the European Molecular Biology Open Software Suite.* Trends in Genetics, Vol. 16, No. 6, pp. 276-277, June 2000. doi:10.1016/s0168-9525(00)02024-2.

[3] J. Ebler, P. Ebert, W.E. Clarke, T. Rausch, P.A. Audano, T. Houwaart, Y. Mao, J.O. Korbel, E.E. Eichler, M.C. Zody, A.T. Dilthey, and T. Marschall, *Pangenome-based genome inference allows efficient and accurate genotyping across a wide spectrum of variant classes.* Nature Genetics, Vol. 54, No. 4, pp. 518-525, April 2022. doi:10.1038/s41588-022-01043-w.
