# Supplementary material for: Genotyping sequence-resolved copy-number variation using pangenomes reveals paralog-specific global diversity and expression divergence of duplicated genes
Source: bioRxiv. 2024 Aug 29:2024.08.11.607269. Originally published 2024 Aug 11. Preprint. [Version 2] doi: 10.1101/2024.08.11.607269 (PMC11326217; doi:10.1101/2024.08.11.607269)
Supplement: Supplementary file 1 [file NIHPP2024.08.11.607269V2-supplement-1.pdf]

bioRxiv preprint doi: https://doi.org/10.1101/2024.08.11.607269; this version posted August 29, 2024. The copyright holder for this preprint (which was not certified by peer review) is the author/funder, who has granted bioRxiv a license to display the preprint in perpetuity. It is made available under aCC-BY 4.0 International license.

Online Methods

Constructing pangenome allele database

We initiated our study by identifying gene duplicates in pangenome assemblies. Our pangenome cohort was composed of assemblies from the Human Pangenome Reference Consortium (HPRC) (N=92, excluding HG02080 due abundant flagged regions), the Chinese-Pangenome Consortium (CPC) (N=114), the Human Genome Structural Variation Consortium (HGSVC) (N=18, only Pacbio HiFI assemblies were used), two telomere to telomere diploid assemblies (N=4), and reference genomes (GRCh38 including alternative loci and CHM13 T2Tv1). The gene database used for annotation was GENCODE v39 based on the GRCh38 reference genome.

The initial application of this study was on 3,203 genes known to have copy number variation detected by the HPRC and CPC studies.

We organized genes into gene 'query sets' where each query set encompassed genes with functional or similar sequence including pseudogenes and genes with distant homologies within the same gene family. The query sets were initially defined based on genes with shared name prefixes, and were used to locate copies of duplicated genes within the pangenome.

Direct sequence alignments might overlook sequences such as small pseudogenes and diverged paralogs, potentially creating biases in our genotyping. To address this, we developed a more sensitive alignment scheme to detect all copies of genes in the pangenome. For each query set, we used low-copy *k*-mers (k = 31) that appeared fewer than 255 times in the CHM13 genome, derived from all initial reference genes, to help locate similar genes. We searched for these *k*-mers in each of the pangenome assemblies and references. We then identified *k*-mer hotspots defined as maximal intervals of mapped *k-mers* containing more than 200 *k*-mers within any 1,000-base window within the interval. To aid in mapping small and fragmented pseudogenes, we included an additional criterion to define hotspots: the presence of 50 exonic *k*-mers within the same interval search.

Subsequently, we used BLASTn[1] to refine the boundaries of each hotspot by aligning all reference genes in this query set to each *k*-mer hotspot extended by 10kb flanking sequences.

The *k*-mer defined hotspots include both individual loci mapped by multiple genes from a query set as well as loci with tandemly duplicated genes multi-mapped by individual genes in a query set. To account for this redundancy, we merged alignments that were

bioRxiv preprint doi: https://doi.org/10.1101/2024.08.11.607269; this version posted August 29, 2024. The copyright holder for this preprint (which was not certified by peer review) is the author/funder, who has granted bioRxiv a license to display the preprint in perpetuity. It is made available under aCC-BY 4.0 International license.

less than 10,000 bases apart, causing tandemly duplicated genes to be merged into single loci. To avoid genotyped loci that may be split by recombination, if an intron exceeded 20,000 bases, we divided the locus at the midpoint of the introns. To ensure the overall sequence size was comparable, flanking sequences both upstream and downstream were adjusted to achieve a total length of 15,000 bases. These methods aimed to standardize the size of each sequence to be roughly 30,000 bases, approximating the size of linkage disequilibrium (LD) blocks. The collection of all sequences mapped by a query set are referred to as initial gene-groups.

Definition of gene-groups and *k-mer* list

Because the initial gene-groups were defined from aligned query sets that potentially arbitrarily grouped genes with unrelated sequences based on name, we used subsequent steps of refinement to exclude unrelated sequences.

Initially, for each genome we extracted all *k*-mers exclusive to aligned locations of the initial gene-groups (hence not found elsewhere in the genome). We also filtered out repetitive *k*-mers with more than two-thirds of the *2*-mers and *3*-mers were redundant, as these were mostly associated with highly repetitive DNA, such as Variable Number Tandem Repeats (VNTRs), microsatellites, and transposable elements. Additionally, we excluded *k*-mers demonstrating a high (>70%) or low (<30%) GC content bias[2].

Subsequently, we filtered sequences predominantly composed of the *k*-mers removed in the previous step. The remaining sequences were then categorized into subgroups based on the number of shared *k*-mers. This classification was achieved using graph partitioning. Each sequence was represented as a node, and edges were made between node pairs sharing an excess of 500 unique *k*-mers, except for *NBPF* and *ANKRD* genes, for which a higher threshold of 2,000 unique *k*-mers was set to further reduce the sizes of partitions for computational efficiency in later analysis. Each partition represents a singular gene-group, and the list of unique *k*-mers specific to each gene-group was compiled and termed as '*k*-mer list'.

As an additional filtration, we filtered out genes from the non-confident regions reported by the HPRC, as well as truncated genes from small scaffolds. The genes included needed to be at least 10,000 base pairs away from both ends of a scaffold, except for sequences from genes taken from the reference genomes located at the telomeres.

k-mers based phylogenetic tree construction

We constructed phylogenetic trees for each gene group based on their *k*-mer composition. Initially, for every gene group, we assembled a *k*-mer matrix, M, that

bioRxiv preprint doi: https://doi.org/10.1101/2024.08.11.607269; this version posted August 29, 2024. The copyright holder for this preprint (which was not certified by peer review) is the author/funder, who has granted bioRxiv a license to display the preprint in perpetuity. It is made available under aCC-BY 4.0 International license.

encapsulates all sequences in the gene group. Within this matrix, individual rows represent distinct gene sequences, while each column corresponds to a unique *k*-mer from the *k*-mer list exclusive to the gene group. The matrix cell values are the counts of each *k*-mer present in the respective gene sequence, which is mostly 0 or 1, but occasionally more than 1 when there are low-copy repeated sequences in the gene, or the row represents a tandemly duplicated locus.

The matrix M allows us to measure the concordance between any two sequences, G_i and G_j, by calculating their inner product, denoted as $<G_i * G_j>$. Consequently, the norm matrix, $N = M * M^T$, reflects the *k*-mer concordances for all sequence pairs within the gene group.

We constructed a similarity matrix, S, where $S_{i,j}$ is the cosine similarity of G_i and G_j representing the sequences. The cosine similarity for any two sequences, G_i and G_j can be obtained by normalizing the norm matrix N according to the squares of *k*-mer vectors (approximately equal to sequence lengths) of the sequences in question.

Finally, we used the Unweighted Pair Group Method with Arithmetic Mean (UPGMA) algorithm on the similarity matrix S to generate the phylogenetic tree for each gene group.

Clustering of pangenome alleles into alle-types

We used phylogenetic trees for the annotation and classification of closely related groups of alleles, which we term 'pangenome allele-types'. The classification of pangenome allele-types is guided by two primary criteria applied across all allele-types:

> Homogeneity within allele-types: A allele-type must exhibit near-identical characteristics amongst its members, which is quantified by ensuring the largest *k*-mer distance between any two members does not exceed 155 *k*-mers, which is roughly equivalent to the variation caused by 5 single nucleotide polymorphisms or a structural variation of approximately 95bp, such that allele-types are capable of representing most common variants in about 30kb range.
>
> Distinctiveness of allele-types: Each allele-type must be distinct from its neighboring allele-types. This is measured using a *k*-mer F-statistic score, which must exceed 2 when compared with adjacent allele-types. In cases where allele-types are composed of fewer than three members, the F-statistic may not be reliable; hence, we default this score to 0 for such small allele-types, but change the cutoff of the former criteria to 155 * 3 to detect singleton rare events.

bioRxiv preprint doi: https://doi.org/10.1101/2024.08.11.607269; this version posted August 29, 2024. The copyright holder for this preprint (which was not certified by peer review) is the author/funder, who has granted bioRxiv a license to display the preprint in perpetuity. It is made available under aCC-BY 4.0 International license.

Employing a 'bottom-up' recursive approach starting from leaves, we applied these criteria to all allele-types, aiming to identify and report the largest possible near-identical allele-types. These are later used to identify equivalent loci after genotyping.

Pangenome allele annotation relative to the reference genome

We annotate CNVs events and duplicated alleles in the pangenome assemblies in relative the GRCh38 genome. This requires us to find out the corresponding GRCh38 gene for each pangenome allele. However, this is a known challenging problem of orthology assignment[3].

First, PAs often align to multiple paralogs on GRCh38, and the gene overlap with their liftover locations may not be the most similar reference gene due to gene conversion and translocation (Figs. 1f and 2a). To address this problem, we designed a method to match PAs to their closest GRCh38 genes based on *k*-mer similarity. For every haplotype, we obtained all pairwise similarities between each of its PAs to each of GRCh38 PAs. We matched PAs to their most similar GRCh38 PAs, starting from the most similar pair, until all PAs were matched or failed to match (had no reference gene with >90% similarity). Secondary redundant matches (match to reference genes that had already been matched) were annotated as duplications (distal).

Second, the former failed to match PAs are likely alleles with large SVs, such as insertion, deletion and local proximal duplications. We attempted to lift them back to GRCh38 using their flanking sequences (100kb either side). Because it is challenging to directly liftover genes in the regions with large segmental duplications, we designed this liftover to be a two-stepped liftover. First, we lifted PAs to the region with the best local alignment coverage, allowing SVs to break alignments into smaller units. Second, we performed a global pairwise alignment between PAs and the lifted region to locate the best aligned gene with the presence of local translocations and tandem duplications (Supplementary Methods).

Third, to annotate the proximal duplications mentioned in the last step as well as to annotate diverged paralogs that failed to match from both prior methods, we annotated PAs regarding the gene transcripts. We aligned all exons from the same gene group to PAs, and based on the exon orders and alignment scores, and determined the optimal combinations of transcripts on each PA (Supplementary Methods). The PAs containing no exons were annotated as introns and PAs containing only transcripts of other non-interested genes were annotated as decoys. Introns and decoys were usually filtered out from analysis and the rest PAs are considered as valid alleles, including

bioRxiv preprint doi: https://doi.org/10.1101/2024.08.11.607269; this version posted August 29, 2024. The copyright holder for this preprint (which was not certified by peer review) is the author/funder, who has granted bioRxiv a license to display the preprint in perpetuity. It is made available under aCC-BY 4.0 International license.

pseudogenes that have no intact protein-coding transcripts and putative protein-coding genes with intact protein-coding transcripts.

It is important to note that, because proximal duplication may be highly associated in inheritance and potentially interference with each other functionally such as co-expression (which found between *HP* vs *HPR*, Supplementary Figure 1), and exonic expansion can be found in gene *LPA* and *NBPF,* we treated PAs with proximal duplications as a new type of a single PA, instead of treating them as multiple independent copies of singletons.

Definition of orthologs and paralogs in the pangenome

Based on annotation results, to illustrate the relation of PAs to their corresponding reference genes regarding orthology and sequence similarities, we classified PAs into four categories, including two types of orthologs and two types of paralogs:

1. Reference alleles are alleles in the same allele-type with GRCh38 alleles, representing the alleles almost identical to the reference sequences.
2. Alternative alleles are orthologs located at the same genomic locus as the reference gene but are distinctly in different allele-types from GRCh38 alleles, including alleles that have a list of small variants in strong linkages or alleles that have large structural variations, such as proximal gene/exon duplications or deletions, as observed in genes like *HPR*, *NBPF*, and the *CYP2D6* (star-alleles) gene.
3. Duplicated paralogs (alleles) consisting of paralogs that have been duplicated to different loci from the reference alleles. Despite being translocated, they retain similarities (>80% in *k*-mers) to the reference alleles. These alleles often reflect large, recent segmental duplications in the genome, including similar paralogs, such as *AMY1A*, *AMY1B*, and *AMY1C*, which are still often considered as the same gene despite their distinct locations.
4. Diverged paralogs (alleles) not only differ in their translocation status but also have sequences that are significantly divergent (<80% in *k*-mers) from reference alleles, such that cannot be simply assigned to a single reference gene. These are typically characterized by highly diverse non-reference paralogs, incomplete gene duplications, and novel processed pseudogenes. An illustrative example of diverged paralogs is found among amylase genes, which indicates a proximal translocation event between *AMY1* and *AMY2B* genes.

Genotyping NGS sample with ctyper

bioRxiv preprint doi: https://doi.org/10.1101/2024.08.11.607269; this version posted August 29, 2024. The copyright holder for this preprint (which was not certified by peer review) is the author/funder, who has granted bioRxiv a license to display the preprint in perpetuity. It is made available under aCC-BY 4.0 International license.

The goal of ctyper is to select a list of pangenome alleles and determine their individual copy numbers to represent the CNVs of unknown NGS samples. Instead of sequence alignment, our genotyping is based on *k*-mer comparison, which is not only more efficient but also not affected by misalignments that are frequent in the genomic regions enriched in structural variation and repetitive elements. Another advantage is that there is little bias in *k*-mers between high quality long-reads and NGS data[4], so the *k*-mer data based on assemblies can be applied to predicting NGS data.

The genotyping proceeds per-gene. Given an NGS sample and a *k*-mer matrix M derived from pangenome allele annotation, we generate a vector V for an NGS dataset that includes the counts of each *k*-mer found in the matrix for the NGS sample normalized by the sequencing coverage. We seek to find a vector X of copy-number of each pangenome allele that minimizes the squared distance to the *k*-mer counts we observed in NGS data, e.g. $\mathrm{argmin}_x (\| M^T * X - V \|)$. Compared with absolute distance, squared distance is more suitable for the normal-like noise in NGS data[5,6]. Although it is possible to directly obtain an integer solution using mixed-integer linear programing (MILP), this is NP-hard[7] and can only be used with very few variants/*k*-mers[8,9]. This restricts the use of MILP on the pangenome. The relaxed non-integer solution has an analytic solution which can be efficiently solved for. In essence, the computational problem is akin to a multivariable linear regression. The non-negative least error (NNLS) solution can be further obtained via Lawson-Hanson algorithm[10].

To make the solution closer to the maximum likelihood estimation, during the regression, we rescaled dimensions of *k*-mers to even their expected uncertainty. Assuming the observation of *k*-mer copy number follows negative binomial distribution with the dispersion small enough to be distinct from Poisson[6], the expected variance is roughly proportional to the square of observation, thus we weighted the *k*-mer to the square of the reciprocal of their observed copy number. We also applied smaller weights (adjust=0.05) on singleton *k*-mers (observed in only one PAs and not observed in NGS as well) because they are more likely to be sequencing errors.

The last step is referred to as reversed phylogenetic regression. Our analysis (Supplementary Methods) reveals two strong relationships between NNLS and the integer solutions under a phylogenetic relationship. First, the coefficient on each known allele is inversely proportional to its cosine vector distance to the unknown NGS allele. Hence, in a pangenome with diverse representation, the coefficients of NNLS will be mostly located on the genes that are very similar to the unknown gene. Second, when the coefficients are located on genes that are very similar to the unknown gene, the sum of total coefficients will be very close to the sum of its integer copy number.

bioRxiv preprint doi: https://doi.org/10.1101/2024.08.11.607269; this version posted August 29, 2024. The copyright holder for this preprint (which was not certified by peer review) is the author/funder, who has granted bioRxiv a license to display the preprint in perpetuity. It is made available under aCC-BY 4.0 International license.

Based on the solution's high "convergence" on the phylogenetic tree, we designed a greedy algorithm to efficiently collect non-integer solutions and round it to integer solutions. This algorithm is iterative, employing a bottom-up approach from leaves to root. At each level of the hierarchy, we round the non-integer values to the integer solution with the least overall residual, and propagate the remainder to the next hierarchy. Because at each hierarchy, there are only two remainders from either branch of the tree, this solution is highly efficient.

Trio analysis

Trio analysis is to determine if the genotype combinations of child-father-mother show possible Mendelian violations. When the copy number of a child is 0, the parents need to be 0 or 1; When the copy number of a child is 1, the parents can not both be 0 or both be 2; When the copy number of a child is 2, the parents both need to be 1 or 2. When the copy number of a child is more than 2, the parents need to have the sum to be greater or equal to this number.

Leave-one-out comparison of genotyping results to pangenome assemblies

To find out the extent to which the genotyping results can represent the individual small variants on each PA, we aligned PAs in the original assemblies to their corresponding PAs in the genotyping results.

First, the original assembly PAs were one-to-one paired to genotyped PAs. This pairing was finished by a greedy method. We obtained all pairwise similarities in *k*-mer between each pair of the PAs across original assemblies and genotyping results. Starting from the most similar pair, we paired those alleles without replacement and iterated this until all original assemblies PAs are either paired or failed to be paired (has no genotyped PAs with >90% similarity).

Second, the paired PAs were then aligned using global pairwise alignment tool Stretcher[11]. From the global alignments, we obtained the number of mismatched bases in the unmasked region, where the low copy repeat *k*-mers are used in *k*-mer matrices.

Classification of errors

We classified four types of errors for our benchmarking:

1. False positive: the genotyping results have an additional copy;
2. False negative: the genotyping results have a missing copy;
3. Miss typing: assign a copy to incorrect type;
4. Out of reference: the singleton type among the pangenome and lost reference during leave-one-out.

bioRxiv preprint doi: https://doi.org/10.1101/2024.08.11.607269; this version posted August 29, 2024. The copyright holder for this preprint (which was not certified by peer review) is the author/funder, who has granted bioRxiv a license to display the preprint in perpetuity. It is made available under aCC-BY 4.0 International license.

Benchmarking *CYP2D* genes and visualization

We plotted the benchmarking results on *CYP2D* genes from all 39 HPRC samples with NGS data from the leave-one-out analysis. First, we generated the graphical MSA for all 39 HPRC samples, excluding four original assembly alleles that were filtered due to low quality. Second, because all copy numbers were correctly called, we simply obtained the graphical MSA results of their corresponding genotyped alleles and mapped to the same location. Third, overlapping variants (TP) were colored black, variants missed in the original PAs (FP) were highlighted in red, and variants missed in genotyping results (FN) were highlighted in blue. The overall F1 score was determined to be 94.0%.

In comparison, using the same comparison scheme, we also benchmarked a CYP2D6 genotyping tool, Aldy, with its default settings. We took the phased results of Aldy and matched them to their corresponding original PAs. In a range of about 6 kb, where the variants could be found (first SNP reported at chr22:42126309, last SNP reported at chr22:42132374), Aldy genotyped the variants with an F1 score of 85.2%, and ctyper genotyped the variants with an F1 score of 95.7%. However, it is important to note that our benchmarking strictly requires all variants to be both correctly called and phased, so the “incorrect” variants might still be correctly called by Aldy but incorrectly phased.

Total number of duplication events from genotyping results

Based on ctyper’s genotyping results, we calculated the total number of duplication events for each 1kgp sample, excluding 7 samples due to having extreme values different from the population mean by more than five standard deviations. The total number of each reference gene is measured in each genome and compared to GRCh38 chromosomes excluding alternate haplotypes. Each duplication event is called if the genome has more copy number than twice of GRCh38, excluding decoys/introns and sex chromosome genes. The total number of duplication events is reported for each genome. It is important to note that these duplications also included pseudogenes and small exonic fragments besides known protein-coding genes.

Measuring F-statistic values

Because allele-types may have copy numbers beyond of two and may not be applicable to Fixation index (F_{st}), we instead used F-statistic value to measure the population specificity of allele-types. The F-statistic value is based on the F-test, where we obtained the variances of copy numbers within all continental populations (within-group variance), and use it to divide the variances of copy numbers across different populations (between-group variance).

bioRxiv preprint doi: https://doi.org/10.1101/2024.08.11.607269; this version posted August 29, 2024. The copyright holder for this preprint (which was not certified by peer review) is the author/funder, who has granted bioRxiv a license to display the preprint in perpetuity. It is made available under aCC-BY 4.0 International license.

Relative paralog divergence

Relative paralog divergence (RPD) measures the mean divergences of the paralogs to other alleles, in relative to the mean divergence between only orthologs. RPD was determined for each reference gene and based on the graphic multiple sequence alignments (gMSAs, Supplementary Methods) of PAs assigned to that reference gene as well as ctyper's genotyping results.

First, the divergence value was determined for each pair of PAs assigned to the same reference gene. It was measured based on the alignment scores of unmasked bases (misalignment and gap open = -4, and gap extend = 0, normalized by total alignment length) from gMSAs.

Second, we obtained mean divergence of the orthologs by averaging divergence values between the two PAs from samples with CN = 2.

Third, we then determined the population median copy numbers for each reference gene, and divided samples into those with additional copy numbers (copy numbers more than the median) and those with no additional copy numbers (copy numbers not more than the median).

It is unreliable to directly distinguish the paralog from orthologs due to complex rearrangements (e.g. Figure 2a). To overcome this limitation and only obtain the divergence values from additional copies, we performed statistical estimations based on large populations. We first estimated the mean divergence values from samples with no additional copy numbers and used it as the unit baseline B. When the population median CN = Y, because there are Y(Y-1)/2 pairs, then the total baseline is B * Y(Y-1)/2, which will be subtracted from total divergence values of samples with duplications, and Y(Y-1)/2 will be subtracted from the total number of pairs (the denominator) as well.

After subtracting the total baseline, the mean paralog divergence value of the additional copies were determined for all samples with additional copy numbers. This mean paralog divergence was then normalized by mean divergence of the orthologs obtained in step two.

Multi-allelic linkage disequilibrium

Multi-allelic linkage disequilibrium (mLDs) is an analytic continuation of SNP-based bi-allelic linkage disequilibrium to allow computing linkages between multiple genotypes on neighboring loci. When there are only two genotypes on both loci, mLDs equals LD value. When there are more than two genotypes, mLDs measures LDs between each

bioRxiv preprint doi: https://doi.org/10.1101/2024.08.11.607269; this version posted August 29, 2024. The copyright holder for this preprint (which was not certified by peer review) is the author/funder, who has granted bioRxiv a license to display the preprint in perpetuity. It is made available under aCC-BY 4.0 International license.

pair of genotypes across different loci, and takes the weighted average of all pairs. This weight is the product of both allele frequencies of the pair.

Defining gene-units

We represented each gene by the major transcripts from the MANE (Matched Annotation from NCBI and EMBL-EBI[12]) project. Second, individual exons were aligned. Transcripts were recursively clustered together if they overlapped with previously clustered transcripts with more than 98% overall similarity taking the average similarity of all aligned exons from the transcripts. We call these clusters as gene-units. Third, for each gene-unit, we identified all its exons and looked for unique exons that did not overlap with exons from other gene-units. Fourth, we used these unique exons to represent each gene-unit and filtered out gene-units that have no unique exons (2079 out of 2579 filtered genes were known pseudogenes). Lastly, we assigned PAs to each gene-unit if they contain any of the corresponding unique exons with at least 98% similarity.

Expression correction

For individual tissue analysis, similar to the prior study[13], we logistically corrected the raw TPMs using tool PEER together with the first three principal components obtained from reported genotypes in chr1[14]. For cross-tissue analysis we corrected raw TPMs using DESeq2[15].

Association between CNVs to gene expression

We first associated gene aggregate copy number to expression levels using Pearson correlation (linear-fitting). The p-values and residuals of this fit were recorded. To test if including allele-specific information would improve the correlation, we used the ctyper's pangnome allele-specific copy numbers to replace the aggregate copy numbers to perform multi-variable linear regression using allele-specific copy numbers as dependent variables and gene expression level as independent variables. We compared the residuals of multi-variable linear regression with residuals from Pearson correlation using F-test, and one-tailed p-values of the reduced residual was reported. Both p-values were corrected by the number of gene-units tested (N=3,224).

Linear mixed model

We performed linear mixed modeling to measure the individual expression of each allele-type. We used the total gene expression values as the vector of observed dependent variables, different allele-types as the vector of independent fixed variables and the copy numbers from ctyper genotyping results were used as their coefficient

bioRxiv preprint doi: https://doi.org/10.1101/2024.08.11.607269; this version posted August 29, 2024. The copyright holder for this preprint (which was not certified by peer review) is the author/funder, who has granted bioRxiv a license to display the preprint in perpetuity. It is made available under aCC-BY 4.0 International license.

matrix. The effect sizes of fixed variables were then solved using ordinary least squares (OLS) regression.

Alternative expression of allele-types

To determine whether an allele-type has an alternative expression level compared to other allele-types of the same gene, we merged all other allele-types assigned to the same reference gene into a single variable, separating from the allele-type currently being tested. Additionally, we included other factors, such as paralogs that might also influence total expression, as additional parameters to adjust for their potential interference. For allele-types within solvable matrices with more than 10 non-zero expressions, using a linear mixed model and the R lm function[16], we regressed the expression values to all variables to get their effect sizes. We then compared the effect-size of currently tested allele-type and the effect-size of other allele-types of the same gene using Chi-squared distribution with the linearHypothesis tool[17]. This p-value was then corrected by the number of total allele-types tested (N=18,518).

Across tissue expression comparison

In order to determine if an allele-type has an alternative most expression tissue compared to other allele-types of the same gene, we merged all other allele-types assigned to the same reference gene into a single variable, separating from the currently tested allele-type. Additionally, we included other factors, such as paralogs that might also influence total expression, as additional parameters to adjust for their potential interference. For allele-types within solvable matrices with more than 10 non-zero expressions, we performed linear mixed models to estimate the gene expression level of each allele-type within each of the 57 tissues in GTEx V8. The tissue with the highest expression level was recorded and compared to the tissue with the second highest expression using the Chi-squared test. We then compared the results between currently tested allele-type and all other allele-types of the same gene to see if they had the different highest expressed tissue. When the highest expressed tissues were different, we tested the p-value of either events happening by combining the p-values from both side as p-combined = p1 + p2 - p1 * p2. This p-value was then corrected by the number of allele-types tested on all 57 tissues (N=776,902).

ANOVA (Analysis Of Variance) test on gene expression

We first measured the total expression variance for each eQTL gene-unit, filtering out units with per-sample variance less than 0.1 to exclude genes not sufficiently expressed in the Geuvadis cohort. We estimated experimental noise by measuring expression variance between different trials of the same individuals (mean = 10.5% of the total

bioRxiv preprint doi: https://doi.org/10.1101/2024.08.11.607269; this version posted August 29, 2024. The copyright holder for this preprint (which was not certified by peer review) is the author/funder, who has granted bioRxiv a license to display the preprint in perpetuity. It is made available under aCC-BY 4.0 International license.

variance) and excluded gene-units with experimental noise exceeding 70% of the total variance, resulting in 639 total gene-units on expression. We applied the one-in-ten rule to restrict the number of variants tested to be not greater than 45 (10% of the sample-size) to avoid over-fitting. We filtered out 18 units involving more than 45 PAs; When there were more than 45 known eQTL variants, we used 45 variants with the lowest p-values. The valid expression variance was obtained by subtracting experimental noise from total expression variance. Using ANOVA, we estimated the explained valid variance and adjusted the results by subtracting a baseline, defined as the mean expression variance explained by permuting the orders of all samples (estimated by the mean of 100 trials). If there are no reported eQTL variants, a value of 0 is used for known eQTL variants.

For paCNV, we further investigated the part of variance explained by gene aggreCNs, applying ANOVA to a random matrix with aggreCN information, such that had randomly assigned allele-types, but with the total copy number equal to the original matrix. We subtracted the variance explained by this random matrix from the total explained variance to obtain the variance explained by allele-type information.

bioRxiv preprint doi: https://doi.org/10.1101/2024.08.11.607269; this version posted August 29, 2024. The copyright holder for this preprint (which was not certified by peer review) is the author/funder, who has granted bioRxiv a license to display the preprint in perpetuity. It is made available under aCC-BY 4.0 International license.

1. Altschul, S. F., Gish, W., Miller, W., Myers, E. W. & Lipman, D. J. Basic local alignment search tool. *J. Mol. Biol.* **215**, 403–410 (1990).
2. Ross, M. G. *et al.* Characterizing and measuring bias in sequence data. *Genome Biol.* **14**, R51 (2013).
3. Kirilenko, B. M. *et al.* Integrating gene annotation with orthology inference at scale. *Science* **380**, eabn3107 (2023).
4. Murigneux, V. *et al.* Comparison of long-read methods for sequencing and assembly of a plant genome. *Gigascience* **9**, (2020).
5. Hodson, T. O. Root-mean-square error (RMSE) or mean absolute error (MAE): when to use them or not. *Geosci. Model Dev.* **15**, 5481–5487 (2022).
6. Daley, T. & Smith, A. D. Predicting the molecular complexity of sequencing libraries. *Nat. Methods* **10**, 325–327 (2013).
7. Hartmanis, J. Computers and intractability: A guide to the theory of NP-completeness (Michael R. garey and David S. johnson). *SIAM Rev. Soc. Ind. Appl. Math.* **24**, 90–91 (1982).
8. Numanagić, I. *et al.* Allelic decomposition and exact genotyping of highly polymorphic and structurally variant genes. *Nat. Commun.* **9**, 828 (2018).
9. Ford, M. K. B. *et al.* ImmunoTyper-SR: A computational approach for genotyping immunoglobulin heavy chain variable genes using short-read data. *Cell Syst* **13**, 808–816.e5 (2022).
10. Lawson, C. L. & Hanson, R. J. Back Matter. *Solving Least Squares Problems* 312–337 Preprint at https://doi.org/10.1137/1.9781611971217.bm (1995).
11. Rice, P., Longden, I. & Bleasby, A. EMBOSS: the European Molecular Biology

bioRxiv preprint doi: https://doi.org/10.1101/2024.08.11.607269; this version posted August 29, 2024. The copyright holder for this preprint (which was not certified by peer review) is the author/funder, who has granted bioRxiv a license to display the preprint in perpetuity. It is made available under aCC-BY 4.0 International license.

Open Software Suite. *Trends Genet.* **16**, 276–277 (2000).

12. Morales, J. *et al.* A joint NCBI and EMBL-EBI transcript set for clinical genomics and research. *Nature* **604**, 310–315 (2022).
13. Mohammadi, P., Castel, S. E., Brown, A. A. & Lappalainen, T. Quantifying the regulatory effect size of cis-acting genetic variation using allelic fold change. *Genome Res.* **27**, 1872–1884 (2017).
14. Byrska-Bishop, M. *et al.* High-coverage whole-genome sequencing of the expanded 1000 Genomes Project cohort including 602 trios. *Cell* **185**, 3426–3440.e19 (2022).
15. Love, M. I., Huber, W. & Anders, S. Moderated estimation of fold change and dispersion for RNA-seq data with DESeq2. *Genome Biol.* **15**, 550 (2014).
16. Fox, J. & Weisberg, S. Mixed-effects models in R. *An R Companion to Applied Regression; SAGE: Thousand Oaks, CA, USA* (2002).
17. Fox, J., Weisberg, S. & Price, B. Car: Companion to applied regression. *CRAN: Contributed Packages* The R Foundation https://doi.org/10.32614/cran.package.car (2001).
