# Supplementary material for: High-resolution global diversity copy number variation maps and association with ctyper
Source: bioRxiv. 2024 Aug 11:2024.08.11.607269. Preprint. [Version 1] doi: 10.1101/2024.08.11.607269 (PMC11326217; doi:10.1101/2024.08.11.607269)
Supplement: Supplementary file 1 [file media-1.pdf]

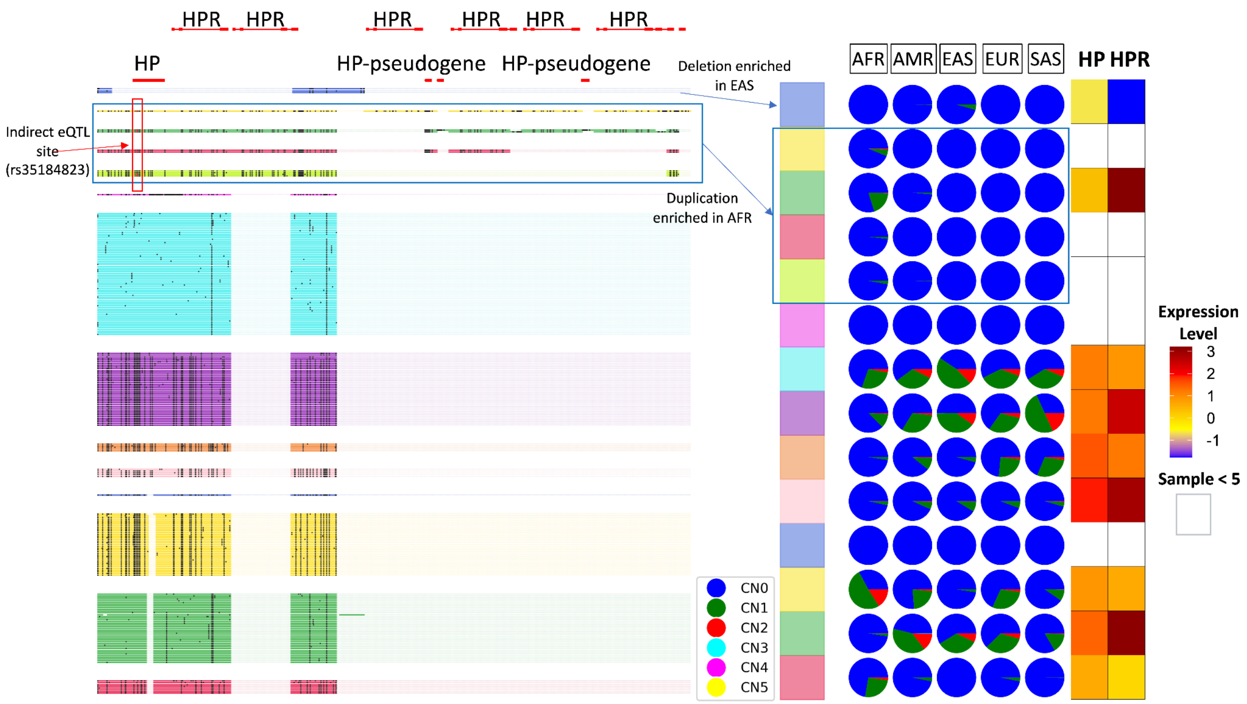

Supplementary Figure 1. Comprehensive case study on *HPR* and *HP* genes. Left: the mutant plot for all PAs on the *HPR* and *HP* locus based on graphic MSA; Proximal duplication and an eQTL site associated with proximal duplications are annotated. Middle: the genotyping results based on 2504 unrelated 1kpg samples. Each pie chart represents the copy number distribution of each allele-type in each population. Right: the expression level for each allele-type estimated from the published GETx whole blood TPM. using linear-mixed-model. The allele-types found in less than 5 samples are excluded. Co-expression has been found between *HPR* and *HP* (t = 24.089, p-value = 6.2e-78, pearson correlation).

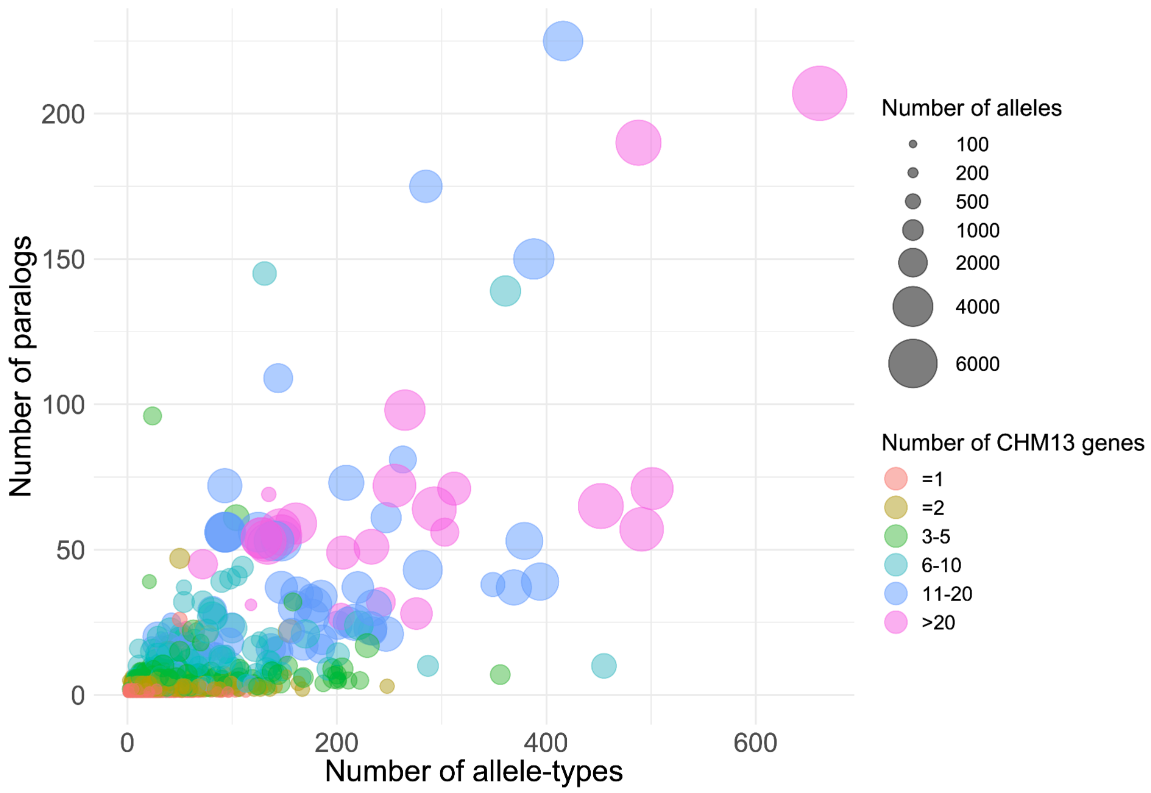

Supplementary Figure 2. The characteristics of all gene groups. Each dot represents each of 3,307 gene groups. The correlation has been found between number of alleles and number of allele-types (t=54.6, p-value = 1e-179), but stronger between number of alleles and number of allele-types (t=72.9, p-value = 1e-219).

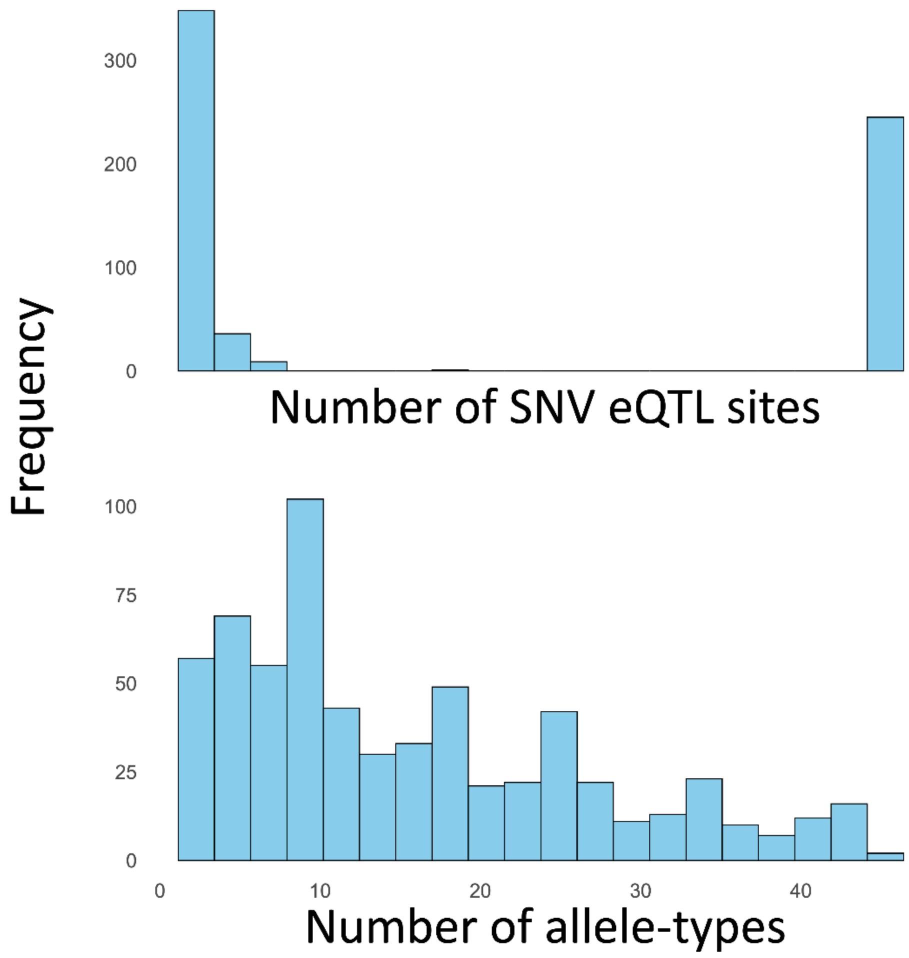

Supplementary Figure 3. The distribution of numbers of SNV eQTL sites (cis) and numbers of allele-type involved in gene expression. The values are based on 639 QTL units used. The units with more than 45 SNV eQTL sites are capped at 45.

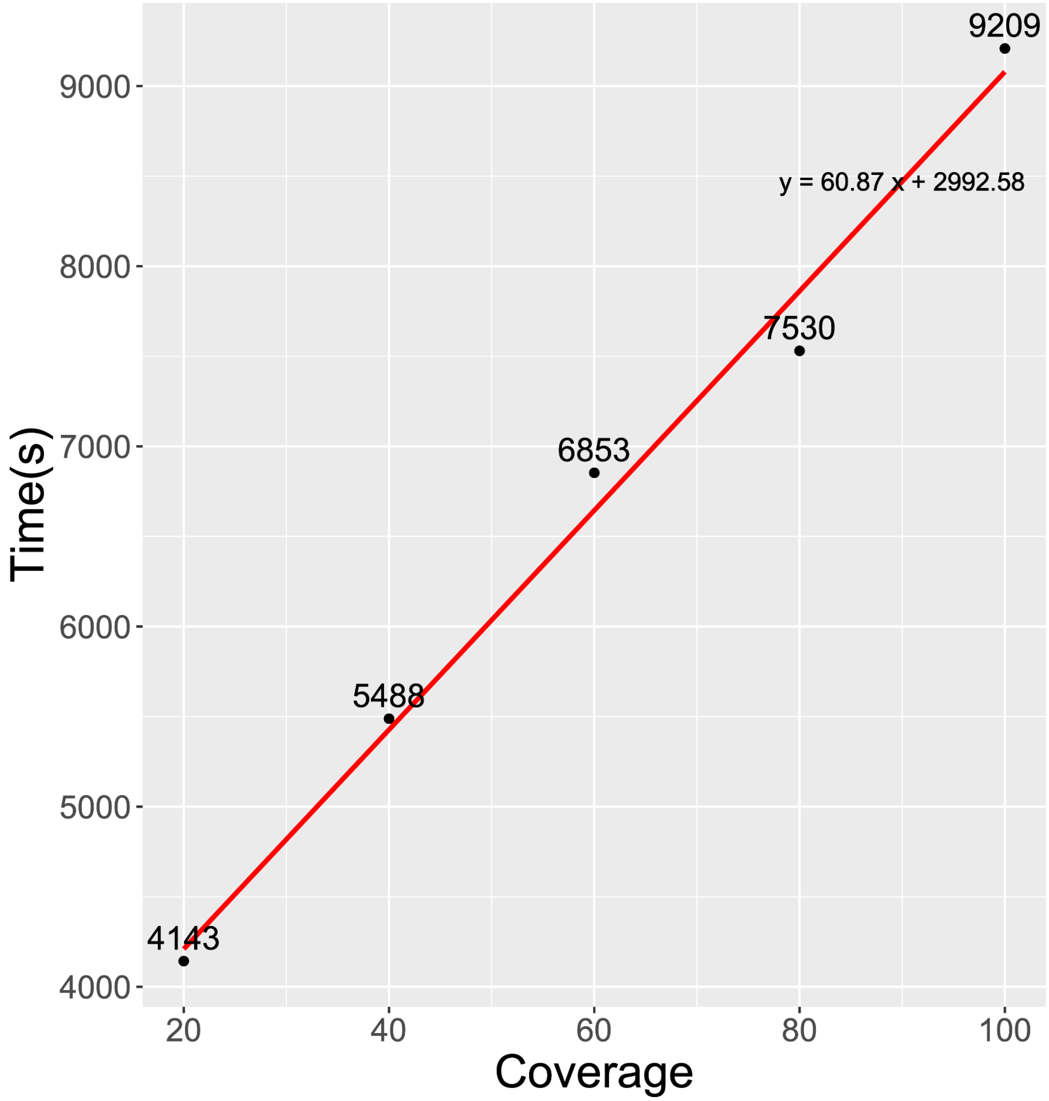

Supplementary Figure 4. The time cost of ctyper on NGS data at different coverages. The same NGS data was downsampled into different coverages and converted to standard cram files. We ran all coverages independently with one thread CPU (AMD EPYC 7513 32-Core Processor) and 16G RAM. We also provide a multiple thread function to let Ctyper be able to process multiple files in parallel using shared memory.

Supplementary methods

August 11, 2024

1 Construction and visualization of graphic multiple sequence alignment

Representing rearrangement, tandem duplication, and translocation events using progressive multiple-sequence alignment is challenging and computationally intensive, particularly for large sets of sequences due to the $O(N^2)$ number of pairwise alignments. To address these challenges, we developed a method that constructs a pseudo multiple-sequence alignment using graph-based alignment, which can be achieved with linear complexity.

Unlike traditional genomic graphs, in this graph alignment, each vertex represents a structural variation breakpoint, and each edge represents the sequence segment between these breakpoints. On each edge, we merge a collection of sequence segments from the query sequences that have high sequence identity and no large (>300 bp) structural variation. With all segments represented or aligned on edges, each query sequence corresponds to a path within this graph.

1.1 Finding the minimum sufficient set of segments to represent all sequences

Given a list of query sequences, we define a collection of segments as a reference set if all query sequences can be fully covered by combinations of local alignments on this collection, provided the alignments have high identity (95%).

The first step in forming a graph-based multiple sequence alignment is to identify a reference set of minimal size. This process begins by initializing the reference set with the largest allele from GRCh38. Subsequently, the remaining sequences are iteratively locally aligned to the reference set. Local alignments with a similarity greater than 95%, spanning at least 300 bases, and containing a minimum of 100 shared k-mers are considered valid. Any portion of the query sequences exceeding 300 bases that lacks a valid alignment is deemed uncovered and is added to the reference set.

After assembling the reference set, we post-process it to identify and exclude internal repeats longer than 300 bases and with 95% identity. Specifically, regions within the reference set that align to any upstream location or prior sequences are removed to prevent redundancy.

1.2 Identifying alignment breakpoints on query sequences to construct pan-gene alignment graph

We then aligned each query sequence to the reference set to identify structural variant breakpoints. Sequences were mapped to the reference set, retaining alignments over 300 bases and 95% identity. The starting and ending points of alignments were recorded on each reference set sequence, and breakpoints were defined as clusters of alignment endpoints within 50 bps.

The vertices of the graph were then defined by the sequences' endpoints and an enumeration of breakpoints. Edges were established based on the reference set segments between corresponding endpoints or breakpoints of the vertices. Consequently, each query sequence corresponded to a path in the graph, which could then be used as a template to align the query sequence with high identity and no structural variation. We refer to this as a sequence path.

We constructed a directed graph using all sequence paths, along with a Start node (S) that connects the start points of all sequence paths, and an End node (E) that connects the end points of all sequence paths. This graph is referred to as the pan-gene alignment graph.

1.3 Linearization of the pan-gene alignment graph

First, in order to represent the repetitive sequences in the graph, we rename the different visits of the same breakpoints as different breakpoints. For example, the second occurrence of the breakpoint $B1$ will be renamed to $B1_2$.

We then records all breakpoints and aim to sort them into a list that has the best overall agreement with the orders in all sequence paths.

1. Starting from the Start node S, we propagate to its most frequent unvisited successor across all sequence paths.
2. We keep propagating and record every new node we visit to the list at the location right after the last visited node, which is important when we re-visit the same node again.
3. If all successors of a node have already been visited, the propagation moves to the End node E, if connected, or returns to the last visited node.
4. When we reach the END node, we re-propagate it to Start node and repeat the cycle until all node has been visited.

The resulting list determines the order of linearization. Using this ordered list of nodes, we identify the corresponding edges (if any) and arrange the associated segments in the determined order. We then assemble these segments to create a template, referred to as the global linear template.

1.4 Pairwise global alignments to determine and visualize small variants

To enhance alignment quality, we re-aligned each query sequence to the pan-gene linear template. We extracted the segments from each query sequence that mapped to the template and assembled them into what we refer to as a local linear template. Global pairwise alignments were then performed between each sequence and its corresponding local linear template. These alignments were subsequently transferred back to the pan-gene linear template. For segments of sequences that did not map to the local linear template, gaps were introduced.

We then assembled all pairwise alignments onto the pan-gene linear template, ensuring that sequence lengths were uniform at locations with insertions or deletions (indels). The result is a graphic multiple sequence alignment (MSA).

Variants within each sequence were readily identified and annotated based on the alignments. These variants were visualized as a mutant plot, providing a clear and informative overview of genetic diversity, including both structural variations and small variants, across different sequences.

2 k-mer counting, normalization and bias correction in NGS data

During genotyping of NGS samples, we count all k-mers in each gene group's k-mer list. Specifically, we encode 31-mers as 64-bit integers for hashing purposes. We selected approximately one million random k-mers from all exons of protein-coding genes to establish background sequencing coverage.

We acknowledge that certain genomic regions, such as the HLA-DRB gene—previously identified as the most problematic in other studies[3]—exhibit strong batch effects. The bias overall increases the coverage of NGS data in this region and might potentially cause false positive calling of duplications and false negative of detecting deletions.

To address this, we provide a function for bias correction. We benchmark individual k-mers against long-read assemblies, which serve as the golden standard for true copy number determination. To identify k-mers with the lowest confidence, we calculate a posterior P-value for each k-mer's occurrence based on the Poisson distribution of ground-truth copy numbers. The k-mers with more than 15% of samples (6 out of 40) exhibiting a P-value below 0.05 were flagged. We assessed the average ratio discrepancies between ground-truth and observed copy numbers across 40 NGS samples, applying ratio corrections to the k-mers with low confidence.

After background normalization and bias correction, a k-mer vector V is obtained, denoting the most likely integer copy numbers of all k-mers in the original genome.

The bias correction we performed largely reduced the Hardy Weinberg disequilibrium of HLA-DRB genotyping, from 34.3% (48 out of 140) to no Hardy Weinberg disequilibrium.

However, the underlying cause of this bias remains to be investigated, and we observed this bias in both cell-line and non-cell line NGS samples.

3 Detection of novel partial duplication and deletion below the scale of pangenome-alleles

To detect novel partial gene duplications and deletions that have not been previously observed in the pangenome cohorts and are of much smaller scale than pangenome alleles, we designed an auxiliary function in ctyper that utilizes k-mer coverages.

Recall that all pangenome alleles (PAs) are included in the graphic MSAs by the minimum sufficient sets of segments. We annotate the k-mers that occupy exclusive positions (i.e., k-mers found only in one segment and at a single position on that segment) and record these in our database.

After completing genotyping, we calculate the coverage ratio for each k-mer using the solution. The coverage ratio is defined as the ratio between the expected k-mer count from genotyping and the observed k-mer count in NGS data. We average the coverage ratios within each window on each segment (default window size = 60). This coverage can be further averaged over larger scales; we recommend using a scale of at least 6K.

These coverage patterns can also be visualized as a dot plot, which allows us to identify and visualize fragmented gene duplication and deletion events.

However, due to the rarity and difficulty of detecting these events, benchmarking the results is challenging. It is often difficult to distinguish real events from misassemblies, making comprehensive benchmarking infeasible. Nonetheless, this method can also serve as a tool for correcting errors in genotyping. It is estimated to reduce discrepancies from a *de novo* assembly (CN1) by 15% for events larger than 6K.

4 Two step alignments for better lift-over of CNV genes

Our phylogenetic-based annotation and classification relies on sequence similarities among different pangenome alleles and are independent of their specific genomic locations. However, understanding these locations is important for distinguishing between paralogs and orthologs, especially in cases involving highly mutated sequences.

While the Human Pangenome Reference Consortium (HPRC) and the Human Genome Structural Variation Consortium (HGSVC) have previously conducted their own lift-over processes and analyses, these efforts may not be sufficient for accurate ortholog identification. This limitation is largely due to the fact that copy number variable genes often reside in segmental duplication regions, which are susceptible to structural variations and repetitive sequences. Aligning these genes to a reference genome can result in fragmented alignments and multiple high-confidence alignments, presenting significant challenges for conventional sequence aligners.

To overcome these challenges, we developed a highly sensitive, multi-step alignment pipeline that allows for the alignment and lift-over of sequences through a series of fragmented local alignments.

For this lift-over, we utilized the CHM13 T2T and GRCh38 genomes, including all annotated alternative loci, as templates. We constructed a BLAST database encompassing all these references.

Each lifted sequence was extended by 10,000 base pairs (bps) upstream and downstream to serve as anchor sequences. These sequences, along with the anchors, were then aligned using the local alignment tool BLASTn[1], selecting alignments with over 90% similarity and lengths exceeding 500 bps.

We first merged alignments located on the same reference chromosome within a 10,000 bp range, forming what we refer to as alignment clusters. For each cluster, we created a pseudo-global alignment. For each query region, we selected the local alignment with the highest local alignment score (giving priority to higher-ranked alignments in case of score ties) and concatenated these to form a pseudo-global alignment. This approach provides a spliced global alignment between the query and the loci corresponding to each alignment cluster, optimizing the total alignment score.

To calculate a global alignment score for each locus within an alignment cluster, we assigned a score of +1 for each matched base and -4 for each mismatched base. Gaps in the query were penalized with a score of -3, plus an additional penalty equal to the gap length. The global score is the sum of these individual scores. Loci with a score greater than zero were considered valid. Queries without valid loci were considered unmapped, and for those with multiple valid loci, the one with the highest score was selected as the most likely location.

However, local tandem duplications and translocations on the query or mapped locus can obscure the precise location of the query. To address this challenge, we performed an additional pairwise global alignment using Stretcher[2] between the query and the mapped locus. This approach allowed us to more accurately pinpoint and annotate the query's location based on the results of the Stretcher alignment.

5 Determine the optimal transcripts based on exon alignments

Aligning transcripts to each pangenome-allele is essential for annotating diverged paralogs that differ from reference genes in both genomic location and sequence. This process is important for determining local duplications, distinguishing full gene duplications from exon expansions, and identifying pseudogenes with incomplete transcripts.

We first aligned all reference exons within the gene group to the pangenome-allele, using only exons with more than 90% similarity to the reference exons. We then assembled the aligned exons into transcripts.

We consider a transcript to be validly assembled if:
1.It contains no redundant exons, so any duplicated exons are treated as new units.
2. It maintains the correct exon order, with no rearrangements.
3. Its overall alignment score equals the total alignment score of all its exons, with a penalty of -100.0 for each missing exon.

Using a greedy strategy, we independently find the highest possible alignment score for each involved transcript in each iteration.

To find the highest possible alignment score, we applied a dynamic programming scheme to determine the global optimal choice for each exon:
We let $E_{i,j}$ represent the solution where, at the ith exon on the whole contig, the last exon on the transcript is its jth exon in the reference.
2. We can conclude the relation:

$$E_{i,j} = \begin{cases} E_{i-1,j-1} & \text{if not include this exon into transcripts,} \\ \max\{E_{i-1,x} \mid x < a\} + S_i + 100 & \text{where } S_i \text{ is the alignment score of the } i\text{th exon on the whole contig,} \\ & \text{and } a \text{ is the index number of this exon in the reference gene.} \end{cases} \quad (1)$$

3. Start from $E_{0,0} = -100 * \text{exon number}$, and end at the last exon on the contig.

After we finished this iteration for all involved transcripts, we choose the one with the highest score and prior on protein-coding transcripts (protein-coding transcripts will increase by 10 times). We extract this transcript and remove all exons in this transcript from future computation, and continue the next iteration until no transcript could be found.

6 Mathematic analysis on the relationship between true integer results and least error non-integer solution

Let $V_1, V_2 ... V_n$ are m×1 vectors that denote individual unknown alleles in the unknown sample. Thus $V = \sum_{i=1..n} V_i$, the sum of all unknown alleles. By the rule of the association, the regression result

$X = V \times Q = \sum_{i=1..n}(V_i \times Q)$. As a result, if every individual k-mer vector v of an arbitrary allele has a high confident to be projected to its correct genotype, the sum of all alleless V will also have high confidence to be projected to all correct genotypes without the need of phasing the reads.

When we are projecting v to known alleles in the data set, let the projection result $x = v \times Q = (x_1, x_2...x_n)$, where x_i is the projected coefficient on the ith allele. Because we have
$x = argmin_x\{ \parallel x \times M - v \parallel \}$,

$x = argmin_x\{ \parallel \sum_{i=1}^{n} \ \ x_i \times (G_i - \frac{v}{\sum_{i=1..n} x_i}) \ \ \parallel\}$, similar above, G_i is the counts of all k-mers in ith allele.

Because they are all similar alleles, $G_i \approx v$, so $x \times M \approx \ (\sum_{i=1}^{n} x_i) * v$, and $(\sum_{i=1}^{n} x_i) \approx x \times M \times v^{-1}$, which means we have a good approximation on the total number of alleles.
$x = argmin_x\{ \parallel \sum_{i=1}^{n} \ \ x_i \times (G_i - v) \ \ \parallel\}$

Let all alleles be either clade into A or B on the binary tree, let G_{A_i} to be the k-mer vector of gene A_i, and G_{B_i} to be the k-mer vector of gene B_i,

we denote:
the mean distance from v to group A, $\sigma_A := G_{\bar{A}} - v$
the mean distance from v to group B, $\sigma_B := G_{\bar{B}} - v$
the distance from A_i to mean of group A, $\sigma_{A_i} := G_{A_i} - \sigma_A$,
the distance from B_i to mean of group B, $\sigma_{B_i} := G_{B_i} - \sigma_B$,

let $\sigma_B := \sigma_A + \sigma_{\hat{B}}$, where $\sigma_{\hat{B}} := \sigma_B - \sigma_A$, it is the distance from group B to group A.
let $x_A = \sum_{A_i \in A} x_i$, and $x_B = \sum_{B_i \in A} x_i$

$x = argmin_x\{ \parallel \sum_{i=1}^{n} \ \ x_i \times (G_i - v) \ \ \parallel\}$
$= argmin_x\{ \ \parallel \sum_{A_i \in A} \ \ (x_{A_i} \times (G_{A_i} - v)) \ + \sum_{B_i \in B} \ \ (x_{B_i} \times (G_{B_i} - v)) \ \ \parallel \}$
$= argmin_x\{ \parallel \sum_{A_i \in A} x_{A_i} \times \ \ (\sigma_{A_i} + \sigma_A) + \ \sum_{B_i \in B} x_{B_i} \times \ \ (\sigma_{B_i} + \sigma_B) \parallel\}$

Because $\sigma_A, \sigma_{A_1}....\sigma_{A_n}, \sigma_{B_1}....\sigma_{B_n}, \sigma_{\hat{B}}$ correspond to independent phylogeny processes, they may be considered as close to orthogonal vectors and $\perp$ to each other. Thus:

$$
\begin{aligned}
x = argmin_x\{ \| \sum_{A_i \in A} x_{A_i} \times \ (\sigma_{A_i} + \sigma_A) + \sum_{B_i \in B} x_{B_i} \times \ \ (\sigma_{B_i} + \sigma_B) \| \} \\
= argmin_x\{\| \sum_{A_i \in A} (x_{A_i} \times \sigma_{A_i}) + \sum_{A_i \in A} (x_{A_i} \times \sigma_A) + \\
\sum_{B_i \in B} (x_{B_i} \times \ \sigma_{B_i}) + \sum_{B_i \in B} (x_{B_i} \times \ \sigma_{\hat{B}}) + \sum_{B_i \in B} (x_{B_i} \times \sigma_A) \| \} \\
= argmin_x\{\| \sum_{A_i \in A} (x_{A_i} \times \sigma_{A_i}) + (\sum_{A_i \in A} v_{A_i} + \sum_{B_i \in B} x_{B_i}) \times \sigma_A + \\
\sum_{B_i \in B} (x_{B_i} \times \ \sigma_{B_i}) + \sum_{B_i \in B} (x_{B_i} \times \ \sigma_{\hat{B}})) \| \} \\
= argmin_x\{(\sum_{A_i \in A} (v_{A_i} \times \sigma_{A_i}) + (\sum_{A_i \in A} x_{A_i} + \sum_{B_i \in B} x_{B_i}) \times \sigma_A + \\
\sum_{B_i \in B} (x_{B_i} \times \ \sigma_{B_i}) + \sum_{B_i \in B} (x_{B_i} \times \ \sigma_{\hat{B}})))^2 \} \\
= argmin_x\{(x_A + x_B)^2 \ \times \ \sigma_A^2 + \ x_B^2 \times \sigma_{\hat{B}}^2 + \sum_{A_i \in A} (x_{A_i}^2 \times \sigma_{A_i}^2) + \sum_{B_i \in B} (x_{B_i}^2 \times \sigma_{B_i}^2) \}
\end{aligned}
$$

Because $\sigma_{B_i}^2 << \sigma_{\hat{B}}^2$, so

$$
\sum_{B_i \in B} (x_{B_i}^2 \times \sigma_{B_i}^2) << \sum_{B_i \in B} (x_{B_i}^2 \times \sigma_{\hat{B}}^2) << x_B^2 \times \sigma_{\hat{B}}^2
$$

$$
x \approx argmin_x\{(x_A + x_B)^2 \ \times \ \sigma_A^2 + \ x_B^2 \times \sigma_{\hat{B}}^2 + \sum_{A_i \in A} (x_{A_i}^2 \times \sigma_{A_i}^2) \}
$$

$$
x \approx argmin_{x,(\sum_{A_i \in A} V_{A_i}) + x_B = 1}\{v_B^2 \times \ \sigma_{\hat{B}}^2 + \sum_{A_i \in A} (x_{A_i}^2 \times \sigma_{A_i}^2) \}
$$

By the Cauchy–Schwarz inequality,
$x_B^2 \times \ \sigma_{\hat{B}}^2 + \sum_{A_i \in A} (x_{A_i}^2 \times \sigma_{A_i}^2)$ has the minimum value when:

$x_{A_1} : x_{A_2} .... : x_B = \frac{1}{\|\sigma_{A_1}\|} : \frac{1}{\|\sigma_{A_2}\|} : .... \frac{1}{\|\sigma_{\hat{B}}\|}$
$x_A : x_B = (\sum_{A_i \in A} \frac{1}{\|\sigma_{A_1}\|}) : \frac{1}{\|\sigma_{\hat{B}}\|}$

For a sufficient sample set without bias and all alleles follow phylogenetic relationships, we might consider that $\frac{1}{\|\sigma_{\hat{B}}\|}$ will coverage to a positive constant too as the sample size increases as $\sigma_{\hat{B}}$ converges to a positive constant. On the other hand, $(\sum_{A_i \in A} \frac{1}{\|\sigma_{A_1}\|})$ diverges to positive infinite as the distance to the closest neighbor $\min_{A_i \in A} \|\sigma_{A_1}\|$ converges to 0. Hence, x_A will converge to 1, the correct integer solution.

This means the model will have a good precision to detect known alleles close to the unknown allele close and have a increasing power with the increase of the data size.

References

[1] S.F. Altschul, W. Gish, W. Miller, E.W. Myers, and D.J. Lipman, *Basic local alignment search tool.* Journal of Molecular Biology, Vol. 215, No. 3, pp. 403-410, October 1990. doi:10.1016/S0022-2836(05)80360-2.

[2] P. Rice, I. Longden, and A. Bleasby, *EMBOSS: the European Molecular Biology Open Software Suite.* Trends in Genetics, Vol. 16, No. 6, pp. 276-277, June 2000. doi:10.1016/s0168-9525(00)02024-2.

[3] J. Ebler, P. Ebert, W.E. Clarke, T. Rausch, P.A. Audano, T. Houwaart, Y. Mao, J.O. Korbel, E.E. Eichler, M.C. Zody, A.T. Dilthey, and T. Marschall, *Pangenome-based genome inference allows efficient and accurate genotyping across a wide spectrum of variant classes.* Nature Genetics, Vol. 54, No. 4, pp. 518-525, April 2022. doi:10.1038/s41588-022-01043-w.
